# Structural Optimization and Biological Activity of Pyrazole Derivatives: Virtual Computational Analysis, Recovery Assay and 3D Culture Model as Potential Predictive Tools of Effectiveness against *Trypanosoma cruzi*

**DOI:** 10.3390/molecules26216742

**Published:** 2021-11-08

**Authors:** Lorraine Martins Rocha Orlando, Guilherme Curty Lechuga, Leonardo da Silva Lara, Byanca Silva Ferreira, Cynthia Nathalia Pereira, Rafaela Corrêa Silva, Maurício Silva dos Santos, Mirian Claudia S. Pereira

**Affiliations:** 1 Laboratório de Ultraestrutura Celular, Instituto Oswaldo Cruz, Fiocruz, Av. Brasil 4365, Manguinhos, Rio de Janeiro 21040-900, RJ, Brazil; lorrainemartins07@hotmail.com (L.M.R.O.); guilherme.lechuga@yahoo.com.br (G.C.L.); leonardosilva.lara@hotmail.com (L.d.S.L.); 2 Laboratório de Síntese de Sistemas Heterocíclicos (LaSSH), Instituto de Física e Química (IFQ), Universidade Federal de Itajubá, Av. BPS 1303, Pinheirinho, Itajubá 37500-903, MG, Brazil; byancaferreira@outlook.com (B.S.F.); cynthianathpereira@gmail.com (C.N.P.); rafaelacorrea13@gmail.com (R.C.S.); mauriciosantos@unifei.edu.br (M.S.d.S.)

**Keywords:** *Trypanosoma cruzi*, pyrazole derivatives, trypanocidal activity, 3D culture model

## Abstract

Chagas disease, a chronic and silent disease caused by *Trypanosoma cruzi*, is currently a global public health problem. The treatment of this neglected disease relies on benznidazole and nifurtimox, two nitroheterocyclic drugs that show limited efficacy and severe side effects. The failure of potential drug candidates in Chagas disease clinical trials highlighted the urgent need to identify new effective chemical entities and more predictive tools to improve translational success in the drug development pipeline. In this study, we designed a small library of pyrazole derivatives (44 analogs) based on a hit compound, previously identified as a *T. cruzi* cysteine protease inhibitor. The in vitro phenotypic screening revealed compounds **3g**, **3j**, and **3m** as promising candidates, with IC_50_ values of 6.09 ± 0.52, 2.75 ± 0.62, and 3.58 ± 0.25 µM, respectively, against intracellular amastigotes. All pyrazole derivatives have good oral bioavailability prediction. The structure–activity relationship (SAR) analysis revealed increased potency of 1-aryl-1*H*-pyrazole-imidazoline derivatives with the Br, Cl, and methyl substituents in the *para*-position. The **3m** compound stands out for its trypanocidal efficacy in 3D microtissue, which mimics tissue microarchitecture and physiology, and abolishment of parasite recrudescence in vitro. Our findings encourage the progression of the promising candidate for preclinical in vivo studies.

## 1. Introduction

Chagas disease, caused by the protozoan *Trypanosoma cruzi*, is among the 20 neglected tropical diseases combated by the World Health Organization [1]. This century-old disease, also known as American trypanosomiasis, remains endemic in 21 countries in Latin America but has become globalized due to the migratory flow of infected individuals to different continents [2]. Currently, it is estimated that this disease affects 6–7 million people worldwide with 10,000 deaths per year and 75 million people at risk of contracting the disease [3]. Acute *T. cruzi* infection, which lasts for 4–8 weeks, is usually asymptomatic and undiagnosed, but mild symptoms such as fever, headache, anorexia, hepatomegaly, splenomegaly and tachycardia may occur. The chronic phase is mostly asymptomatic (indeterminate form) in 60–70% of cases. However, this silent disease may progress to cardiomyopathy (30–40%) as well as digestive (megacolon and megaesophagus; 10%) and neurological (10%) manifestations [4,5,6]. Heart failure and sudden death, caused by the damage of the heart muscle and its nervous system, are important outcomes of chronic Chagas cardiomyopathy (CCC).

So far, little progress has been made in the treatment of Chagas disease, which remains based on benznidazole (Bz) and nifurtimox (Nif). These drugs, discovered in the 1960s, have partial efficacy in the acute phase (60–70% efficacy), due to *T. cruzi* natural resistant strains, and low effectiveness in the chronic phase in addition to serious side effects [7]. The Benznidazole Evaluation for Interrupting Trypanosomiasis (BENEFIT) clinical trial highlighted Bz′s failure to prevent chronic chagasic cardiomyopathy progression during a five-year follow up [8]. A great expectation was projected on the potential of CYP51 inhibitors, such as posaconazole and ravuconazole prodrug (E1224) for the treatment of the disease, but the results of Chagazol and Stop Chagas clinical trials have shown no efficacy of posaconazole in monotherapy [9] or combined therapy with Bz [10] as also reported for ravuconazole [11,12]. The Benznidazole New Doses Improved Treatment and Therapeutic Associations (BENDITA) clinical trial shed light on the effectiveness of Bz at lower concentrations and reduced treatment regimens [12], favoring treatment adherence due to lower side effects. Despite the promising result of BENDITA, the search for safe and effective drugs in both stages of the disease is still urgently needed.

In the last few decades, rational drug design has been intensified in an attempt to identify more selective and effective compounds against *T. cruzi*. Although the compound potency is considered an important driving force, other favorable features such as prediction of the physicochemical and pharmacokinetic properties contribute to the success of the optimization of the hit-to-lead process. Recently, we have reported 5-amino-1-aryl-4-(4,5-dihydro-1*H*-imidazol-2-yl)-1*H*-pyrazoles derivatives as a potential *T. cruzi* cysteine protease inhibitor [13]. Hydrophobic and hydrogen bond interactions of the pyrazole-imidazoline derivatives within the active site of cruzipain, revealed by molecular docking, induced more than 50% inhibition of *T. cruzi* cysteine protease. Cruzipain, a key enzyme implicated in parasite invasion [14], intracellular replication and differentiation [15,16], as well as host immune system evasion [17], is a relevant molecular target for anti-*T. cruzi* drug design. Low tolerability in primates and dogs of the vinyl sulfone irreversible inhibitor of cruzipain (K777), a promising candidate for Chagas disease treatment, led to discontinuity of the preclinical assays [18]. The adverse reaction was controlled by neutralizing the pH of the drug solution and allowed for the conclusion of the study with 28 days of treatment with funding from the European Union, highlighting the potential of K777 in the treatment of parasitic infections [19]. Several reversible cruzipain inhibitors, odanacatib analogs containing a nitrile moiety, have shown potent in vitro anti-*T. cruzi* activity [20] and high cure rates (90%) in a murine model of *T. cruzi* infection [21]. Phenotypic screening of GlaxoSmithKline human African trypanosomiasis (HAT) and Chagas chemical boxes led to the identification of novel cruzipain inhibitors scaffolds [22]. Moreover, based on drug repurposing criteria, 3180 approved FDA drugs were virtually searched as a cruzipain inhibitor, highlighting four selected drugs (etofylline clofibrate, piperacillin, cefoperazone, and flucloxacillin) with potent in vitro antiparasitic activity but low efficacy in vivo [23].

Herein, we focus on drug optimization as a strategy for improving trypanocidal effectiveness and drug-likeness of the pyrazole-imidazoline derivatives, previously identified as hit compounds [13]. The introduction of more predictive experimental models in the phenotypic screening platform, evaluating drug efficacy in the microtissue and parasite recrudescence in vitro, contributed to the identification of a potential anti-*T. cruzi* candidate.

## 2. Results and Discussion

### 2.1. Drug Design and Synthesis

**Figure 1:**
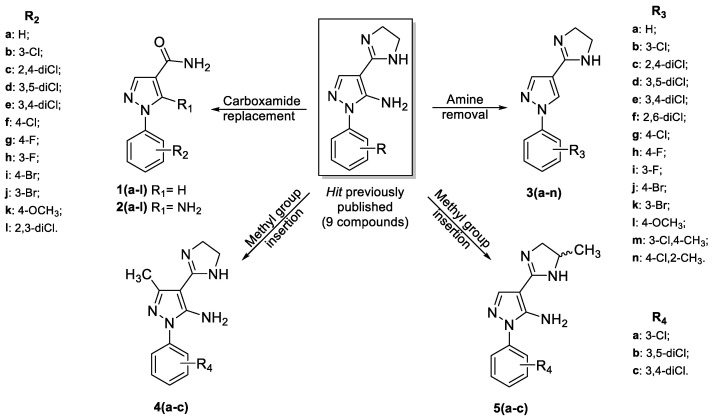
Rational planning of **1**(**a**–**l**), **2**(**a**–**l**), **3**(**a**–**n**), **4**(**a**–**c**), and **5**(**a**–**c**) from the hit previously published.

This study focused on the optimization of hit compounds, 5-amino-1-aryl-4-(4,5-dihydro-1*H*-imidazol-2-yl)-1*H*-pyrazole, previously identified by the group [13], capable of binding cruzipain and partially inhibiting (>50%) the cysteine protease activity of *T. cruzi*. Changes in the hit compounds were designed based on the compound 3H5 (*N*-(1*H*-benzoimidazol-2-yl)-1,3-dimethyl-1*H*-pyrazole-4-carboxamide), identified in the Protein Data Bank (PDB) as a cruzipain inhibitor [24]. Considering that the carboxamide-containing region (CONH_2_) of this compound interacts strongly with the active site of cruzipain, the imidazoline ring of the hit compound was replaced by carboxamide, series **1**(**a**–**l**) and **2**(**a**–**l**), in attempt to potentiate the inhibition of cysteine protease and increase the trypanocidal activity (Figure 1). The deaminated pyrazole-imidazoline derivatives **3**(**a**–**n**) were planned from the removal of the amine group bonded to pyrazole ring of the hit compound (Figure 1). The best trypanocidal activity of the hit was achieved with *m*-chloro substituted compounds: 3-Cl, 3,4-diCl, and 3,5-diCl [13]. Therefore, analog compounds with methyl group were also outlined: series **4**(**a**–**c**) and **5**(**a**–**c**) (Figure 1).

The optimization of the pyrazole-imidazoline compound with the addition of amino and methyl substituents aimed to modulate the molecule polarity. Amines are highly reactive due to their basicity and nucleophilicity and can also interfere with the compound water solubility. Furthermore, the addition of 3-methyl to the pyrazole ring (series 4) or the imidazoline ring (series 5) can improve the biological activity of the compounds, favoring their interaction with hydrophobic domains at the protein binding site [25]. Finally, electron donor and acceptor substituents, including halogens as well as methyl and methoxy groups, were inserted in different positions of the benzene ring to modulate the electrostatic interactions that have great relevance in molecular recognition (ligand-target biomolecule). 

**Scheme 1:**
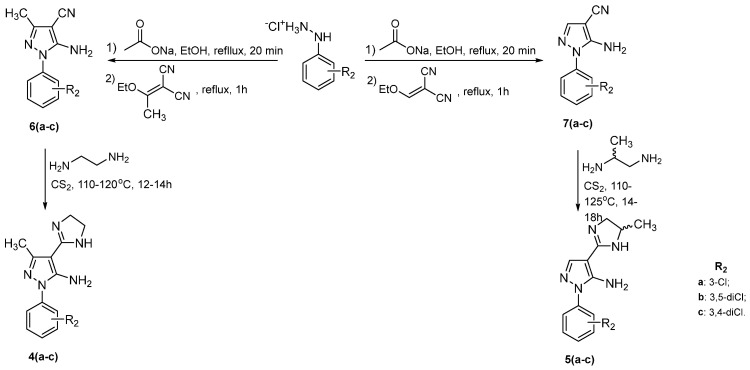
Synthetic route to obtain **4**(**a**–**c**) and **5**(**a**–**c**).

The pyrazole-carboxamides **1**(**a**–**l**) and **2**(**a**–**l**) were synthesized as previously reported by our research group, except **1c**, **1l**, and **2c**, which have been prepared for the first time, using methodology quite similar [26]. Recently, the synthesis of pyrazole-imidazoline **3**(**a**–**n**) was optimized and published by our group employing microwave irradiation [27]. The same methodology was utilized in this work. The new compounds **4**(**a**–**c**) and **5**(**a**–**c**) were obtained in two steps (Scheme 1).

Firstly, arylhydrazine hydrochlorides reacted with sodium acetate, under reflux, for 20 min. After that, 1-ethoxyethylidenemalononitrile or ethoxymethylenemalononitrile was added to give the corresponding key intermediates **6**(**a**–**c**) and **7**(**a**–**c**), respectively, after 1–2 h, under reflux [28,29]. Compounds **6**(**a**–**c**) were treated with ethylenediamine and carbon disulfide, at 110–120 °C for 12–14 h, to give the targets **4**(**a**–**c**), in 63–89% yield. Finally, compounds **5**(**a**–**c**) were synthesized through the reaction involving **7**(**a**–**c**), 1,2-diaminopropane, carbon disulfide, at 110–125 °C for 14–18 h, in 24–80% yield [30]. 

### 2.2. Physicochemical Properties Prediction

**Figure 2:**
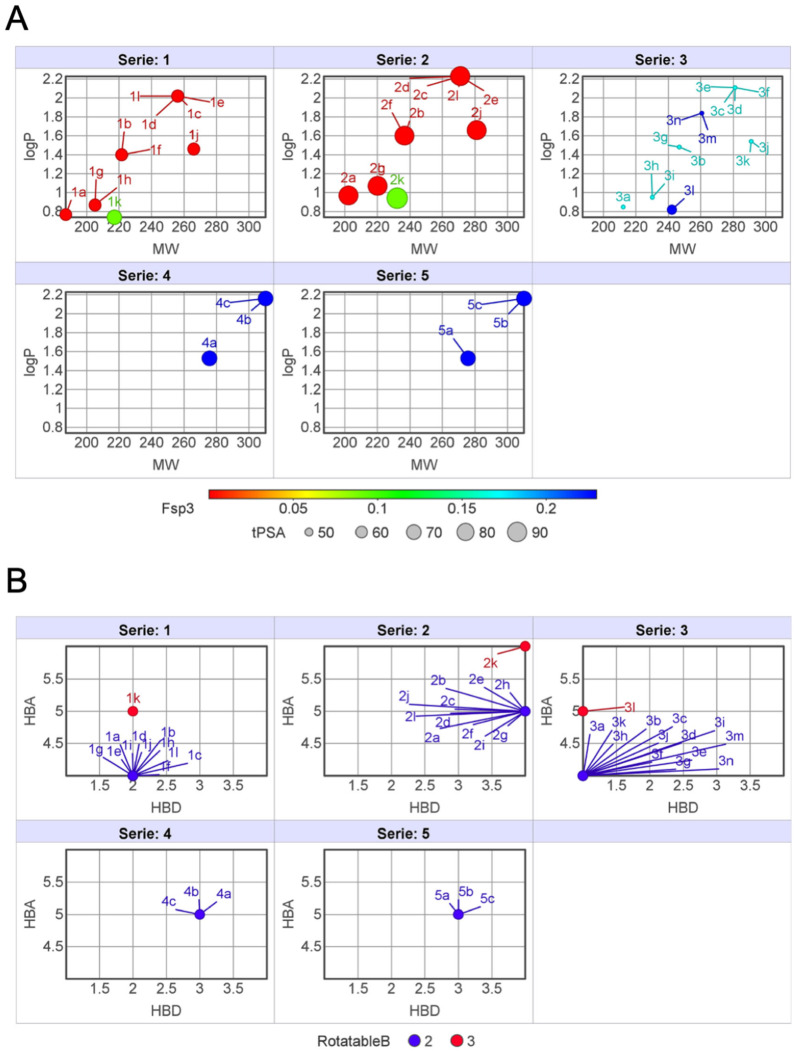
Physicochemical properties of pyrazole derivatives, **1**(**a**–**l**), **2**(**a**–**l**), **3**(**a**–**n**), **4**(**a**–**c**) and **5**(**a**–**c**), were calculated using DataWarrior software. (**A**) Classification of compounds according to the lipophilicity (LogP), molecular weight (MW), the fraction of sp^3^ carbon atoms (Fsp^3^; color scale) and the topological polar surface area (tPSA; label size). (**B**) Distribution graph of compounds based on number of hydrogen bond donors (HBD) and acceptors (HBA), and rotatable bonds (Rotatable B; colored circles).

Drug oral bioavailability, an important issue for the success of a potential therapeutic agent, is highly influenced by physicochemical properties [31]. Thus, the prediction of solubility and permeability among other physicochemical parameters contribute to the drug development process. First, we assessed the compliance of the series of pyrazole derivatives, **1**(**a**–**l**), **2**(**a**–**l**), **3**(**a**–**n**), **4**(**a**–**c**), and **5**(**a**–**c**), with Lipinski’s criterion (Figure 2). The in silico analysis revealed that all pyrazole derivatives obeyed Lipinski’s rule of five (Ro5), including molecular weight (MW < 500), lipophilicity (LogP < 5), number of hydrogen bond donors (HBD < 5), acceptors (HBA < 10), and topological polar surface area (tPSA < 140 Å2), without any violation. A small variation in molecular weight was observed among the 44 pyrazole derivatives, with MW intervals for series 1 of 187.2 ≤ MW ≤ 266.09, series 2 of 202.21 ≤ MW ≤ 281.11, series 3 of 212.25 ≤ MW ≤ 291.15, series 4 and 5 of 275.74 ≤ MW ≤ 310.18 (Figure 2). Lipophilicity, referred to as LogP, has a relevant impact on solubility and affects drug permeability and absorption, influencing pharmacokinetic properties [32]. 

Most compounds (76.2%) have an optimum solubility characteristic with LogP values between 1 and 2.23, the number of hydrogen bond (1 ≤ HBD ≤ 4; 5 ≤ HBA ≤ 10) and topological polar surface area (tPSA ≤ 96 Å^2^) fitting the Ro5 (Figure 2), suggesting their potential for oral absorption with a low probability of adverse in vivo outcomes. The oral bioavailability of the pyrazole derivatives was also predicted with the SwissADME server [33], which analyzes, in an integrated way, six physicochemical properties: lipophilicity, size, polarity, solubility, saturation, and flexibility. All pyrazole derivatives fit the criteria for good oral bioavailability, with **3m**, **3n**, **4**(**a**–**c**), and **5**(**a**–**c**) matching all parameters in the radar plot area (Supplementary Figures S1–S4).

Drug properties are a challenge faced in the development of new molecular entities and efforts have been made to reach a good balance between physicochemical properties and pharmacokinetic/pharmacodynamic efficacy. Molecules with high lipophilicity (LogP > 3) increase drug promiscuity and toxicity since they tend to bind to hydrophobic targets instead of the main target [34]. On the other hand, low lipophilicity decreases permeability and absorption. Thus, the identification of an ideal candidate requires the balance and integration of multiple physicochemical properties of the molecule sought in the drug rational design.

### 2.3. Toxicity and Trypanocidal Effect of Pyrazole Derivatives

The cytotoxicity of the pyrazole derivatives was evaluated on mammalian cells, using Vero cells as a model. After the incubation (72 h) of cell monolayers with pyrazole derivatives in the range of 15.62–500 µM, cell viability was analyzed by ATP quantification. The results revealed the low toxicity of the pyrazole derivatives. A CC_50_ value > 500 µM, similar to Bz, was evidenced in 23% of the compounds analyzed, while other analogs showed CC_50_ values between 160.51 and 479.66 µM (Table 1).

The phenotypic screening showed that the pyrazole derivatives, series **1**(**a**–**l**), **2**(**a**–**l**), **3**(**a**–**n**), **4**(**a**–**c**), and **5**(**a**–**c**), have low activity against trypomastigote forms of *T. cruzi* compared to Bz (IC_50_ = 18.71 ± 4.58 µM), the reference drug, with only 11.4% of the compounds with IC_50_ value < 100 µM (Table 1). Among the compounds analyzed, **3m** (IC_50_ = 34.54 ± 8.32 µM) was the most active against trypomastigotes (Table 1). These data diverge from the activity of the hit compound [13], which showed activity 2-fold greater than Bz against trypomastigotes (IC_50_ = 9.5 ± 1.2 µM), reinforcing that a small structural change drastically influence the biological activity of the derivatives.

Drug activities were also evaluated against the intracellular parasites. Low activity of the pyrazole-carboxamide hybrids, series **1**(**a**–**l**) and **2**(**a**–**l**), was observed against intracellular amastigotes, with IC_50_ values higher than 50 µM (Table 1). The replacement of secondary amide in the 3H5 compound by primary amide may be responsible for the loss of trypanocidal activity, probably hindering its proper insertion into the active site of cruzipain. In the 3H5 compound, the carboxamide has a central position in its chemical structure, between the pyrazole and benzimidazole rings. The nitrogen and oxygen atoms of the carboxamide of this compound interact by hydrogen bonds with residues aspartate 161 (ASP161), serine 25 (SER25), and glycine 23 (GLY23) located in the active site cleft of cruzipain [24]. Therefore, in derivatives of series 1 and 2, the outermost positioning of the carboxamide may interfere with the fit of pyrazole-carboxamide derivatives in the enzyme cleft and influence the ligand-target binding affinity and biological activity.

Series **3**(**a**–**n**), structurally similar to the hit compound (pyrazole-imidazoline hybrid) without the amino group (NH_2_), showed three analogs (**3g**, **3j**, and **3m**) with high activity against intracellular amastigotes (Table 1). Compounds **3j** (IC_50_ = 2.75 ± 0.62 µM) and **3m** (IC_50_ = 3.58 ± 0.25 µM) were more active than Bz (IC_50_ = 4.67 ± 0.22 µM). The analysis of the efficacy of the series 3 derivatives revealed that 50% of the analogs had IC_90_ values lower than Bz (IC_90_ > 100 µM), with emphasis on **3j** (IC_90_ = 9.67 ± 0.30 µM) and **3m** (IC_90_ = 21.37 ± 1.25 µM). It is also noteworthy the high selectivity **3g**, **3j**, and **3m**, reaching a selectivity index (SI) of 45.52, 112.48 and 44.83, respectively (Table 1). The insertion of the methyl group in the pyrazole **4**(**a**–**c**) or imidazoline **5**(**a**–**c**) ring resulted in the loss of trypanocidal effect, with IC_50_ values varying between 32.95 and 93.83 µM (Table 1) and, therefore, less active than series 3 and Bz. Among the active derivatives, **3g**, **3j**, and **3m** were identified as the most promising against *T. cruzi*. 

Selective activity for intracellular amastigotes was also evidenced in 3-methyl-1-phenyl-1*H*-pyrazolo[3,4-*b*]pyridine-4-carbohydrazide derivatives. These analogs showed little or no activity against trypomastigotes, with the loss of trypanocidal activity attributed to the insertion of fluorine in C6, a strong electron-withdrawing group (EWG) [35]. Moreover, a similar phenomenon has been reported with posaconazole, an inhibitor of ergosterol biosynthesis, which has excellent activity against intracellular amastigotes, in the nanomolar range, but it is less effective against the non-replicative stage and low-replication cycle amastigotes (dormant amastigotes), suggesting that replication and the speed of its cycles may affect the efficacy of posaconazole [36].

### 2.4. Structure-Activity Relationship (SAR) of Pyrazole Derivatives

Series **1**(**a**–**l**), **2**(**a**–**l**), **3**(**a**–**n**), **4**(**a**–**c**) and **5**(**a**–**c**) were synthesized to optimize the hit compound by replacing the imidazoline ring with carboxamide (series 1 and 2), removing the amino group (series 3) and addition of the methyl group in the pyrazole (series 4) or imidazoline (series 5) ring. The distinct activity profile of pyrazole derivatives (series 1–5) against intracellular amastigotes highlights the importance of the molecule’s chemical structure in the potency (pIC50) of bioactive compounds (Table 2).

The SAR analysis showed that the replacement of the imidazoline ring of the hit compound by carboxamide (series 1 and 2) impacted the trypanocidal activity and resulted in a drop in biological potency (pIC50 < 4.3) (Table 2). The removal of the amino group and addition of substituents, such as halogens (Br, Cl and F), methyl (CH_3_) and methoxy (OCH_3_) groups, in the benzene ring (series 3) caused a differential increase in the biological activity of the pyrazole-imidazoline **3**(**b**–**n**) compared to the unsubstituted derivative **3a** (pIC50 = 4.03). Disubstituted analogues **3**(**c**–**f)** with dichloro substituents in C2 (2,4-Cl_2_ and 2,6-Cl_2_) and C3 (3,4-Cl_2_ and 3,5-Cl_2_) showed low potency (4.09 ≤ pIC50 ≤ 4.26). However, the replacement of the Cl atom by methyl in the *para* position of the 3,4-dichloro **3e**, leading to the 3-chloro-4-methyl **3m** (pIC50 = 5.45), resulted in trypanocidal activity 21-fold higher than **3e** (pIC50 = 4.12). The improved activity was also evidenced when the Cl atom was replaced by the methyl group in the *ortho* position of the 2,4-dichloro **3c** (pIC50 = 4.25), giving rise to the 2-methyl-4-chloro **3n** (pIC50 = 4.61). The Cl atom in the *ortho* position appears to negatively modulate trypanocidal activity. Compound **3f** (2,6-dichloro), for example, showed an even greater drop in potency (pIC50 = 4.09), consistent with the observation that C2 is a suboptimal place for substitution. Loss of biological activity was evidenced in fluorinated derivatives in C4 (**3h**) and C3 (**3i**), both compounds with a pIC50 value of 4.19. The addition of the methoxy group in C4 (**3l**) also resulted in the loss of activity (pIC50 = 4.14). On the other hand, the introduction of Cl and Br in the *para* position, **3g** (pIC50 = 5.22) and **3j** (pIC50 = 5.56), respectively, increased trypanocidal activity (Table 2). In contrast, attempts to move the Cl (**3b**) and Br (**3k**) to the *meta* position led to a five- and 10-fold drop-in activity, respectively, compared to **3g** and **3j** analogs, respectively (Table 1). Thus, only the structural changes of **3g**, **3j**, and **3m**, with the introduction of the substituents Br, Cl and methyl in the *para* position induced a large increase in activity, with potency values similar to Bz (pIC50 = 5.33) (Table 2). These data differ from the *meta* position previously identified in hit compound (3,4- and 3,5-diCl) as a potential activity regulator [13]. Although **3m** (3-Cl,4-CH_3_) has similarities with the hit compound, the substitution of the Cl atom by methyl in the *para* position, associated with the subtraction of the amino group in the series 3 of the pyrazole derivatives, increased by 3.8-fold the activity against intracellular amastigotes, probably giving the new analog greater permeability by making the molecule slightly more lipophilic, facilitating the interaction with the molecular target. Pyrazole *N*-ethylurea derivatives showed potent activity against *T. brucei brucei* and *T. cruzi*, with a drastic reduction of parasitemia after 6 days of treatment and efficacy in the murine model of acute *T. cruzi* infection. The SAR study for *N*-ethylurea pyrazole derivatives revealed that the *meta* position in the aromatic ring substantially influences the trypanocidal activity, with the compounds with fluorine and chlorine substituents being the most effective [37].

Six analogs were also prepared by inserting the methyl group in the pyrazole **4**(**a**–**c**) or imidazoline **5**(**a**–**c**) ring containing the 3-Cl, 3,5- and 3,4-dichloro substituents based on the good activity of the chlorinated derivatives, mainly dichlorinated, of the hit compound. The introduction of the methyl group in the 5-amino-pyrazole-imidazoline compounds did not result in a beneficial effect for the trypanocidal activity. The compounds with the addition of methyl in the pyrazole ring showed an even greater decrease in potency with pIC50 values of approximately 4.

### 2.5. Effect of Pyrazole Derivatives on Parasite Recrudescence

**Figure 3:**
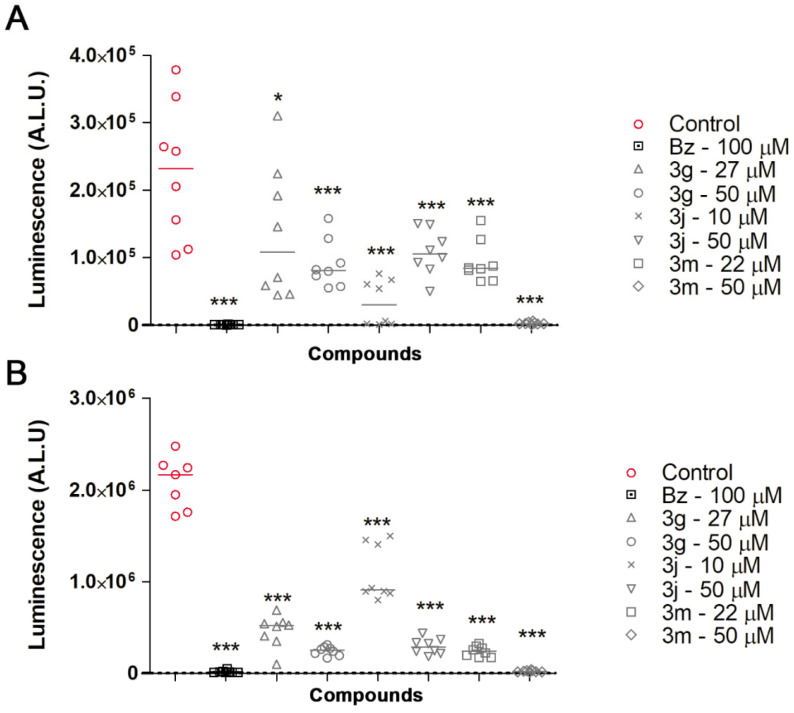
*T. cruzi* washout assay with promising pyrazole derivatives and Bz. Luminescence signal, represented as arbitrary luminescence unit (ALU), of Vero cell cultures infected with *T. cruzi* treated with **3g**, **3j**, **3m** or Bz, using IC_90_ or 50 µM concentrations, after washing and 72 h incubation in the absence of the compounds. (**A**) Analysis of viable parasites (trypomastigotes) released in the culture supernatants. (**B**) Infection profile of Vero cell monolayers. (*) Statistically significant in relation to the untreated group using the One-Way ANOVA test, being *p* < 0.05 (*) and *p* < 0.001 (***).

To address the question of whether the treatment with **3g**, **3j**, and **3m** leads to parasite clearance, an intracellular *T. cruzi* washout assay was performed as a strategy for monitoring the parasite recrudescence. In this approach, cultures of infected Vero cells (Dm28c-Luc) were treated for 72 h at IC_90_ and 50 µM (2 to 5-fold de IC_90_) concentrations of the promising compounds **3g**, **3j**, and **3m** and, after washing the cell monolayers, the cultures were kept for another 72 h without drug treatment. Both cell monolayers and their supernatants were evaluated for the presence of viable parasites after luciferin addition. All derivatives analyzed relapsed at the IC_90_ concentration (Figure 3). However, treatment with **3g**, **3j**, and **3m** led to a significant reduction in the parasite load, reaching a decrease of 4.5-, 1.9-, and 8.7-fold, respectively, when compared to untreated infected Vero cells cultures (Figure 3). The emergence of trypomastigotes in the supernatant, at IC_90_ concentration, was observed three days after removal of drug pressure, but with a significant reduction in viable parasites compared to untreated cultures (Figure 3). Parasite recrudescence was not seen in 100 µM Bz treatment (Figure 3).

Treatment with 50 µM of **3g**, **3j**, and **3m** (2–5 times IC_90_) revealed the efficacy of **3m**, which showed the same activity profile as 100 µM Bz (Figure 3). The effectiveness of **3m** (50 µM) in eliminating intracellular amastigotes, such as Bz (100 µM), with inhibition of trypomastigotes released in the culture supernatant is highlighted (Figure 3), being identified as promising to advance in preclinical in vivo assays. Also, the prolonged drug pressure or even adequacy of treatment concentrations, reflecting the therapeutic index of promising derivatives, may improve their biological activity, especially of **3j**, which showed better potency (pIC50 = 5.56) and higher selective index (SI = 112.48). Furthermore, the combination of the promising candidates of pyrazole derivatives (**3g**, **3j**, and **3m**) with Bz could lead to a synergistic effect and potentiate the trypanocidal activity. These analyzes will be subject to further investigation.

Pyrrolo-[2,3-*b*]pyridine nucleoside analogs, which have high in vitro (submicromolar) trypanocidal activity, were able to suppress parasitemia and induce 100% survival in a murine model of acute *T. cruzi* infection, but there was a reactivation of parasitemia after immunosuppression, as predicted in the in vitro washout assay [38]. Evidence has also shown that posaconazole and five new 3-pyridyl derivatives, identified as putative CYP51 inhibitors, are not able to maintain sustainable trypanocidal activity in vitro, with reactivation of infection after two days without compounds pressure [39]. These washout data supported the results of the clinical trial with posaconazole and ravuconazole that suppressed parasitemia only during the treatment period (60 days) [10,11]. Thus, the incorporation of a washout assay in the drug screening platform is an important strategy to improve the in vivo translation, allowing the ineffectiveness of candidate compounds to be identified in advance and preventing their progression through the pipeline of new drug discovery for Chagas disease.

### 2.6. 3D Culture Model as Potential Drug Efficacy Prediction

**Figure 4:**
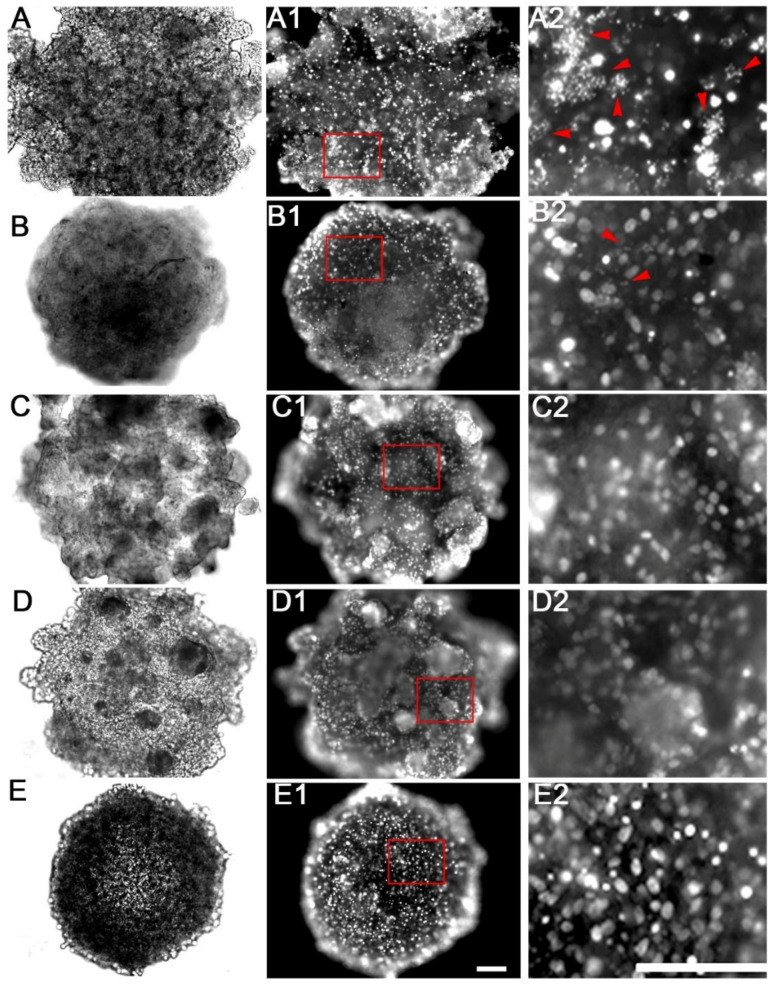
Effect of **3g**, **3j** and **3m** on *T. cruzi*-infected microtissue. Brightfield microscopy images of spheroids from untreated *T. cruzi* (Dm28c-Luc) infected Vero cells (**A**), treated with Bz (**B**) or pyrazole-imidazoline derivatives (**C**–**E**). DAPI stained fluorescence images of untreated infected spheroid (**A**–**A2**) and after 72 h-treatment with Bz (**B**–**B2**), **3g** (**C**–**C2**), **3j** (**D**–**D2**) and **3m** (**E**–**E2**). High magnification of the spheroid region marked in red (insert) (**A**–**E2**). Note the presence of intracellular amastigote on microtissue (arrowheads).

The application of 3D cell culture in the drug discovery has been of great interest since 3D culture mimics the physiological microenvironments in vivo, with cell–cell and cell–extracellular matrix interaction similar to tissue architecture influencing the cell phenotype and drug response, making it a more relevant and reliable system [40]. The cultivation of Vero cells in agarose-coated plates, inhibiting cell adhesion to the substrate, favored the three-dimensional intercellular interaction and the spheroid formation. Thus, *T. cruzi*-infected spheroids were used to evaluate the effectiveness of **3g**, **3j**, and **3m** in permeating and eliminating the parasites in the microtissue. Intracellular amastigote nests, visualized by DAPI staining (DNA dye), were mostly distributed in the outer layers of the untreated spheroids, but parasites were also seen deep inside the microtissue (Figure 4). A drastic reduction in microtissue infection was observed with all derivatives, as observed with Bz (Figure 4). Rare parasites were observed in microtissues treated with Bz as well as in the treatment with **3g**, **3j**, and **3m**. Trypomastigotes transmigration through tissue seems to be related to *T. cruzi* virulence, with only virulent strains having the ability to transmigrate and infect deep cell layers in the microtissue [41].

**Figure 5:**
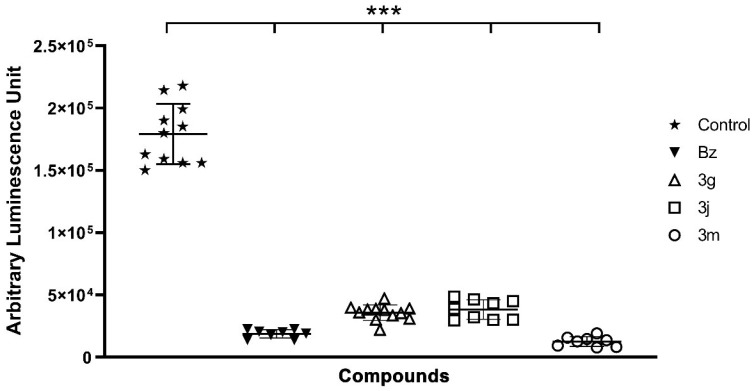
Biological activity of **3g**, **3j** and **3m** in *T. cruzi*-infected microtissue. Determination of intracellular parasite viability by luciferase enzyme activity (Arbitrary luminescence unit; ALU). Treatment with pyrazole derivatives, with emphasis on **3m**, and Bz induced a reduction of parasite load in the microtissue. (***) Statistically significant in relation to the untreated group using the One-Way ANOVA test, with *p* < 0.001 (***).

The viability of intracellular parasites in the microtissue was measured, by the addition of luciferin, after **3g**, **3j**, **3m**, and Bz treatment (72 h). The luminescence intensity data, represented as arbitrary luminescence unit (ALU), revealed a high parasite load in untreated infected microtissues (ALU = 175,040 ± 24,826) (Figure 5). A significant reduction in the viability of intracellular parasites was demonstrated with **3g** (ALU = 36,130 ± 5764), **3j** (ALU = 39,001 ± 9952), and **3m** (ALU = 12,683 ± 3976) compared to the untreated infected microtissues (Figure 5). Among the compounds analyzed, **3m** stands out for reducing microtissue infection by 93%, with a trypanocidal activity profile like Bz (ALU = 18,858 ± 3726) (Figure 5).

Posaconazole treatment of *T. cruzi*-infected cardiac microtissue, a 3D model that reproduces the cardiac fibrosis seen in Chagas′ cardiomyopathy [42], reduced the parasite load by 50% at 5 nM, as well as the fibronectin and laminin expression, significantly reducing cardiac fibrosis induced by *T. cruzi* infection [43]. The translational potential of the 3D platforms has also been demonstrated for the liver stage of *Plasmodium* infection. Treatment of *P. berghei*-infected HepG2 spheroids with DDD107498 (M5717) and atovaquone demonstrated that the 3D model reproduces the in vivo response, highlighting this important tool in identifying effective new drugs for malaria [44]. Microtissues have also been widely employed in cancer therapy, showing the potential to increase the predictive value of preclinical drug research. The comparison between 2D and 3D models of colorectal cancer cell lines and their response to irradiation and chemotherapy with doxorubicin, 5-FU, mitomycin C, and cisplatin showed greater resistance of the 3D model to treatments, reflecting more accurately the tumors in patients [45]. Further, three-dimensional cultures models improve the preclinical prediction of safety and efficacy of the candidate compound and are more reliable in vitro assays of human susceptibility to drug response.

### 2.7. ADMET Analysis

The pharmacokinetics of xenobiotics is dependent on the ADMET phenomenon (absorption, distribution, metabolism, excretion and transport). Thus, considering **3g**, **3j**, and **3m**, which showed good activity against intracellular amastigotes (IC_50_ < 10 µM) and similar efficacy to Bz in microtissue, as promising candidates to proceed for in vivo pharmacokinetic and in vivo preclinical assays, their ADMET parameters were analyzed in silico (Table 3). Derivatives **3g**, **3j**, and **3m** showed a similar ADMET profile with few peculiarities. As for absorption, all derivatives had a favorable absorption profile, demonstrating good absorption in the intestinal epithelium, Caco-2 cells and blood-brain barrier, with the prediction of good oral bioavailability (Table 3), compatible with the data on physicochemical properties. Furthermore, **3g** and **3m** are predictive of mitochondrial localization while the **3j** can be accumulated in lysosomes.

Overall, the pyrazole derivatives are not promiscuous inhibitors of cytochrome P450 (CYP450) enzymes, with only CYP1A2 inhibition, suggesting that the promising derivatives undergo phase I biotransformation in the liver. The **3m** derivative was also predicted as a substrate for P-glycoprotein (P-gp), a xenobiotic efflux pump located in the plasma membrane, and CYP3A4 (Table 3). As a substrate for P-gp and CYP3A4, **3m** may induce the activity of this constituent of the ABC transporter family (P-gp) and the CYP3A4 isoenzyme.

The toxicity profile of the promising compounds is also quite favorable. Derivatives **3g**, **3j**, and **3m** are not carcinogenic, do not induce mutagenicity of AMES, but have a prediction of hepatotoxicity and inhibition of hERG (voltage-dependent potassium channels type hERG), which can cause disturbances in the electrical conduction of the heart (Table 3). Thus, a more detailed analysis of the pharmacokinetics of promising pyrazole derivatives would be essential for the determination of doses and treatment regimens within the therapeutic index, avoiding adverse effects in preclinical in vivo assays.

### 2.8. Enzyme Activity

**Figure 6:**
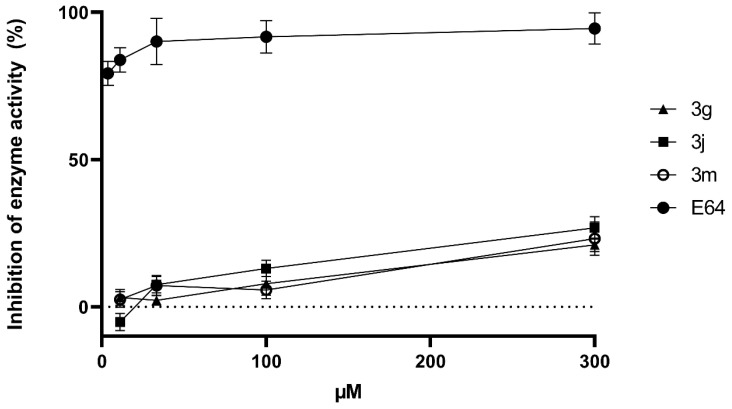
Inhibition of *T. cruzi* cysteine protease activity. Total protein extracts of trypomastigotes (5 µg), was incubated at different concentrations (300–11.11 µM) of compounds E64 (black circle), **3g** (black triangle), **3j** (black square) and **3m** (white circle) in enzyme activation buffer pH 5.0, at 37 °C. Enzyme activity was measured using a specific fluorogenic peptide substrate Z-FR-AMC (60 µM). Results are presented as percentage of inhibition of enzyme activity (%) and represent the mean and standard deviation (±) of three independent experiments. Hence, 100% of activity is relative to 0.22 µmol min^−1^ µg of protein^−1^.

In an attempt to assess whether **3g**, **3j**, and **3m** had properties of inhibiting cysteine protease, as reported for the hit compound [13], the effect of the derivatives was evaluated on an enzyme activity kinetic (0–1 h at 37 °C) in the presence of the fluorogenic substrate Z-FR-AMC. Incubation of trypomastigote total protein extracts with promising pyrazole derivatives did not change the enzyme activity profile. The results revealed an inexpressive inhibitory activity of the three derivatives analyzed (Figure 6).

In general, the maximum inhibition of enzymatic activity, approximately 26%, was achieved with 300 µM for the analyzed pyrazole-imidazoline derivatives (Figure 6). The specificity of the activity of cysteine proteases was evaluated by measuring the hydrolysis of the substrate Z-FR-AMC in the presence of E-64, an irreversible and selective inhibitor of cysteine protease (Figure 6), which revealed effective inhibition of the enzymatic activity, reaching inhibition rates greater than 80% at compound concentrations ≥ 11.11 µM (Figure 6).

The enzymatic activity data demonstrated that **3g**, **3j**, and **3m** are weak inhibitors of cysteine protease and, therefore, the improvement of trypanocidal activity against intracellular parasites involves other mechanisms of action not yet elucidated. Evidence has shown that, in addition to cruzipain, the iron-dependent superoxide dismutase (Fe-SOD) [46,47] and CYP51 [48] are molecular targets implicated in the trypanocidal activity of the pyrazole derivatives. Thus, it is possible that the trypanocidal activity of promising pyrazole derivatives is associated with mitochondrial dysfunction, as predicted by the pyrazole derivatives distribution in the ADMET analysis, which will be a target of further analysis.

## 3. Materials and Methods

### 3.1. Compound Synthesis

All commercial raw materials and solvents were used as received. The progress of reactions was monitored by thin-layer chromatography (TLC)on aluminum plates precoated with F254 silica gel (Macherey-Nagel, Düren, Germany). The melting points were determined on an Allerbest or a Fisatom 430 apparatus (Fisatom, São Paulo, SP, Brazil). Fourier transform infrared (FT-IR) spectra were obtained on a Spectrum 100 instrument (PerkilElmer, Waltham, MA, USA), equipped with ATR diamond-ZnSe system. Nuclear Magnetic Resonance (NMR) spectra were recorded on a Bruker Avance (500 or 400 MHz), at 25 °C, in CDCl_3_ or deuterated dimethyl sulfoxide (DMSO-*d*_6_) as solvent. The High-Resolution Mass Spectrometry (HRMS) was performed using Micromass ZQ-4000 spectrometer with Electrospray Ionization (Waters, Milford, MA, USA). The ^1^H and ^13^C NMR spectra of all compounds, **1**(**a**–**l**), **2**(**a**–**l**), **3**(**a**–**n**), **4**(**a**–**c**), and **5**(**a**–**c**), are available on Supplementary Data (Figure S5).

*1-aryl-1H-pyrazole-carboxamides 1(a-l) and 5-amino-1-aryl-1H-pyrazole-carboxamides 2*(**a**–**l**)

The compounds **1**(**a**–**l**) and **2**(**a**–**l**) were synthesized according to the methodology previously described by our research group [26]. The analytical results are shown below.

*1-phenyl-1H-pyrazole-4-carboxamide* (**1a**)

Yield: 72%; m.p.: 224–225 °C; FT-IR ν (cm^−1^): 3399–3110, 1647, 1617–1419; ^1^H NMR (500 MHz, CDCl_3_) δ 8.43 (s, 1H), 7.97 (s, 1H), 7.70 (d, *J* = 7.7 Hz, 2H), 7.49 (t, *J* = 7.7 Hz, 2H), 7.37 (t, *J* = 7.7 Hz, 1H), 5.83 (br, 2H); ^13^C NMR (125 MHz, CDCl_3_) δ 164.6, 139.8, 139.2, 129.6, 129.3, 127.7, 119.6, 118.4; HRMS (ESI) *m/z* [M + Na]^+^ = 210.0634 (found), [M + Na]^+^ = 210.0643 (calculated).

*1-(3-chlorophenyl)-1H-pyrazole-4-carboxamide* (**1b**)

Yield: 91%; m.p.: 202–204 °C; FT-IR ν (cm^−1^): 3392–3118, 1646, 1614–1433; ^1^H NMR (500 MHz, CDCl_3_) δ 8.41 (s, 1H), 7.96 (s, 1H), 7.78 (s, 1H), 7.58 (d, *J* = 7.8 Hz, 1H), 7.42 (t, *J* = 7.8 Hz, 1H), 7.34 (d, *J* = 7.8 Hz, 1H), 5.72 (br, 2H); ^13^C NMR (125 MHz, CDCl_3_) δ 163.9, 140.4, 140.2, 135.7, 130.9, 129.3, 127.8, 120.2, 117.5, 116.4; HRMS (ESI) *m/z* [M + Na]^+^ = 244.0246 (found), [M + Na]^+^ = 244.0254 (calculated).

*1-(2,4-dichlorophenyl)-1H-pyrazole-4-carboxamide* (**1c**)

Yield: 83%; m.p.: 184–186 °C; FT-IR ν (cm^−1^): 3351–3194, 1663, 1609–1424; 1H NMR (400 MHz, DMSO-*d*_6_) δ 8.55 (s, 1H), 8.13 (s, 1H), 7.92 (d, *J* = 2.2 Hz, 1H), 7.74 (br, 1H), 7.67 (d, *J* = 8.6 Hz, 1H), 7.62 (dd, *J* = 8.6, 2.2 Hz, 1H), 7.21 (br, 1H);13C NMR (100 MHz, DMSO-*d*_6_) δ 163.4, 141.2, 136.8, 134.4, 133.9, 130.5, 129.8, 129.5, 128.9, 120.5; HRMS (ESI) *m*/*z* [M + Na]^+^ = 277.9854 (found), [M + Na]^+^ = 277.9864 (calculated).

*1-(3,5-dichlorophenyl)-1H-pyrazole-4-carboxamide* (**1d**)

Yield: 88%; m.p.: 240–242 °C; FT-IR ν (cm^−1^): 3341–3093, 1655, 1614–1423; ^1^H NMR (400 MHz, DMSO-*d*_6_) δ 9.05 (s, 1H), 8.18 (s, 1H), 7.97 (d, *J* = 1.8 Hz, 2H), 7.70 (br, 1H), 7.61 (t, *J* = 1.8 Hz, 1H), 7.27 (br, 1H); ^13^C NMR (100 MHz, DMSO-*d*_6_) δ 163.1, 141.7, 141.3, 135.5, 130.8, 126.6, 121.8, 117.8; HRMS (ESI) *m/z* [M + Na]^+^ = 277.9852 (found), [M + Na]^+^ = 277.9864 (calculated).

*1-(3,4-dichlorophenyl)-1H-pyrazole-4-carboxamide* (**1e**)

Yield: 84%; m.p.: 190–192 °C; FT-IR ν (cm^−1^): 3403–3108, 1648, 1613–1429; ^1^H NMR (500 MHz, CDCl_3_) δ 8.39 (s, 1H), 7.96 (s, 1H), 7.89 (s, 1H), 7.56 (s, 2H), 5.73 (br, 2H); ^13^C NMR (125 MHz, CDCl_3_) δ 163.7, 140.3, 138.6, 134.1, 131.7, 131.5, 129.2, 121.6, 120.1, 118.5; HRMS (ESI) *m/z* [M + H]^+^ = 256.0042 (found), [M + H]^+^ = 256.0044 (calculated).

*1-(4-chlorophenyl)-1H-pyrazole-4-carboxamide* (**1f**)

Yield: 86%; m.p.: 234–236 °C; FT-IR ν (cm^−1^): 3401–3109, 1646, 1619–1408; ^1^H NMR (400 MHz, DMSO-*d*_6_) δ 8.92 (s, 1H), 8.14 (s, 1H), 7.88 (d, *J* = 9.0 Hz, 2H), 7.70 (br, 1H), 7.59 (d, *J* = 9.0 Hz, 2H), 7.22 (br, 1H); ^13^C NMR (100 MHz, DMSO-*d*_6_) δ 163.4, 141.3, 138.4, 131.5, 130.0, 129.9, 121.3, 120.8; HRMS (ESI) *m/z* [M + Na]^+^ = 244.0240 (found), [M + Na]^+^ = 244.0254 (calculated).

*1-(4-fluorophenyl)-1H-pyrazole-4-carboxamide* (**1g**)

Yield: 85%; m.p.: 244–246 °C; FT-IR ν (cm^−1^): 3400–3107, 1645, 1617–1412; ^1^H NMR (400 MHz, DMSO-*d*_6_) δ 8.87 (s, 1H), 8.12 (s, 1H), 7.88 (dd, *J* = 8.8, 4.7 Hz, 2H), 7.69 (br, 1H), 7.38 (t, *J* = 8.8 Hz, 2H), 7.21 (br, 1H); ^13^C NMR (100 MHz, DMSO-*d*_6_) δ 163.5, 161.1 (d, *J* = 243.6 Hz), 141.0, 136.2 (d, *J* = 2.7 Hz), 129.8, 121.3 (d, *J* = 8.6 Hz), 121.1, 116.8 (d, *J* = 23.1 Hz); HRMS (ESI) *m/z* [M + Na]^+^ = 228.0536 (found), [M + Na]^+^ = 228.0549 (calculated).

*1-(3-fluorophenyl)-1H-pyrazole-4-carboxamide* (**1h**)

Yield: 93%; m.p.: 206–208 °C; FT-IR ν (cm^−1^): 3406–3113, 1648, 1602–1410; ^1^H NMR (400 MHz, DMSO-*d*_6_) δ 8.97 (s, 1H), 8.16 (s, 1H), 7.76–7.71 (m, 3H), 7.60–7.54 (m, 1H), 7.23–7.19 (m, 2H); ^13^C NMR (100 MHz, DMSO-*d*_6_) δ 163.4, 163.0 (d, *J* = 242.5 Hz), 141.3, 141.0 (d, *J* = 10.6 Hz), 132.0 (d, *J* = 9.3 Hz), 130.1, 121.4, 115.0 (d, *J* = 2.9 Hz), 114.0 (d, *J* = 21.1 Hz), 106.5 (d, *J* = 26.7 Hz); HRMS (ESI) *m/z* [M + H]^+^ = 206.0718 (found), [M + H]^+^ = 206.0730 (calculated).

*1-(4-bromophenyl)-1H-pyrazole-4-carboxamide* (**1i**)

Yield: 92%; m.p.: 242–244 °C; FT-IR ν (cm^−1^): 3392–3109, 1644, 1618–1405; ^1^H NMR (400 MHz, DMSO-*d*_6_) δ 8.93 (s, 1H), 8.14 (s, 1H), 7.82 (d, *J* = 9.0 Hz, 2H), 7.72 (d, *J* = 9.0 Hz, 2H), 7.71 (br, 1H), 7.22 (br, 1H); ^13^C NMR (100 MHz, DMSO-*d*_6_) δ 163.4, 141.3, 138.8, 132.9, 129.8, 121.4, 121.1, 119.8; HRMS (ESI) *m/z* [M + Na]^+^ = 287.9729 (found), [M + Na]^+^ = 287.9748 (calculated).

*1-(3-bromophenyl)-1H-pyrazole-4-carboxamide* (**1j**)

Yield: 81%; m.p.: 212–214 °C; FT-IR ν (cm^−1^): 3450–3105, 1654, 1611–1428; ^1^H NMR (400 MHz, DMSO-*d*_6_) δ 8.98 (s, 1H), 8.15 (s, 1H), 8.08 (t, *J* = 2.0 Hz, 1H), 7.88 (ddd, *J* = 8.1, 2.0, 1.0 Hz, 1H), 7.69 (br, 1H), 7.56 (ddd, *J* = 8.1, 2.0, 1.0 Hz, 1H), 7.49 (t, *J* = 8.1 Hz, 1H), 7.23 (br, 1H); ^13^C NMR (100 MHz, DMSO-*d*_6_) δ 163.3, 141.4, 140.8, 132.1, 130.1, 130.0, 122.8, 121.7, 121.4, 118.1; HRMS (ESI) *m/z* [M + Na]^+^ = 287.9725 (found), [M + Na]^+^ = 287.9748 (calculated).

*1-(4-methoxyphenyl)-1H-pyrazole-4-carboxamide* (**1k**)

Yield: 88%; m.p.: 220–222 °C; FT-IR ν (cm^−1^): 3378–3109, 1645, 1613–1412; ^1^H NMR (500 MHz, CDCl_3_) δ 8.32 (s, 1H), 7.93 (s, 1H), 7.60 (d, *J* = 8.9 Hz, 2H), 6.99 (d, *J* = 8.9 Hz, 2H), 5.73 (br, 2H), 3.86 (s, 3H); ^13^C NMR (125 MHz, CDCl_3_) δ 159.2, 139.4, 129.3, 129.2, 121.8, 121.4, 115.0, 114.9, 55.8; HRMS (ESI) *m/z* [M + Na]^+^ = 240.0724 (found), [M + Na]^+^ = 240.0749 (calculated).

*1-(2,3-dichlorophenyl)-1H-pyrazole-4-carboxamide* (**1l**)

Yield: 69%; m.p.: 220–224 °C; FT-IR ν (cm^−1^): 3459–3337, 1661, 1623–1425; ^1^H NMR (400 MHz, DMSO-*d*_6_) δ 8.56 (s, 1H), 8.14 (s, 1H), 7.83 (d, *J* = 7.2 Hz, 1H), 7.74 (br, 1H), 7.63 (d, *J* = 7.2 Hz, 1H), 7.55 (t, *J* = 8.0 Hz, 1H), 7.22 (br, 1H); ^13^C NMR (100 MHz, DMSO-*d*_6_) δ 163.4, 141.2, 139.5, 134.0, 133.3, 131.4, 129.3, 127.7, 127.6, 120.5; HRMS (ESI) *m/z* [M + Na]^+^ = 277.9855 (found), [M + Na]^+^ = 277.9864 (calculated).

*5-amino-1-phenyl-1H-pyrazole-4-carboxamide* (**2a**)

Yield: 27%; m.p.: 148–150 °C; FT-IR ν (cm^−1^): 3370–3168, 1653, 1596–1440; ^1^H NMR (500 MHz, DMSO-*d*_6_) δ 7.90 (s, 1H), 7.57–7.50 (m, 4H), 7.38 (t, *J* = 7.2 Hz, 1H), 7.37 (br, 1H), 6.84 (br, 1H), 6.36 (br, 2H); ^13^C NMR (125 MHz, DMSO-*d*_6_) δ 166.0, 149.1, 138.8, 138.1, 129.2, 126.8, 122.9, 97.4; HRMS (ESI) *m/z* [M + Na]^+^ = 225.0741 (found), [M + Na]^+^ = 225.0752 (calculated).

*5-amino-1-(3-chlorophenyl)-1H-pyrazole-4-carboxamide* (**2b**)

Yield: 83%; m.p.: 168–170 °C; FT-IR ν (cm^−1^): 3415–3192, 1656, 1612–1447; ^1^H NMR (500 MHz, CDCl_3_) δ 7.64 (s, 1H), 7.61 (s, 1H), 7.49–7.43 (m, 2H), 7.39 (d, *J* = 7.2 Hz, 1H), 5.50 (br, 4H); ^13^C NMR (125 MHz, CDCl_3_) δ 166.4, 149.4, 138.8, 138.4, 135.8, 131.0, 128.4, 124.2, 121.7, 97.5; HRMS (ESI) *m/z* [M + Na]^+^ = 259.0346 (found), [M + Na]^+^ = 259.0363 (calculated).

*5-amino-1-(2,4-dichlorophenyl)-1H-pyrazole-4-carboxamide* (**2c**)

Yield: 75%; m.p.: 212–214 °C; FT-IR ν (cm^−1^): 3437–3236, 1641, 1607–1444; ^1^H NMR (400 MHz, CDCl_3_) δ 7.64 (s, 1H), 7.60-761 (m, 1H), 7.42 (s, 2H), 5.35 (br, 4H); ^13^C NMR (100 MHz, CDCl_3_) δ 166.1, 150.4, 138.5, 136.6, 133.2, 133.1, 130.7, 130.6, 128.5, 96.6; HRMS (ESI) *m/z* [M + H]^+^ = 271.0155 (found), [M + H]^+^ = 271.0148 (calculated).

*5-amino-1-(3,5-dichlorophenyl)-1H-pyrazole-4-carboxamide* (**2d**)

Yield: 78%; m.p.: 184–186 °C; FT-IR ν (cm^−1^): 3437–3236, 1641, 1607–1444; ^1^H NMR (400 MHz, DMSO-*d*_6_) δ 7.96 (s, 1H), 7.64 (d, *J* = 1.8 Hz, 2H), 7.63 (d, *J* = 1.8 Hz, 1H), 7.46 (br, 1H), 6.94 (br, 1H), 6.64 (br, 2H); ^13^C NMR (100 MHz, DMSO-*d*_6_) δ 166.3, 150.3, 140.7, 140.4, 135.0, 126.7, 121.7, 98.4; HRMS (ESI) *m/z* [M + Na]^+^ = 292.9956 (found), [M + Na]^+^ = 292.9973 (calculated).

*5-amino-1-(3,4-dichlorophenyl)-1H-pyrazole-4-carboxamide* (**2e**)

Yield: 74%; m.p.: 204–212 °C; FT-IR ν (cm^−1^): 3430–3204, 1645, 1607–1440; ^1^H NMR (400 MHz, DMSO-*d*_6_) δ 7.94 (s, 1H), 7.83 (d, *J* = 2.5 Hz, 1H), 7.78 (d, *J* = 8.7 Hz, 1H), 7.59 (dd, *J* = 8.7, 2.5 Hz, 1H), 7.44 (br, 1H), 6.91 (br, 1H), 6.56 (br, 2H); ^13^C NMR (100 MHz, DMSO-*d*_6_) δ 166.3, 150.2, 140.2, 138.5, 132.1, 131.6, 129.6, 125.0, 123.5, 98.2; HRMS (ESI) *m/z* [M + Na]^+^ = 292.9948 (found), [M + Na]^+^ = 292.9973 (calculated).

*5-amino-1-(4-chlorophenyl)-1H-pyrazole-4-carboxamide* (**2f**)

Yield: 51%; m.p.: 196–200 °C; FT-IR ν (cm^−1^): 3467–3148, 1654, 1604–1490; ^1^H NMR (400 MHz, DMSO-*d*_6_) δ 7.92 (s, 1H), 7.58 (s, 4H), 7.42 (br, 1H), 6.87 (br, 1H), 6.44 (br, 2H); ^13^C NMR (100 MHz, DMSO-*d*_6_) δ 166.4, 149.9, 139.7, 137.5, 131.6, 129.7, 125.2, 98.1; HRMS (ESI) *m/z* [M + Na]^+^ = 259.0337 (found), [M + Na]^+^ = 259.0363 (calculated).

*5-amino-1-(4-fluorophenyl)-1H-pyrazole-4-carboxamide* (**2g**)

Yield: 69%; m.p.: 232–234 °C; FT-IR ν (cm^−1^): 3482–3128, 1648, 1610–1435; ^1^H NMR (400 MHz, DMSO-*d*_6_) δ 7.89 (s, 1H), 7.58 (dd, *J* = 8.8, 4.9 Hz, 2H), 7.36 (t, *J* = 8.8 Hz, 2H), 7.35 (br, 1H), 6.85 (br, 1H), 6.35 (br, 2H); ^13^C NMR (100 MHz, DMSO-*d*_6_) δ 166.5, 161.1 (d, *J* = 244.1 Hz), 149.8, 139.4, 135.0, 126.0 (d, *J* = 8.8 Hz), 116.5 (d, *J* = 22.9 Hz), 97.9; HRMS (ESI) *m/z* [M + Na]^+^ = 243.0644 (found), [M + Na]^+^ = 243.0658 (calculated).

*5-amino-1-(3-fluorophenyl)-1H-pyrazole-4-carboxamide* (**2h**)

Yield: 51%; m.p.: 178–182 °C; FT-IR ν (cm^−1^): 3346–3196, 1656, 1612–1461; ^1^H NMR (500 MHz, DMSO-*d*_6_) δ 7.93 (s, 1H), 7.58-7.54 (m, 1H), 7.45-7.41 (m, 3H), 7.24-7.20 (m, 1H), 6.88 (br, 1H), 6.51 (br, 2H); ^13^C NMR (125 MHz, DMSO-*d*_6_) δ 165.9, 162.0 (d, *J* = 244.3 Hz), 149.4, 139.6 (d, *J* = 10.5 Hz), 139.3, 130.9 (d, *J* = 9.2 Hz), 118.6 (d, *J* = 2.7 Hz), 113.5 (d, *J* = 21.0 Hz), 109.9 (d, *J* = 25.1 Hz), 97.6; HRMS (ESI) *m/z* [M + Na]^+^ = 243.0631 (found), [M + Na]^+^ = 243.0658 (calculated).

*5-amino-1-(4-bromophenyl)-1H-pyrazole-4-carboxamide* (**2i**)

Yield: 38%; m.p.: 138–142 °C; FT-IR ν (cm^−1^): 3423–3172, 1653, 1607–1440; ^1^H NMR (400 MHz, DMSO-*d*_6_) δ 7.92 (s, 1H), 7.71 (d, *J* = 8.8 Hz, 2H), 7.53 (d, *J* = 8.8 Hz, 2H), 7.45 (br, 2H), 6.78 (br, 2H); ^13^C NMR (100 MHz, DMSO-*d*_6_) δ 166.4, 149.9, 139.8, 138.0, 132.6, 125.4, 119.9, 98.1; HRMS (ESI) *m/z* [M + Na]^+^ = 302.9837 (found), [M + Na]^+^ = 302.9857 (calculated).

*5-amino-1-(3-bromophenyl)-1H-pyrazole-4-carboxamide* (**2j**)

Yield: 84%; m.p.: 138–142 °C; FT-IR ν (cm^−1^): 3424–3188, 1657, 1587–1443; ^1^H NMR (500 MHz, CDCl_3_) δ 7.76 (s, 1H), 7.63 (s, 1H), 7.53 (pseudo-triplet, *J* = 8.8 Hz, 2H), 7.39 (t, *J* = 8.8 Hz, 1H), 5.50 (br, 4H); ^13^C NMR (125 MHz, CDCl_3_) δ 166.3, 149.4, 138.9, 138.4, 131.4, 131.2, 127.1, 123.5, 122.2, 97.5; HRMS (ESI) *m/z* [M + Na]^+^ = 302.9837 (found), [M + Na]^+^ = 302.9857 (calculated).

*5-amino-1-(4-methoxyphenyl)-1H-pyrazole-4-carboxamide* (**2k**)

Yield: 26%; m.p.: 214–218 °C; FT-IR ν (cm^−1^): 3423–3173, 1635, 1597–1458; ^1^H NMR (400 MHz, DMSO-*d*_6_) δ 7.85 (s, 1H), 7.43 (d, *J* = 9.0 Hz, 2H), 7.37 (br, 1H), 7.06 (d, *J* = 9.0 Hz, 2H), 6.81 (br, 1H), 6.21 (br, 2H), 3.80 (s, 3H); 13C NMR (100 MHz, DMSO-*d*_6_) δ 166.6, 158.6, 149.6, 138.9, 131.5, 125.5, 114.9, 97.6, 55.9; HRMS (ESI) *m/z* [M + Na]^+^ = 255.0856 (found), [M + Na]^+^ = 255.0858 (calculated).

*5-amino-1-(2,3-dichlorophenyl)-1H-pyrazole-4-carboxamide* (**2l**)

Yield: 84%; m.p.: 160–164 °C; FT-IR ν (cm^−1^): 3356–3189, 1667, 1603–1435; ^1^H NMR (500 MHz, DMSO-*d*_6_) δ 7.88 (s, 1H), 7.81 (dd, *J* = 7.9, 1.5 Hz, 1H), 7.52-7.47 (m, 2H), 7.35 (br, 1H), 6.79 (br, 1H), 6.30 (s, 2H); ^13^C NMR (125 MHz, DMSO-*d*_6_) δ 166.0, 150.6, 139.3, 136.9, 132.6, 131.4, 130.8, 129.1, 128.8, 96.0; HRMS (ESI) *m/z* [M + Na]^+^ = 292.9962 (found), [M + Na]^+^ = 292.9973 (calculated).

*1-aryl-4-(4,5-dihydro-1H-imidazol-2-yl)-1H-pyrazoles***3**(**a**–**n**)

The synthesis of **3**(**a**–**n**) was carried out according to the methodology published by our research group [27]. The analytical results are shown below.

*4-(4,5-dihydro-1H-imidazol-2-yl)-1-phenyl-1H-pyrazole* (**3a**)

Yield: 44%; m.p.: 149–150 °C; FT-IR ν (cm^−1^): 3142–3075, 2934–2848, 1630–1562; ^1^H NMR (500 MHz, DMSO-*d*_6_) δ 8.80 (s, 1H), 8.03 (s, 1H), 7.84 (d, *J* = 7.8 Hz, 2H), 7.53 (t, *J* = 7.8 Hz, 2H), 7.35 (t, *J* = 7.8 Hz, 1H), 3.56 (s, 4H); ^13^C NMR (125 MHz, DMSO-*d*_6_) δ 158.1, 140.5, 139.7, 130.1, 127.7, 127.2, 119.0, 116.6, 49.5; HRMS (ESI) *m/z* [M + H]^+^ = 213.1151 (found), [M + H]^+^ = 213.1140 (calculated).

*1-(3-chlorophenyl)-4-(4,5-dihydro-1H-imidazol-2-yl)-1H-pyrazole* (**3b**)

Yield: 47%; m.p.: 120–124 °C; FT-IR ν (cm^−1^): 3198–3113, 2941–2873, 1633–1566; ^1^H NMR (400 MHz, DMSO-*d*_6_) δ 8.91 (s, 1H), 8.06 (s, 1H), 7.95 (t, *J* = 2.0 Hz, 1H), 7.84 (dd, *J* = 8.0, 2.0 Hz, 1H), 7.55 (t, *J* = 8.0 Hz, 1H), 7.42 (dd, *J* = 8.0, 2.0 Hz, 1H), 3.57 (s, 4H); ^13^C NMR (100 MHz, DMSO-*d*_6_) δ 158.0, 141.0, 140.7, 134.5, 131.8, 128.3, 126.9, 118.7, 117.5, 116.8, 49.6; HRMS (ESI) *m/z* [M + H]^+^ = 247.0752 (found), [M + H]^+^ = 247.0750 (calculated).

*1-(2,4-dichlorophenyl)-4-(4,5-dihydro-1H-imidazol-2-yl)-1H-pyrazole* (**3c**)

Yield: 70%; m.p.: 110–111 °C; FT-IR ν (cm^−1^): 3239–3110, 2935–2873, 1626–1483; ^1^H NMR (500 MHz, DMSO-*d*_6_) δ 8.49 (s, 1H), 8.08 (s, 1H), 7.92 (d, *J* = 1.8 Hz, 1H), 7.67 (d, *J* = 8.6 Hz, 1H), 7.62 (dd, *J* = 8.6, 1.8 Hz, 1H), 3.58 (s, 4H); ^13^C NMR (125 MHz, DMSO-*d*_6_) δ 158.0, 140.6, 136.7, 134.3, 132.5, 130.5, 129.7, 129.5, 128.9, 115.2, 49.2; HRMS (ESI) *m/z* [M + H]^+^ = 281.0357 (found), [M + H]^+^ = 281.0361 (calculated).

*1-(3,5-dichlorophenyl)-4-(4,5-dihydro-1H-imidazol-2-yl)-1H-pyrazole* (**3d**)

Yield: 80%; m.p.: 83–84 °C; FT-IR ν (cm^−1^): 3174–3079, 2944–2870, 1629–1552; ^1^H NMR (500 MHz, DMSO-*d*_6_) δ 8.99 (s, 1H), 8.08 (s, 1H), 7.97 (s, 2H), 7.60 (s, 1H), 3.57 (s, 4H); ^13^C NMR (125 MHz, DMSO-*d*_6_) δ 157.4, 140.9, 135.1, 128.4, 125.9, 117.2, 117.1, 116.7, 49.2; HRMS (ESI) *m/z* [M + H]^+^ = 281.0363 (found), [M + H]^+^ = 281.0361 (calculated).

*1-(3,4-dichlorophenyl)-4-(4,5-dihydro-1H-imidazol-2-yl)-1H-pyrazole* (**3e**)

Yield: 79%; m.p.: 185–186 °C; FT-IR ν (cm^−1^): 3200–3120, 2952–2862, 1628–1581; ^1^H NMR (500 MHz, DMSO-*d*_6_) δ 8.94 (s, 1H), 8.16 (d, *J* = 2.5 Hz, 1H), 8.07 (s, 1H), 7.88 (dd, *J* = 8.8, 2.5 Hz, 1H), 7.79 (d, *J* = 8.8 Hz, 1H), 3.57 (s, 4H); ^13^C NMR (125 MHz, DMSO-*d*_6_) δ 157.3, 140.6, 138.7, 132.0, 131.4, 128.7, 128.0, 120.0, 118.4, 116.4, 49.0; HRMS (ESI) *m/z* [M + H]^+^ = 281.0362 (found), [M + H]^+^ = 281.0361 (calculated).

*1-(2,6-dichlorophenyl)-4-(4,5-dihydro-1H-imidazol-2-yl)-1H-pyrazole* (**3f**)

Yield: 76%; m.p.: 221–223 °C; FT-IR ν (cm^−1^): 3116–3069, 2941–2800, 1620–1550; ^1^H NMR (500 MHz, DMSO-*d*_6_) δ 8.31 (s, 1H), 8.06 (s, 1H), 7.73 (d, *J* = 8.3 Hz, 2H), 7.62 (t, *J* = 8.3 Hz, 1H), 3.55 (s, 4H); ^13^C NMR (125 MHz, DMSO-*d*_6_) δ 157.4, 140.0, 135.3, 133.1, 132.2, 132.0, 128.9, 115.1, 49.0; HRMS (ESI) *m/z* [M + H]^+^ = 281.0373 (found), [M + H]^+^ = 281.0361 (calculated).

*1-(4-chlorophenyl)-4-(4,5-dihydro-1H-imidazol-2-yl)-1H-pyrazole* (**3g**)

Yield: 78%; m.p.: 214–216 °C; FT-IR ν (cm^−1^): 3151–3071, 2934–2878, 1629–1504; ^1^H NMR (500 MHz, DMSO-*d*_6_) δ 8.83 (s, 1H), 8.04 (s, 1H), 7.88 (d, *J* = 8.9 Hz, 2H), 7.59 (d, *J* = 8.9 Hz, 2H), 3.56 (s, 4H); ^13^C NMR (125 MHz, DMSO-*d*_6_) δ 157.5, 140.3, 138.0, 130.8, 129.5, 127.5, 120.1, 116.3, 49.1; HRMS (ESI) *m/z* [M + H]^+^ = 247.0749 (found), [M + H]^+^ = 247.0750 (calculated).

*4-(4,5-dihydro-1H-imidazol-2-yl)-1-(4-fluorophenyl)-1H-pyrazole* (**3h**)

Yield: 87%; m.p.: 210–212 °C; FT-IR ν (cm^−1^): 3142–3105, 2934–2848, 1632–1516; ^1^H NMR (500 MHz, DMSO-*d*_6_) δ 8.78 (s, 1H), 8.02 (s, 1H), 7.87 (dd, *J* = 9.0, 4.7 Hz, 2H), 7.37 (t, *J* = 9.0 Hz, 2H), 3.55 (s, 4H); ^13^C NMR (125 MHz, DMSO-*d*6) δ 160.5 (d, *J* = 243.6 Hz), 157.6, 140.0, 135.8 (d, *J* = 2.6 Hz), 127.5, 120.6 (d, *J* = 8.6 Hz), 116.3 (d, *J* = 23.1 Hz), 116.1, 49.9; HRMS (ESI) *m/z* [M + H]^+^ = 231.1040 (found), [M + H]^+^ = 231.1046 (calculated).

*4-(4,5-dihydro-1H-imidazol-2-yl)-1-(3-fluorophenyl)-1H-pyrazole* (**3i**)

Yield: 55%; m.p.: 164–167 °C; FT-IR ν (cm^−1^): 3170–3075, 2934–2852, 1631–1564; ^1^H NMR (500 MHz, DMSO-*d*_6_) δ 8.88 (s, 1H), 8.05 (s, 1H), 7.75-7.72 (m, 2H), 7.59-7.55 (m, 1H), 7.21-7.18 (m, 1H), 3.56 (s, 4H); ^13^C NMR (125 MHz, DMSO-*d*_6_) δ 162.5 (d, *J* = 244.0 Hz), 157.5, 140.6 (d, *J* = 10.6 Hz), 140.4, 131.5 (d, *J* = 9.4 Hz), 127.8, 116.4, 114.3 (d, *J* = 2.6 Hz), 113.3 (d, *J* = 21.1 Hz), 105.8 (d, *J* = 26.7 Hz), 49.3; HRMS (ESI) *m/z* [M + H]^+^ = 231.1048 (found), [M + H]^+^ = 231.1046 (calculated).

*1-(4-bromophenyl)-4-(4,5-dihydro-1H-imidazol-2-yl)-1H-pyrazole* (**3j**)

Yield: 65%; m.p.: 228–230 °C; FT-IR ν (cm^−1^): 3144–3110, 2931–2863, 1632–1565; ^1^H NMR (500 MHz, DMSO-*d*_6_) δ 8.83 (s, 1H), 8.04 (s, 1H), 7.81 (d, *J* = 8.8 Hz, 2H), 7.72 (d, *J* = 8.8 Hz, 2H), 3.56 (s, 4H); ^13^C NMR (125 MHz, DMSO-*d*_6_) δ 157.5, 140.3, 138.4, 132.4, 127.5, 120.4, 119.0, 116.4, 49.1; HRMS (ESI) *m/z* [M + H]^+^ = 291.0256 (found), [M + H]^+^ = 291.0245 (calculated).

*1-(3-bromophenyl)-4-(4,5-dihydro-1H-imidazol-2-yl)-1H-pyrazole* (**3k**)

Yield: 61%; m.p.: 116–120 °C; FT-IR ν (cm^−1^): 3178–3099, 2936–2867, 1627–1551; ^1^H NMR (500 MHz, DMSO-*d*_6_) δ 8.91 (s, 1H), 8.07 (t, *J* = 1.8 Hz, 1H), 8.06 (s, 1H), 7.88 (d, *J* = 8.1 Hz, 1H), 7.55 (d, *J* = 8.1 Hz, 1H), 7.48 (t, *J* = 8.0 Hz, 1H), 3.58 (s, 4H); ^13^C NMR (125 MHz, DMSO-*d*_6_) δ 157.5, 140.5, 140.3, 131.6, 129.4, 127.9, 122.3, 121.1, 117.4, 116.1, 49.0; HRMS (ESI) *m/z* [M + H]^+^ = 291.0253 (found), [M + H]^+^ = 291.0245 (calculated).

*4-(4,5-dihydro-1H-imidazol-2-yl)-1-(4-methoxyphenyl)-1H-pyrazole* (**3l**)

Yield: 57%; m.p.: 196–197 °C; FT-IR ν (cm^−1^): 3142–3101, 2934–2844, 1631–1518; ^1^H NMR (500 MHz, DMSO-*d*_6_) δ 8.68 (s, 1H), 7.98 (s, 1H), 7.74 (d, *J* = 9.0 Hz, 2H), 7.07 (d, *J* = 9.0 Hz, 2H), 3.80 (s, 3H), 3.55 (s, 4H); ^13^C NMR (125 MHz, DMSO-*d*_6_) δ 158.4, 158.2, 140.0, 133.3, 127.6, 120.6, 116.1, 115.1, 55.9, 49.6; HRMS (ESI) *m/z* [M + H]^+^ = 243.1255 (found), [M + H]^+^ = 243.1246 (calculated).

*1-(3-chloro-4-methylphenyl)-4-(4,5-dihydro-1H-imidazol-2-yl)-1H-pyrazole* (**3m**)

Yield: 92%; m.p.: 168–170 °C; FT-IR ν (cm^−1^): 3199–3122, 2941–2879, 1627–1564; ^1^H NMR (500 MHz, DMSO-*d*_6_) δ 8.85 (s, 1H), 8.02 (s, 1H), 7.92 (d, *J* = 2.2 Hz, 1H), 7.74 (dd, *J* = 8.3, 2.2 Hz, 1H), 7.50 (d, *J* = 8.3 Hz, 1H), 3.55 (s, 4H), 2.36 (s, 3H); ^13^C NMR (125 MHz, DMSO-*d*_6_) δ 157.5, 140.2, 138.2, 134.0, 133.7, 132.1, 127.6, 118.6, 117.0, 116.2, 49.2, 19.0; HRMS (ESI) *m/z* [M + H]^+^ = 261.0929 (found), [M + H]^+^ = 261.0907 (calculated).

*1-(4-chloro-2-methylphenyl)-4-(4,5-dihydro-1H-imidazol-2-yl)-1H-pyrazole* (**3n**)

Yield: 37%; m.p.: 174–176 °C; FT-IR ν (cm^−1^): 3108, 2962–2869, 1621–1547; ^1^H NMR (500 MHz, DMSO-*d*_6_) δ 8.35 (s, 1H), 8.02 (s, 1H), 7.54 (s, 1H), 7.42 (s, 2H), 3.56 (s, 4H), 2.21 (s, 3H); ^13^C NMR (125 MHz, DMSO-*d*_6_) δ 157.6, 139.5, 137.9, 135.0, 132.6, 131.2, 130.8, 127.3, 126.6, 114.7, 48.8, 17.5; HRMS (ESI) *m/z* [M + H]^+^ = 261.0927 (found), [M + H]^+^ = 261.0907 (calculated).

*5-amino-1-aryl-3-methyl-4-(4,5-dihydro-1H-imidazol-2-yl)-1H-pyrazoles***4**(**a**–**c**)

In the first step, the corresponding arylhydrazine hydrochloride (0.01 mol) was stirred with sodium acetate (0.02 mol) in ethanol (20 mL), under reflux. Then, 1-ethoxyethylidenemalononitrile (0.01 mol) was added and, after further stirring for 1 h under reflux, the mixture was poured into cold water, and the precipitate was filtered out and recrystallized from ethanol/water. The key intermediates 5-amino-1-aryl-3-methyl-1*H*-pyrazoles **6**(**a**–**c**) were obtained in good yields: 80-93%. After that, a mixture of **6**(**a**–**c**) (0.001 mol) and ethylenediamine (2 mL) was stirred and kept at room temperature. CS_2_ (0.004 mol) was added dropwise, and the mixture was heated at 110–120 °C for 12–14 h. The product was filtered out and washed with cold water to yield **4**(**a**–**c**) (63–89%).

*5-amino-1-(3-chlorophenyl)-3-methyl-4-(4,5-dihydro-1H-imidazol-2-yl)-1H-pyrazole* (**4a**)

Yield: 89%; m.p.: 116–120 °C; FT-IR ν (cm^−1^): 3409–3267, 2963–2875, 1594–1409; ^1^H NMR (400 MHz, CDCl_3_) δ 7.61 (t, *J* = 2.0 Hz, 1H), 7.47 (ddd, *J* = 8.0, 1.8, 1.2 Hz, 1H), 7.40 (t, *J* = 8.0 Hz, 1H), 7.31 (ddd, *J* = 8.0, 1.8, 1.2 Hz, 1H), 3.69 (s, 4H), 2.41 (s, 3H); ^13^C NMR (100 MHz, CDCl_3_) δ 161.2, 148.3, 146.9, 139.2, 135.4, 130.6, 127.4, 123.6, 121.0, 93.8, 49.8, 14.5; HRMS (ESI) *m/z* [M + H]^+^ = 276.1011 (found), [M + H]^+^ = 276.1016 (calculated).

*5-amino-1-(3,5-dichlorophenyl)-3-methyl-4-(4,5-dihydro-1H-imidazol-2-yl)-1H-pyrazole* (**4b**)

Yield: 63%; m.p.: 193–194 °C; FT-IR ν (cm^−1^): 3433–3257, 2984–2867, 1605–1427; ^1^H NMR (400 MHz, CDCl_3_) δ 7.53 (d, *J* = 1.8 Hz, 2H), 7.30 (t, *J* = 1.8 Hz, 1H), 3.68 (s, 4H), 2.40 (s, 3H); ^13^C NMR (100 MHz, CDCl_3_) δ 161.0, 148.5, 147.4, 140.0, 135.9, 127.0, 121.2, 94.2, 49.6, 14.5; HRMS (ESI) *m/z* [M + H]^+^ = 310.0618 (found), [M + H]^+^ = 310.0626 (calculated).

*5-amino-1-(3,4-dichlorophenyl)-3-methyl-4-(4,5-dihydro-1H-imidazol-2-yl)-1H-pyrazole* (**4c**)

Yield: 64%; m.p.: 221–224 °C; FT-IR ν (cm^−1^): 3438–3285, 2970–2859, 1602–1439; ^1^H NMR (400 MHz, CDCl_3_) δ 7.73 (d, *J* = 2.4 Hz, 1H), 7.54 (d, *J* = 8.6 Hz, 1H), 7.45 (dd, *J* = 8.6, 2.4 Hz, 1H), 3.68 (s, 4H), 2.40 (s, 3H); ^13^C NMR (100 MHz, CDCl_3_) δ 161.1, 148.4, 147.1, 137.5, 133.6, 131.2, 131.1, 125.0, 122.0, 94.1, 49.8, 14.5; HRMS (ESI) *m/z* [M + H]^+^ = 310.0616 (found), [M + H]^+^ = 310.0626 (calculated).

*5-amino-1-aryl-4-(4(5)-methyl-4,5-dihydro-1H-imidazol-2-yl)-1H-pyrazoles***5**(**a**–**c**)

The first step is similar to the methodology to synthesize **6**(**a**–**c**). Arylhydrazine hydrochloride (0.01 mol) was stirred with sodium acetate (0.02 mol) in ethanol (20 mL), under reflux. In the next step, 1ethoxymethylenemalononitrile (0.01 mol) was added and the mixture was kept under reflux for 1 h. Thereafter, it was poured into cold water, the precipitate was filtered out and recrystallized from ethanol/water. The intermediates 5-amino-1-aryl-3-methyl-1*H*-pyrazoles **7**(**a**–**c**) were synthesized in good yields: 85–92%. Finally, a mixture of **7**(**a**–**c**) (0.001 mol) with 1,2-diaminopropane (2 mL) was stirred without heating. CS_2_ (0.004 mol) was added dropwise and the reaction was kept at 110–125 °C for 14–18 h. The solid was filtered out and washed with cold water to give **5**(**a**–**c**) (24–80%).

*5-amino-1-(3-chlorophenyl)-4-(4-(5)-methyl-4,5-dihydro-1H-imidazol-2-yl)-1H-pyrazole* (**5a**)

Yield: 24%; m.p.: 169–172 °C; FT-IR ν (cm^−1^): 3347–3235, 2966–2851, 1605–1425; ^1^H NMR (400 MHz, CDCl_3_) δ 7.63 (t, *J* = 1.9 Hz, 1H), 7.54 (s, 1H), 7.50 (ddd, *J* = 8.0, 2.0, 1.2 Hz, 1H), 7.43 (t, *J* = 8.0 Hz, 1H), 7.34 (ddd, *J* = 8.0, 2.0, 1.2 Hz, 1H), 4.04 (s, 1H), 3.84 (s, 1H), 3.29 (s, 1H), 1.27 (d, *J* = 6.3 Hz, 3H); ^13^C NMR (100 MHz, CDCl_3_) δ 158.8, 147.1, 139.3, 138.0, 135.4, 130.6, 127.7, 123.6, 121.1, 95.1, 21.9; HRMS (ESI) *m/z* [M + H]^+^ = 276.1007 (found), [M + H]^+^ = 276.1016 (calculated).

*5-amino-1-(3,5-dichlorophenyl)-4-(4-(5)-methyl-4,5-dihydro-1H-imidazol-2-yl)-1H-pyrazole* (**5b**)

Yield: 46%; m.p.: 189–192 °C; FT-IR ν (cm^−1^): 3313–3236, 2963–2864, 1611–1429; ^1^H NMR (400 MHz, CDCl_3_) δ 7.56 (d, *J* = 1.8 Hz, 2H), 7.54 (s, 1H), 7.34 (t, *J* = 1.8 Hz, 1H), 4.05 (s, 1H), 3.85 (s, 1H), 3.29 (s, 1H), 1.27 (d, *J* = 6.3 Hz, 3H); ^13^C NMR (100 MHz, CDCl_3_) δ 158.6, 147.2, 140.0, 138.4, 136.0, 127.4, 121.3, 95.5, 21.9; HRMS (ESI) *m/z* [M + H]^+^ = 310.0614 (found), [M + H]^+^ = 310.0626 (calculated).

*5-amino-1-(3,4-dichlorophenyl)-4-(-4(5)-methyl-4,5-dihydro-1H-imidazol-2-yl)-1H-pyrazole* (**5c**)

Yield: 80%; m.p.: 191–193 °C; FT-IR ν (cm^−1^): 3397–3255, 2963–2856, 1608–1426; ^1^H NMR (400 MHz, CDCl_3_) δ 7.75 (d, *J* = 2.4 Hz, 1H), 7.56 (d, *J* = 8.6 Hz, 1H), 7.54 (s, 1H), 7.48 (dd, *J* = 8.6, 2.4 Hz, 1H), 4.05 (s, 1H), 3.84 (s, 1H), 3.29 (s, 1H), 1.27 (d, *J* = 6.3 Hz, 3H); ^13^C NMR (100 MHz, CDCl_3_) δ 158.7, 147.2, 138.2, 137.6, 133.7, 131.5, 131.2, 125.1, 122.1, 95.4, 21.9; HRMS (ESI) *m/z* [M + H]^+^ = 310.0611 (found), [M + H]^+^ = 310.0626 (calculated).

### 3.2. Cell Culture

#### 3.2.1. Two-Dimensional Culture (2D)

Vero cells, obtained from the Rio de Janeiro Cell Bank (BCRJ code 0245), were cultured in RPMI-1640 supplemented with 10% fetal bovine serum (FBS) and 1 mM l-glutamine, as previously described [49]. Vero cells were used for drug screening assays and to obtain trypomastigote forms of *T. cruzi*.

#### 3.2.2. Three-Dimensional Culture (3D)

Vero cells were seeded at a density of 2 × 10^5^ cells/well in a 96-well U-bottom plate previously coated with agarose (1%). The 3D cultures, adapted from [42], were cultivated for 4 days in RPMI 1640 medium supplemented with 10% FBS and l-glutamine for complete 3D microtissue formation. The 3D culture model was used as a tool to evaluate the effectiveness of promising derivatives in eliminating intracellular parasites in the microtissue.

### 3.3. Parasites and Culture Infection

*T. cruzi* Dm28c clone genetically modified to express luciferase, kindly provided by Dr. Cristina Henriques from Oswaldo Cruz Institute–Fiocruz, was used in the drug assays [50]. Trypomastigotes were harvested from *T. cruzi*-infected Vero cultures supernatants at 4th days post-infection (dpi) and used in the phenotypic screening assay. For intracellular amastigotes assays, Vero cells (2D and 3D cultures) were infected at a ratio of 10:1 parasites/host cells.

### 3.4. Cytotoxicity In Vitro Assay

Vero cells were used as a cellular model to analyze the toxic effect of pyrazole derivatives, series **1**(**a**–**l**), **2**(**a**–**l**), **3**(**a**–**n**), **4**(**a**–**c**), and **5**(**a**–**c**), and Benznidazole (Bz) on mammalian cells. Briefly, the cells, plated on 96-well white opaque microplate (1.5 × 10^4^ cells/well), were treated for 72 h at 37 °C with a wide range concentration (500–1.95 µM) of the pyrazole derivatives [13]. The cell viability was determined by measuring ATP levels using the CellTiter Glo Kit (Promega Corporation, Madison, WI, EUA) followed by reading the luminescent signal on the Glomax-Multi Detection plate reader (Promega Corporation, Madison, WI, EUA)). All treatments and controls were performed at a low concentration of DMSO (≤1%). The 50% cytotoxic concentration values (CC_50_; the concentration of the compound that reduces 50% of the cell viability) were estimated by linear regression analysis. Three independent assays were performed in duplicate.

### 3.5. Anti-T. cruzi Compound Screening

The phenotypic compound screening was performed using transgenic *T. cruzi* parasites expressing the firefly luciferase (Dm28c-Luc). Trypomastigotes (1 × 10^6^ parasites/well) were treated for 24 h at 37 °C with the pyrazole derivatives and Bz (0.04–100 μM), followed by incubation with luciferin substrate (300 µg/mL) to determine parasite viability through luciferase activity [13]. The concentration that reduces the number of viable parasites by 50% (IC_50_) or 90% (IC_90_) was calculated by linear regression. The luminescent signal was read on a Glomax-Multi Detection plate reader (Promega Corporation, Madison, WI, EUA)). At least three independent assays were performed in duplicate.

The effect of the pyrazole derivatives against intracellular amastigotes was also evaluated after 72 h treatment (37 °C) with compound concentrations ranging from 0.04–100 µM [13]. Parasite viability, measured by luminescent signal, was determined after luciferin (300 µg/mL) addition. IC_50_ and IC_90_ values were calculated by linear regression. Selectivity index (SI) was calculated as the ratio of the CC_50_ to IC_50_ values. The non-toxic concentration of DMSO (less than 1% *v*/*v*) was used in all assays. A minimum of three independent assays was carried out in duplicate.

The 3D-microtissue-based phenotypic screening was performed with the promising pyrazole derivatives. *T. cruzi*-infected spheroids (24 h) were treated for 72 h at 37 °C with promising compounds at concentrations of IC_90_ and 2 × IC_90_. At the end of the treatment, the 3D cultures supernatants were removed, and luciferin (300 μg/mL) was added to the spheroids. The luminescent signal was read on the Glomax-Multi Detection reader (Promega Corporation, Madison, WI, USA). Bz (100 µM) and DMSO (≤1%) were used as positive and negative control, respectively. The IC_50_ value was calculated by linear regression. At least three independent assays were performed in quadruplicate.

### 3.6. Fluorescence Microscopy

Infected microtissues, treated or not with promising pyrazole derivatives or Bz, were fixed for 20 min at 4 °C with 4% paraformaldehyde in PBS. After washing, the spheroids were stained with 4′,6-Diamidine-2′-phenylindole dihydrochloride (DAPI; 10 µg/mL), a DNA dye, and observed under the Zeiss Axio Imager M2 fluorescence microscope (Carl Zeiss, Baden-Württemberg, Germany).

### 3.7. Reversibility Assay (Washout)

The washout assay, adapted from [36], was performed to evaluate the promising compounds efficacy. Monolayers of Vero cells infected with *T. cruzi* (24 h) were treated for 72 h at 37 °C with the promising pyrazole derivatives at the concentration of IC_90_ and 2-fold the IC_90_ concentration. After successive washing, the cultures were maintained for an additional 72 h at 37 °C in RPMI 1640 medium supplemented with 10% SFB in the absence of the compound. Luciferin (300 µg/mL) was added separately to the culture supernatant, previously removed, and the cell monolayer. The luminescent signal was read on the Glomax-Multi Detection reader (Promega Corporation, Madison, WI, USA). Bz (100 µM) and DMSO (≤1%) were used as positive and negative reaction controls, respectively.

### 3.8. Enzyme Activity in Solution

Total protein extract from trypomastigotes (Dm28c-Luc; 10^8^ parasites/mL) were extracted with lysis buffer [13]. Cysteine protease activity of extracts (5 μg) was measured in acetate buffer using fluorogenic peptide substrate (60 μM of 7-Amino-4-Methylcoumarin hydrochloride, CBZ-L-Phenylalanyl-l-Arginine amide (Z-FR-AMC) [13]. Total protein extracts were incubated for 45 min at room temperature with the compounds and changes in relative fluorescence units monitored using a SpectraMaxM2e spectrophotometer (Molecular Devices, Sunnyvale, CA, USA) for 1 h with excitation at 370 nm and emission at 460 nm. The inhibition assays were performed by coincubation with different compounds (**3g**, **3j**, and **3m**) and concentrations (300–11.11 µM) and transepoxysuccinyl-l-leucylamido-(4-guanidino) butane (E-64). Additional control was carried out with DMSO (≤1%). Enzymatic activity was expressed in µmol/min/mg of protein and the residual activity in percentage.

### 3.9. Physicochemical and ADMET Prediction

Physicochemical properties associated with compounds were calculated using Datawarrior software version 4.7.3 [51] and FAFDrugs4 [52]. For target search, Datawarrior was used to retrieve molecules within the ChEMBL database annotated to target *T. cruzi* proteins. Compounds were filtered by IC_50_ and potency (≤10 µM), and any duplicity was removed. Then, fragments of pyrazole-imidazoline and pyrazole-tetrahydropyrimidine were queried against the small library of compounds. ADMET parameters (adsorption, distribution, metabolism, elimination, and toxicity) were acquired by inserting compounds′ molecular structures in the ADMETsar platform [53].

## 4. Conclusions

Five series of pyrazole derivatives, pyrazole-carboxamide (series **1**(**a**–**l**) and **2**(**a**–**l**)) and pyrazole-imidazoline (**3**(**a**–**n**), **4**(**a**–**c**) and **5**(**a**–**c**)), were designed based on the hit compound [13], which has cruzipain as a target, aiming to optimize the activity against *Trypanosoma cruzi*. In silico analyzes of physicochemical properties revealed a good oral bioavailability profile, without violating Lipinski′s rule. Pyrazole derivatives showed no cytotoxicity, reaching values of CC_50_ > 160 µM. The phenotypic screening demonstrated that, in general, the synthesized pyrazole derivatives have low activity against trypomastigotes (IC_50_ > 34 µM), but three pyrazole-imidazoline analogs (**3g**, **3j**, and **3m**) showed activity against intracellular amastigotes similar to Bz (IC_50_ = 4.67 ± 0.22 µM), reaching IC_50_ values between 2.75 and 6.09 µM and high selectivity index (SI > 40). SAR analysis revealed that the introduction of substituents, such as Cl, Br, and methyl, in the *para* position of the benzene ring causes an increase in the potency of the compounds, with **3j** (pIC50 = 5.56) and **3m** (pIC50 = 5.45) having greater potency than the Bz (pIC50 = 5.33). Furthermore, the *ortho* position of the benzene ring appears to be a suboptimal domain for substitutions. The washout assay demonstrated that all derivatives induced a significant reduction in the parasite load, but only **3m** had similar efficacy to Bz (100 µM), without parasitism recrudescence at a concentration of 2 times the IC_90_ value (50 µM). The activity of promising pyrazole derivatives was also evaluated in the 3D model of Vero cells, mimicking the three-dimensional organization and physiology of tissues. The **3g**, **3j**, and **3m** derivatives caused a significant reduction in the parasitic load, highlighting **3m** analog, which presented an effect similar to Bz, with an evident reduction in parasitism in the microtissue.

The prediction of ADMET properties demonstrates that the promising compounds have good absorption, may undergo biotransformation, show no mutagenic or carcinogenic profile, but present hepatotoxicity and hERG inhibition. Finally, the enzymatic activity analysis, using a fluorogenic substrate, demonstrated that the analogs **3g**, **3j**, and **3m** are not potent cysteine protease inhibitors, with a maximum value of 26% inhibition of this enzyme. Together, the data reinforce the trypanocidal potential of the pyrazole-imidazoline hybrid, whose structural changes benefited the activity against intracellular amastigotes in 2D and 3D culture models, suggestive of good tissue permeability. Compound **3m** stands out for its sustainable trypanocidal activity, without the recrudescence of the parasitism in vitro. New optimization proposals will be developed in order to enhance the trypanocidal activity and advance to pre-clinical in vivo trials, contributing to the identification of compounds with potential to continue in clinical trials for the therapy of Chagas disease.

## Figures and Tables

**Table 1:** Cytotoxicity and trypanocidal effect of pyrazoles derivatives.

Compounds	Trypanocidal Activity (Mean ± SD μM)						Cytotoxicity (Mean ± SD μM)
	Trypomastigotes			Intracellular Amastigotes			
	IC_50_	IC_90_	SI	IC_50_	IC_90_	SI	CC_50_
Series **1**(**a**–**l**)	>100	Nd	Nd	>50	Nd	Nd	>500
Series **2**(**a**–**l**)	>100	Nd	Nd	>70	Nd	Nd	>500
**3a**	>100	Nd	Nd	91.82 ± 2.03	>100	>5.44	>500
**3b**	>100	Nd	Nd	28.16 ± 1.81	86.60 ± 5.55	9.48	267.10 ± 28.15
**3c**	>100	Nd	Nd	56.64 ± 2.52	94.61 ± 3.61	7.21	408.69 ± 16.17
**3d**	66.30 ± 5.53	93.48 ± 1.56	5.78	54.91 ± 7.12	>100	6.99	383.83 ± 22.97
**3e**	64.86 ± 2.85	93.74 ± 0.73	7.39	76.52 ± 8.79	>100	6.26	479.66 ± 21.67
**3f**	>100	Nd	Nd	82.00 ± 2.52	>100	>6	>500
**3g**	64.60 ± 1.56	>100	4.27	6.09 ± 0.52	26.10 ± 14.31	45.52	277.24 ± 15.76
**3h**	>100	Nd	Nd	64.50 ± 3.81	>100	7.80	>500
**3i**	>100	Nd	Nd	64.12 ± 7.09	>100	7.80	>500
**3j**	57.71 ± 3.14	95.32 ± 5.43	5.36	2.75 ± 0.62	9.67 ± 0.30	112.48	309.33 ± 34.31
**3k**	>100	Nd	Nd	26.92 ± 2.19	72.85 ± 3.67	7.84	211.04 ± 31.68
**3l**	>100	Nd	Nd	71.76 ± 2.71	>100	>7	>500
**3m**	34.54 ± 8.32	>100	4.64	3.58 ± 0.25	21.37 ± 1.25	44.83	160.51 ± 16.13
**3n**	>100	Nd	Nd	24.68 ± 3.42	85.72 ± 5.03	13.86	342.17 ± 29.99
**4a**	>100	>100	Nd	>100	>100	>5	>500
**4b**	>100	>100	Nd	90.67 ± 13.17	>100	>5.51	>500
**4c**	>100	>100	Nd	93.83 ± 16.40	>100	3.78	355.4 ± 17.72
**5a**	>100	>100	Nd	>100	>100	Nd	>500
**5b**	>100	>100	Nd	32.95 ± 8.84	>100	>15.17	>500
**5c**	>100	>100	Nd	34.33 ± 4.24	>100	>9.98	342.93 ± 37.0
**Bz**	18.71 ± 4.58	>100	>26.7	4.67 ± 0.22	>100	>107	>500

Mean values of IC_50_ and IC_90_ from three independent experiments ± standard deviation (SD); IC_50_: Concentration that inhibits parasite proliferation by 50%; CC_50_: Concentration that reduces the viability of Vero cells by 50%; Nd = Not determined; Selectivity index (SI) = CC_50_ of Vero cells/IC_50_ of trypomastigote and intracellular amastigote forms of *T. cruzi*.

**Table 2:** Structure-activity relationship (SAR) analysis of pyrazole derivatives (pyrazole-carboxamide and pyrazole-imidazoline series).

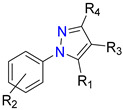						
Compounds	Structures	R1	R2	R3	R4	Intracellular Amastigotes
						pIC50
**1a**	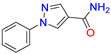	H	H	C(O)NH_2_	H	<4.3
**1b**	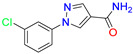	H	3-Cl	C(O)NH_2_	H	<4.3
**1c**	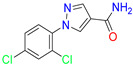	H	2,4-diCl	C(O)NH_2_	H	<4.3
**1d**	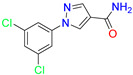	H	3,5-diCl	C(O)NH_2_	H	<4.3
**1e**	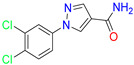	H	3,4-diCl	C(O)NH_2_	H	<4.3
**1f**	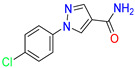	H	4-Cl	C(O)NH_2_	H	<4.3
**1g**	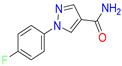	H	4-F	C(O)NH_2_	H	<4.3
**1h**	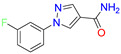	H	3-F	C(O)NH_2_	H	<4.3
**1i**	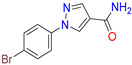	H	4-Br	C(O)NH_2_	H	<4.3
**1j**	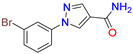	H	3-Br	C(O)NH_2_	H	<4.3
**1k**	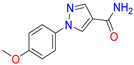	H	4-OCH_3_	C(O)NH_2_	H	<4.3
**1l**	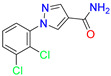	H	2,3-diCl	C(O)NH_2_	H	<4.3
**2a**	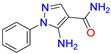	NH_2_	H	C(O)NH_2_	H	<4.15
**2b**	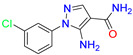	NH_2_	3-Cl	C(O)NH_2_	H	<4.15
**2c**	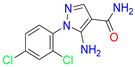	NH_2_	2,4-diCl	C(O)NH_2_	H	<4.15
**2d**	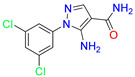	NH_2_	3,5-diCl	C(O)NH_2_	H	<4.15
**2e**	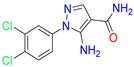	NH_2_	3,4-diCl	C(O)NH_2_	H	<4.15
**2f**	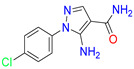	NH_2_	4-Cl	C(O)NH_2_	H	<4.15
**2g**	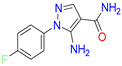	NH_2_	4-F	C(O)NH_2_	H	<4.15
**2h**	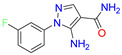	NH_2_	3-F	C(O)NH_2_	H	<4.15
**2i**	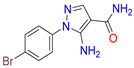	NH_2_	4-Br	C(O)NH_2_	H	<4.15
**2j**	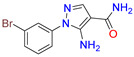	NH_2_	3-Br	C(O)NH_2_	H	<4.15
**2k**	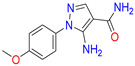	NH_2_	4-OCH_3_	C(O)NH_2_	H	<4.15
**2l**	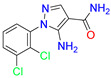	NH_2_	2,3-diCl	C(O)NH_2_	H	<4.15
**3a**	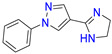	H	H	C_3_H_5_N_2_	H	4.03
**3b**	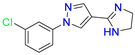	H	3-Cl	C_3_H_5_N_2_	H	4.55
**3c**	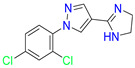	H	2,4-diCl	C_3_H_5_N_2_	H	4.25
**3d**	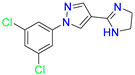	H	3,5-diCl	C_3_H_5_N_2_	H	4.26
**3e**	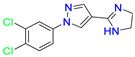	H	3,4-diCl	C_3_H_5_N_2_	H	4.12
**3f**	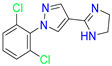	H	2,6-diCl	C_3_H_5_N_2_	H	4.09
**3g**	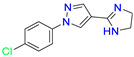	H	4-Cl	C_3_H_5_N_2_	H	5.22
**3h**	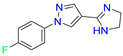	H	4-F	C_3_H_5_N_2_	H	4.19
**3i**	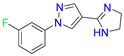	H	3-F	C_3_H_5_N_2_	H	4.19
**3j**	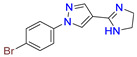	H	4-Br	C_3_H_5_N_2_	H	5.56
**3k**	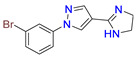	H	3-Br	C_3_H_5_N_2_	H	4.57
**3l**	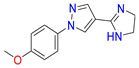	H	4-OCH_3_	C_3_H_5_N_2_	H	4.14
**3m**	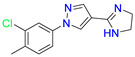	H	3-Cl,4-CH_3_	C_3_H_5_N_2_	H	5.45
**3n**	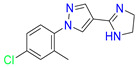	H	2-CH_3_,4-Cl	C_3_H_5_N_2_	H	4.61
**4a**	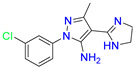	NH_2_	3-Cl	C_3_H_5_N_2_	CH_3_	<4
**4b**	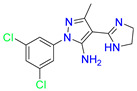	NH_2_	3,5-diCl	C_3_H_5_N_2_	CH_3_	4.04
**4c**	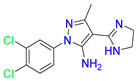	NH_2_	3,4-diCl	C_3_H_5_N_2_	CH_3_	4.03
**5a**	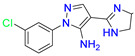	NH_2_	3-Cl	C_4_H_7_N_2_	H	<4
**5b**	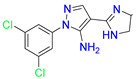	NH_2_	3,5-diCl	C_4_H_7_N_2_	H	4.48
**5c**	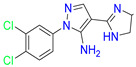	NH_2_	3,4-diCl	C_4_H_7_N_2_	H	4.46
**Bz**	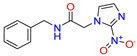	-	-	-		5.33

The reported values have a maximum standard deviation value of ±0.75 based on at least 3 experimental assays (*n* ≥ 3). pIC50 is the negative log of the IC_50_ value when converted to molar. Higher values of pIC50 indicate exponentially more potent inhibitors.

**Table 3:** In silico prediction of ADMET properties.

Properties	Compounds Result (Probability %)			
	Bz	3g	3j	3m
**Absorption**				
Human Intestinal Absorption	+0.95	+0.99	+0.99	+1.00
Caco-2	+0.74	+0.89	+0.83	+0.82
Blood-brain barrier	+0.98	+0.99	+0.99	+0.99
Human oral bioavailability	+0.73	+0.87	+0.84	+0.83
**Distribution**				
Subcellular localization	Mitochondria 0.77	Mitochondria 0.38	Lysosome 0.40	Mitochondria 0.47
**Metabolism**				
P-glycoprotein inhibitor	−0.96	−0.94	−0.95	−0.95
P-glycoprotein substrate	−0.80	−0.70	−0.63	+0.61
CYP3A4 substrate	−0.51	+0.55	−0.53	+0.58
CYP2C9 substrate	−0.78	−1.00	−1.00	−0.79
CYP2D6 substrate	−0.91	−0.90	−0.89	−0.91
CYP3A4 inhibition	−0.82	−0.93	−0.88	−0.88
CYP2C9 inhibition	−0.90	−0.67	−0.66	−0.66
CYP2C19 inhibition	−0.82	−0.69	−0.71	−0.59
CYP2D6 inhibition	−0.92	−0.82	−0.85	−0.75
CYP1A2 inhibition	−0.80	+0.88	+0.88	+0.88
CYP inhibitory promiscuity	−0.58	−0.66	−0.56	−0.54
**Toxicity**				
Carcinogenicity (binary)	−0.83	−0.89	−0.89	−0.87
Carcinogenicity (trinary)	Warning 0.41	No 0.50	No 0.48	No 0.55
Ames mutagenesis	+0.93	−0.79	−0.77	−0.80
hERG inhibition	−0.65	+0.76	+0.76	+0.81
Hepatotoxicity	+0.73	+0.88	+0.65	+0.63
Acute Oral Toxicity	III 0.60	III 0.59	III 0.60	III 0.58

The acute oral toxicity of compounds was classified based on the EPA (US Environmental Protection Agency) criteria. Class III-LD50 from 500 mg/kg to 5000 mg/kg.

## Data Availability

Data not contained within the article can be found in the attached Supplementary Materials.

## Supplementary Materials

The following are available online. Figures S1–S4: Bioavailability radar charts; Figure S5: NMR spectra of the compounds.

## Sample Availability

Samples of the compounds **1**(**a**–**l**), **2**(**a**–**l**), **3**(**a**–**n**), **4**(**a**–**c**) and **5**(**a**–**c**) are not available from the authors.
