# Supplementary material for: Structural Optimization and Biological Activity of Pyrazole Derivatives: Virtual Computational Analysis, Recovery Assay and 3D Culture Model as Potential Predictive Tools of Effectiveness against Trypanosoma cruzi
Source: Molecules. 2021 Nov 8;26(21):6742. doi: 10.3390/molecules26216742 (PMC8587750; doi:10.3390/molecules26216742)
Supplement: Supplementary file 1 [file molecules-26-06742-s001.zip › Supplementary data S1-S4.pdf]

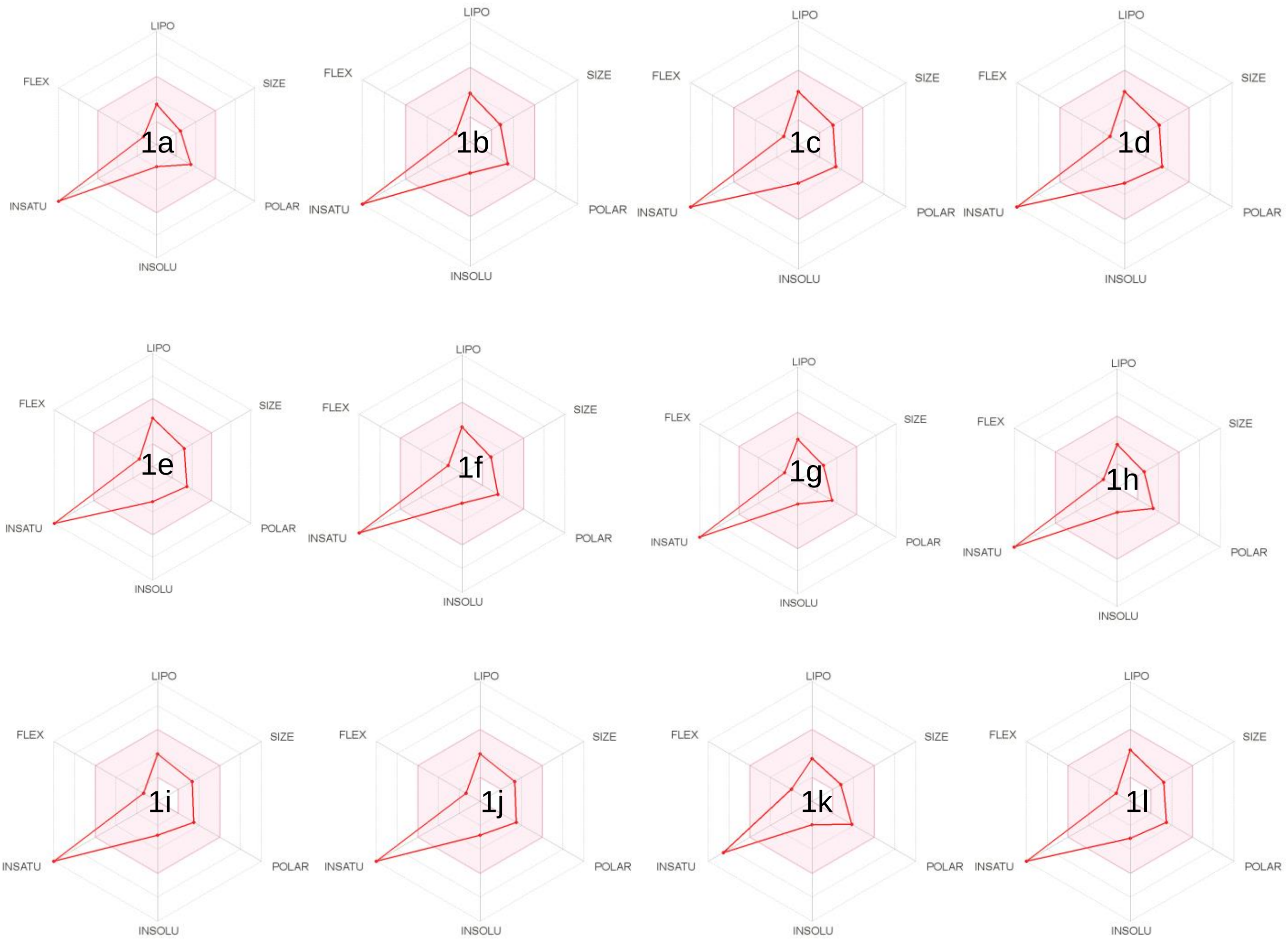

Figure S1. Bioavailability radar chart of pyrazole-carboxamide hybrids (series 1). LIPO (lipophilicity) = XLOGP3 between -0.7 and +5.0; SIZE (molecule size) = between 150 and 500 g/mol; POLAR (polarity) = TPSA between 20 and 130 Å2; INSOLU (insolubility)= LOG S <6; INSATU (saturation)= sp3 carbon fraction ≥0.25; FLEX (flexibility) ≤9 rotatable groups.

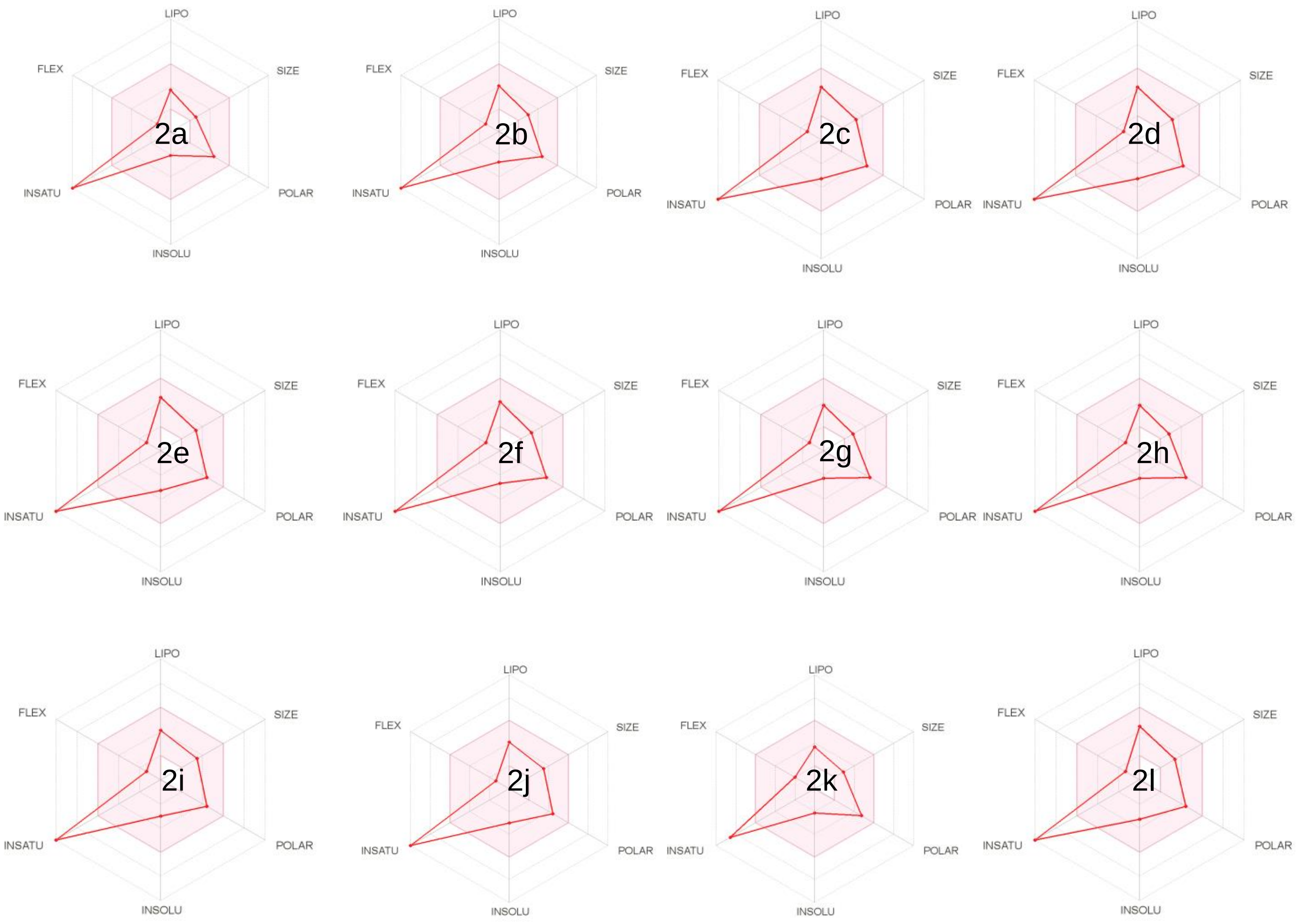

Figure S2. Bioavailability radar chart of pyrazole-carboxamide hybrids (series 2). LIPO (lipophilicity) = XLOGP3 between -0.7 and +5.0; SIZE (molecule size) = between 150 and 500 g/mol; POLAR (polarity) = TPSA between 20 and 130 Å2; INSOLU (insolubility)= LOG S <6; INSATU (saturation)= sp3 carbon fraction ≥0.25; FLEX (flexibility) ≤9 rotatable groups.

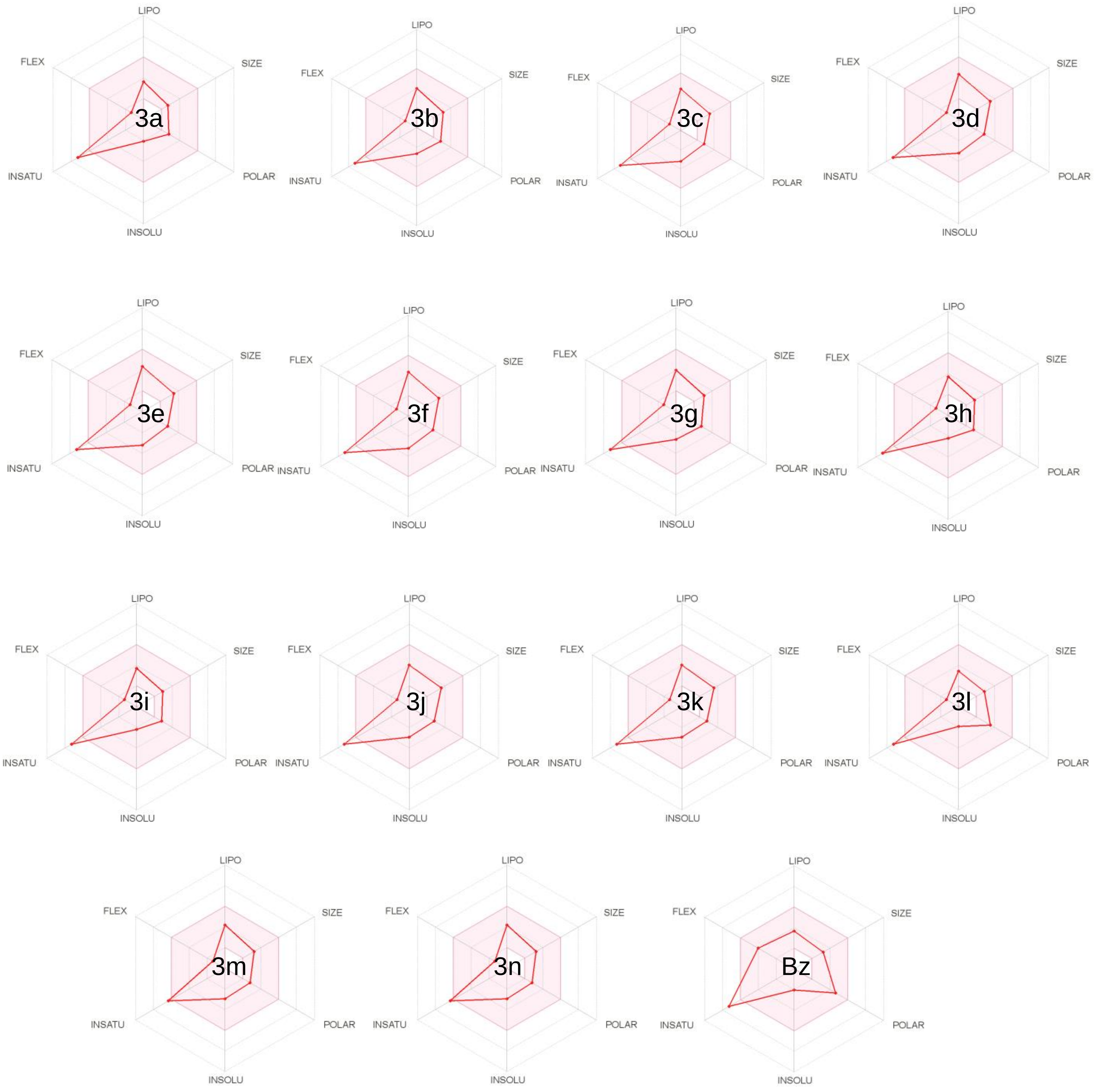

Figure S3. Bioavailability radar chart of pyrazol-imidazoline hybrids (series 3) and Bz. LIPO (lipophilicity) = XLOGP3 between -0.7 and +5.0; SIZE (molecule size) = between 150 and 500 g/mol; POLAR (polarity) = TPSA between 20 and 130 Å2; INSOLU (insolubility)= LOG S <6; INSATU (saturation)= sp3 carbon fraction ≥0.25; FLEX (flexibility) ≤9 rotatable groups.

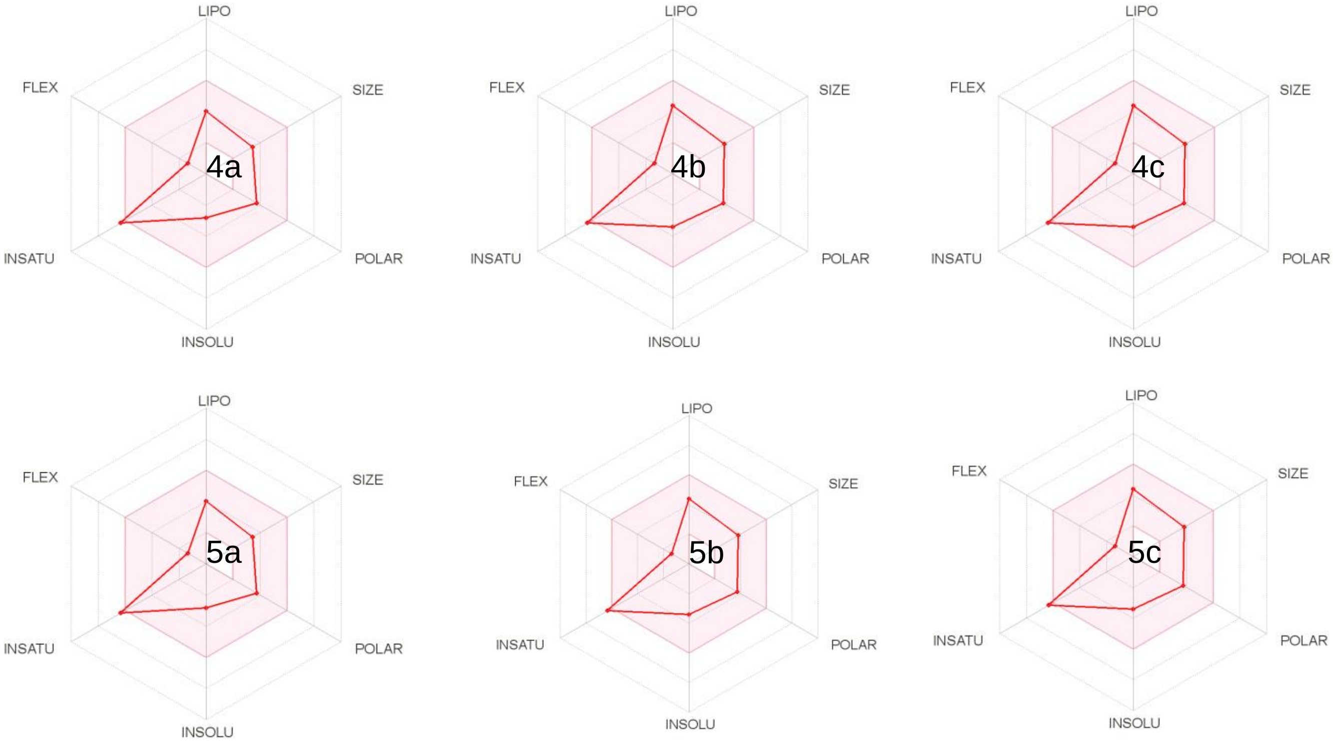

Figure S4. Bioavailability radar chart of pyrazole-imidazoline hybrids (series 4 and 5). LIPO (lipophilicity) = XLOGP3 between -0.7 and +5.0; SIZE (molecule size) = between 150 and 500 g/mol; POLAR (polarity) = TPSA between 20 and 130 Å2; INSOLU (insolubility)= LOG S <6; INSATU (saturation)= sp3 carbon fraction ≥0.25; FLEX (flexibility) ≤9 rotatable groups.
