# Supplementary material for: Structural Optimization and Biological Activity of Pyrazole Derivatives: Virtual Computational Analysis, Recovery Assay and 3D Culture Model as Potential Predictive Tools of Effectiveness against Trypanosoma cruzi
Source: Molecules. 2021 Nov 8;26(21):6742. doi: 10.3390/molecules26216742 (PMC8587750; doi:10.3390/molecules26216742)

^{1}H NMR of compound **1a**

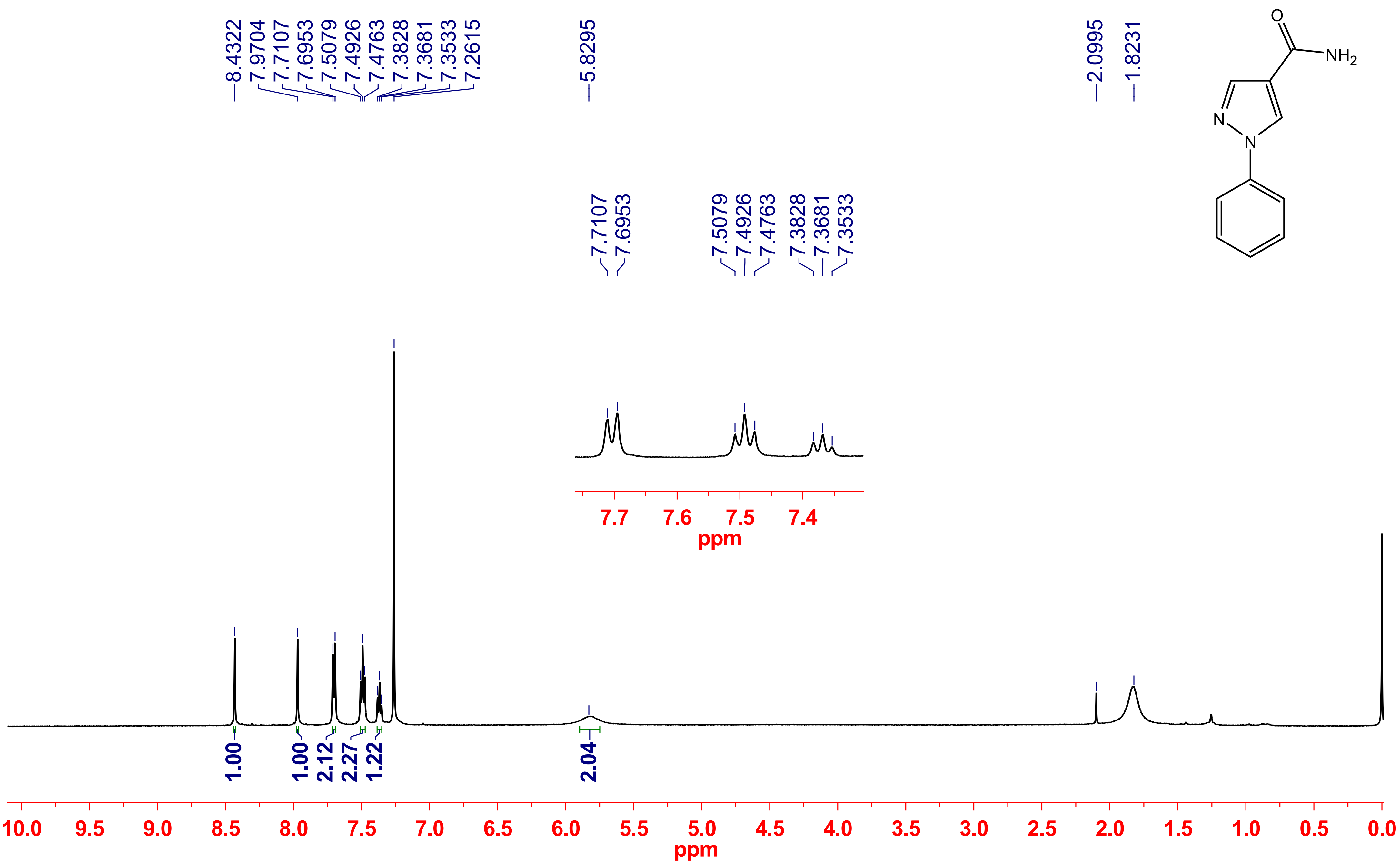

^{13}C NMR of compound **1a**

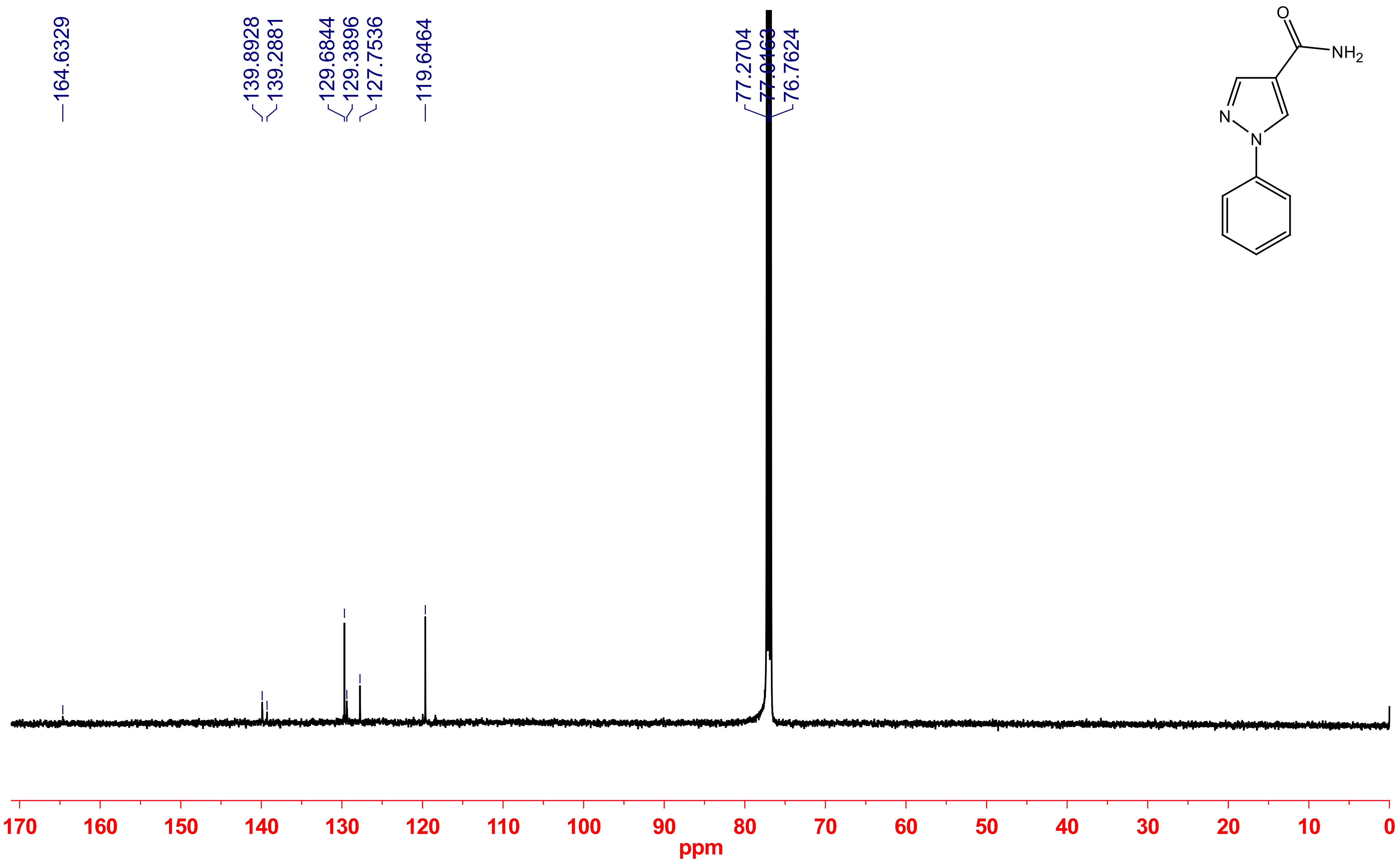

^{1}H NMR of compound **1b**

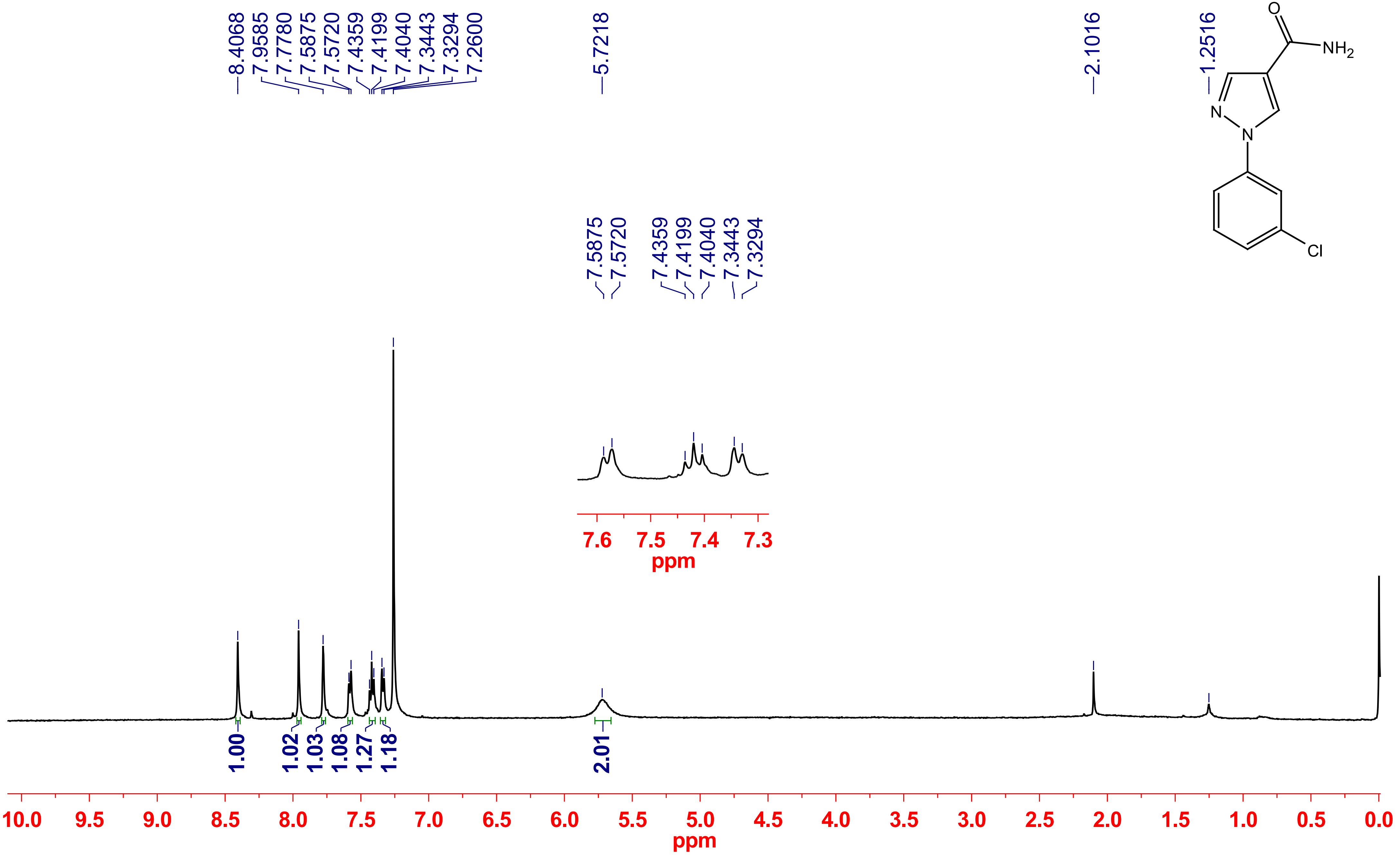

^{13}C NMR of compound **1b**

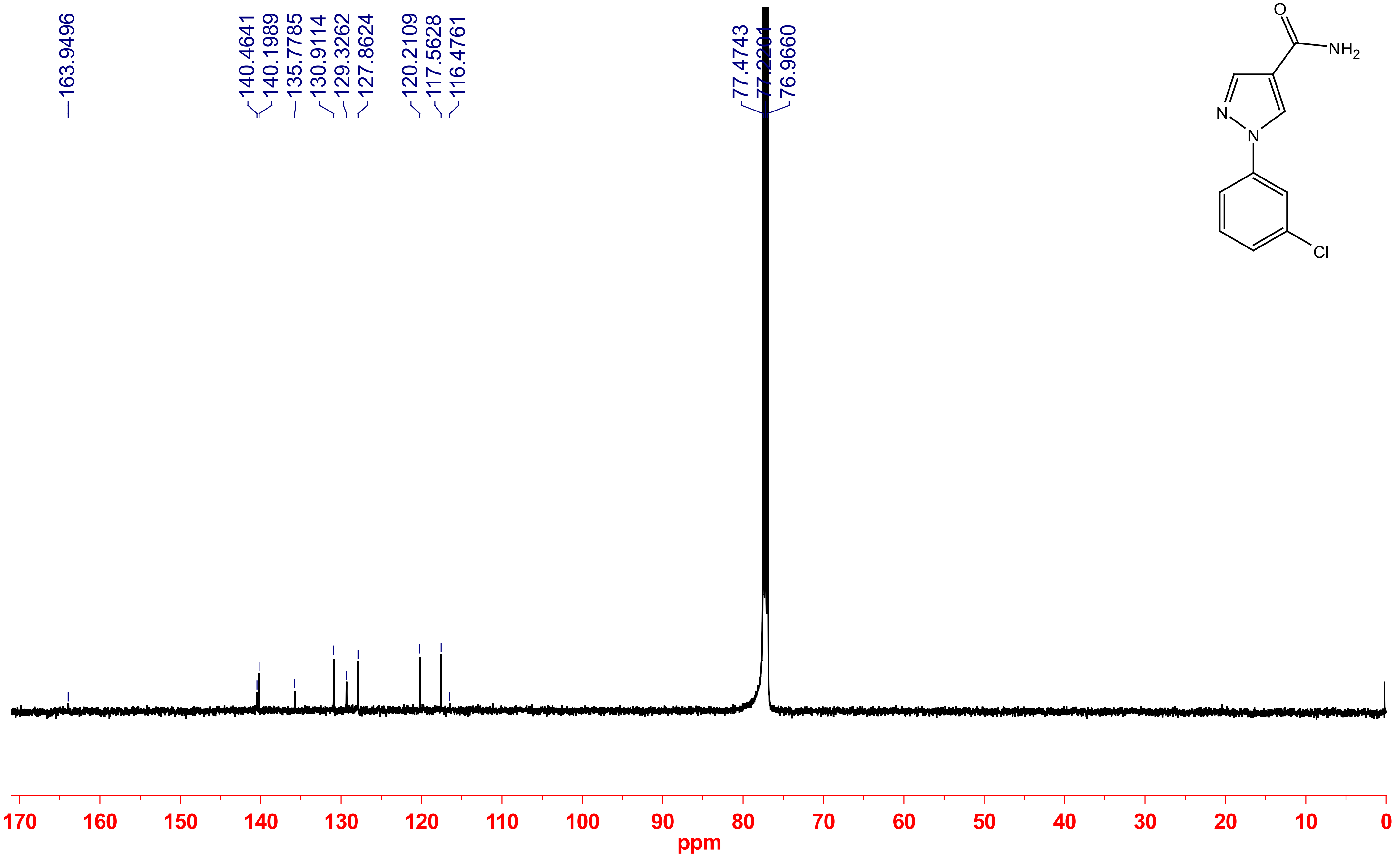

^{1}H NMR of compound **1c**

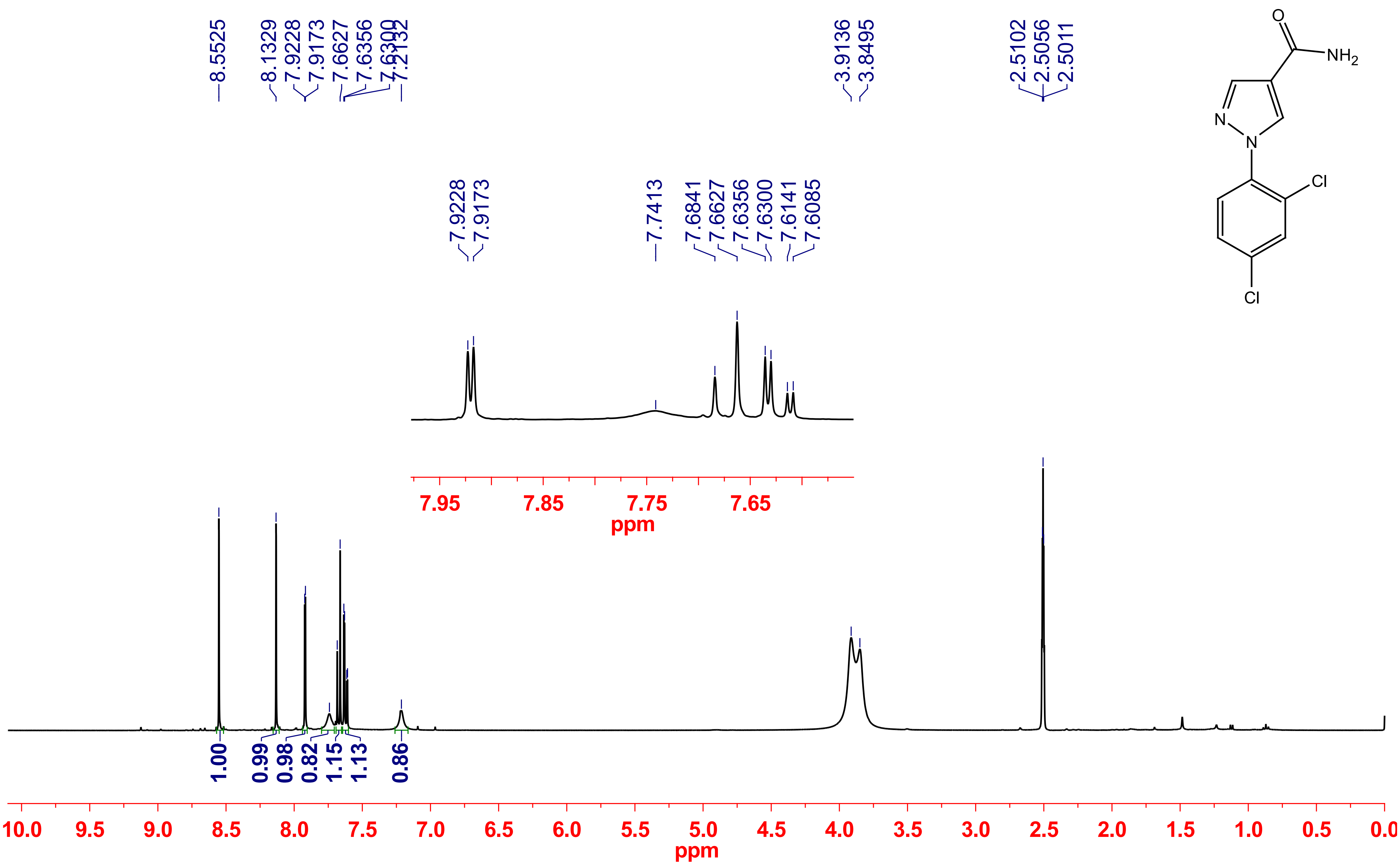

^{13}C NMR of compound **1c**

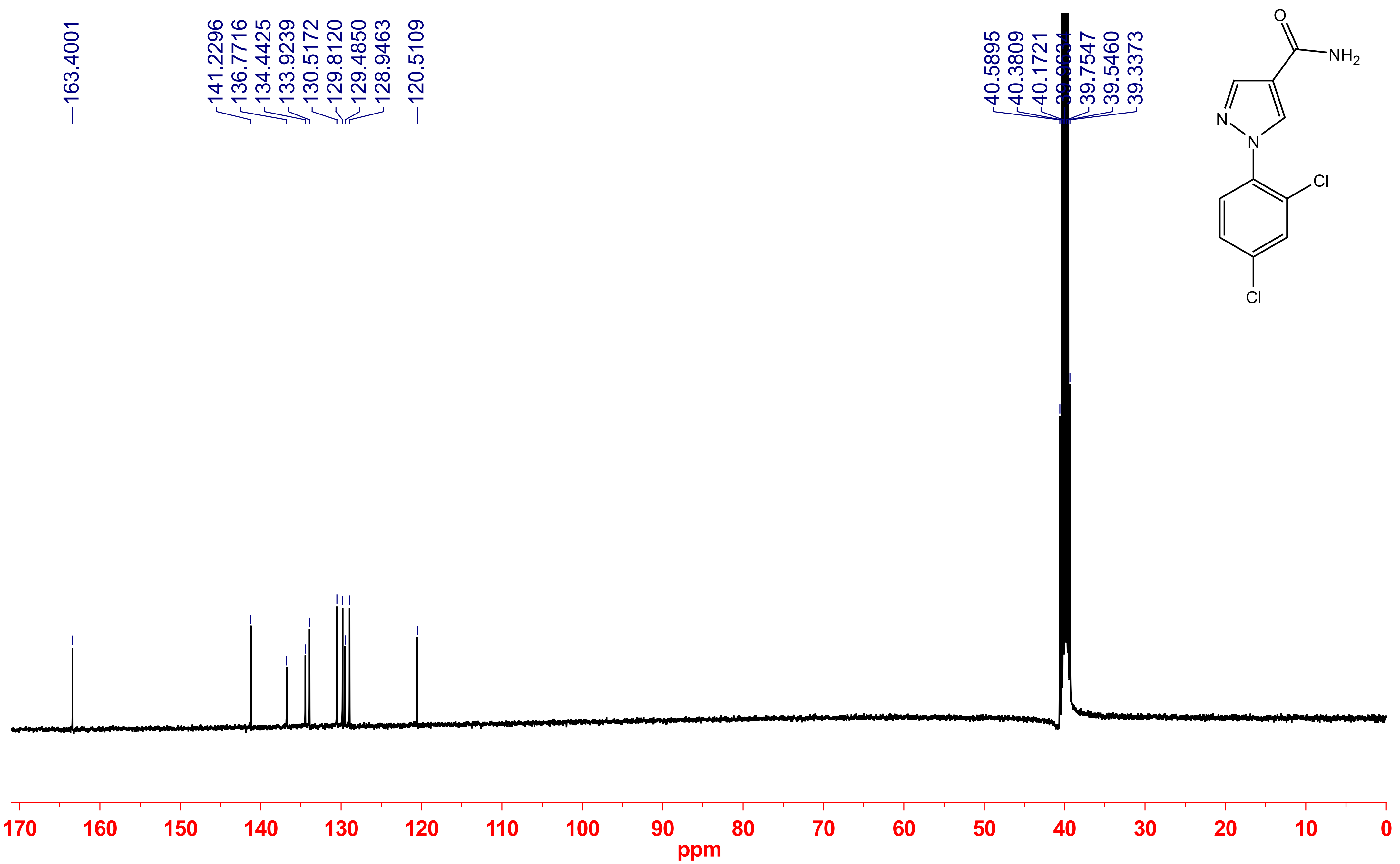

^{1}H NMR of compound **1d**

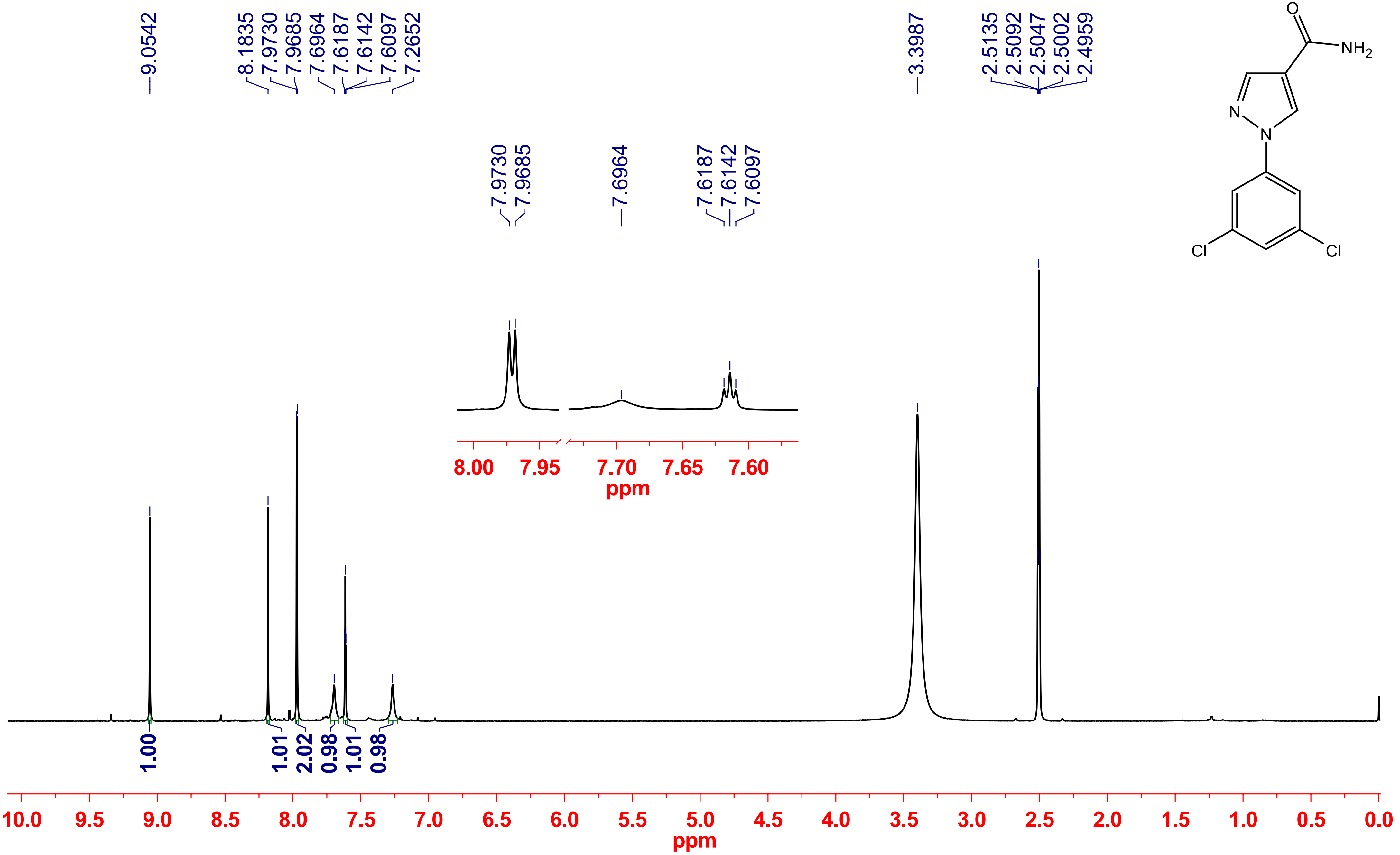

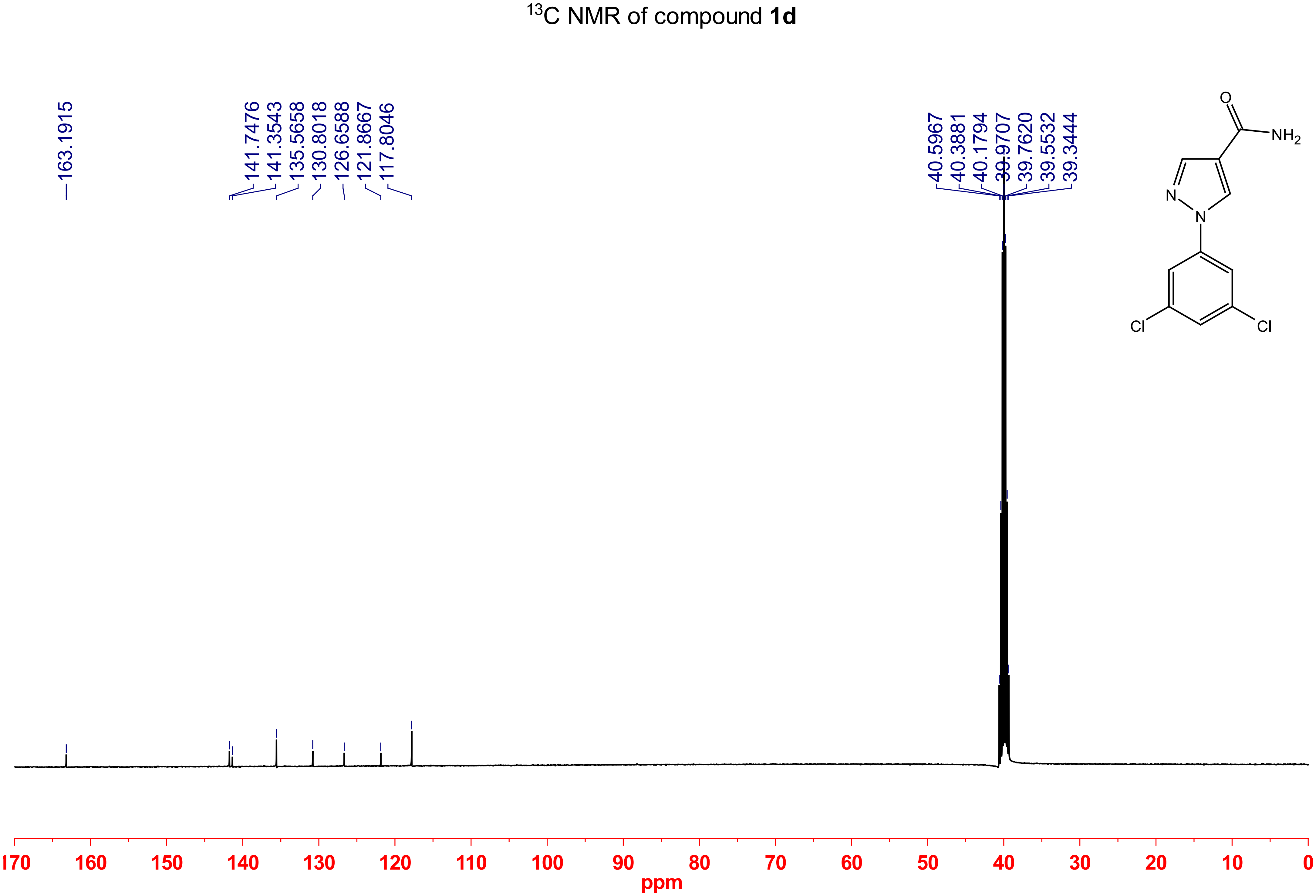

13C NMR of compound 1d
163.1915
141.7476
141.3543
135.5658
130.8018
126.6588
121.8667
117.8046
40.5967
40.3881
40.1794
39.9707
39.7620
39.5532
39.3444
O
NH2
N
N
Cl
Cl
170
160
150
140
130
120
110
100
90
80
70
60
50
40
30
20
10
0
ppm

^{1}H NMR of compound **1e**

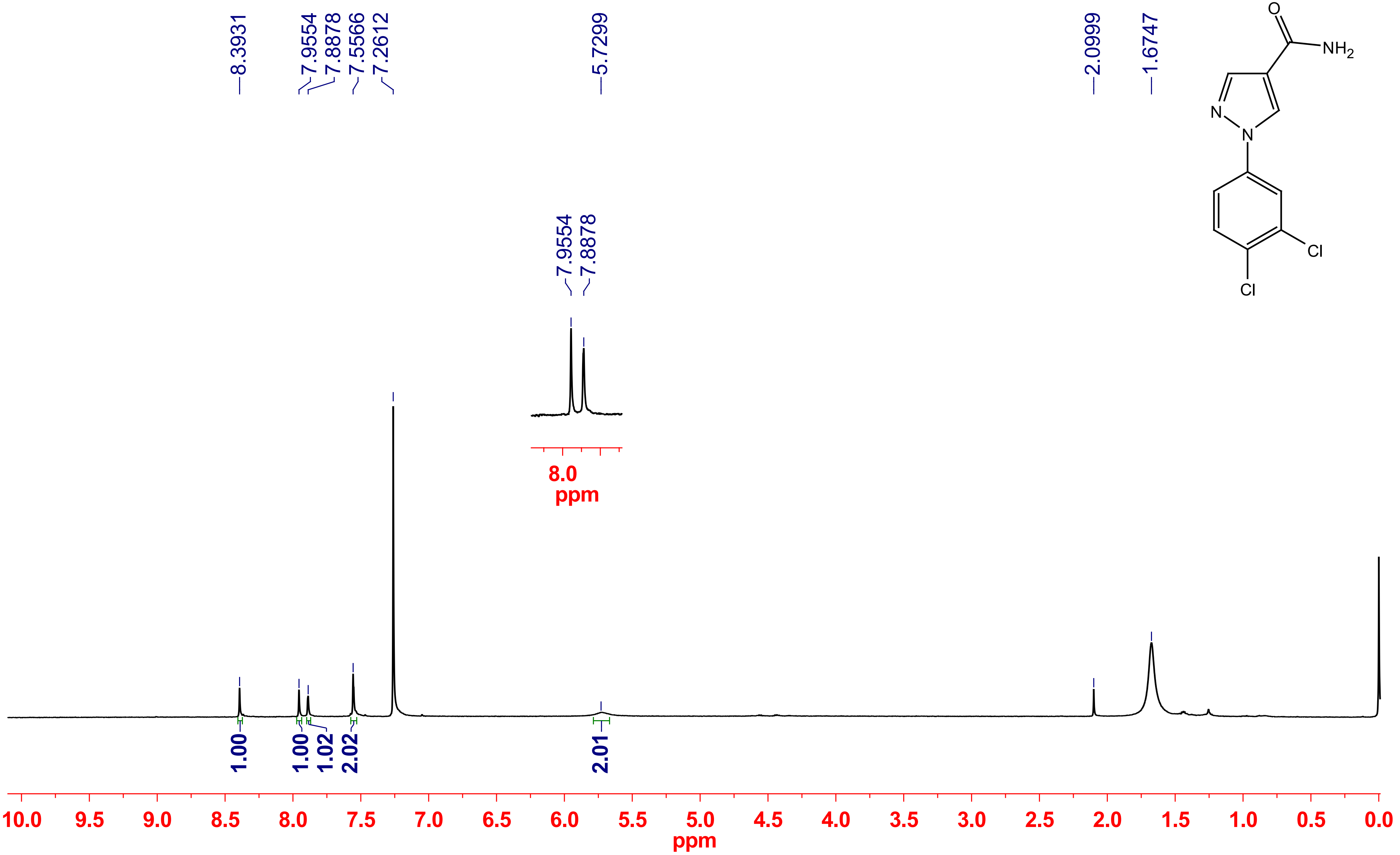

^{13}C NMR of compound **1e**

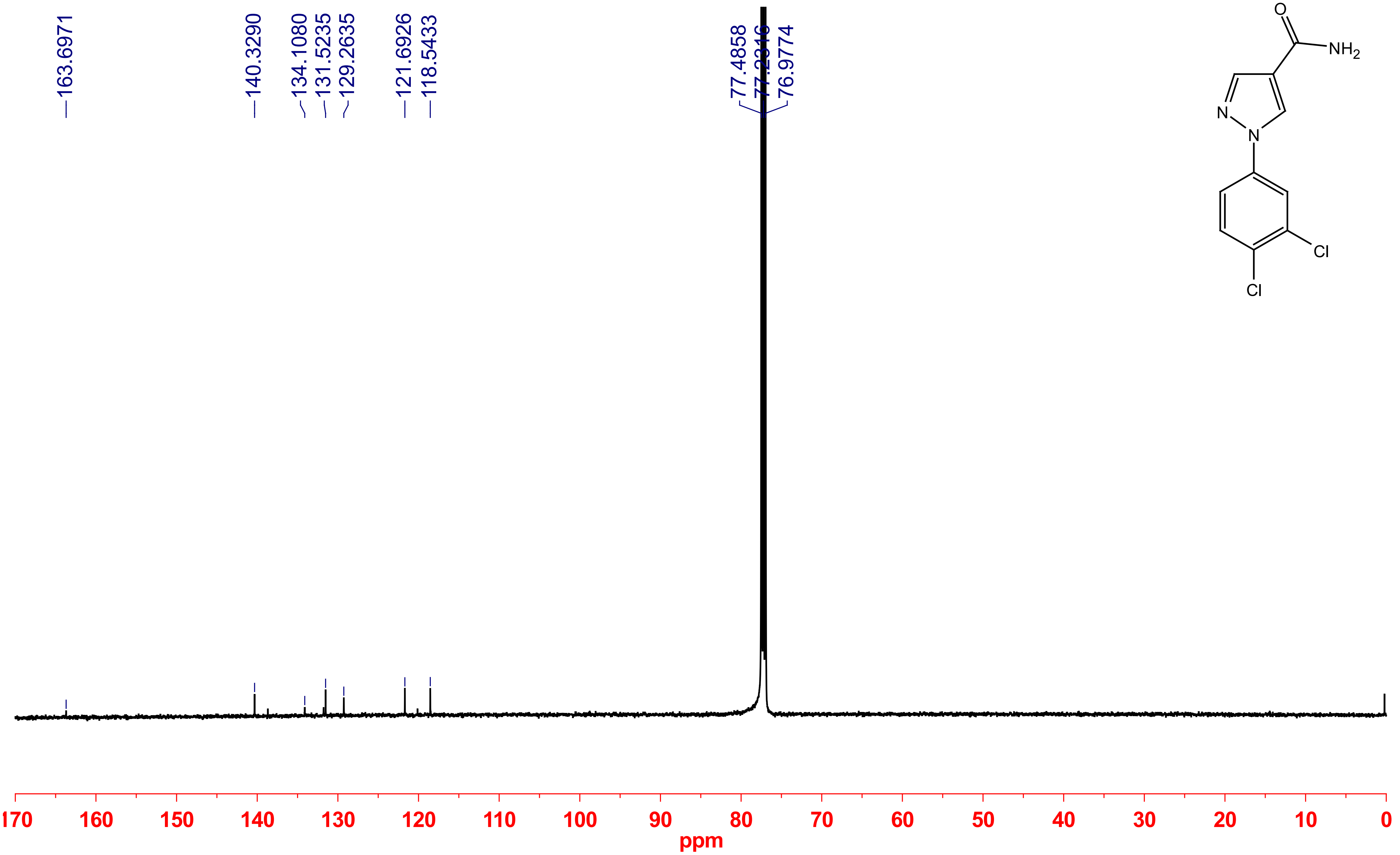

^{1}H NMR of compound **1f**

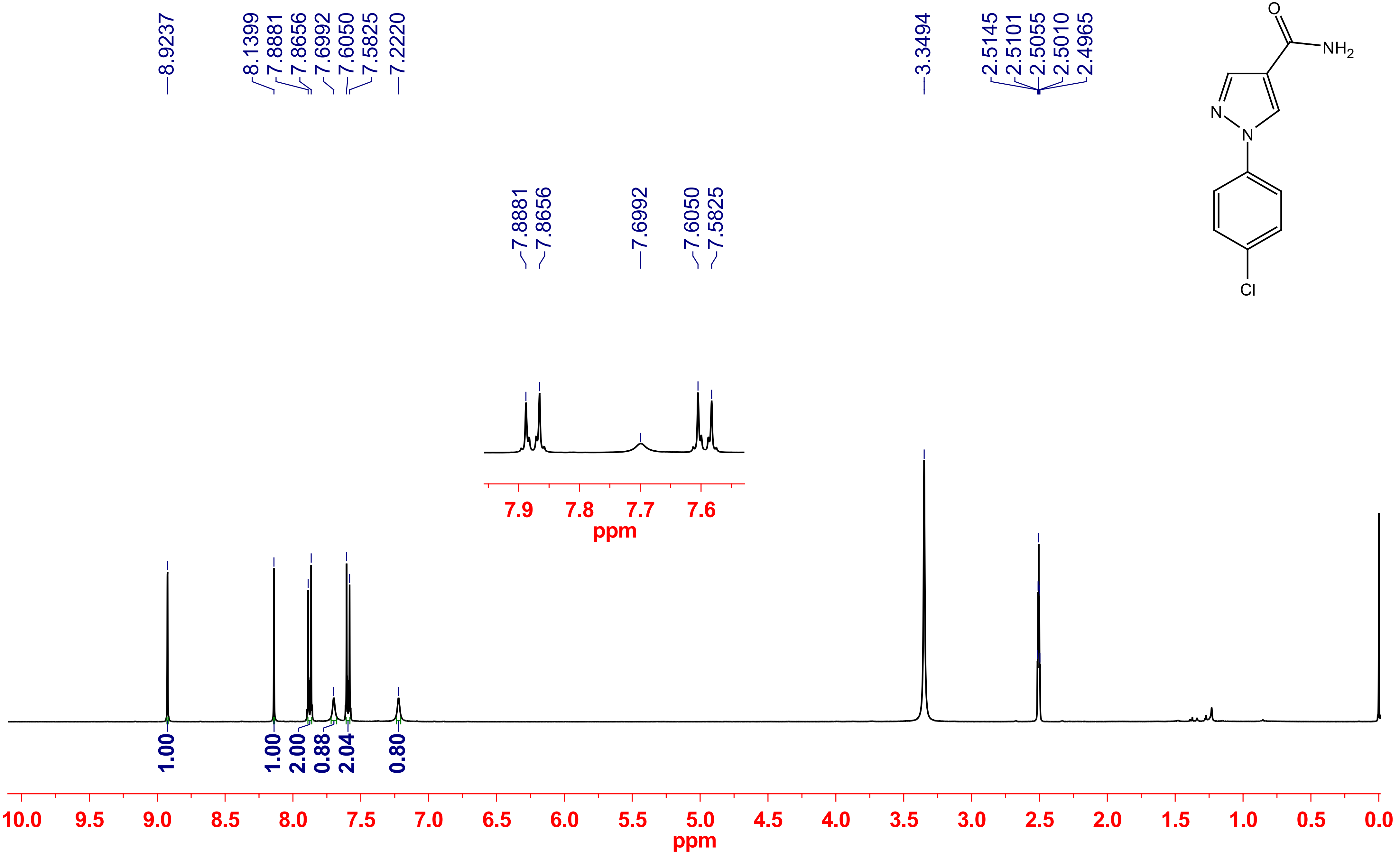

^{13}C NMR of compound **1f**

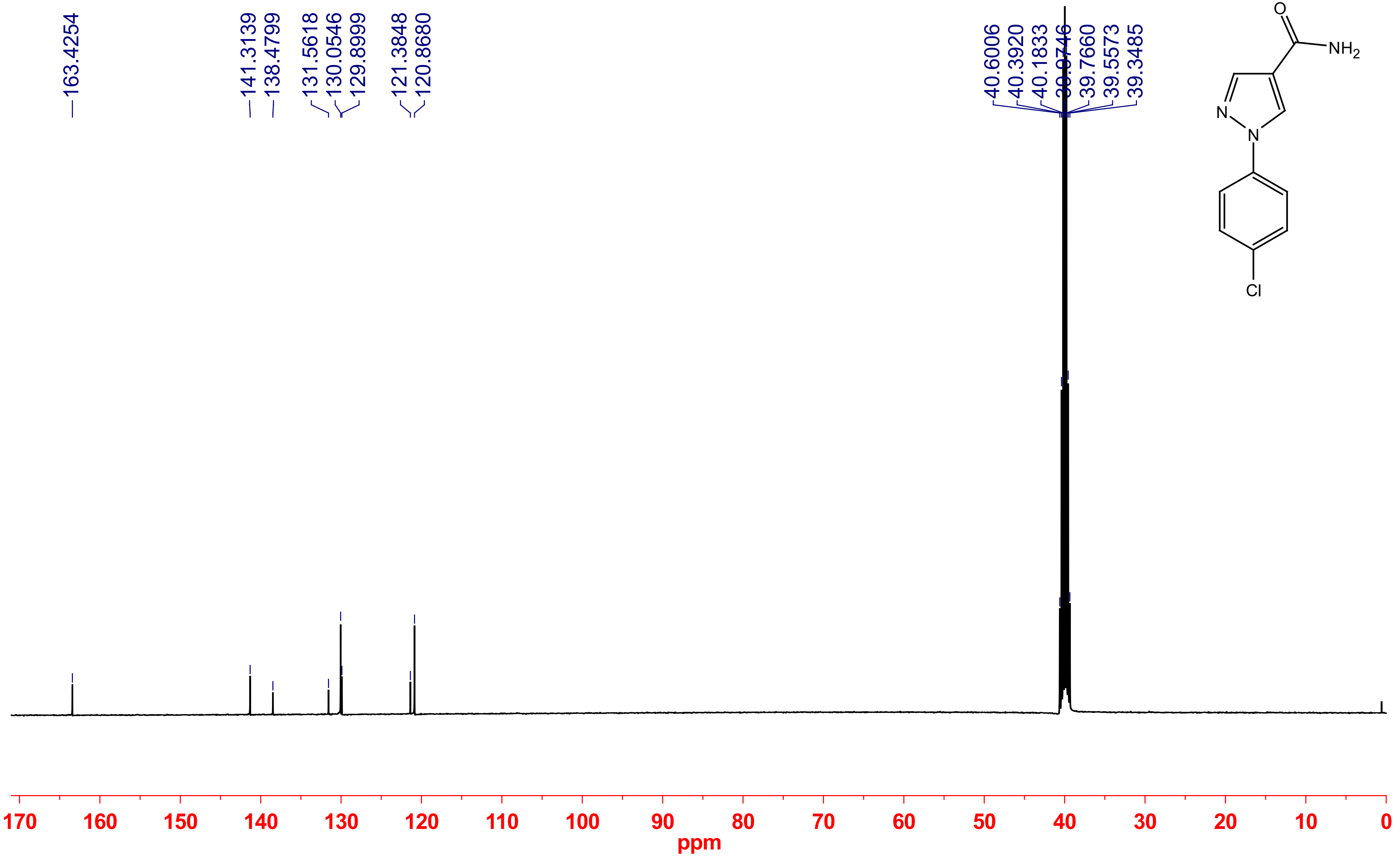

^{1}H NMR of compound **1g**

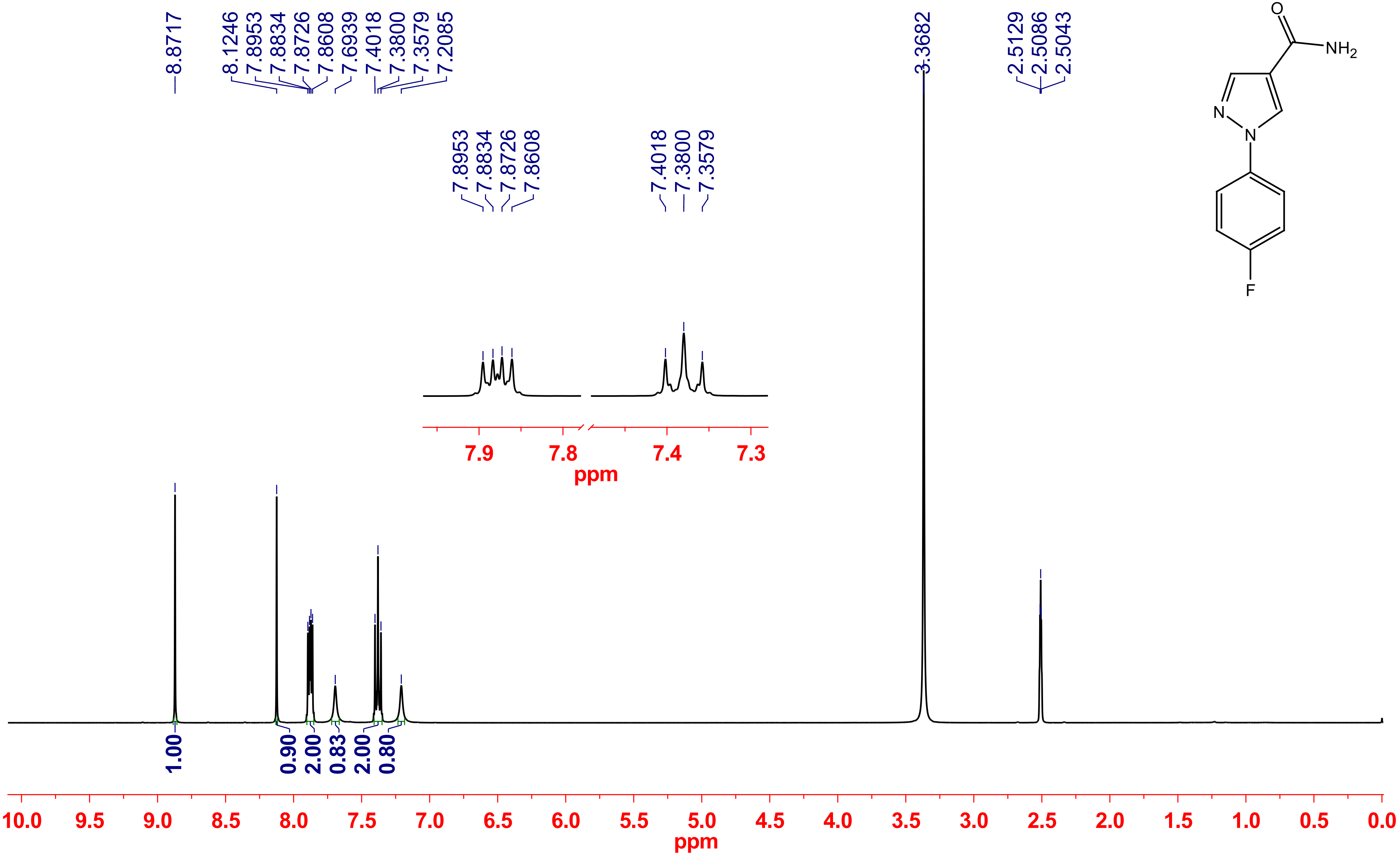

^{13}C NMR of compound **1g**

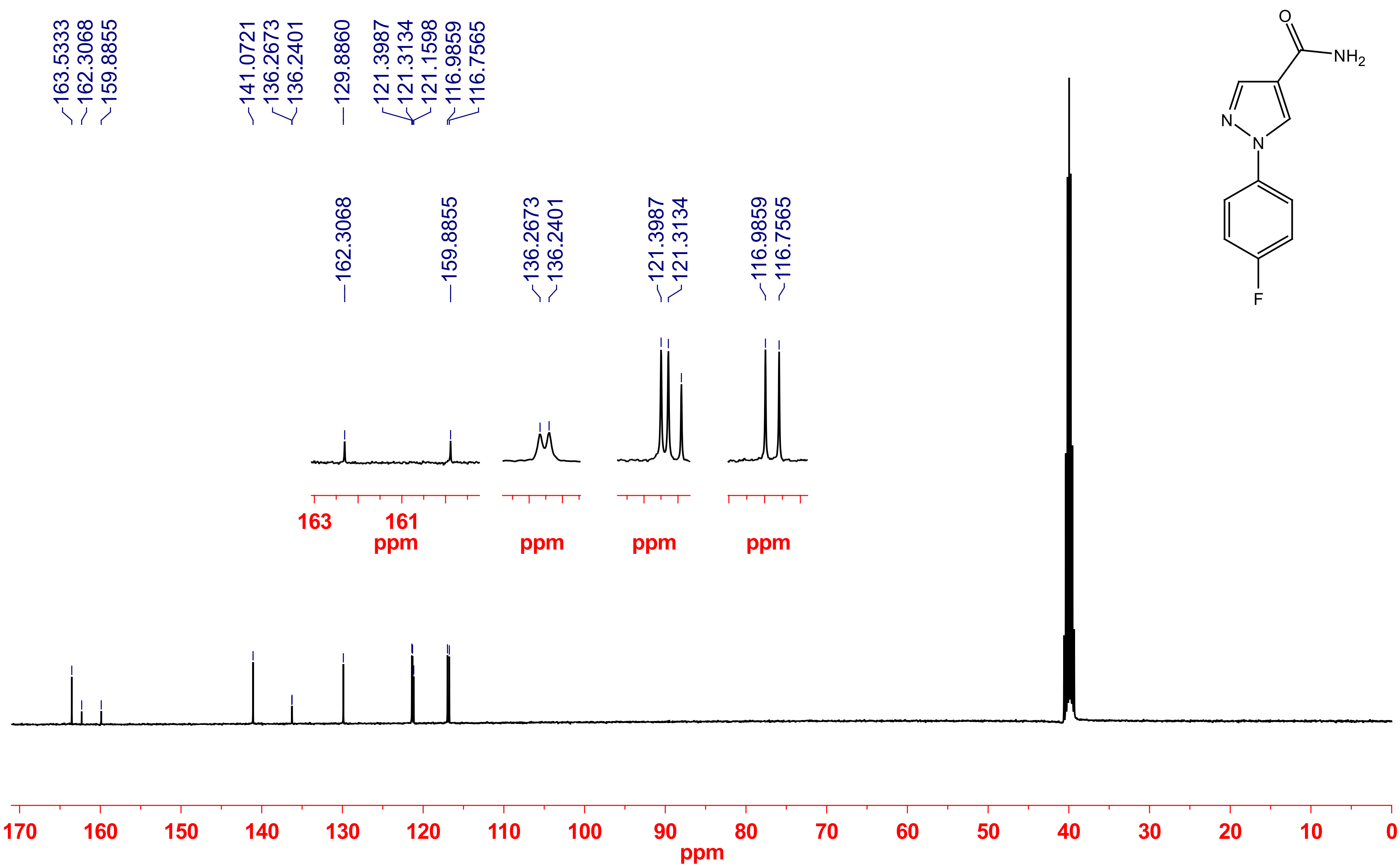

^{1}H NMR of compound **1h**

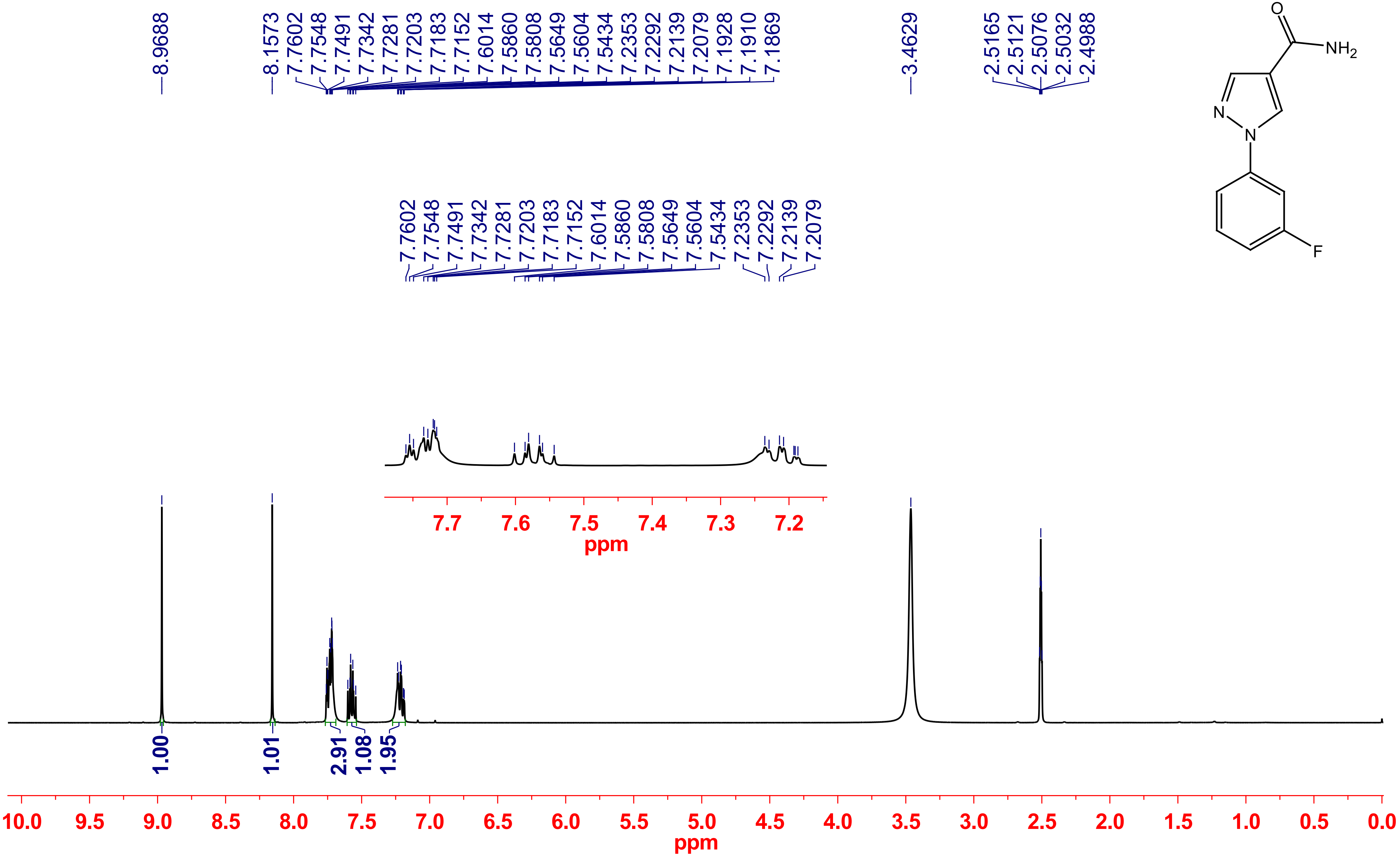

^{13}C NMR of compound **1h**

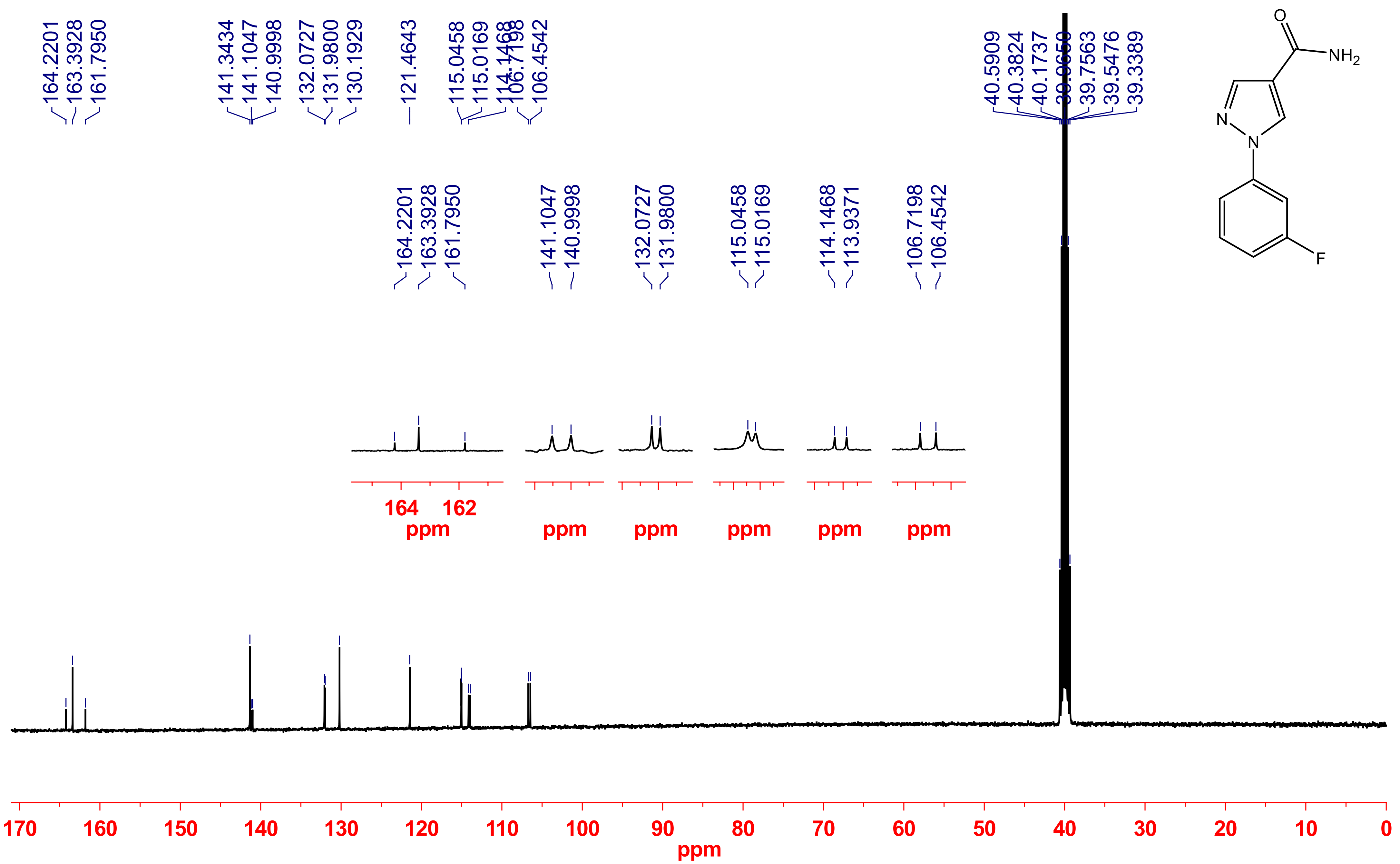

^{1}H NMR of compound **1i**

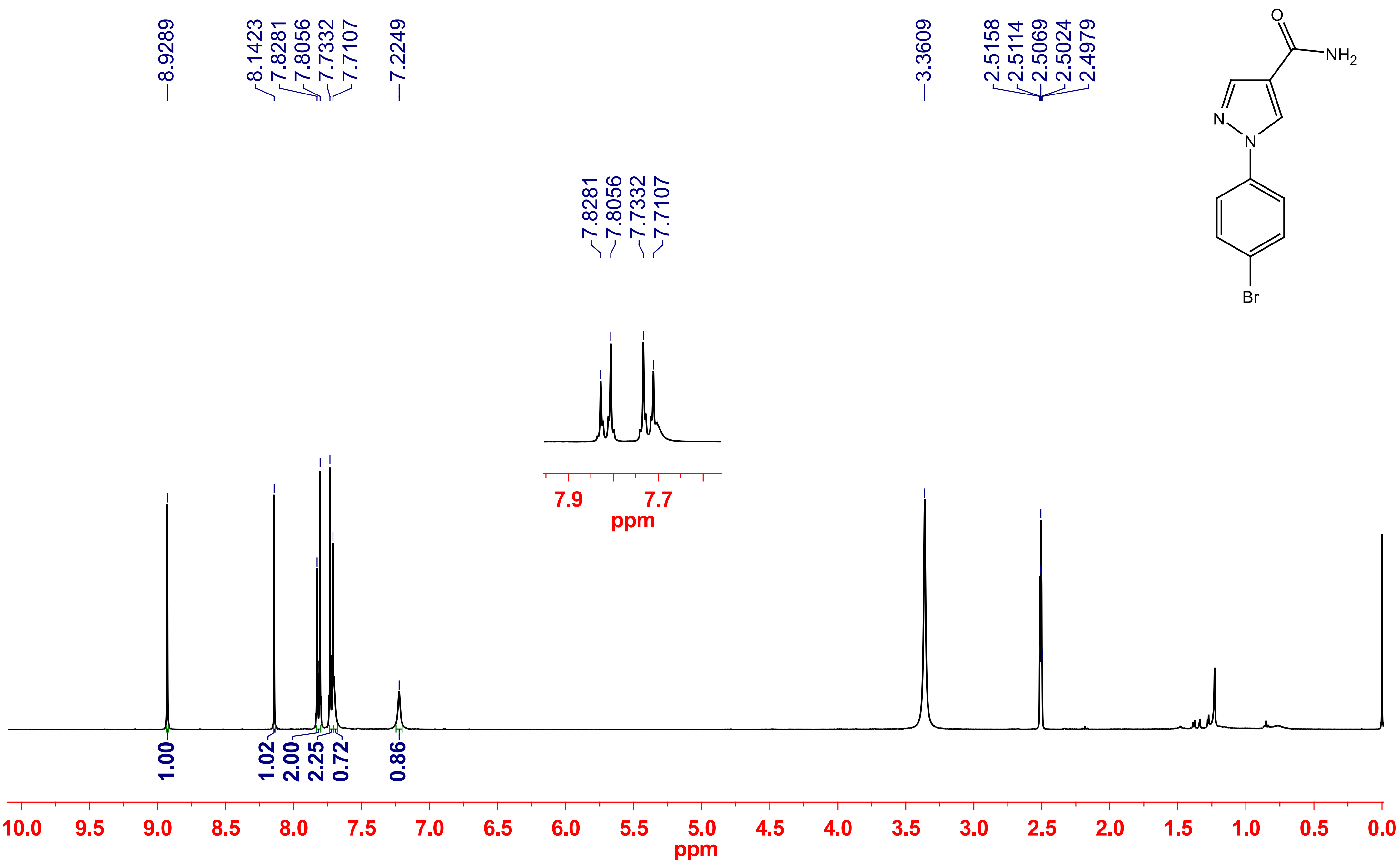

^{13}C NMR of compound **1i**

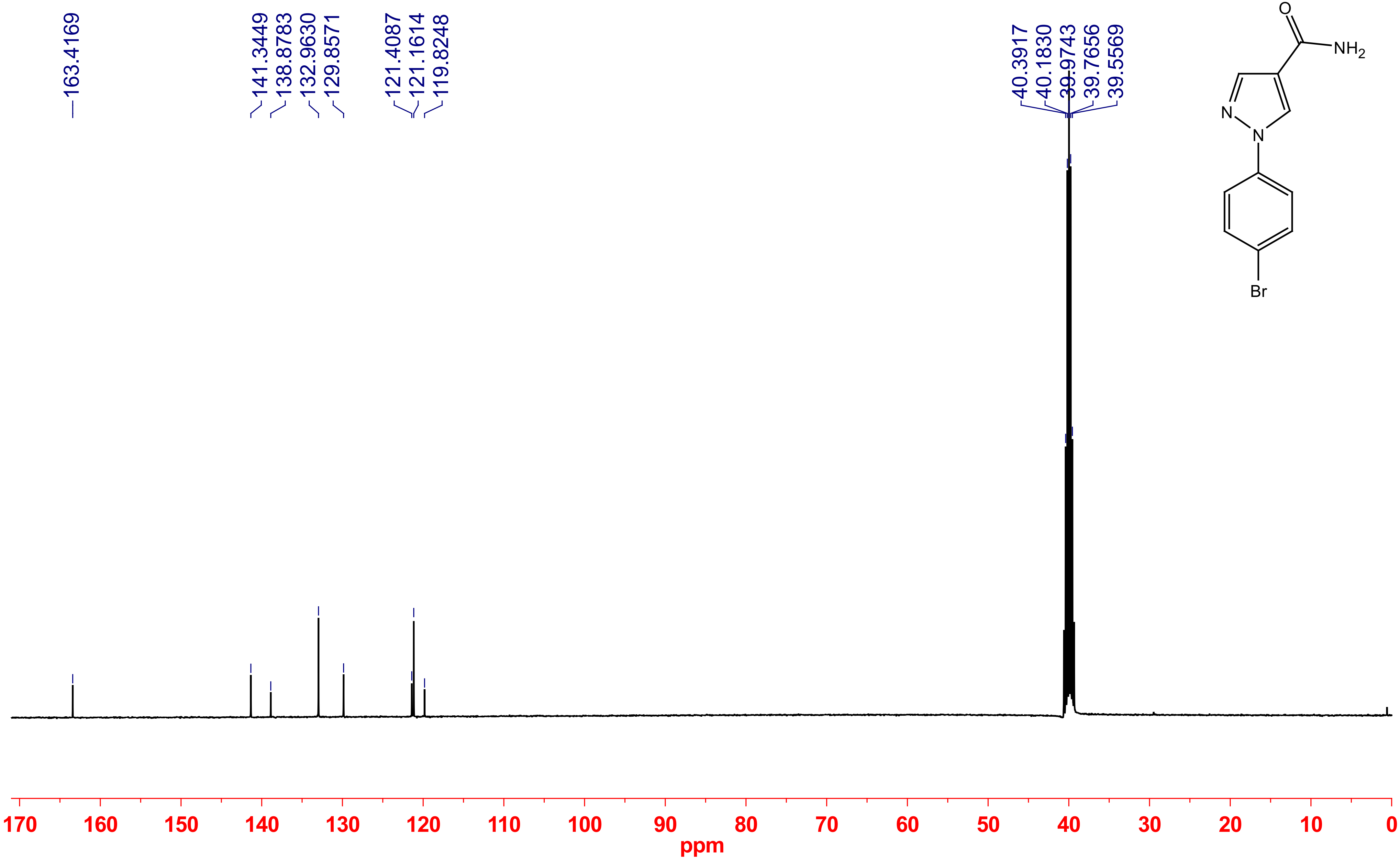

^{1}H NMR of compound **1j**

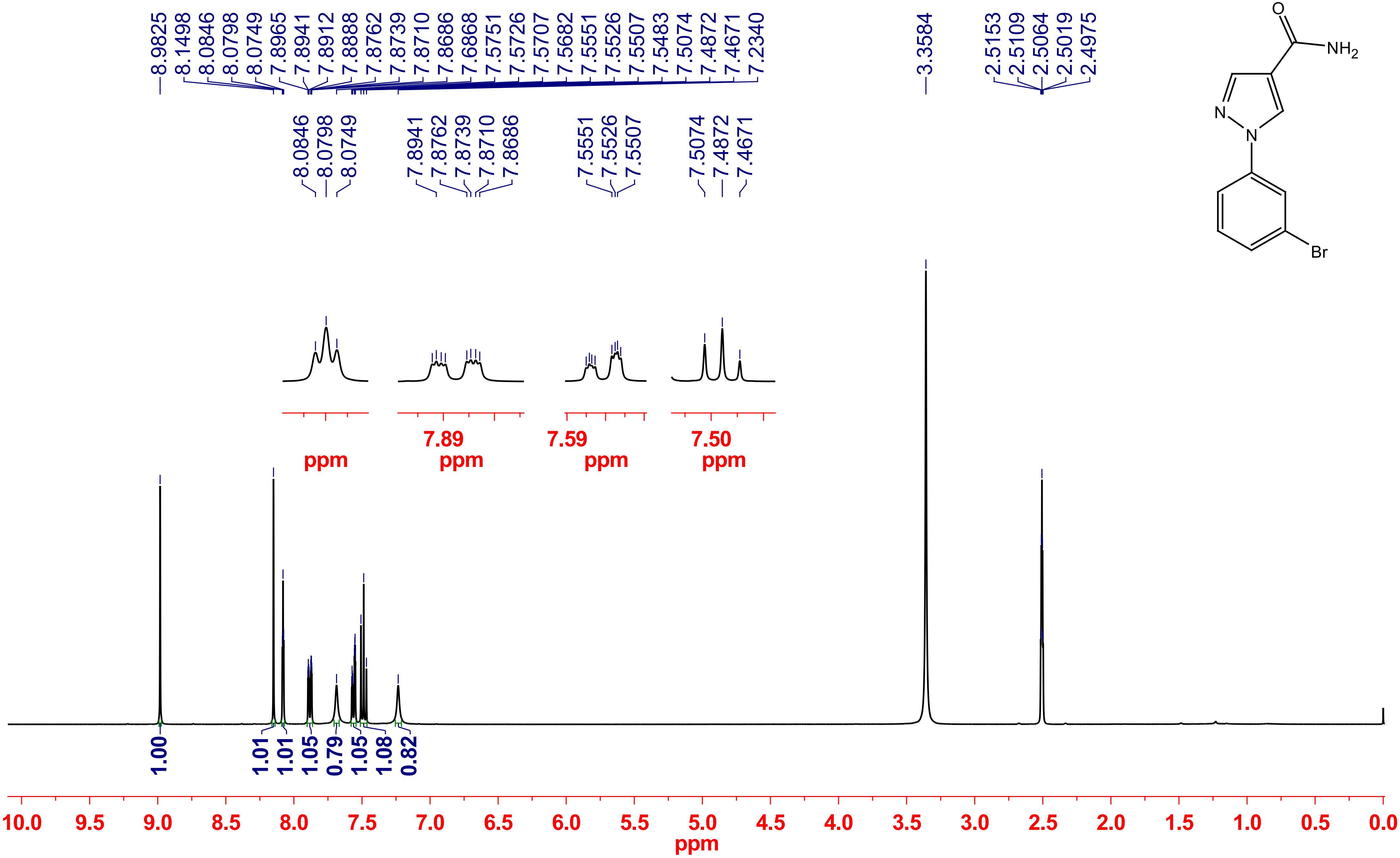

^{13}C NMR of compound **1j**

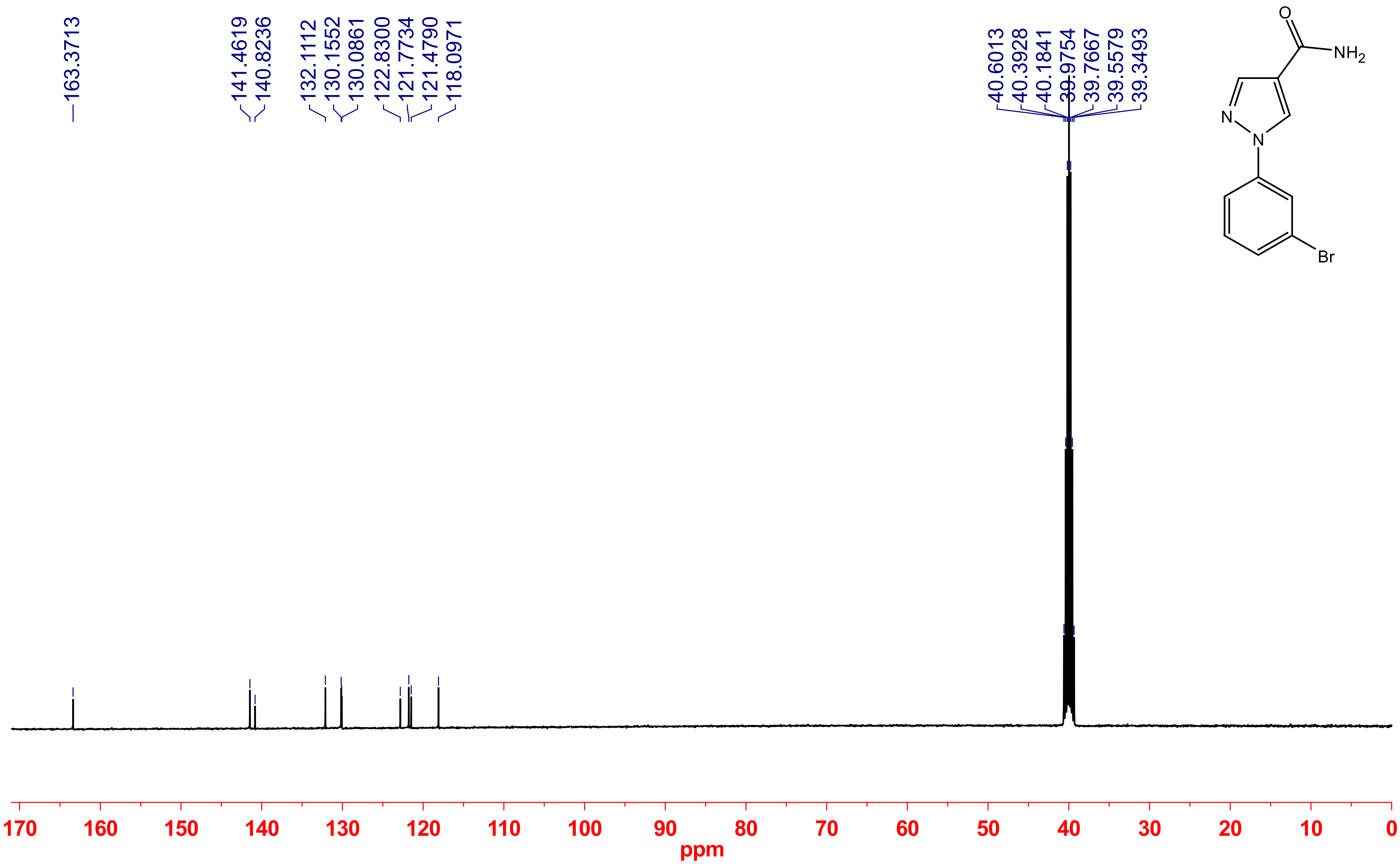

^{1}H NMR of compound **1k**

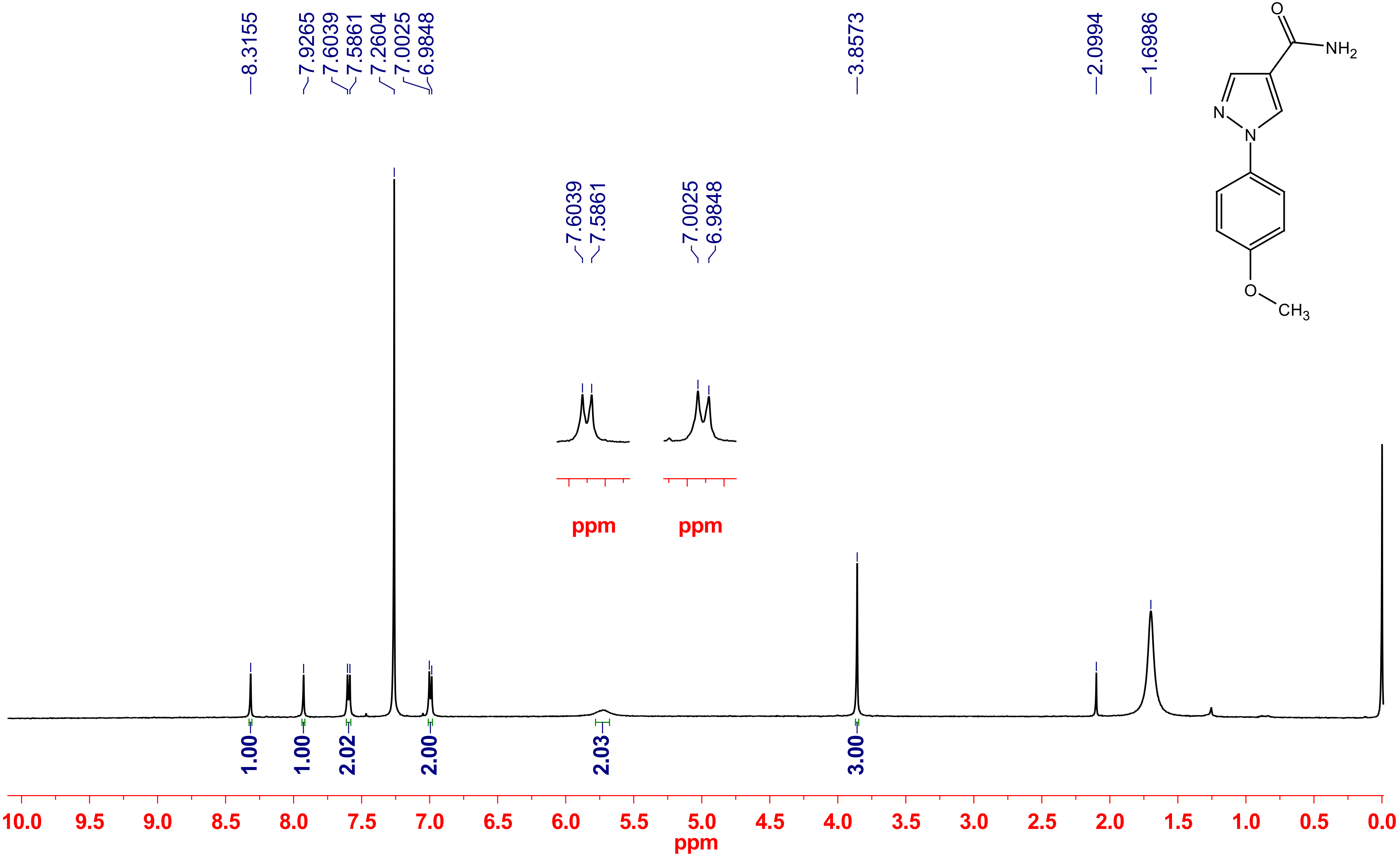

^{13}C NMR of compound **1k**

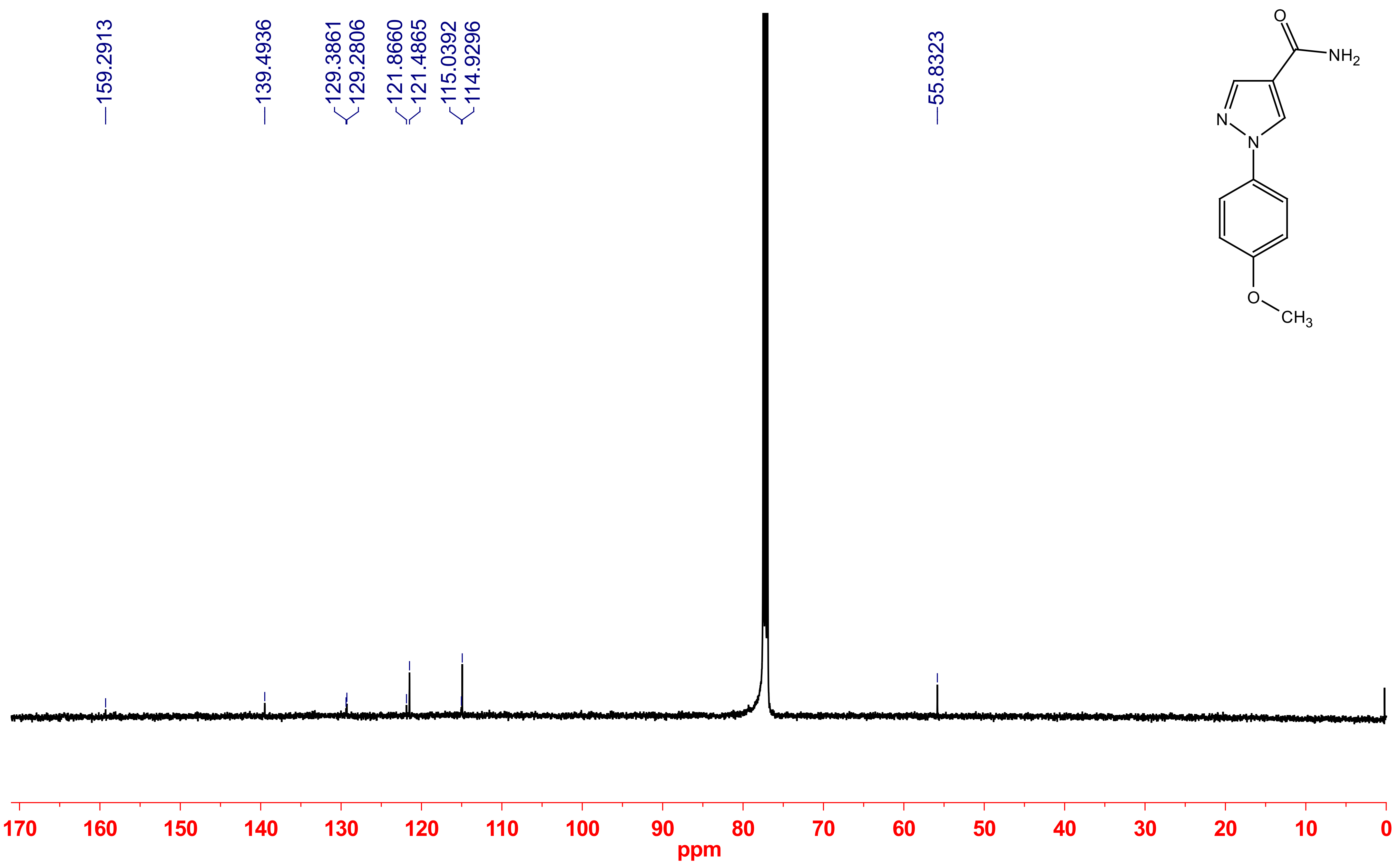

^{1}H NMR of compound **1l**

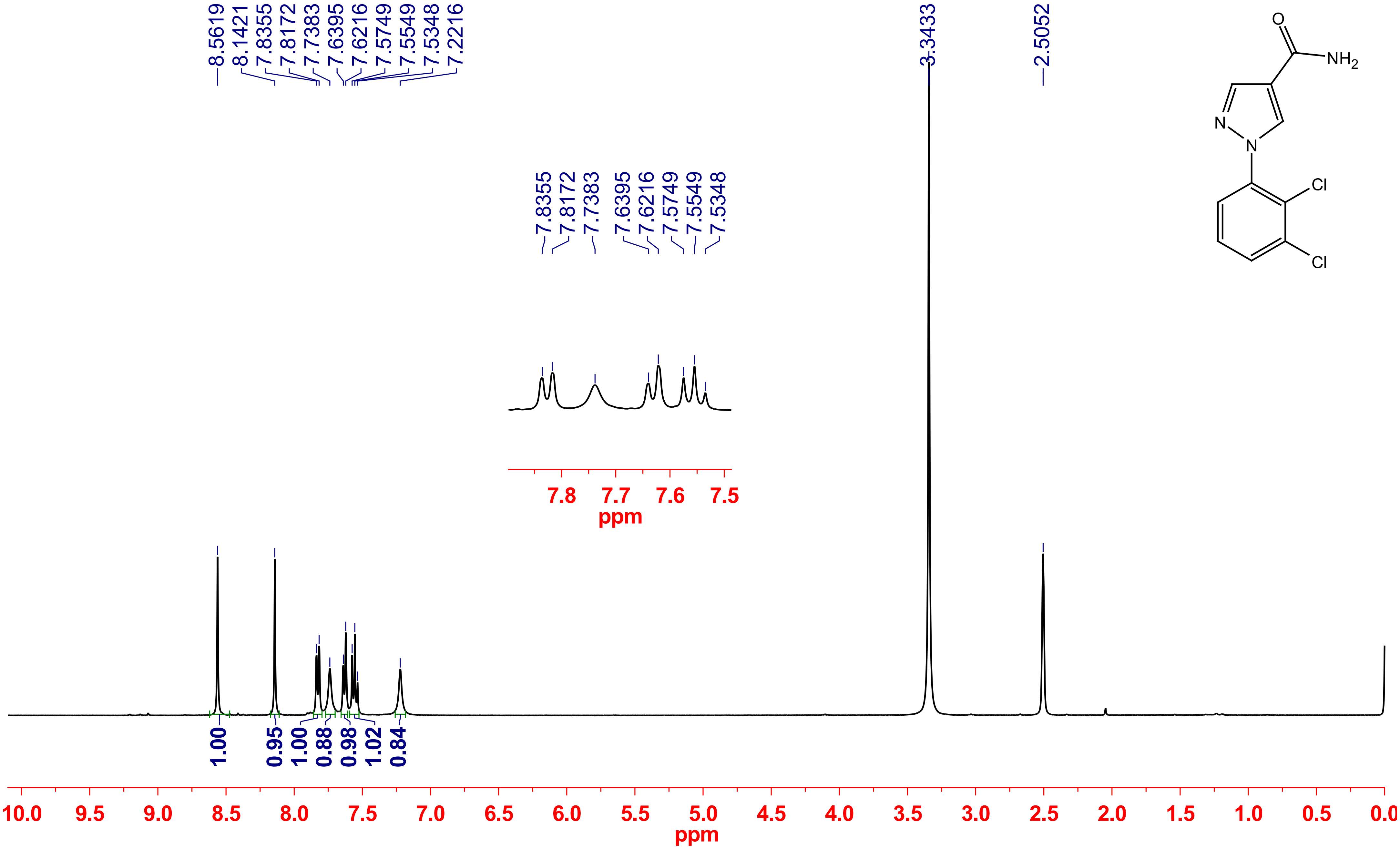

^{13}C NMR of compound **1l**

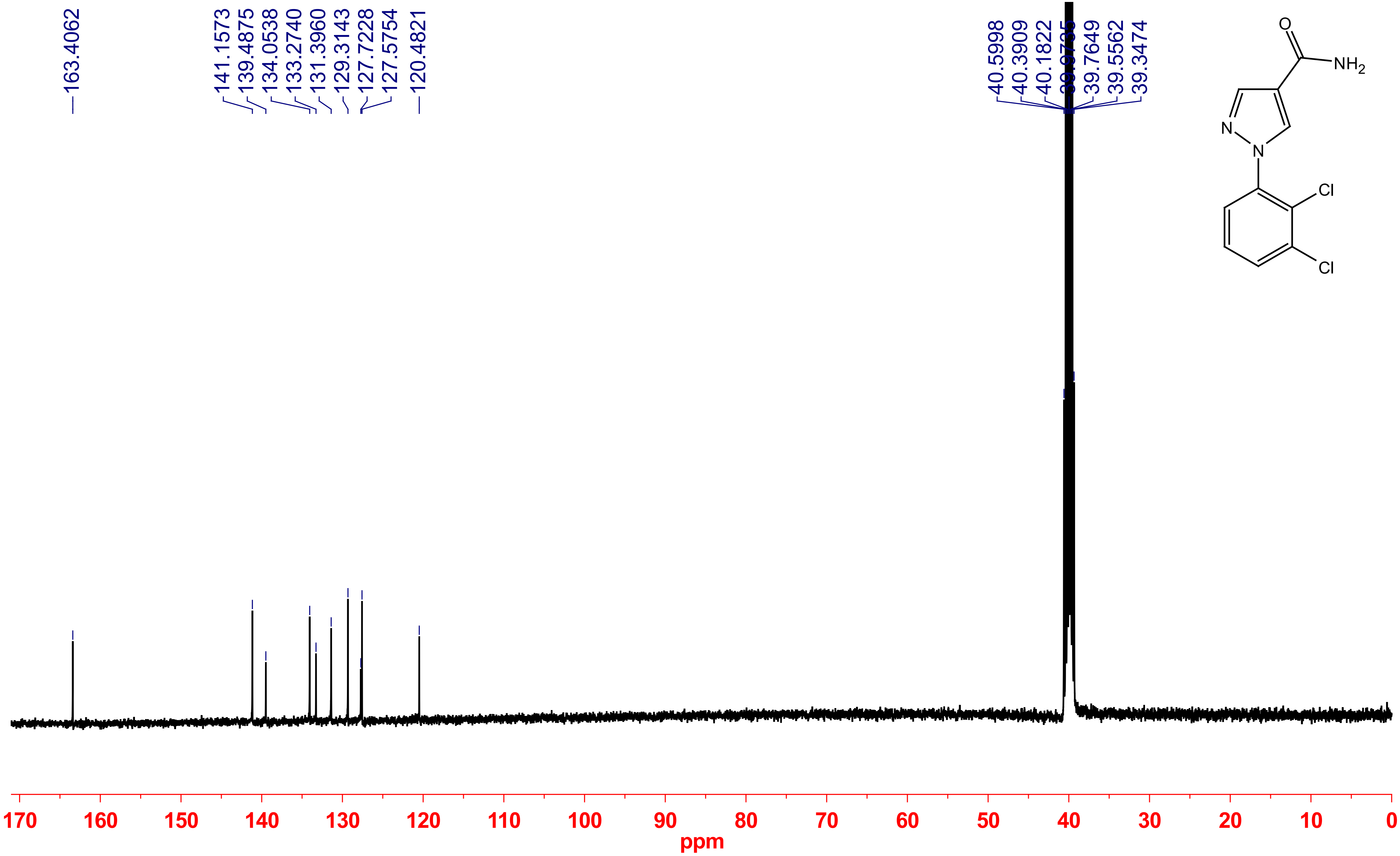

^{1}H NMR of compound **2a**

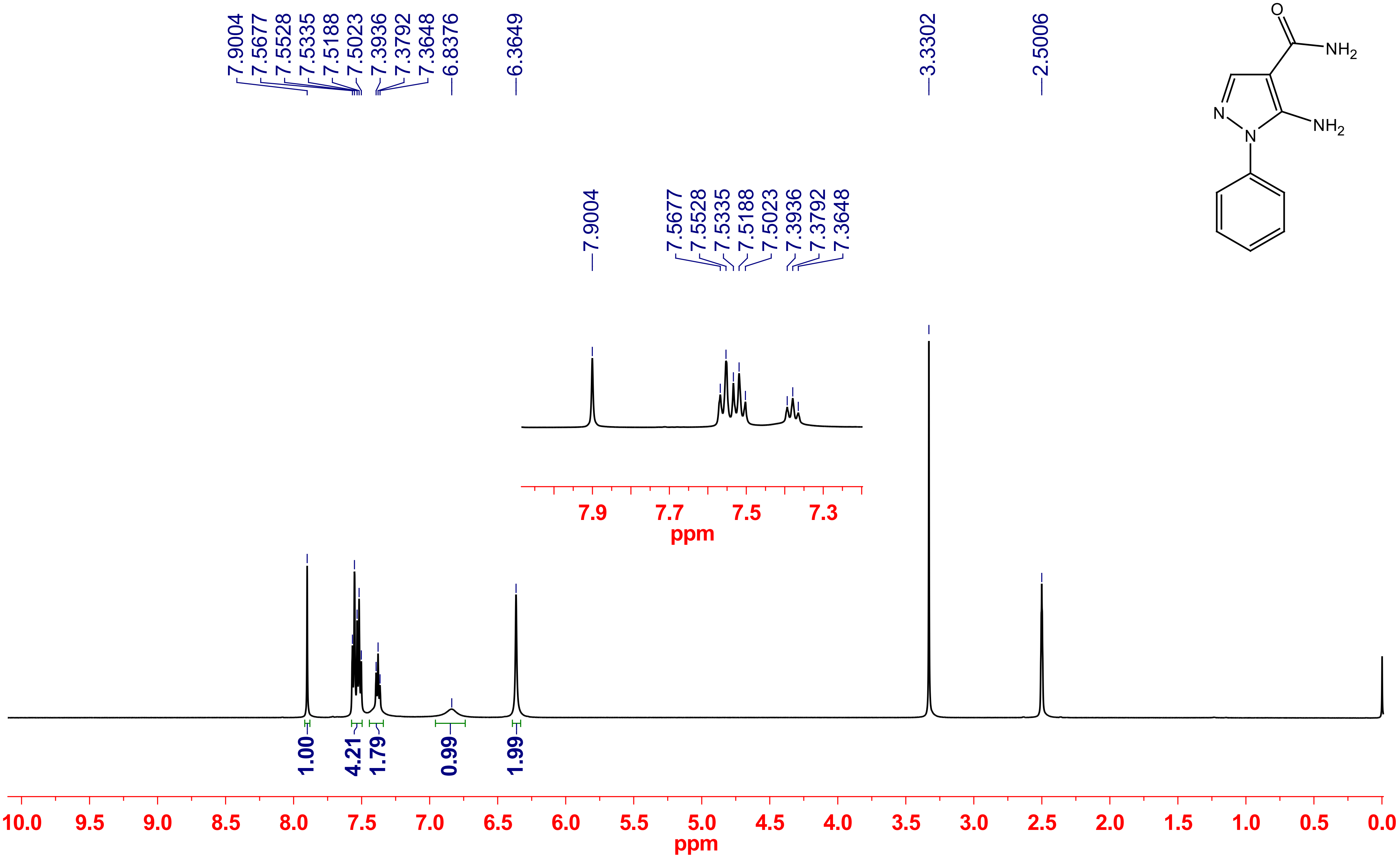

^{13}C NMR of compound **2a**

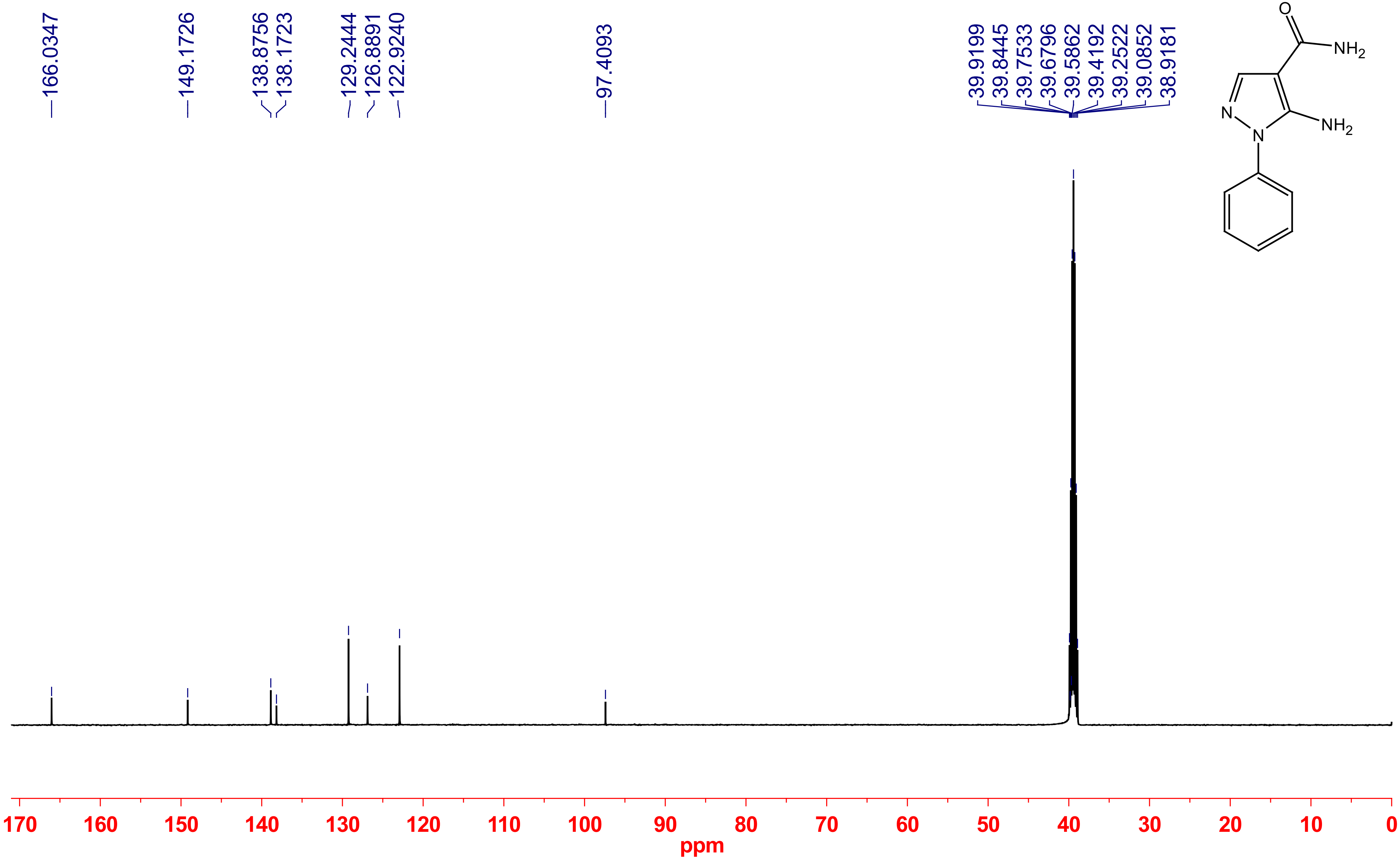

^{1}H NMR of compound **2b**

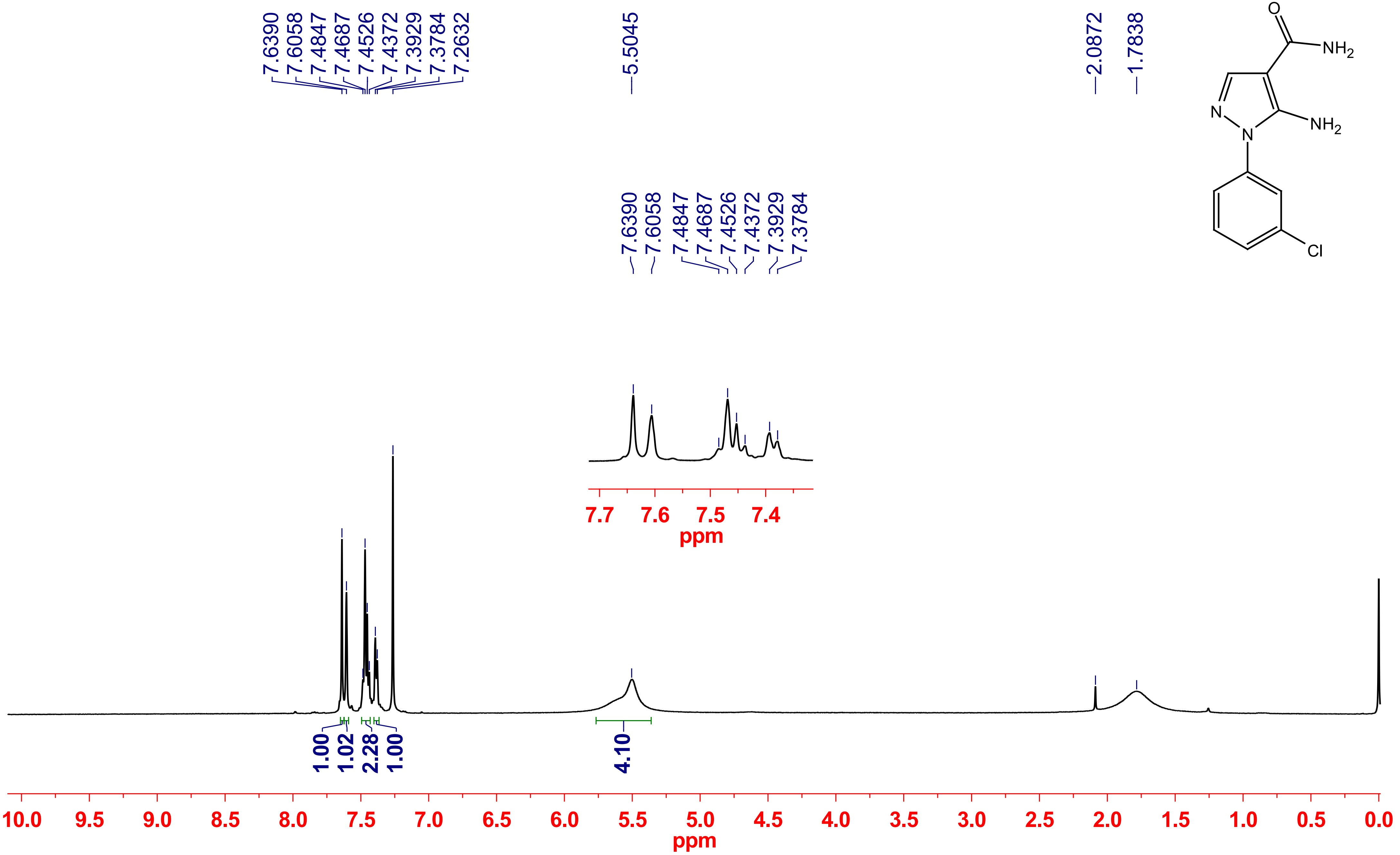

^{13}C NMR of compound **2b**

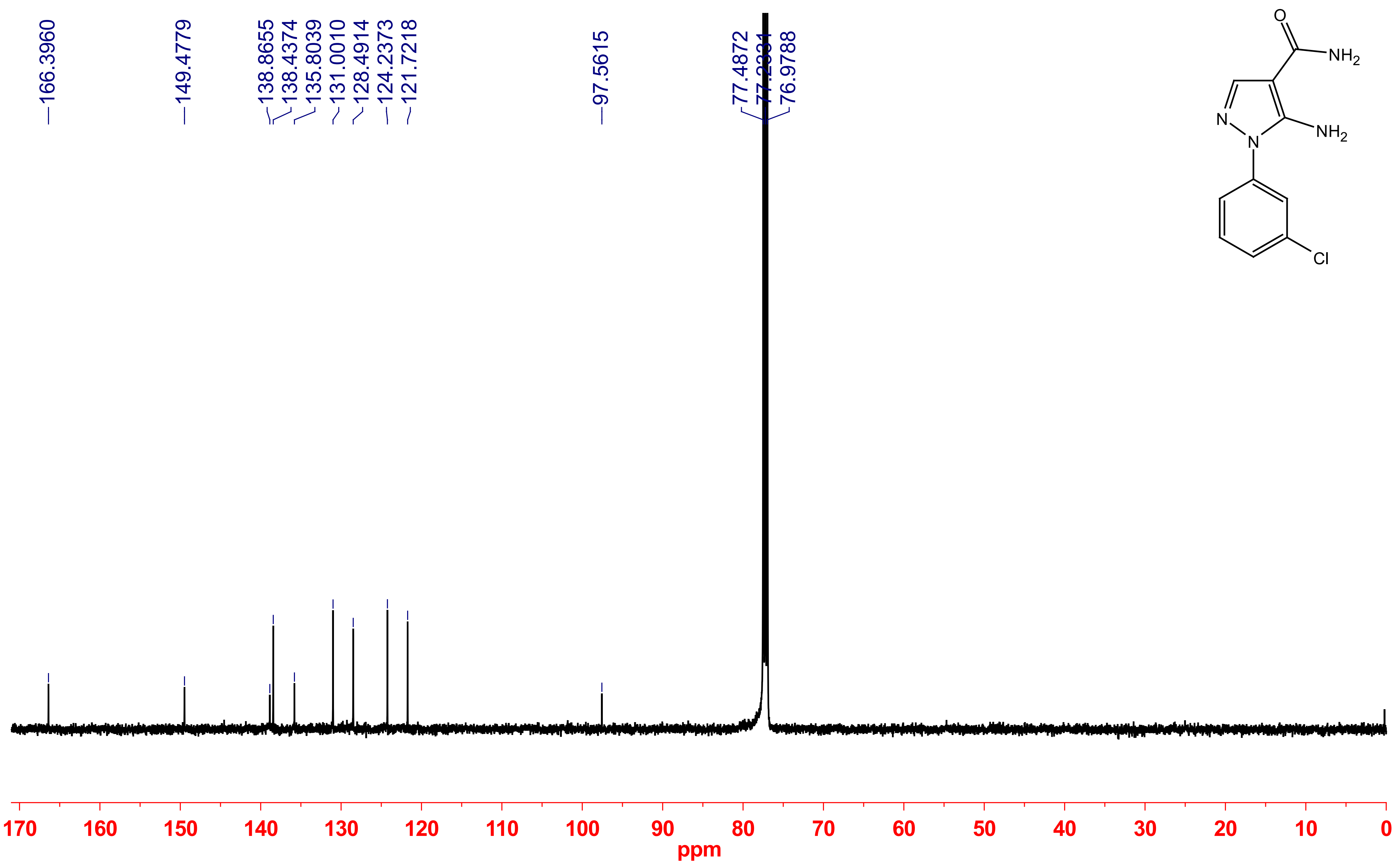

^{1}H NMR of compound **2c**

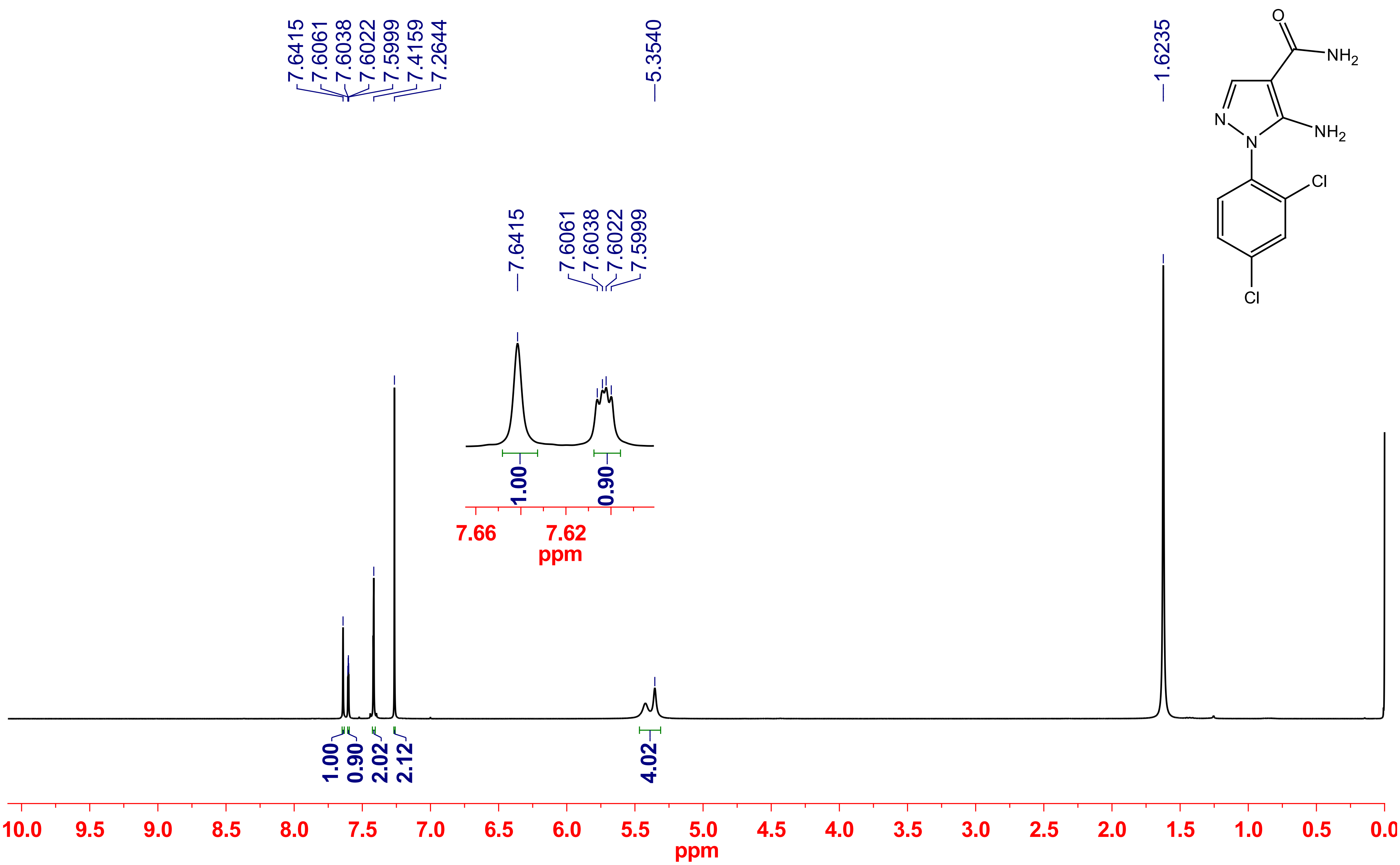

^{13}C NMR of compound **2c**

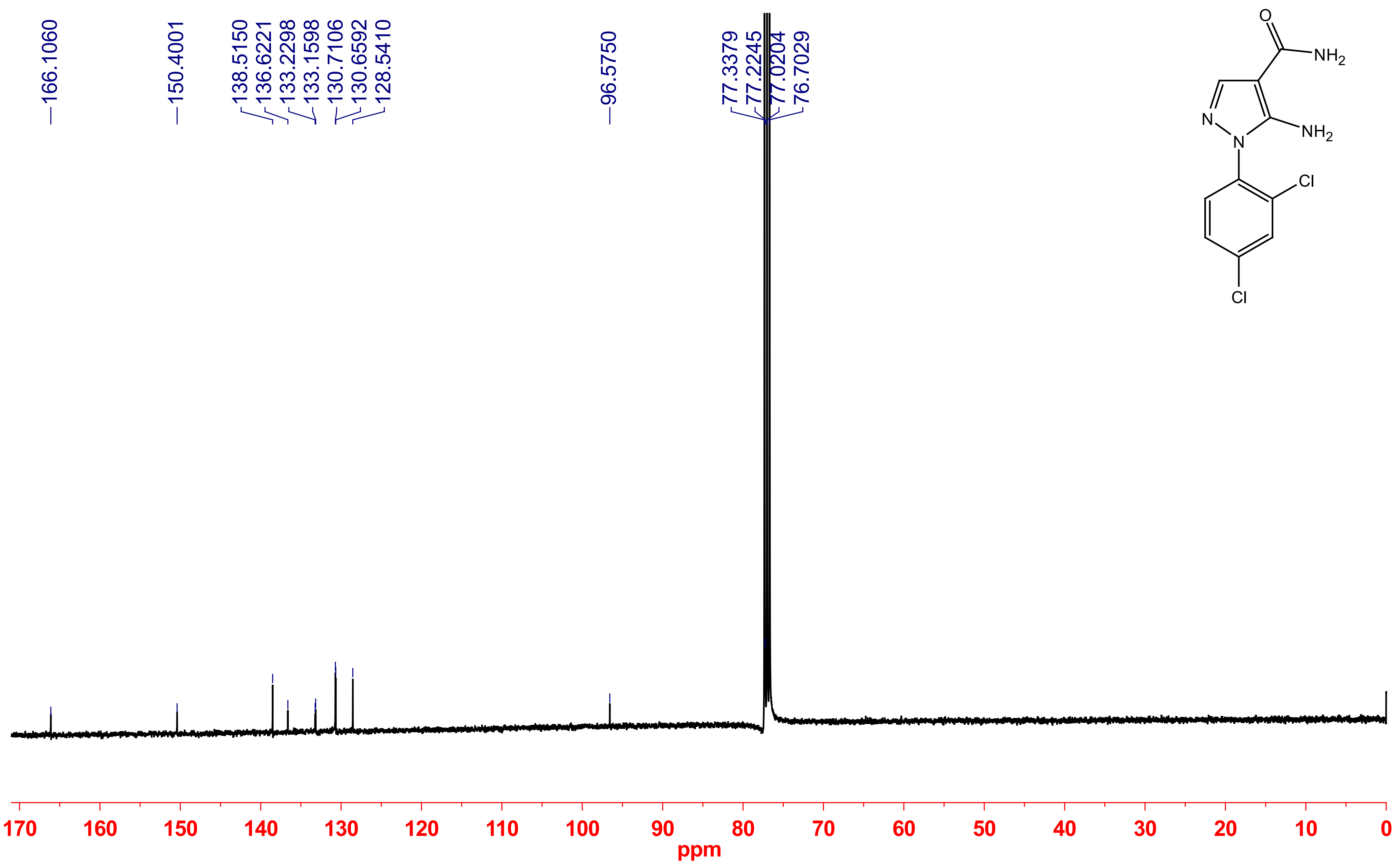

^{1}H NMR of compound **2d**

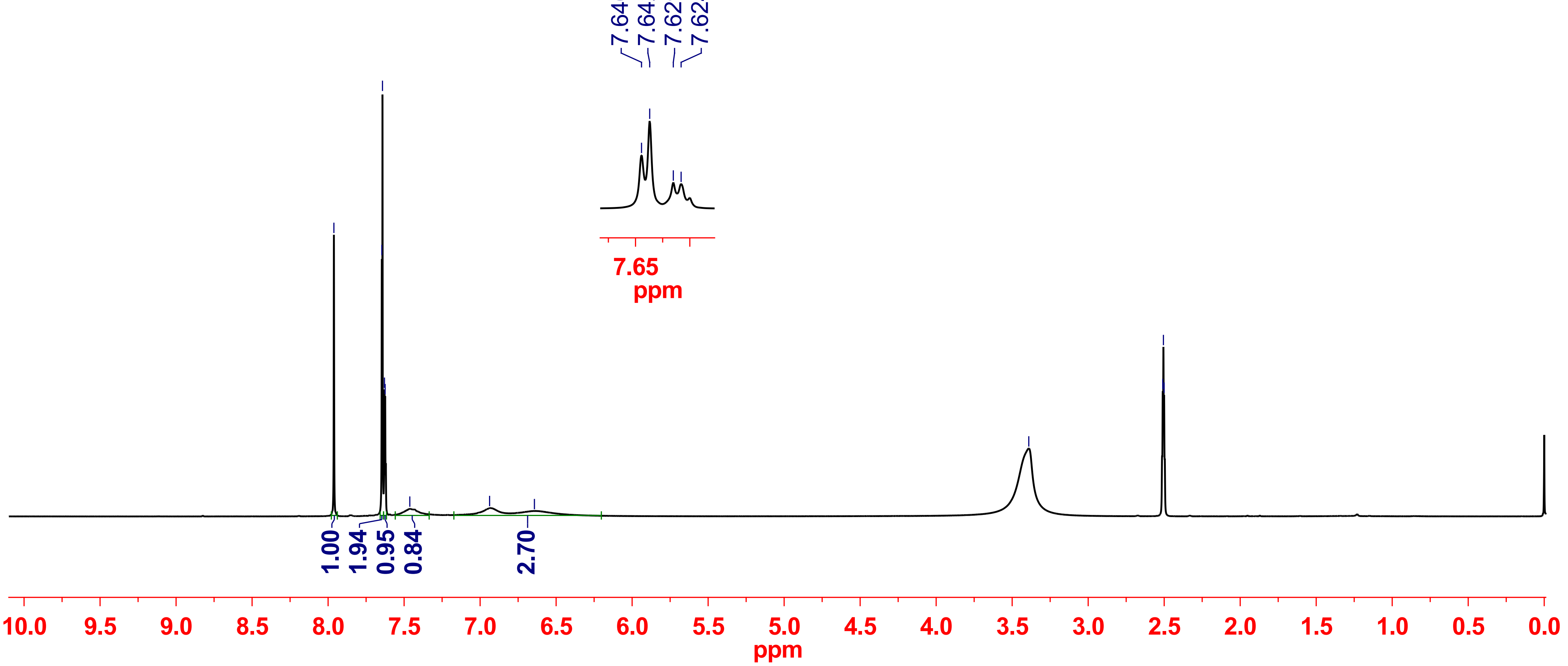

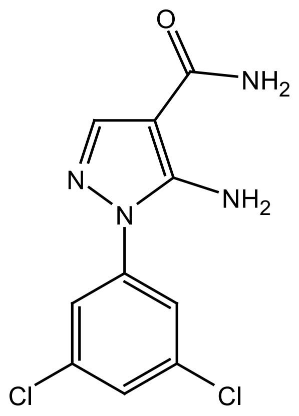

^{13}C NMR of compound **2d**

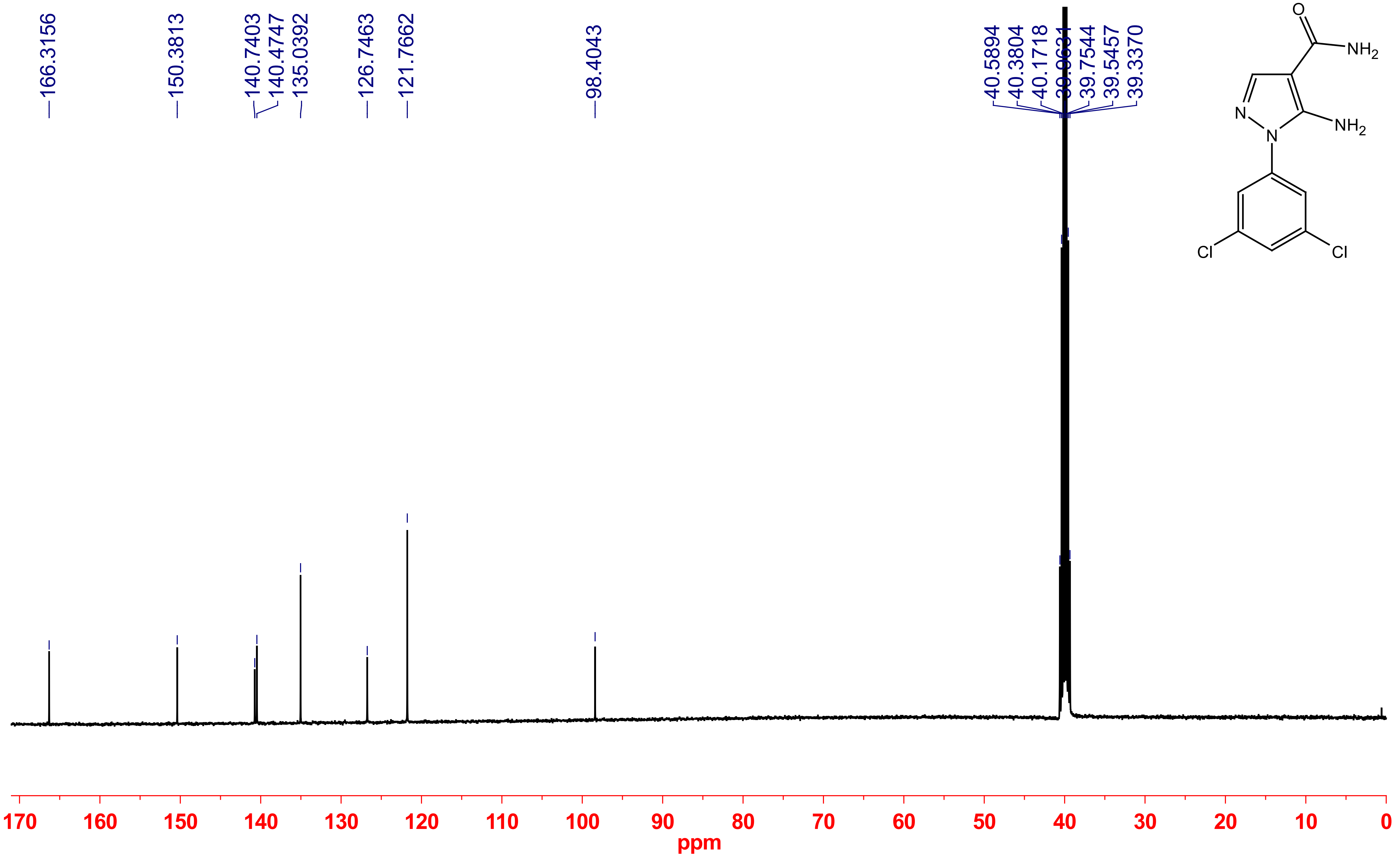

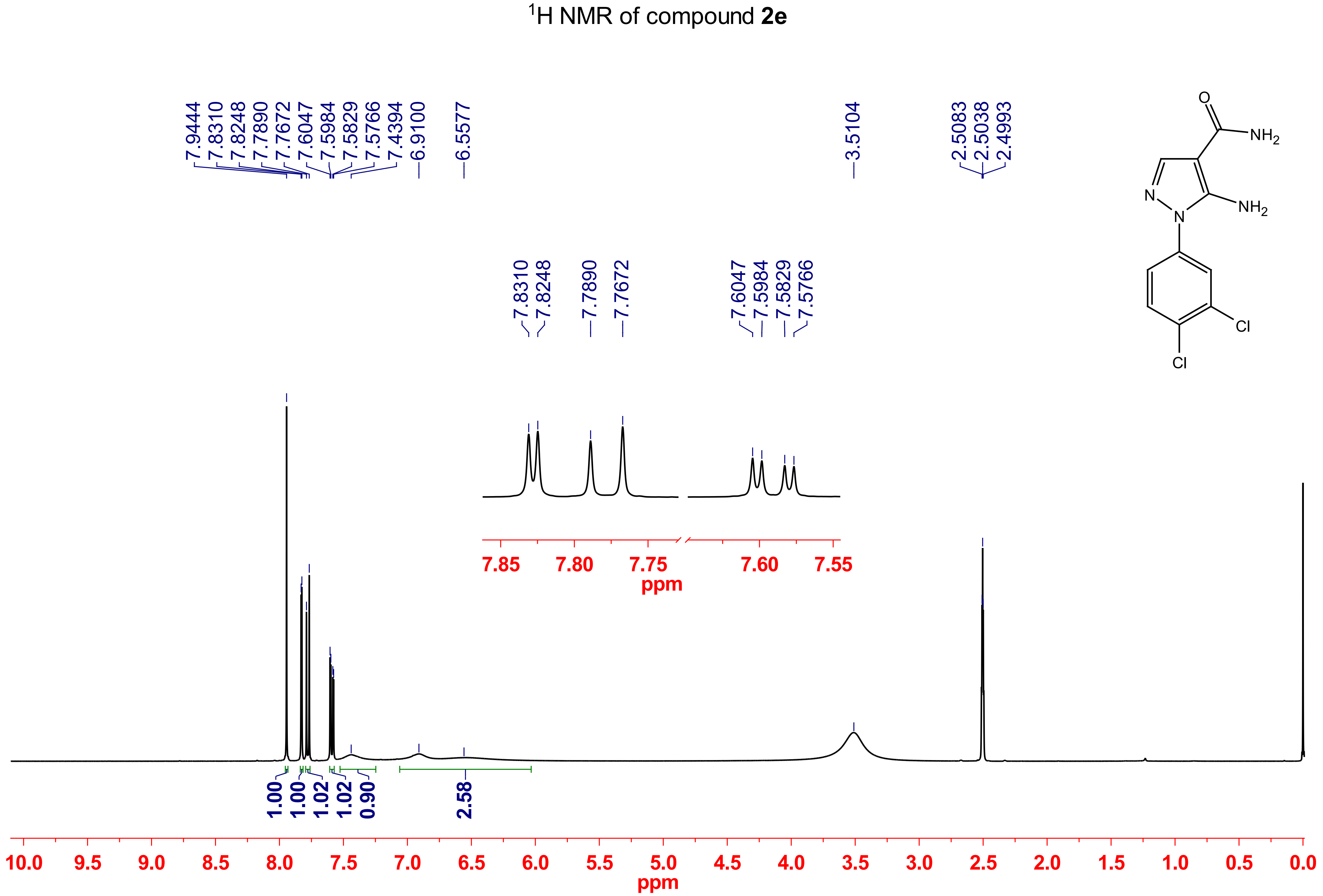
1H NMR of compound 2e
7.9444
7.8310
7.8248
7.7890
7.7672
7.6047
7.5984
7.5829
7.5766
7.4394
6.9100
6.5577
3.5104
2.5083
2.5038
2.4993
O
NH2
N
N
NH2
Cl
Cl
1.00
1.00
1.02
1.02
0.90
2.58
ppm

^{13}C NMR of compound **2e**

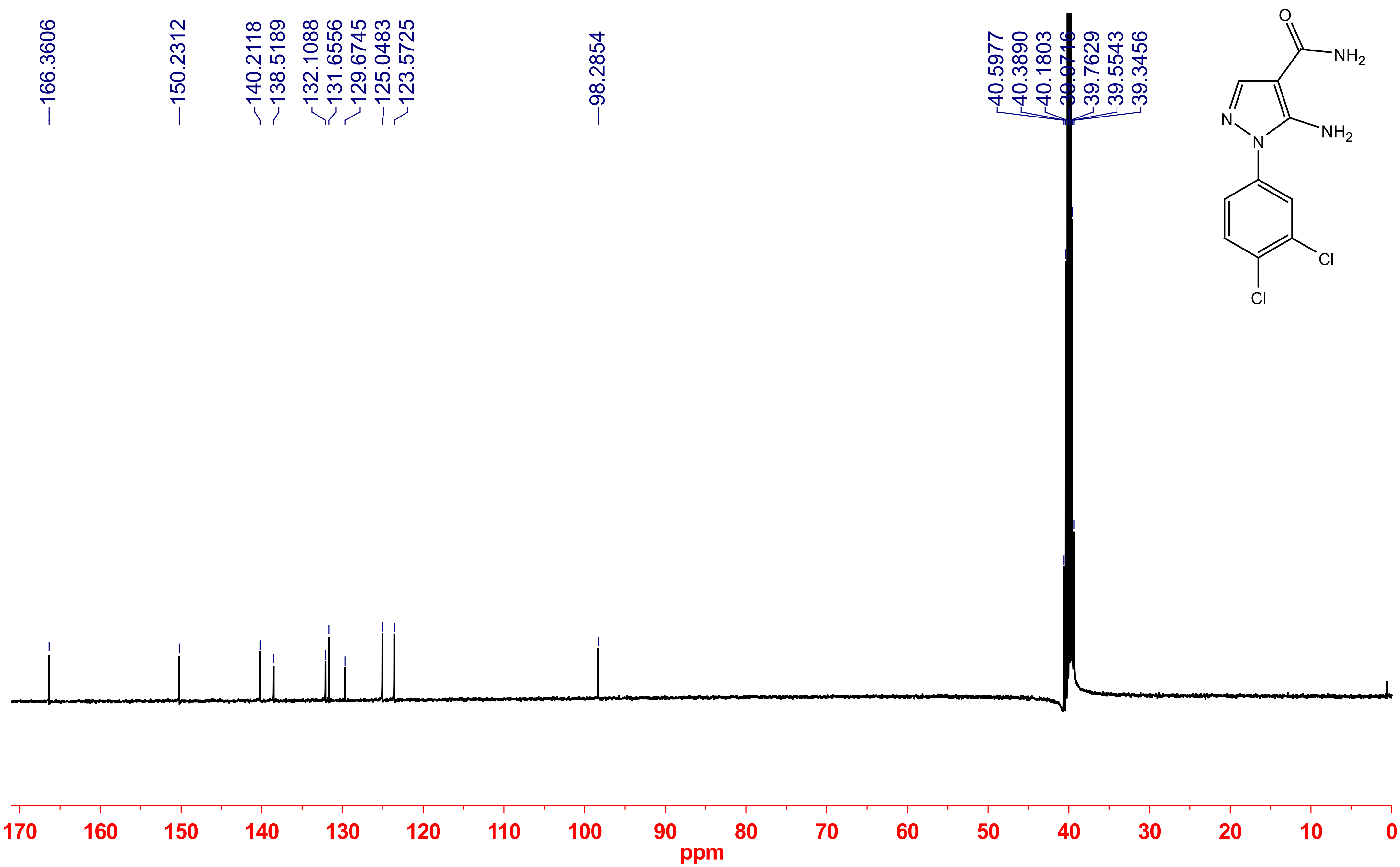

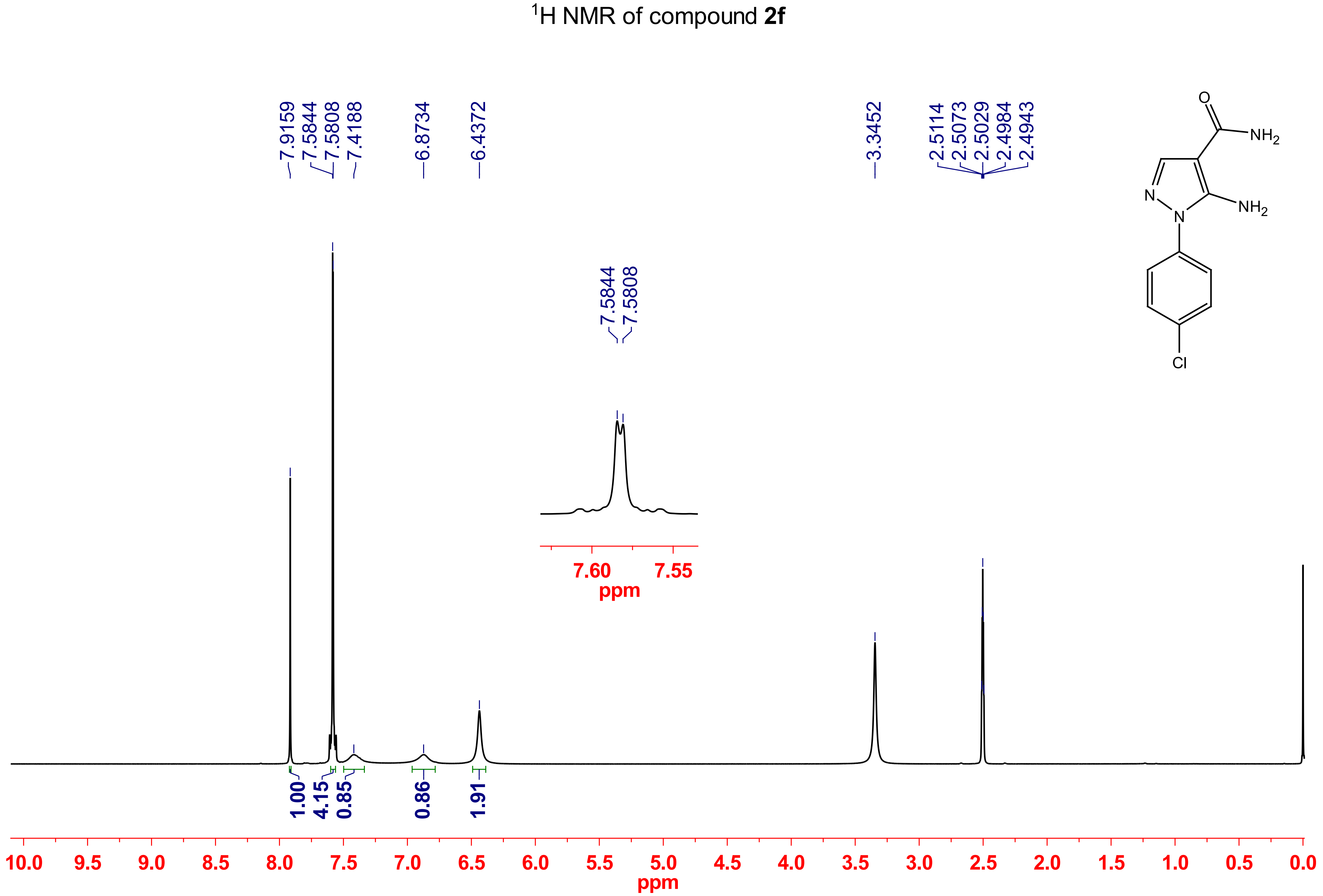

^{1}H NMR of compound **2f**
7.9159
7.5844
7.5808
7.4188
6.8734
6.4372
3.3452
2.5114
2.5073
2.5029
2.4984
2.4943
7.5844
7.5808
7.60
7.55
ppm
1.00
4.15
0.85
0.86
1.91
10.0 9.5 9.0 8.5 8.0 7.5 7.0 6.5 6.0 5.5 5.0 4.5 4.0 3.5 3.0 2.5 2.0 1.5 1.0 0.5 0.0
ppm
O
NH2
N
N
NH2
Cl

^{13}C NMR of compound **2f**

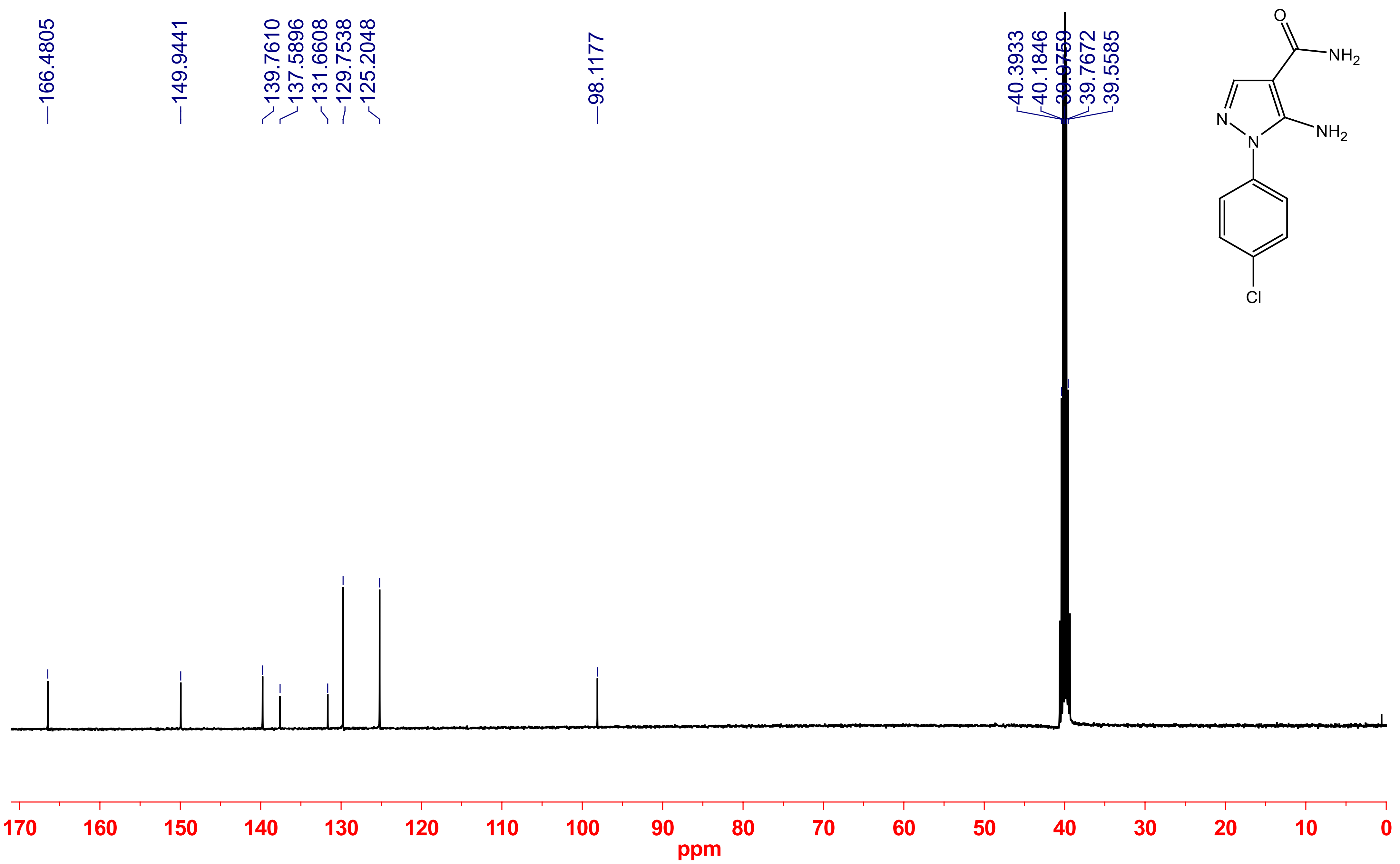

^{1}H NMR of compound **2g**

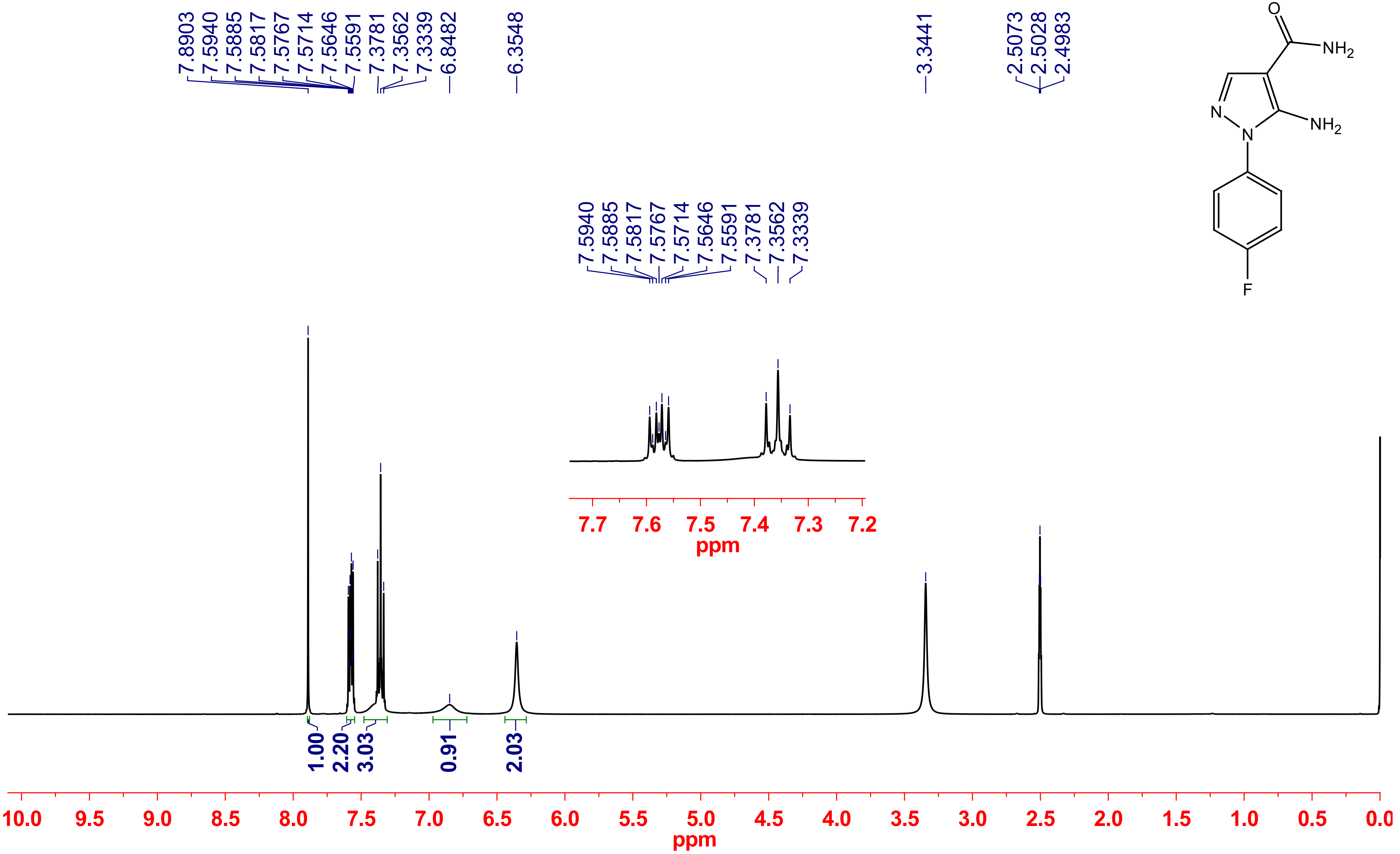

^{13}C NMR of compound **2g**

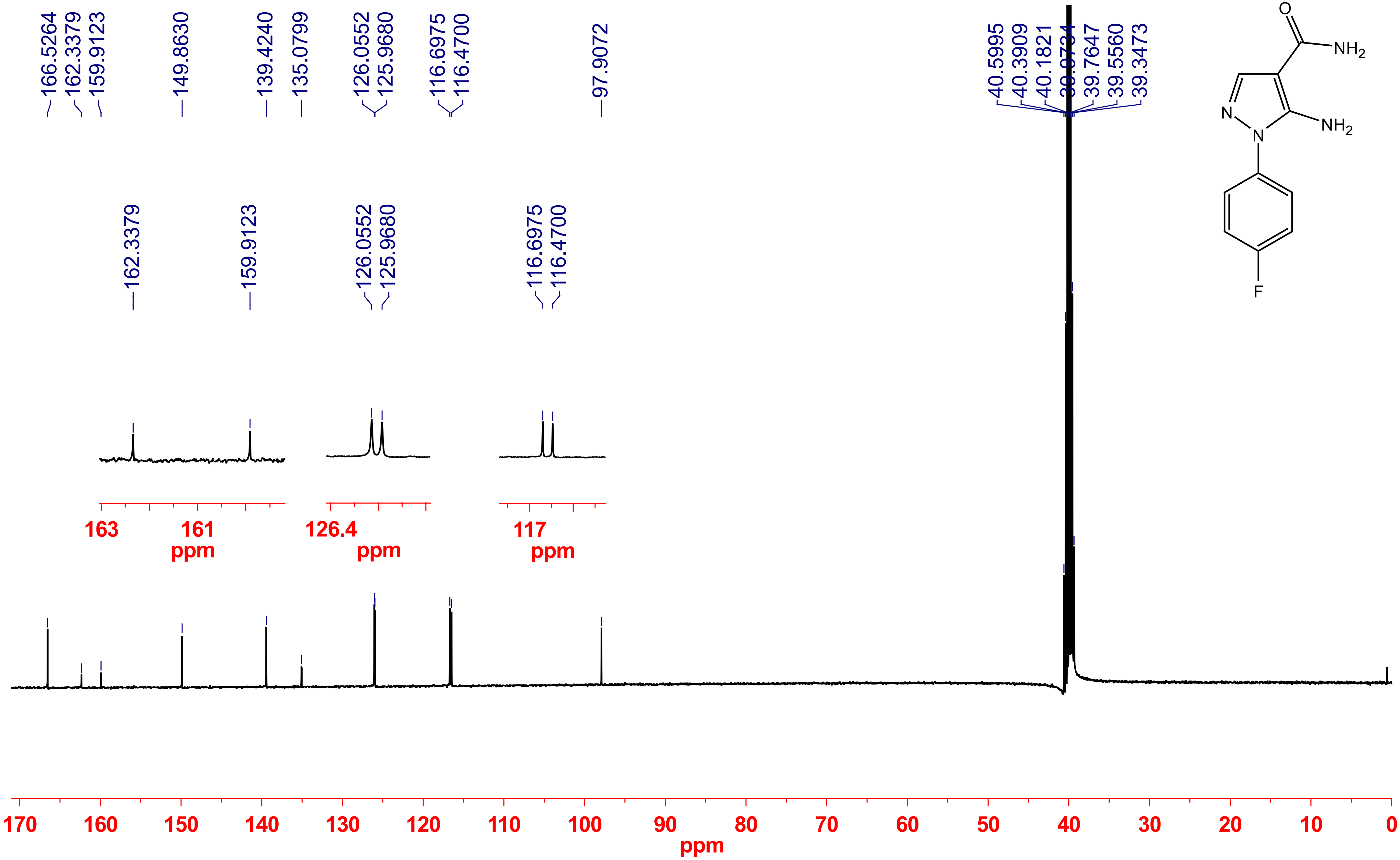

^{1}H NMR of compound **2h**

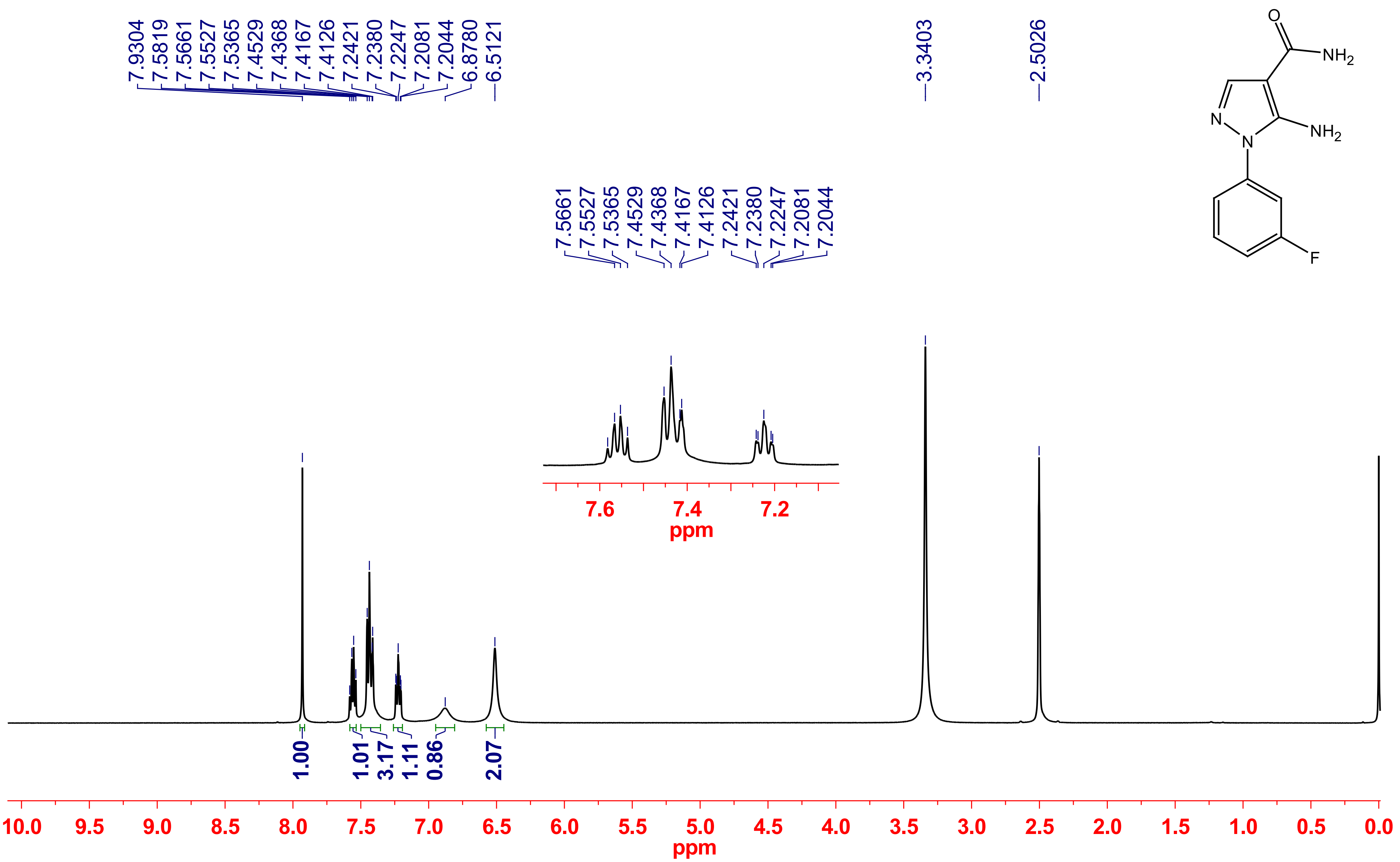

^{13}C NMR of compound **2h**

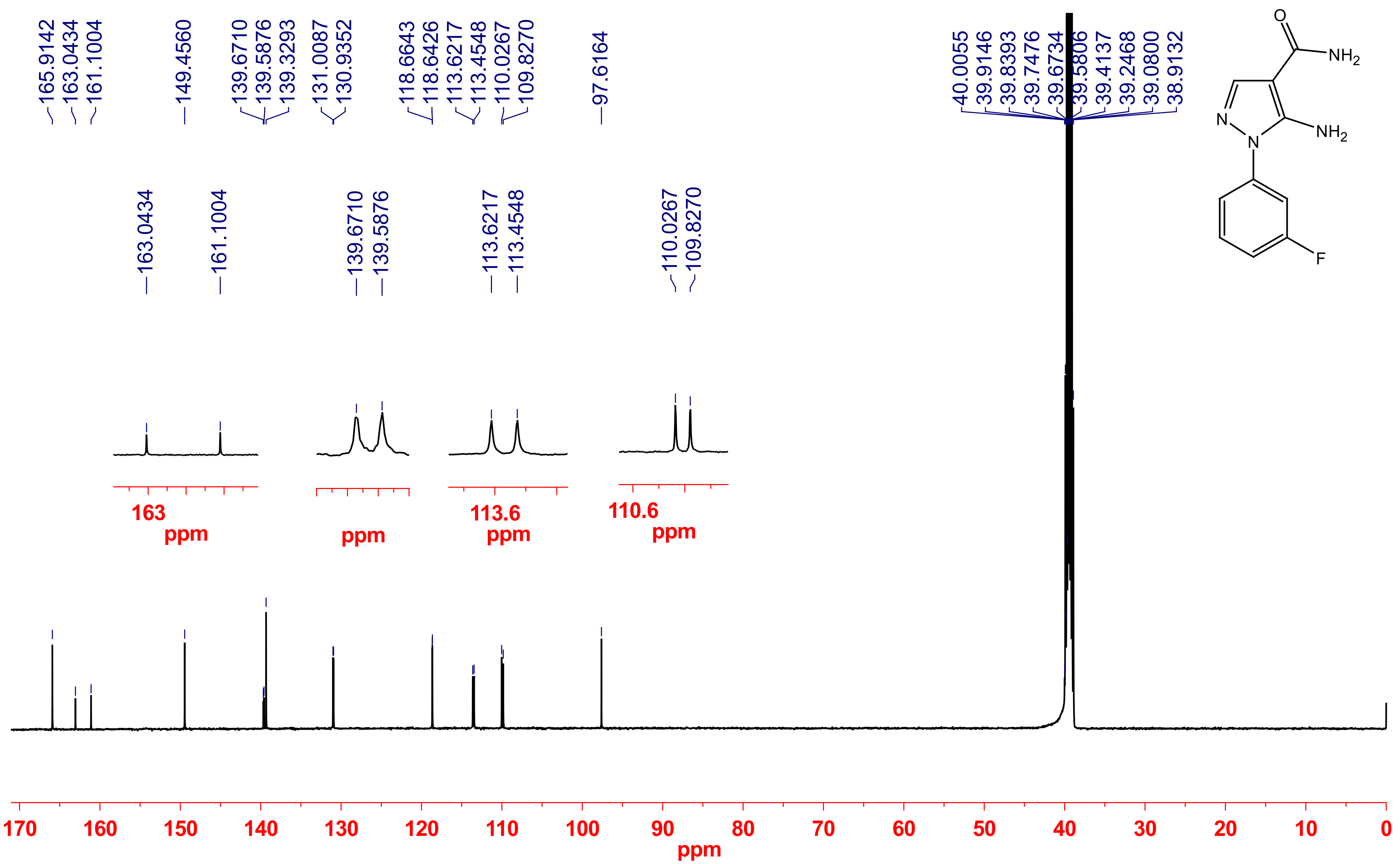

^{1}H NMR of compound **2i**

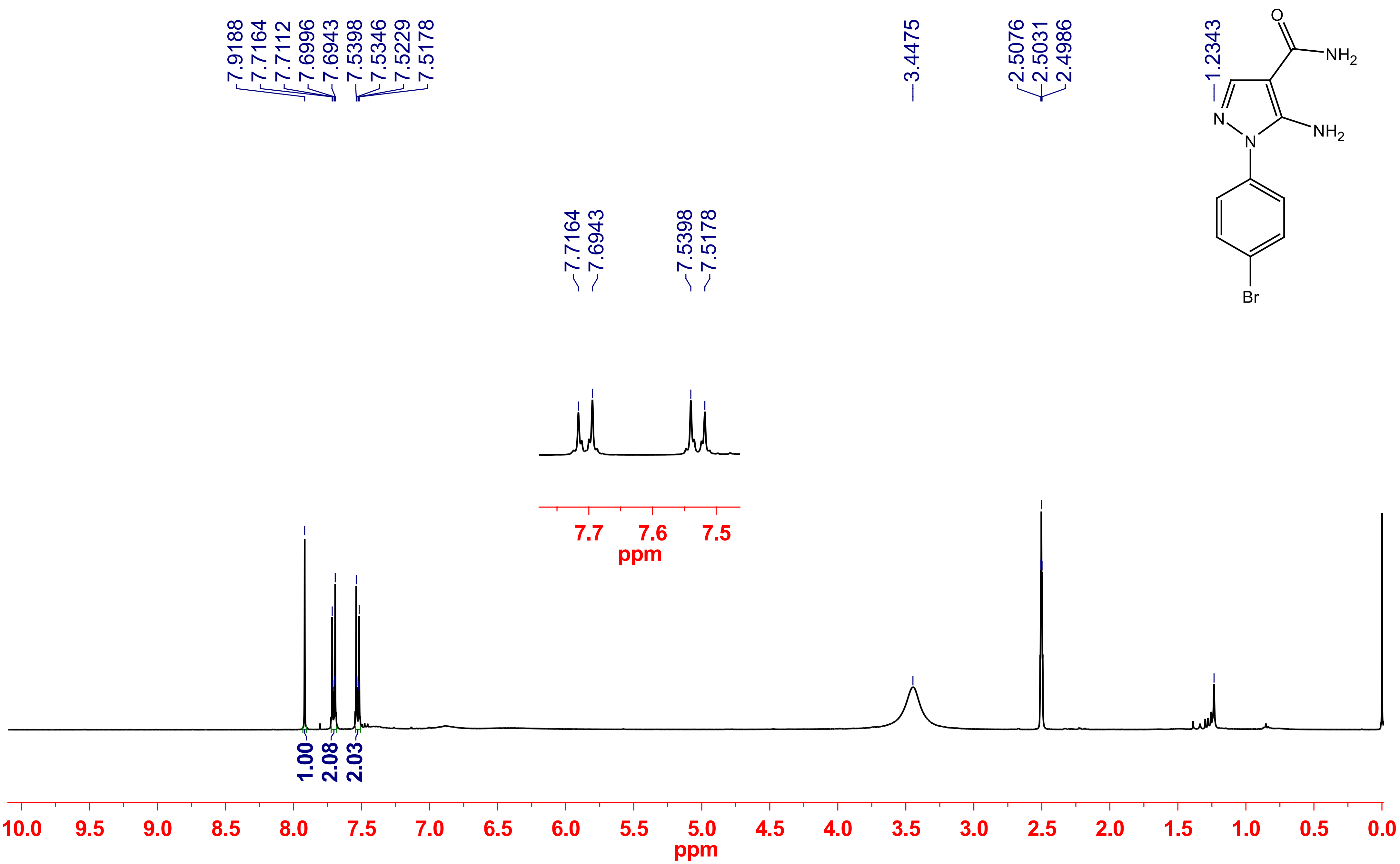

^{13}C NMR of compound **2i**

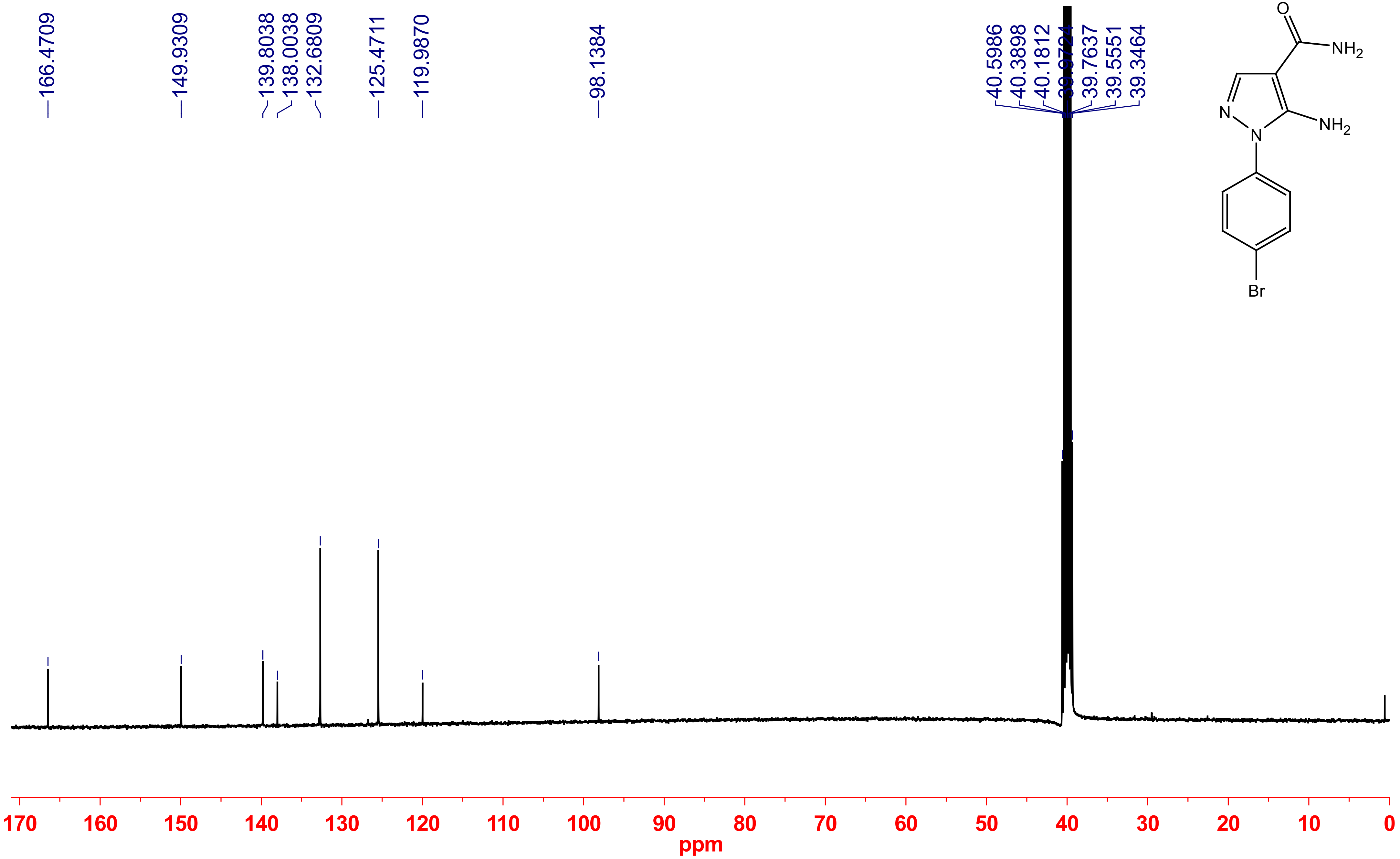

^1H NMR of compound **2j**

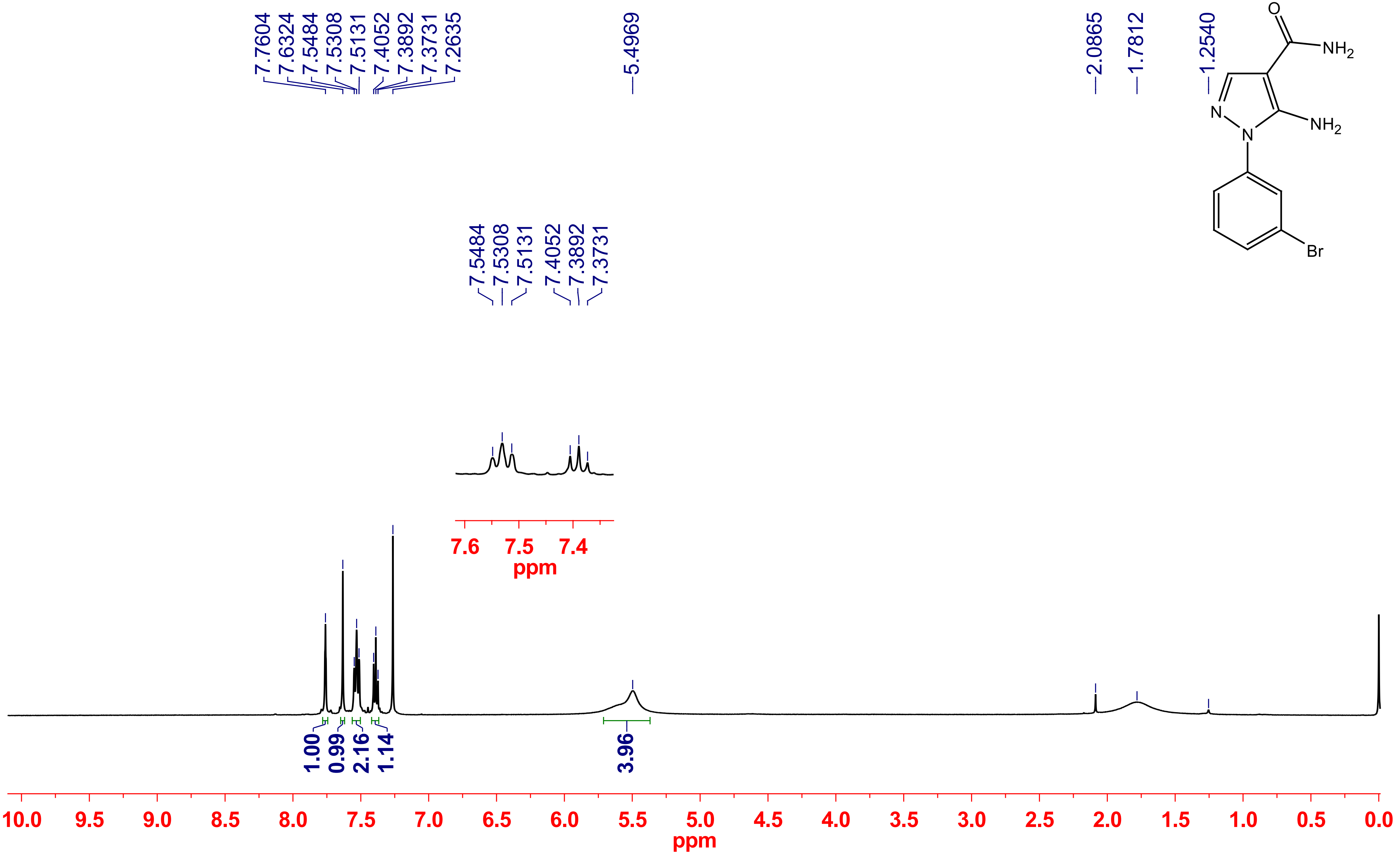

^{13}C NMR of compound **2j**

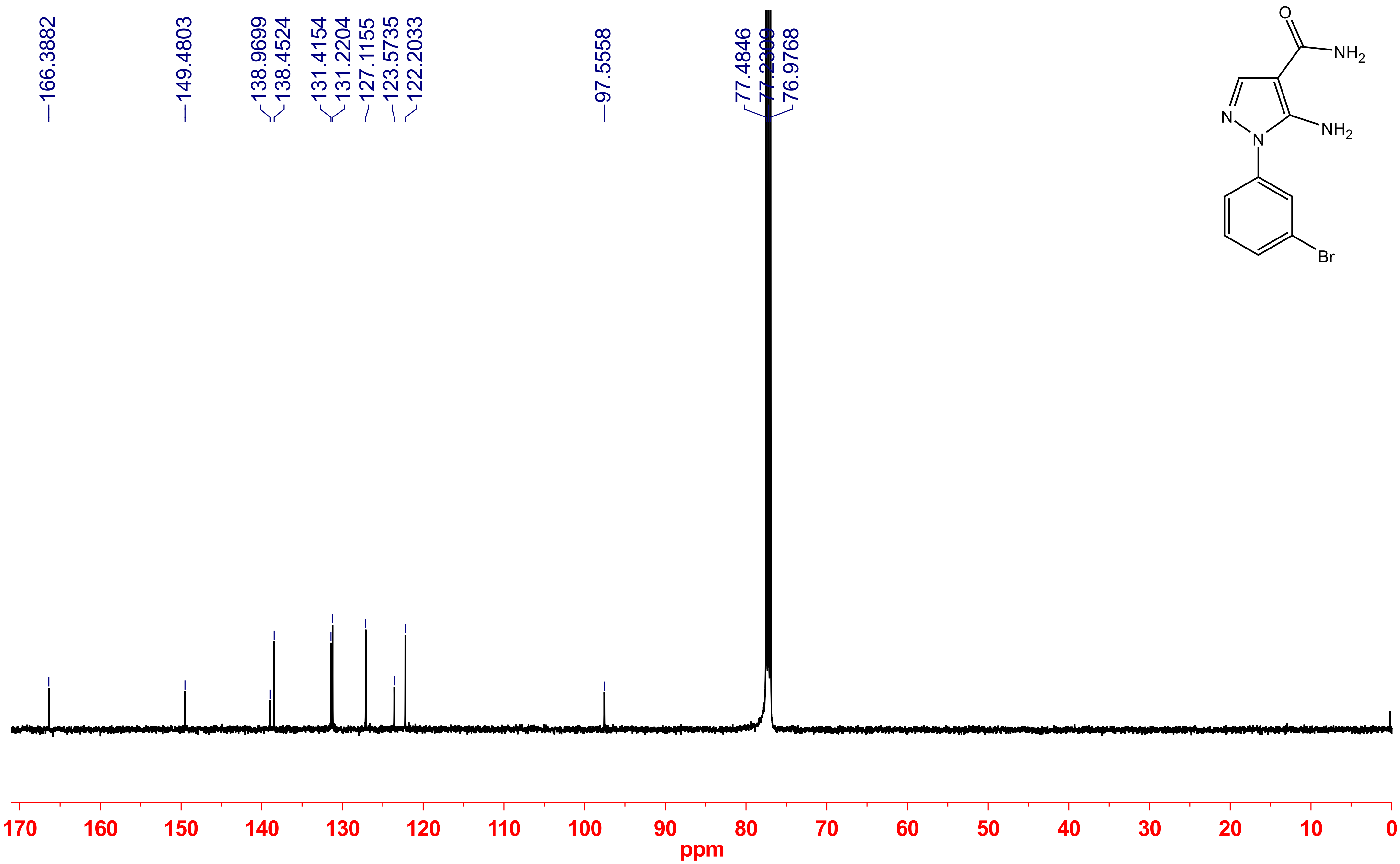

^{1}H NMR of compound **2k**

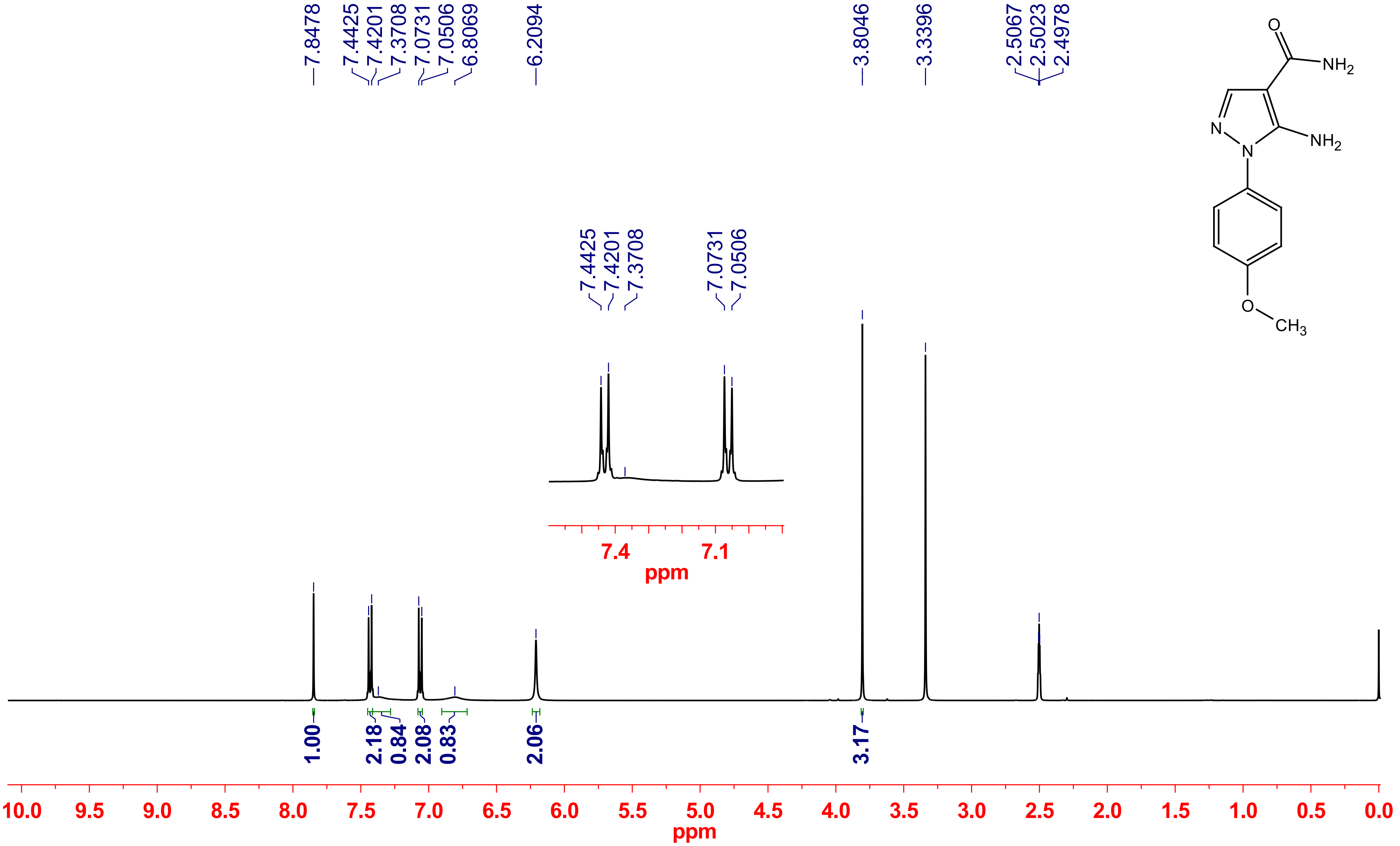

^{13}C NMR of compound **2k**

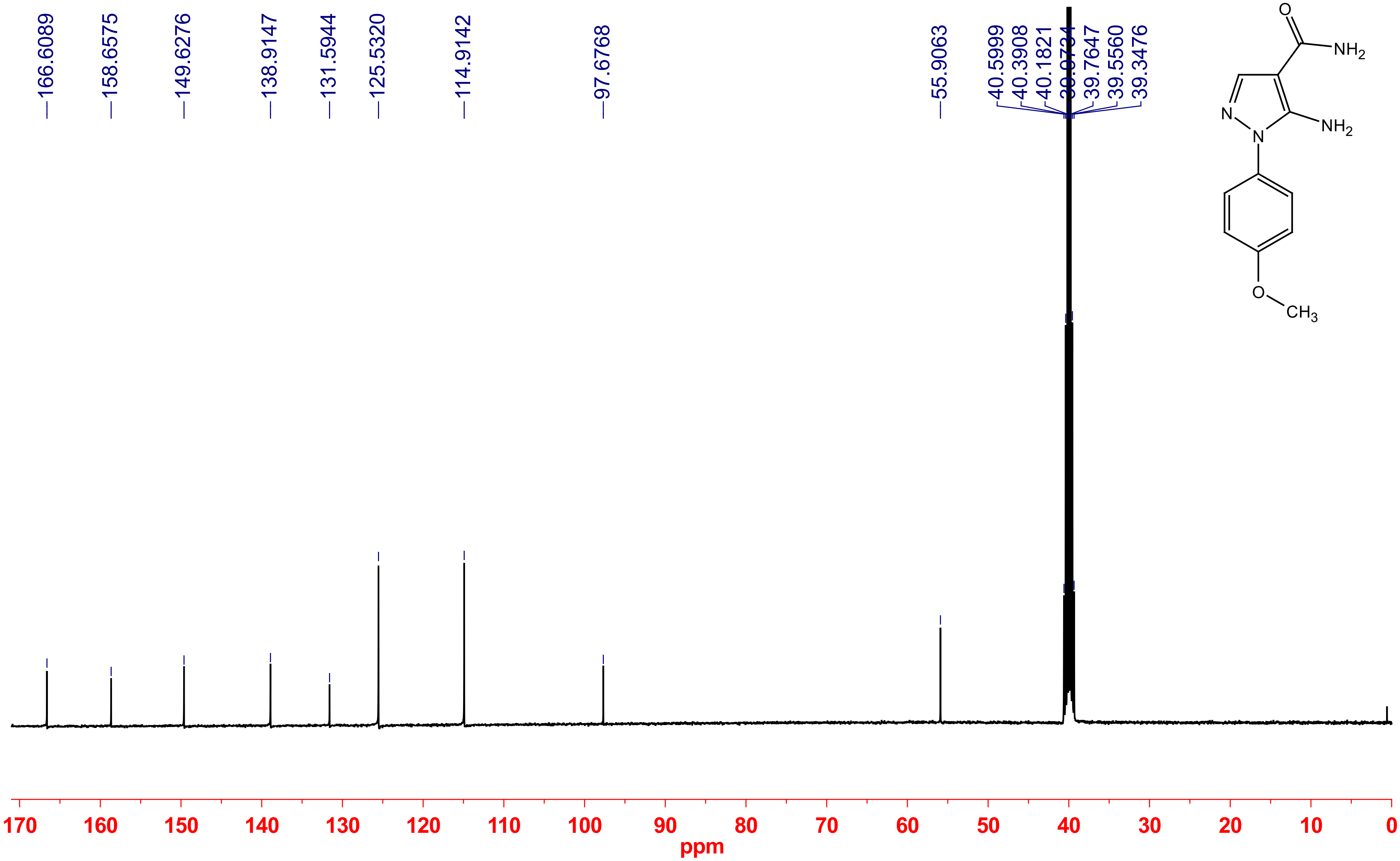

^{1}H NMR of compound **2l**

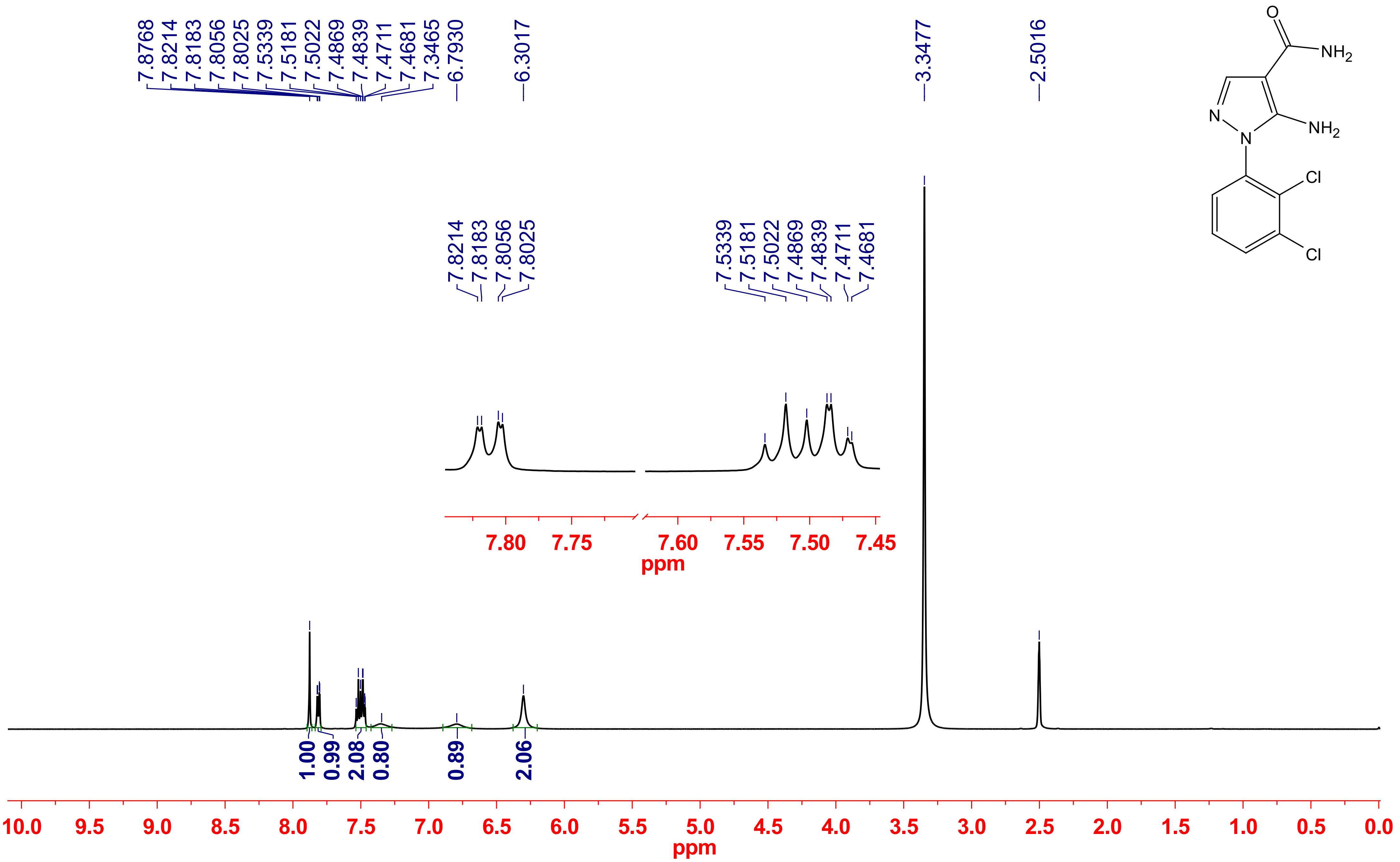

^{13}C NMR of compound **2l**

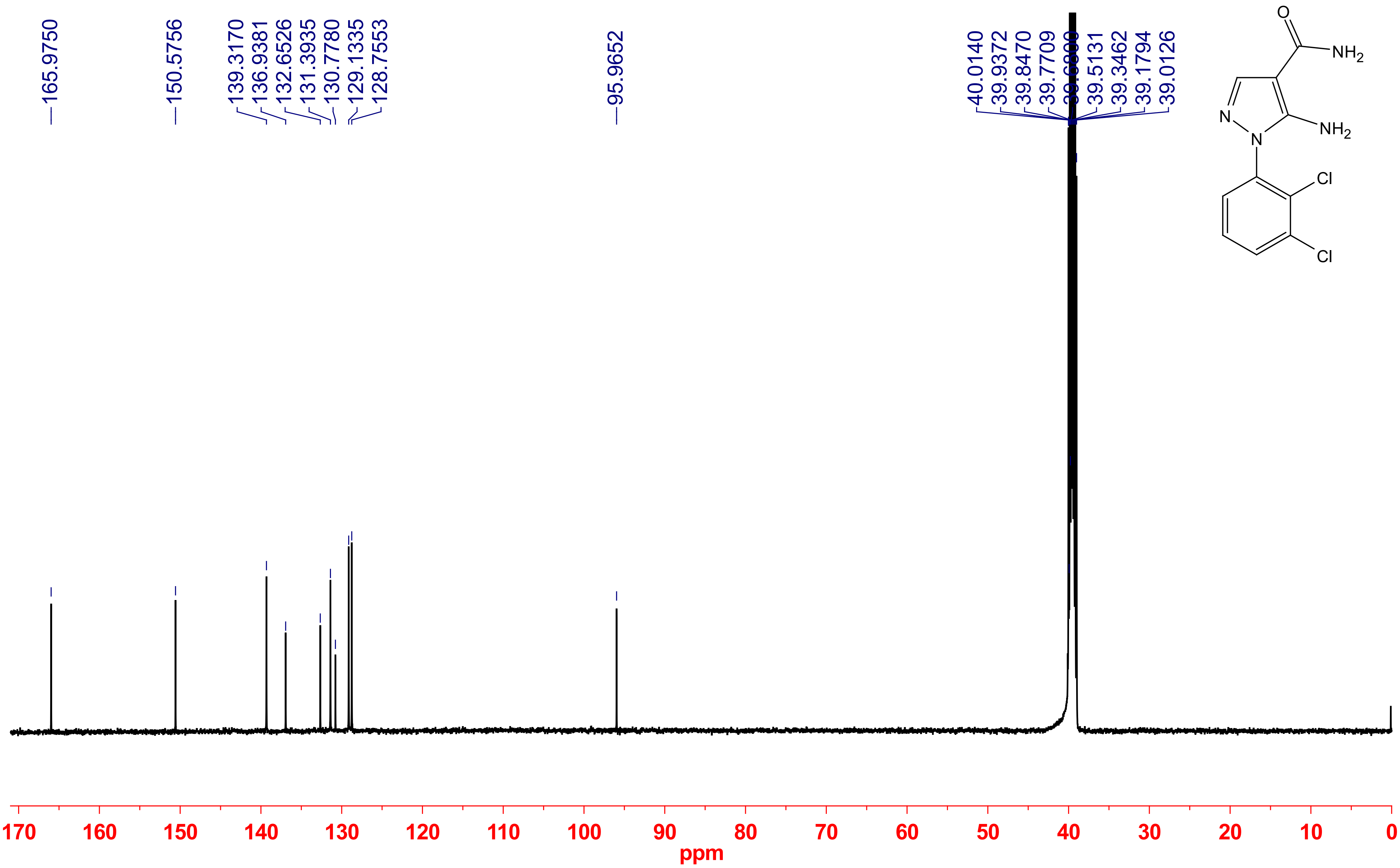

^{1}H NMR of compound **3a**

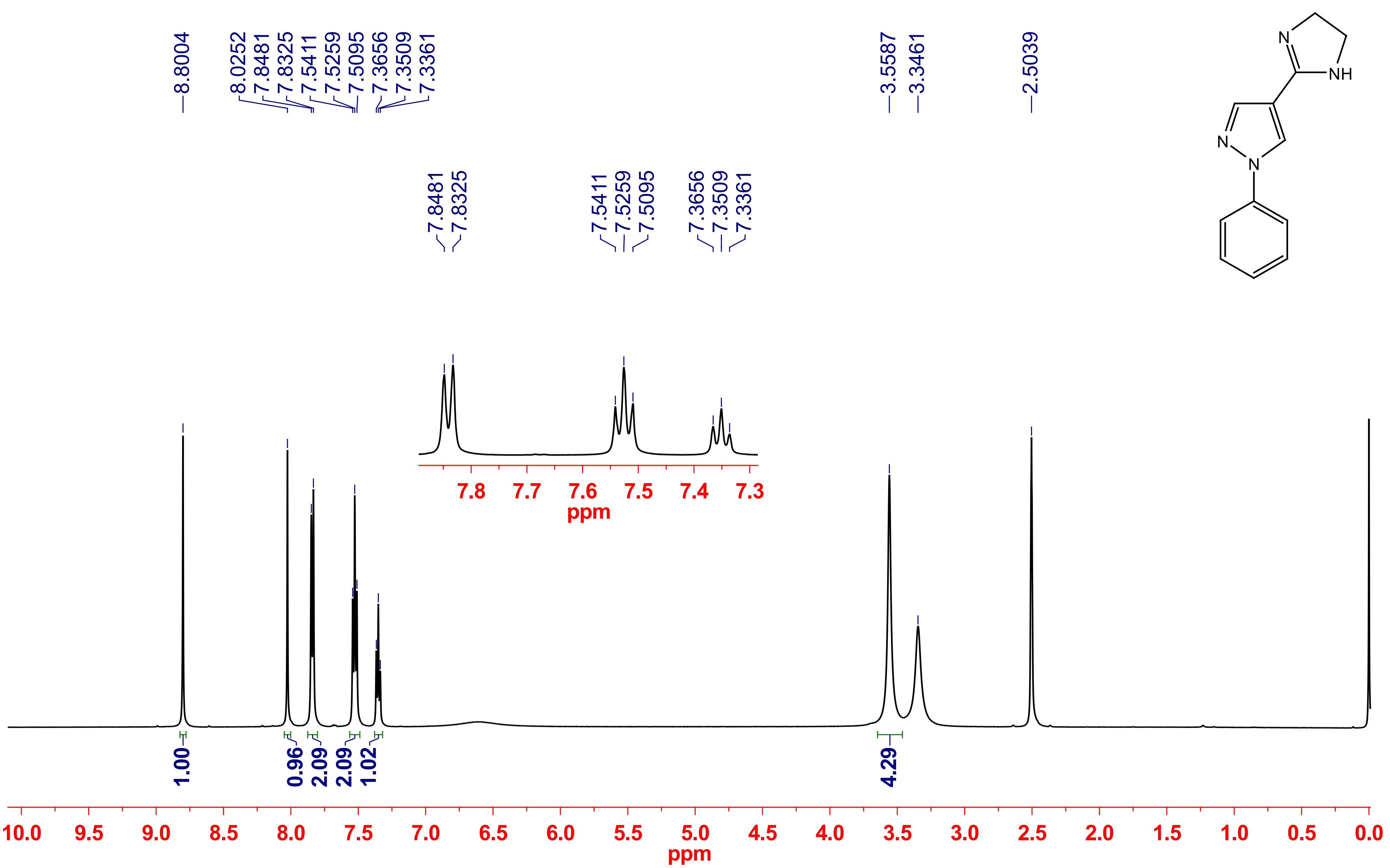

^{13}C NMR of compound **3a**

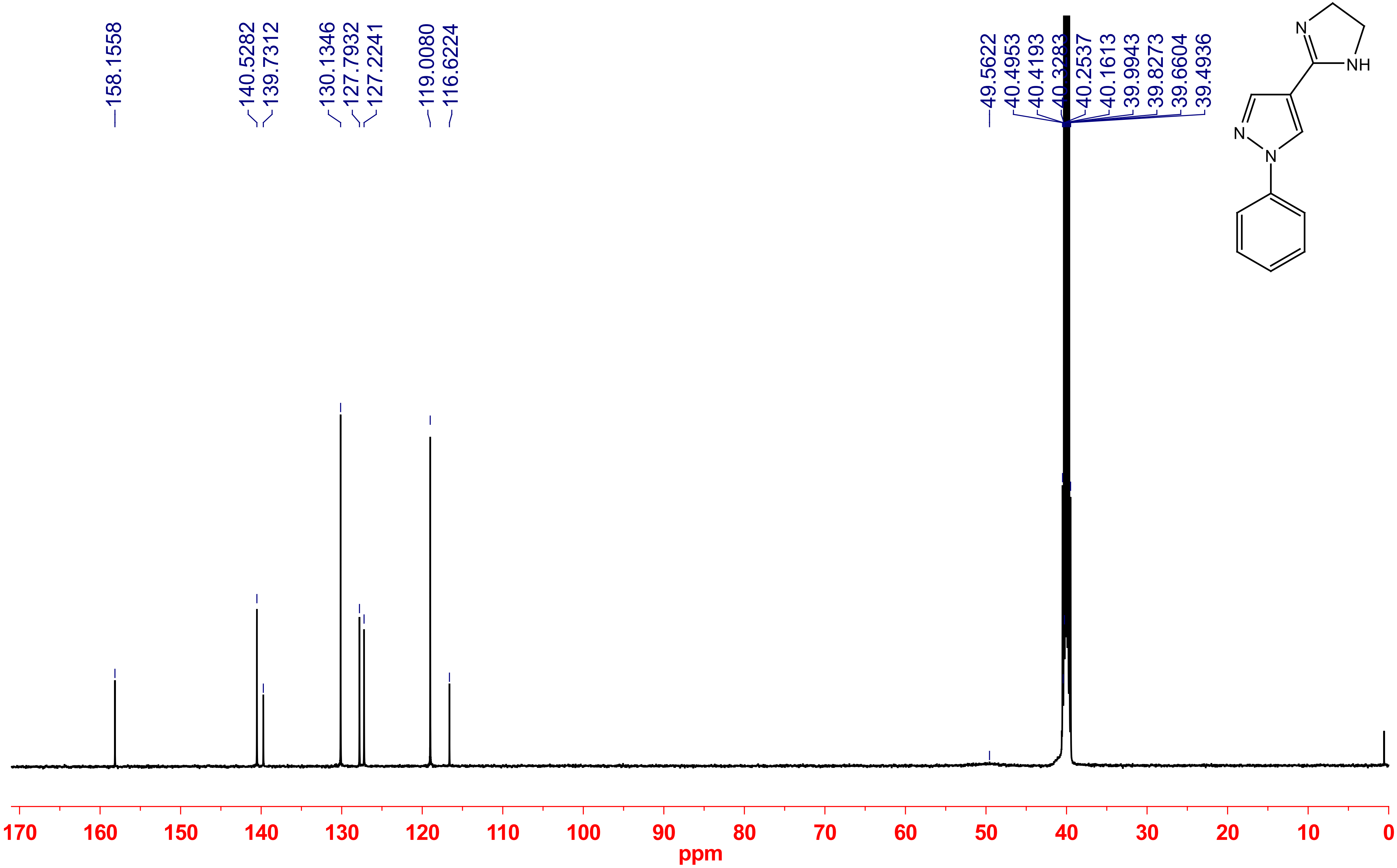

^{1}H NMR of compound **3b**

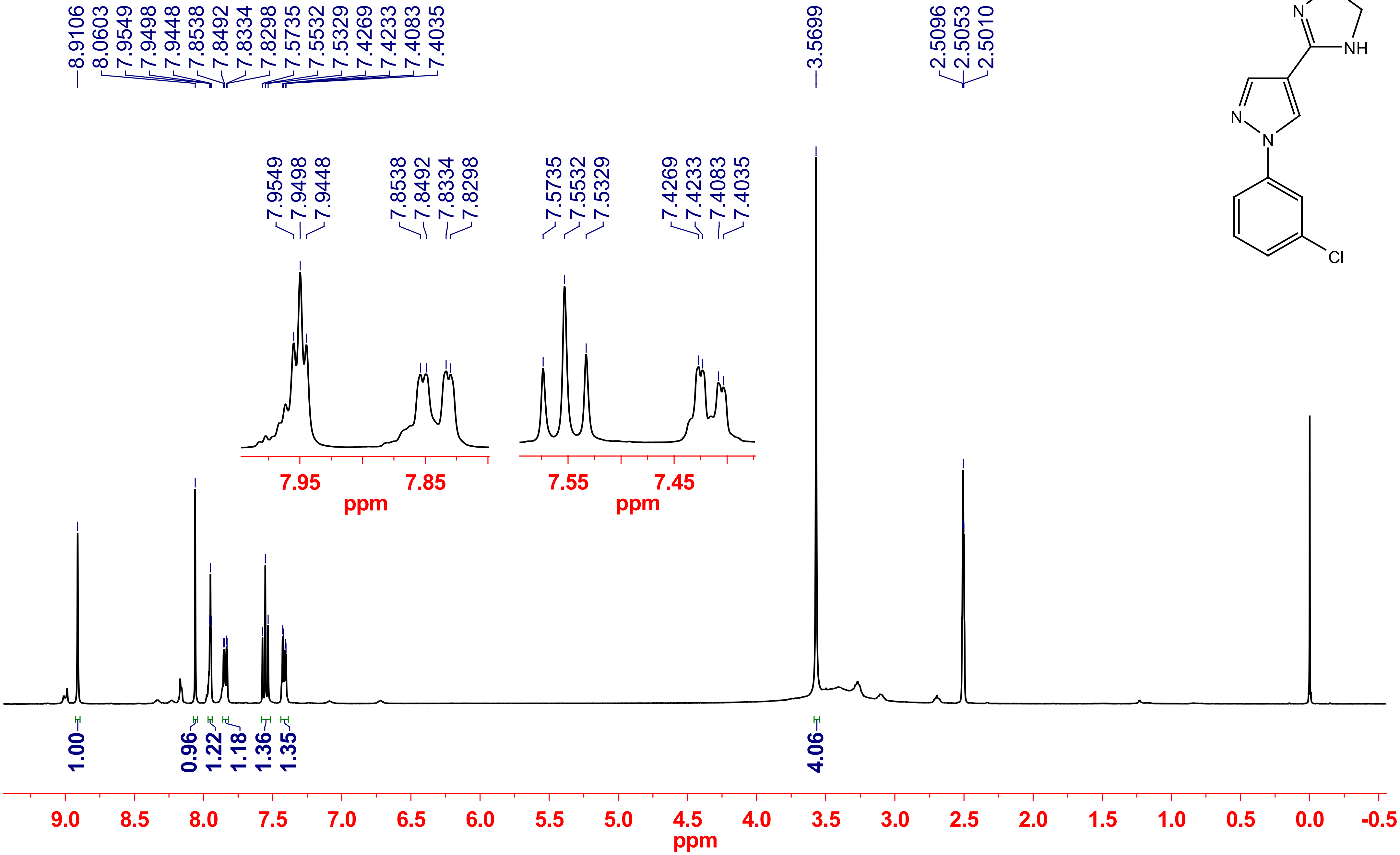

^{13}C NMR of compound **3b**

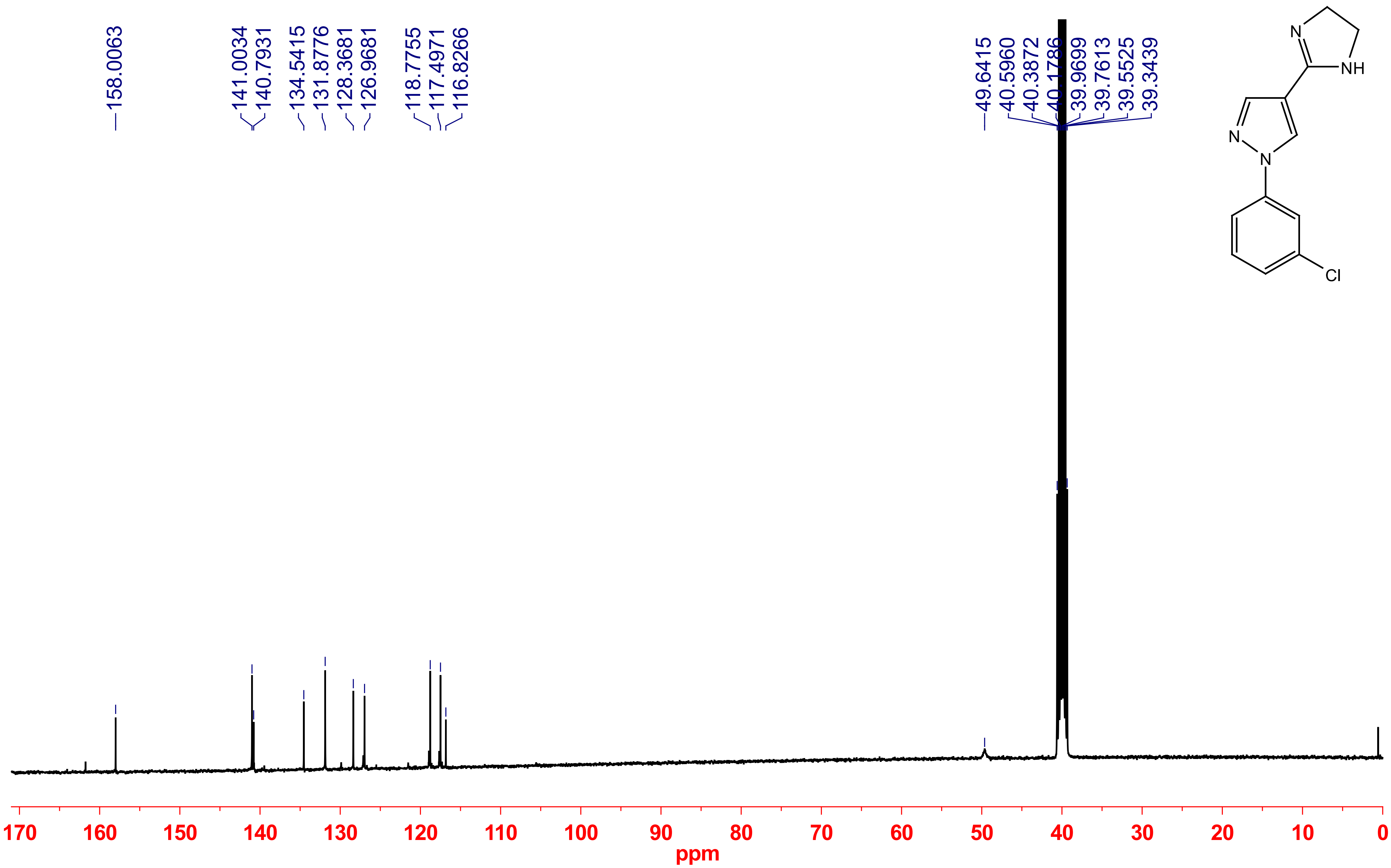

^{1}H NMR of compound **3c**

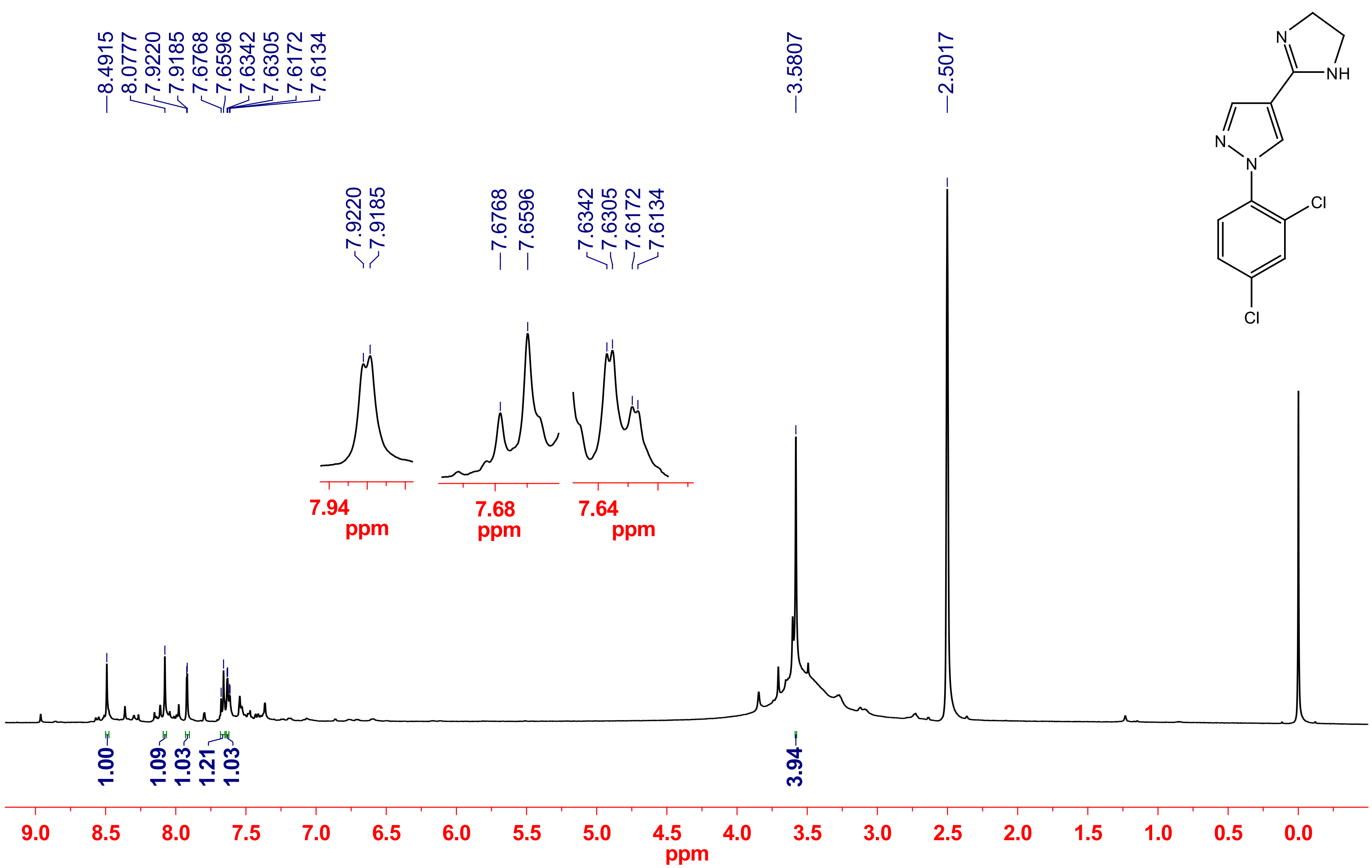

^{13}C NMR of compound **3c**

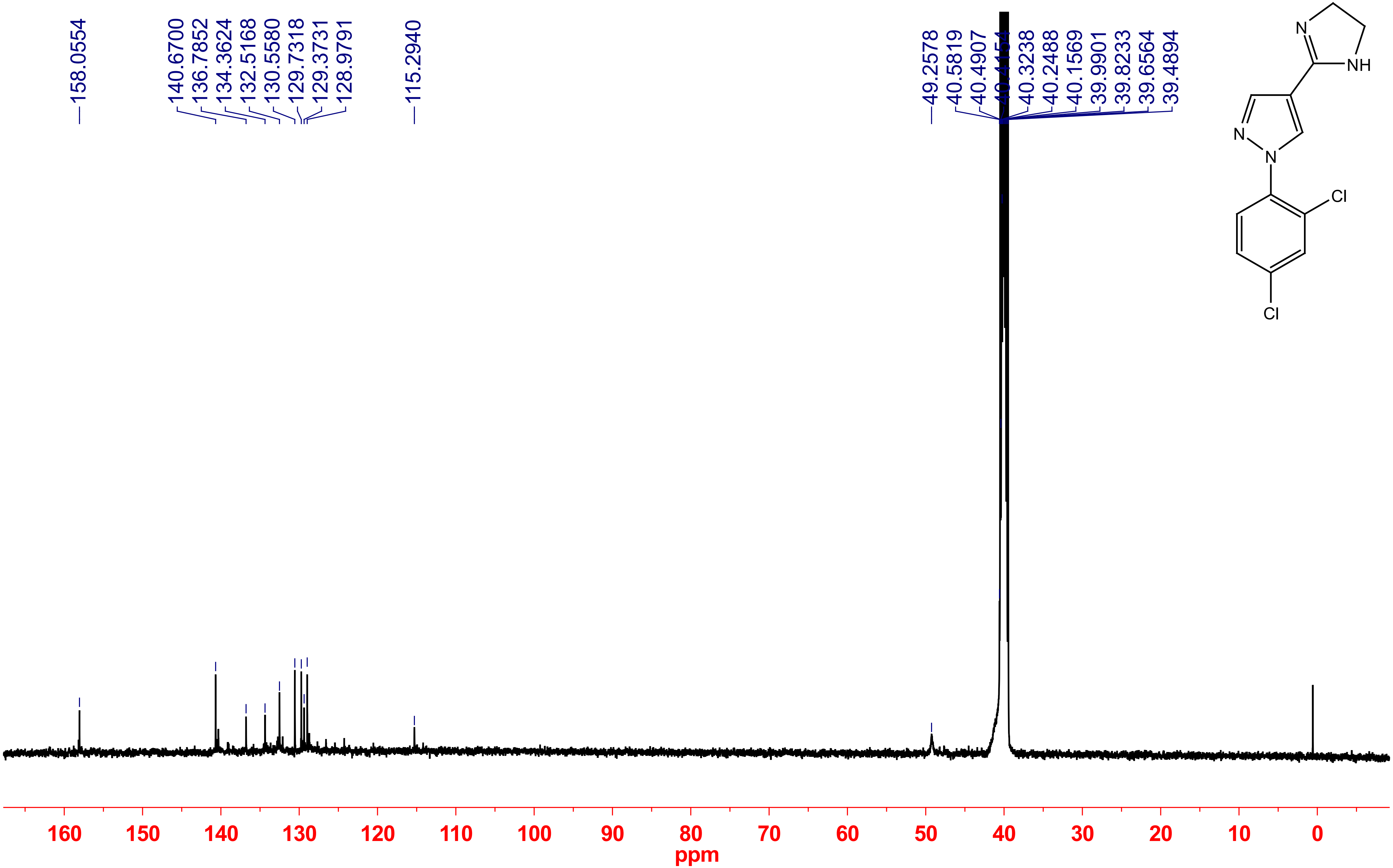

^{1}H NMR of compound **3d**

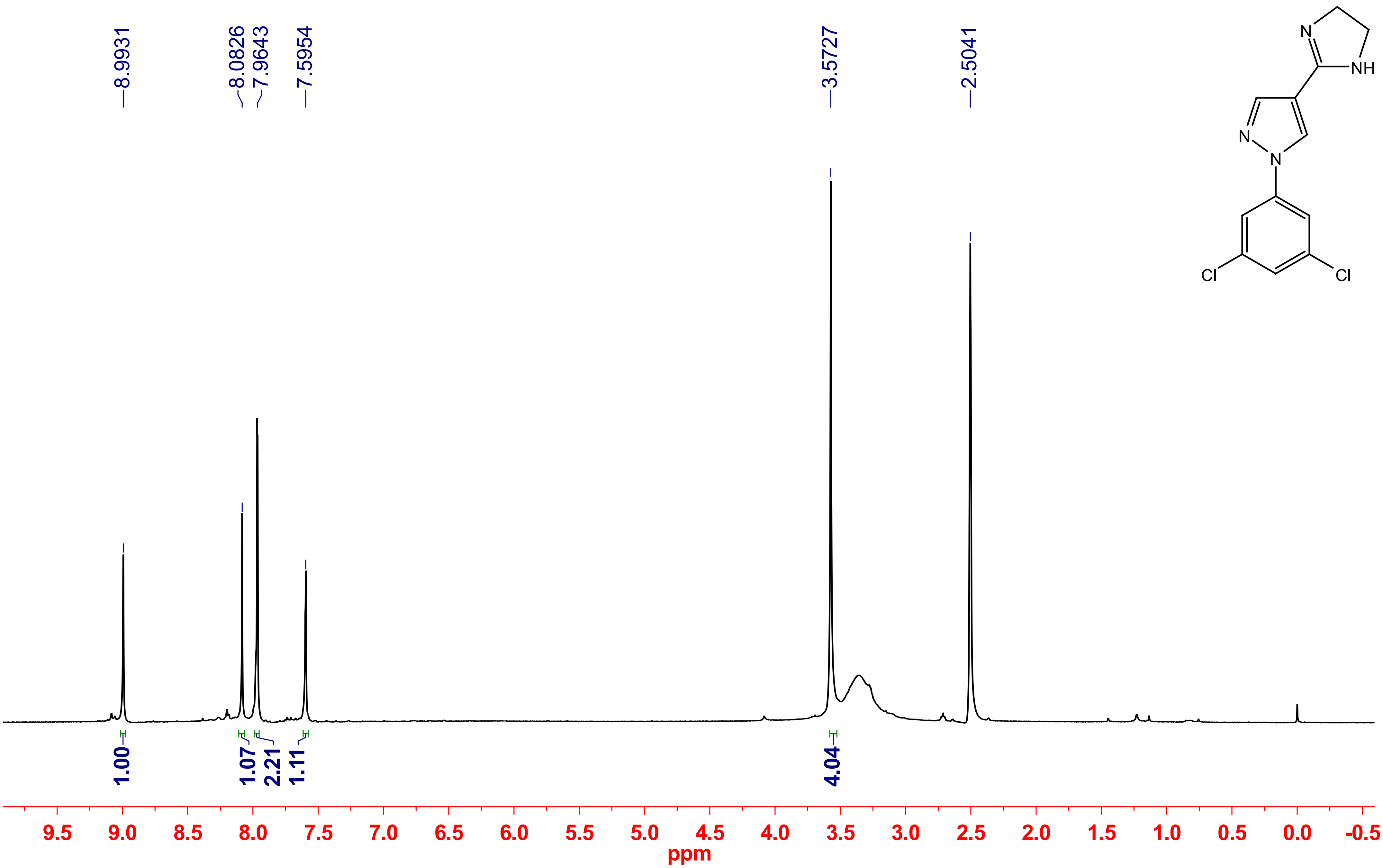

^{13}C NMR of compound **3d**

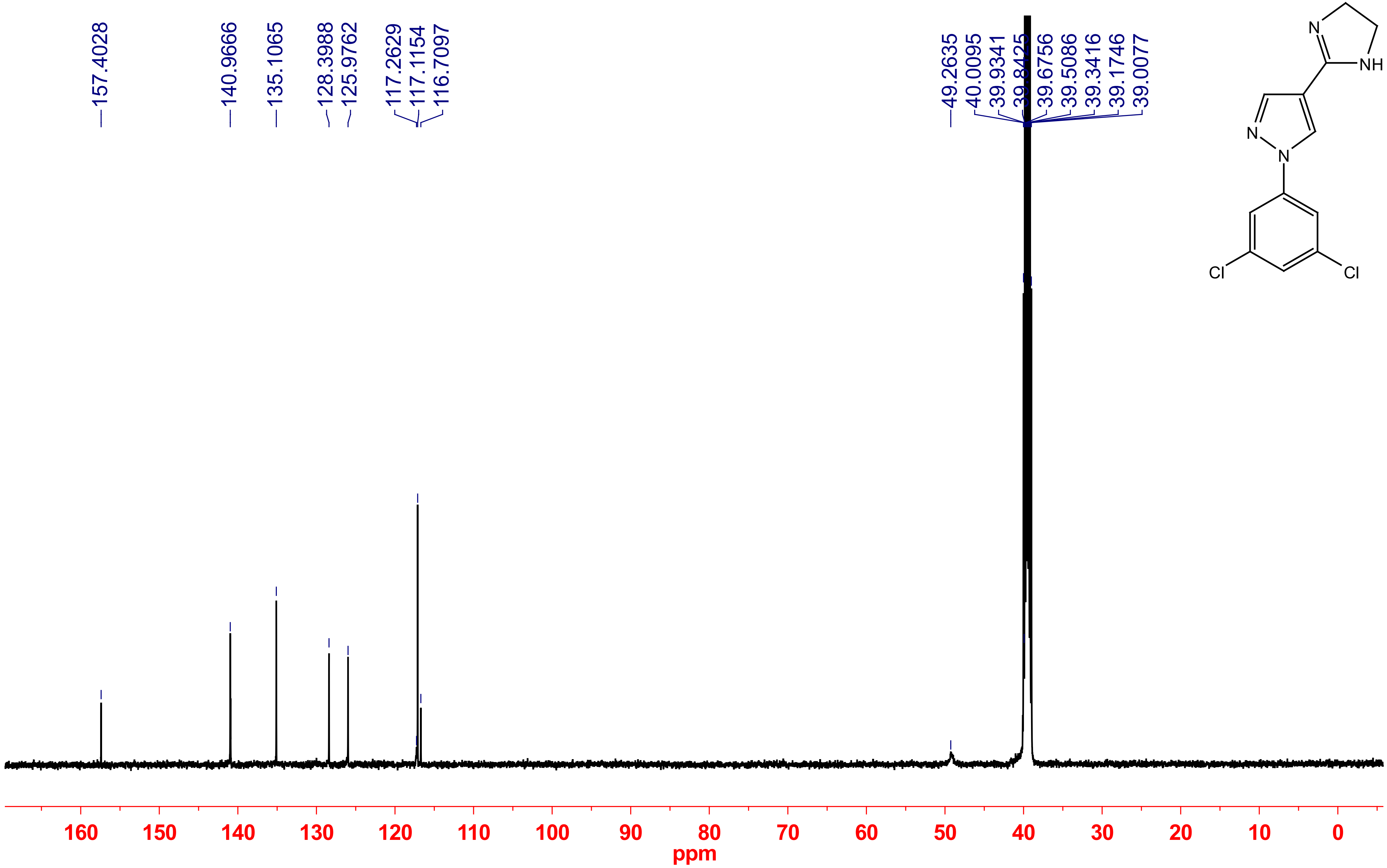

^{1}H NMR of compound **3e**

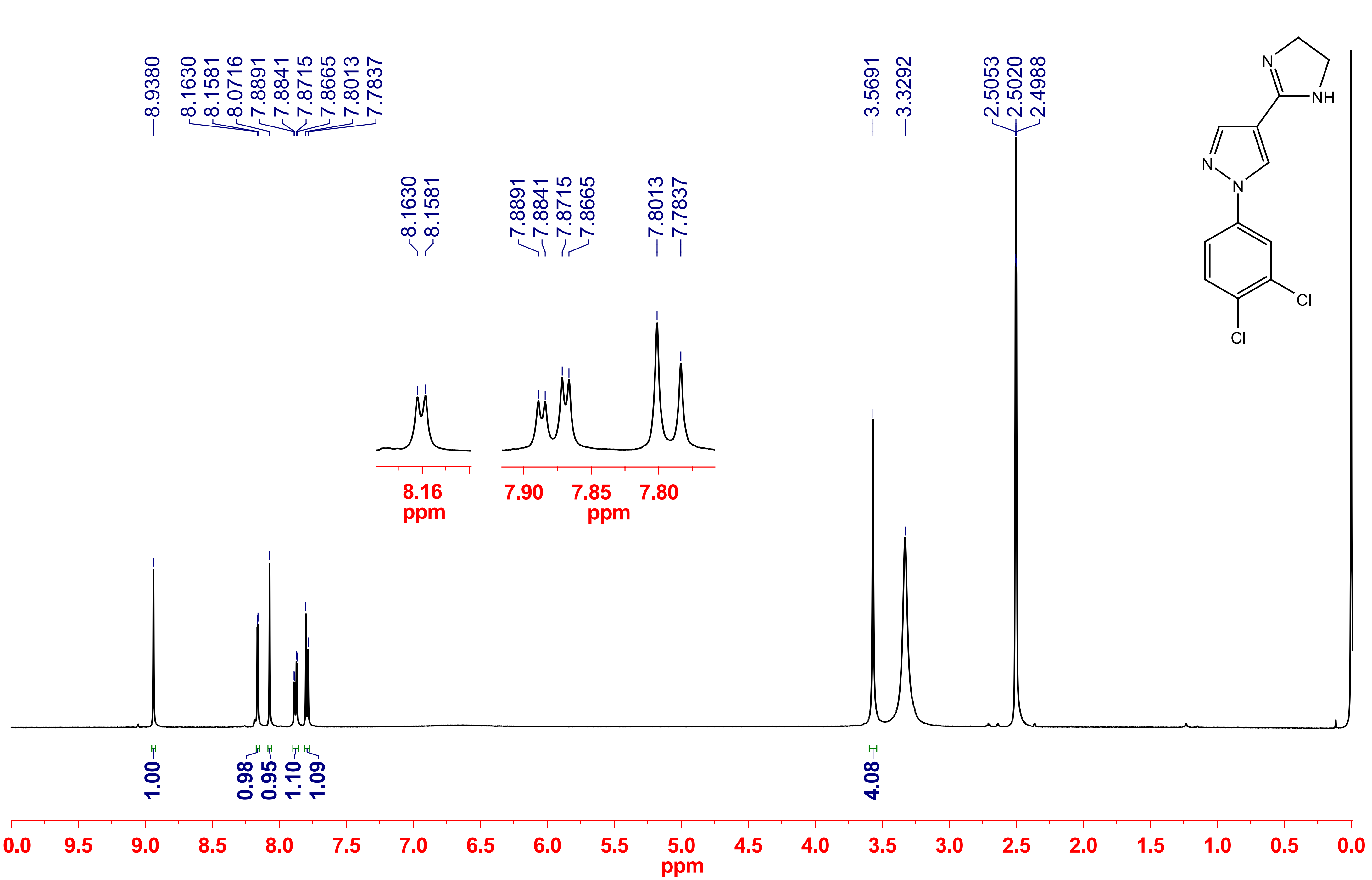

¹³C NMR of compound **3e**

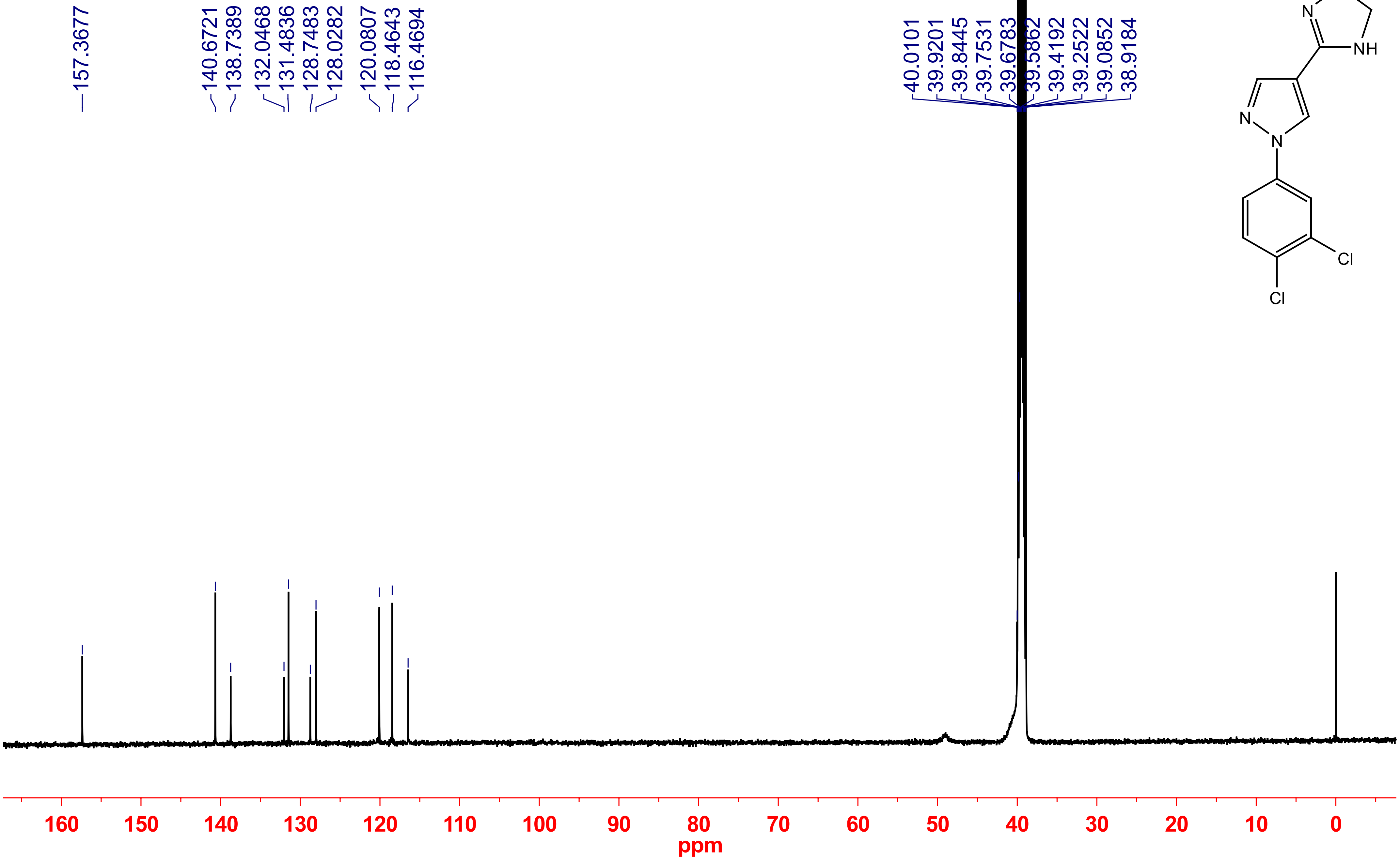

^{1}H NMR of compound **3f**

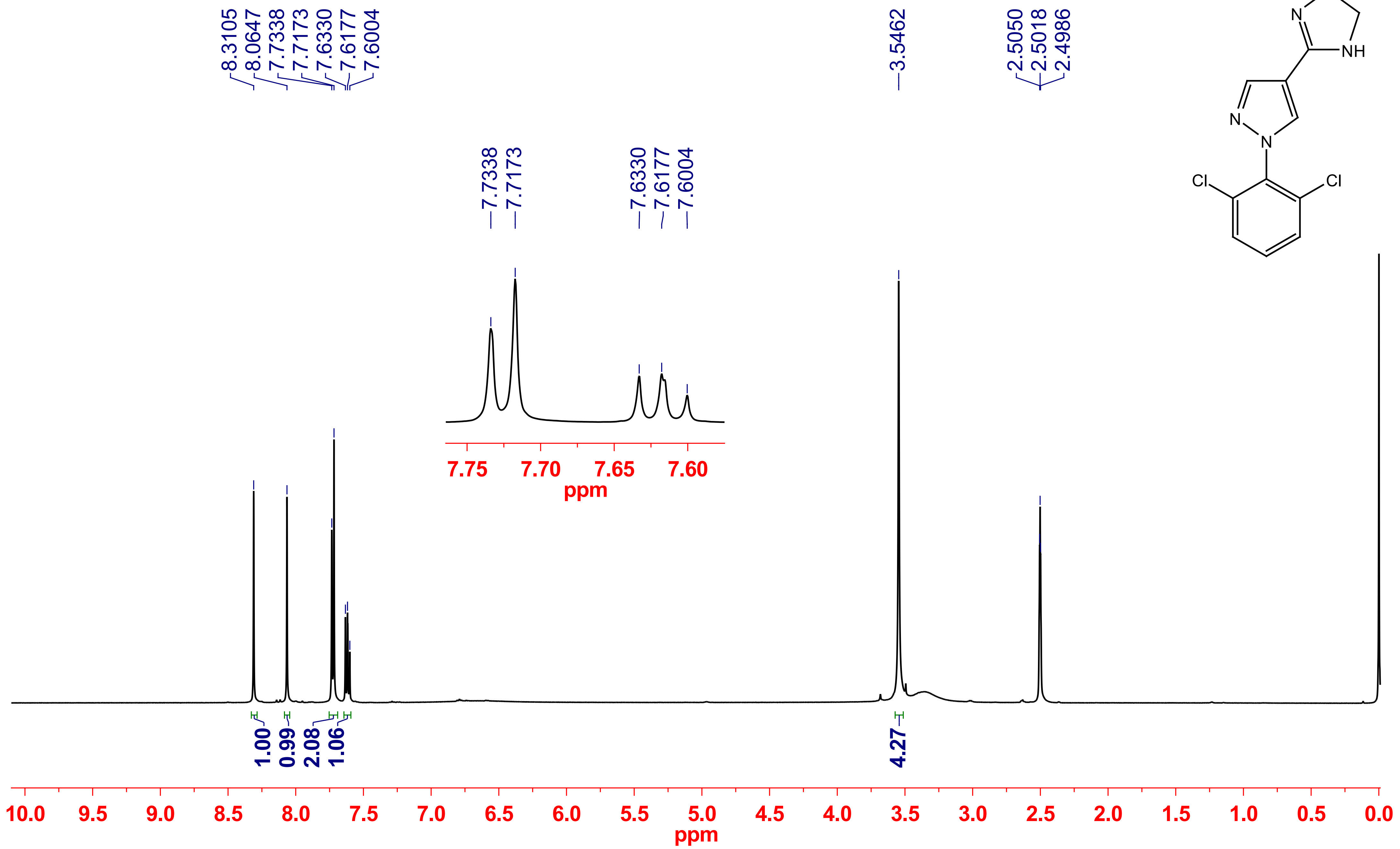

^{13}C NMR of compound **3f**

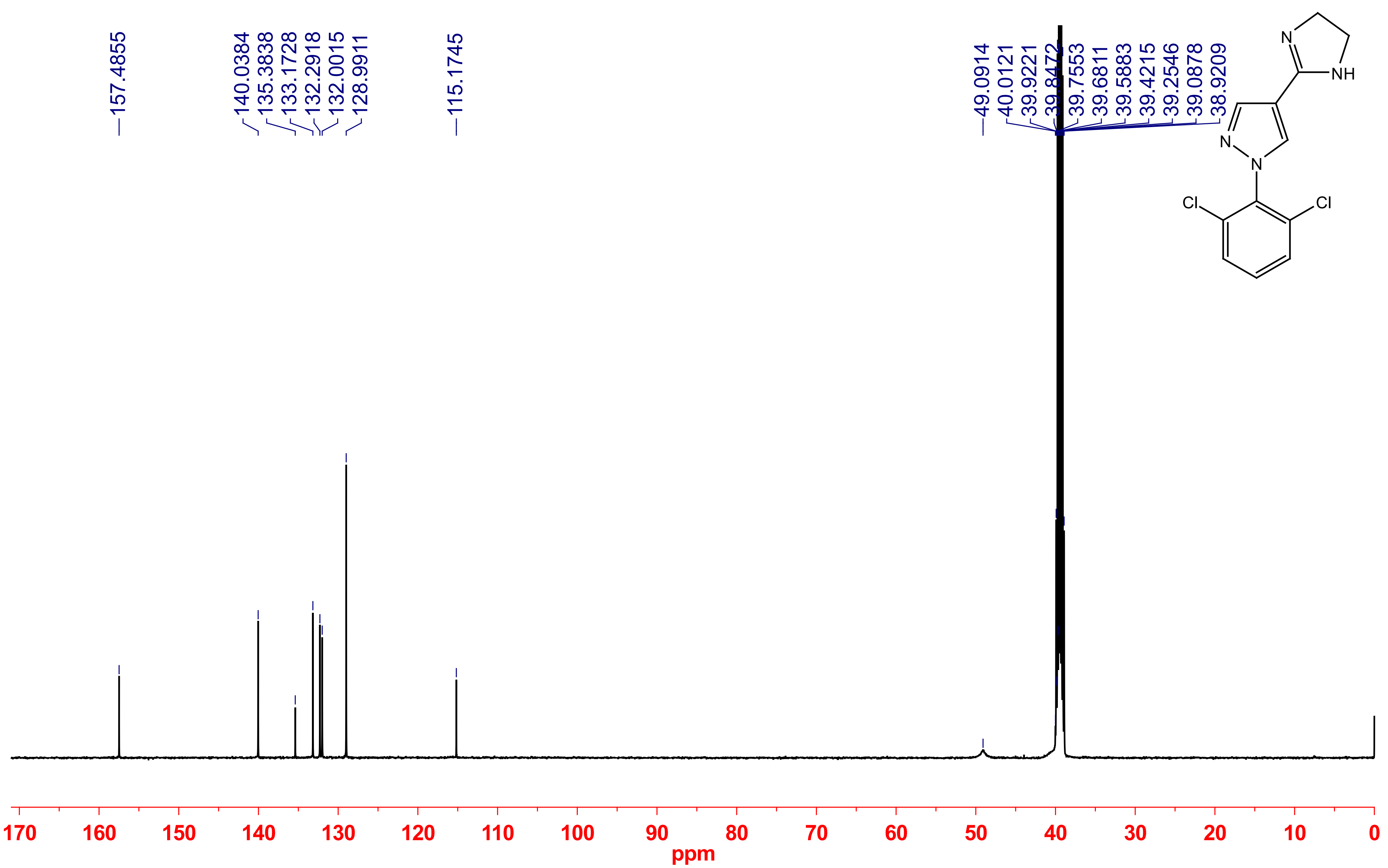

^{1}H NMR of compound **3g**

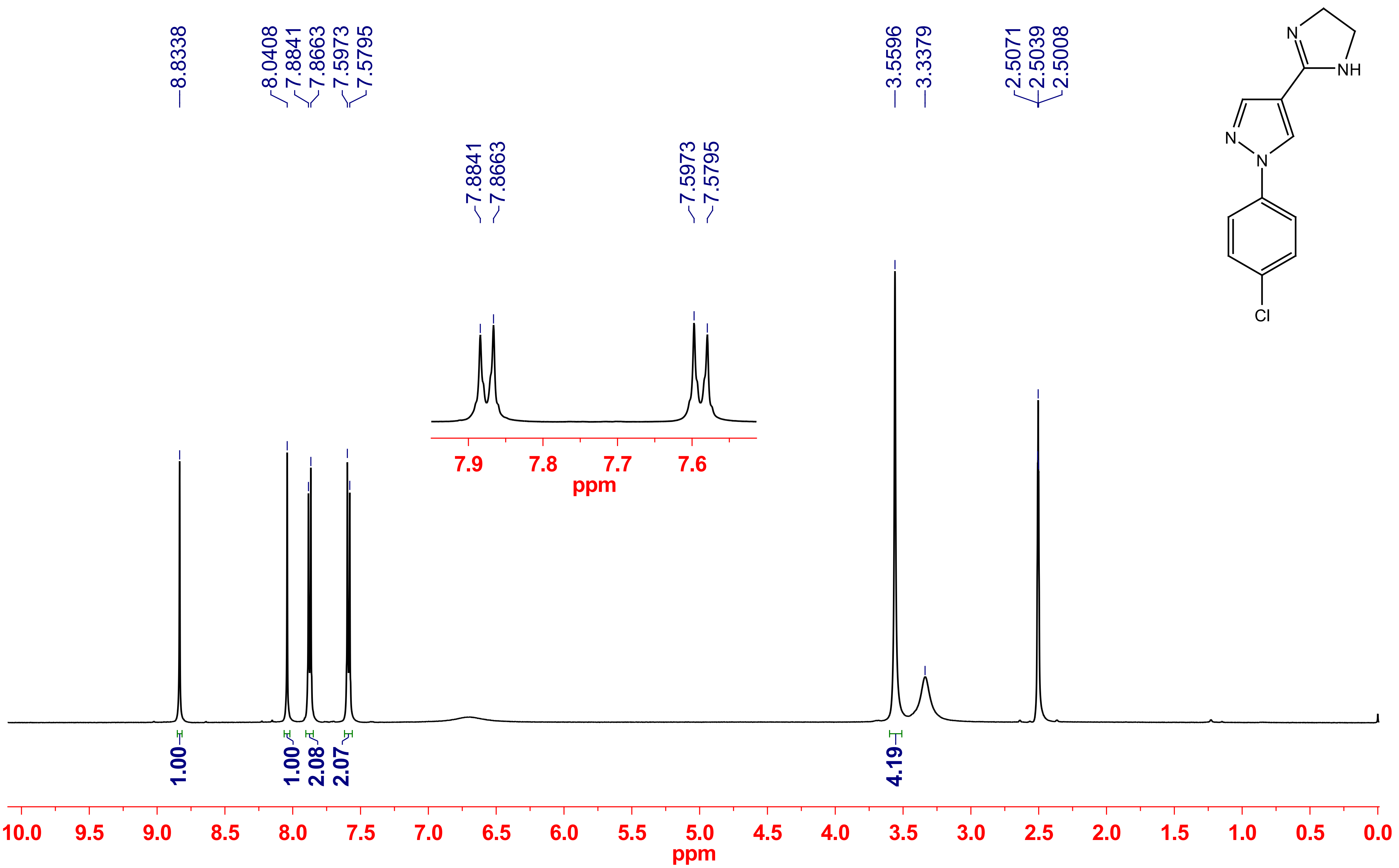

^{13}C NMR of compound **3g**

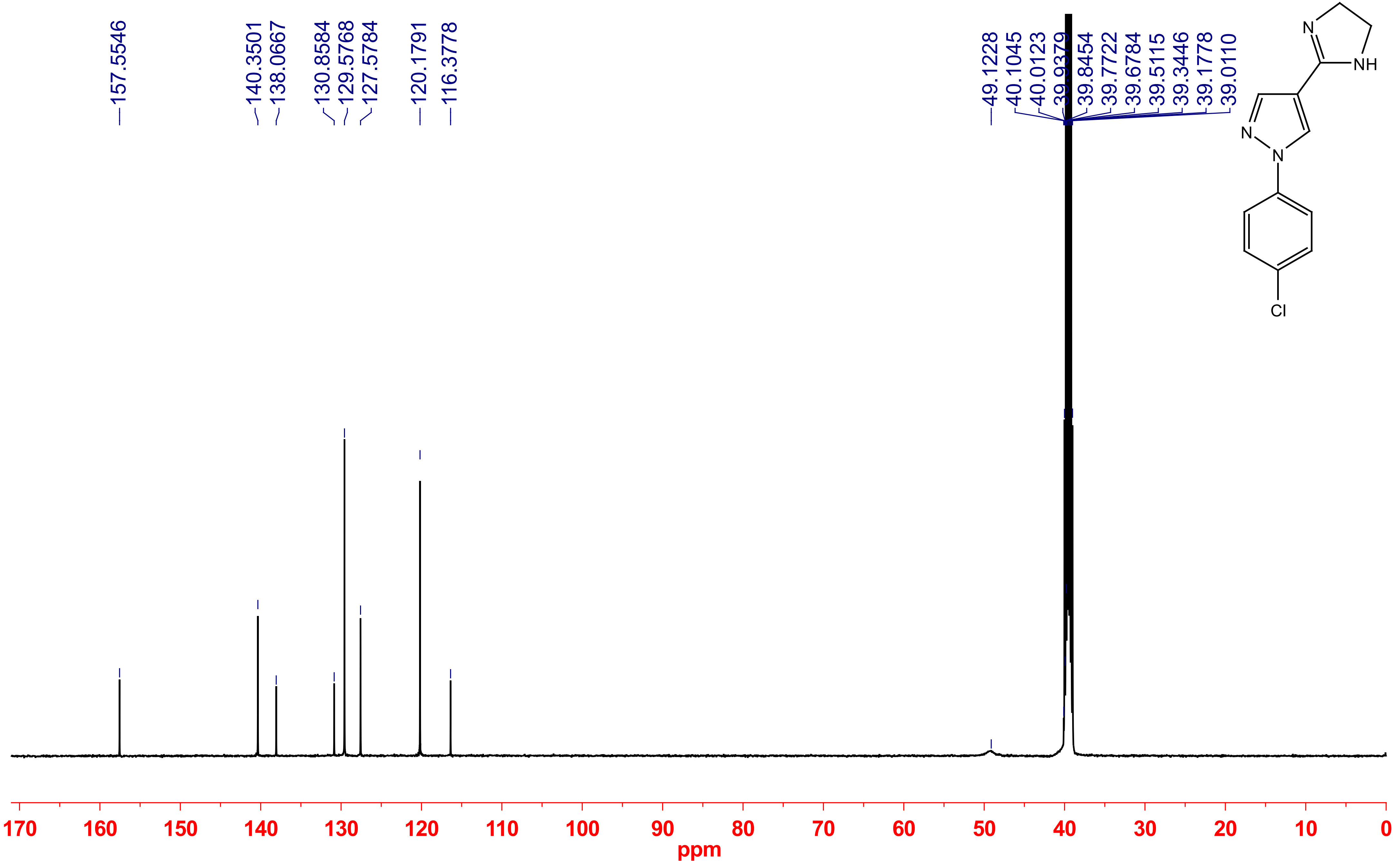

^1H NMR of compound **3h**

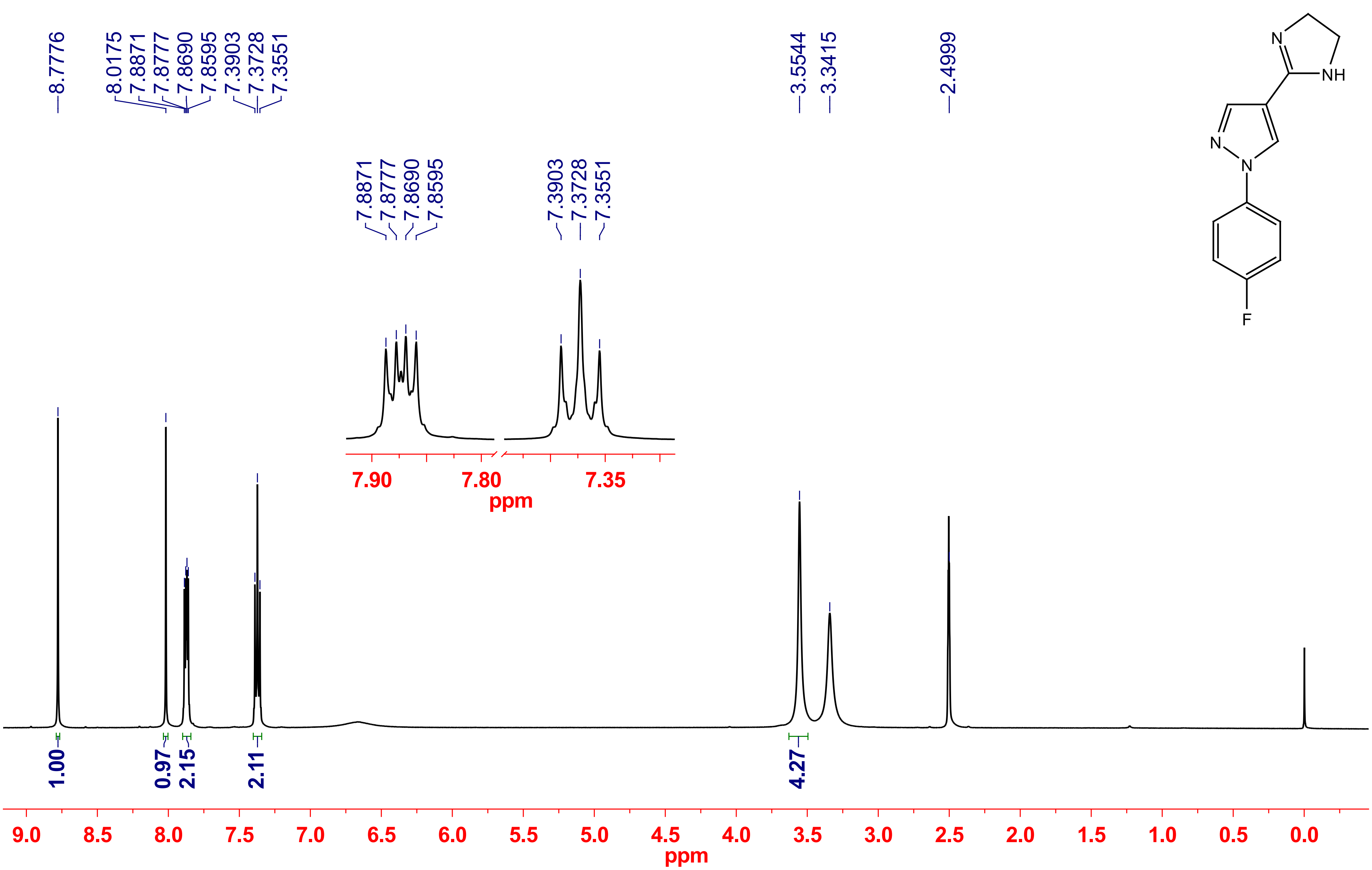

^{13}C NMR of compound **3h**

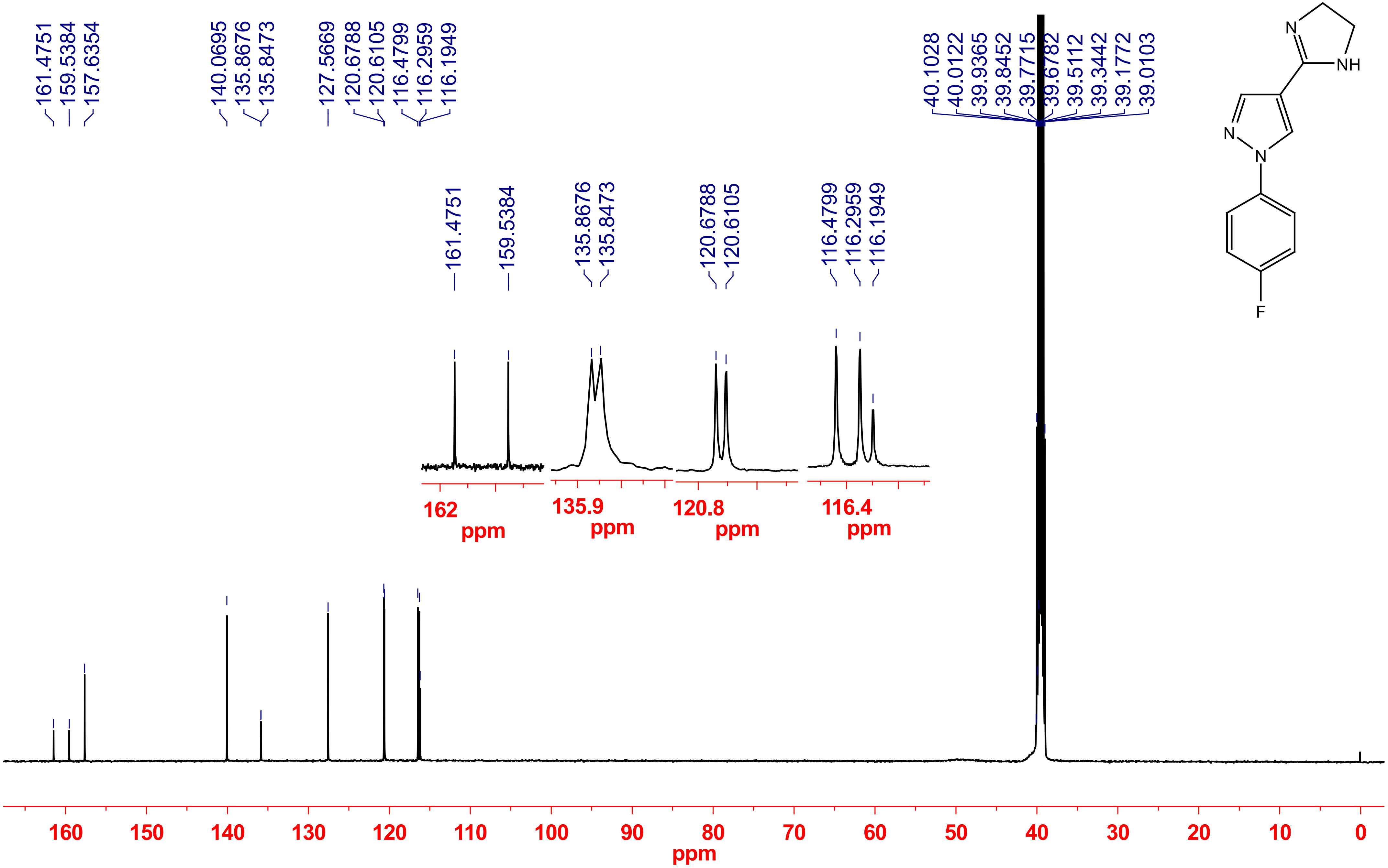

^{1}H NMR of compound **3i**

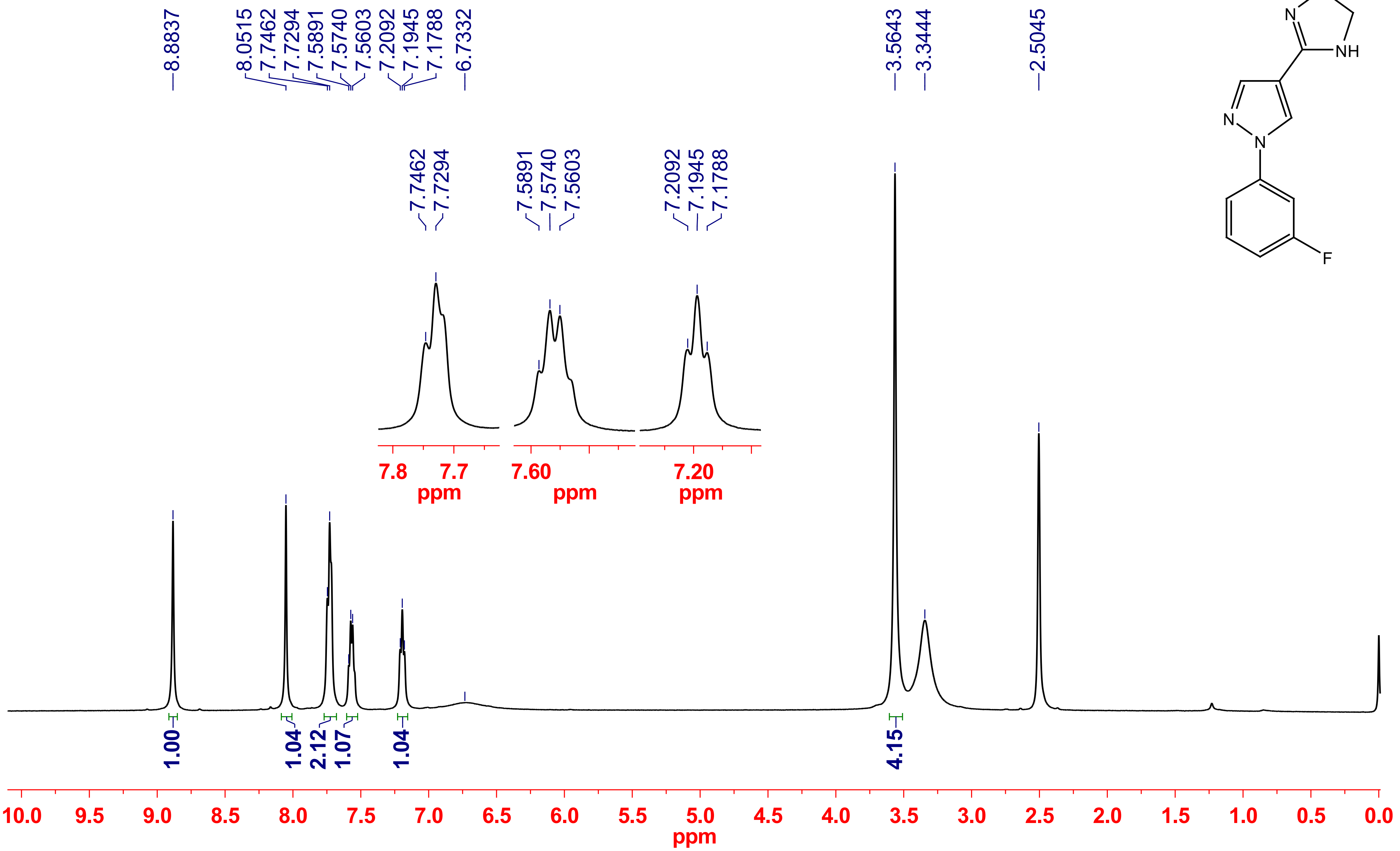

^{13}C NMR of compound **3i**

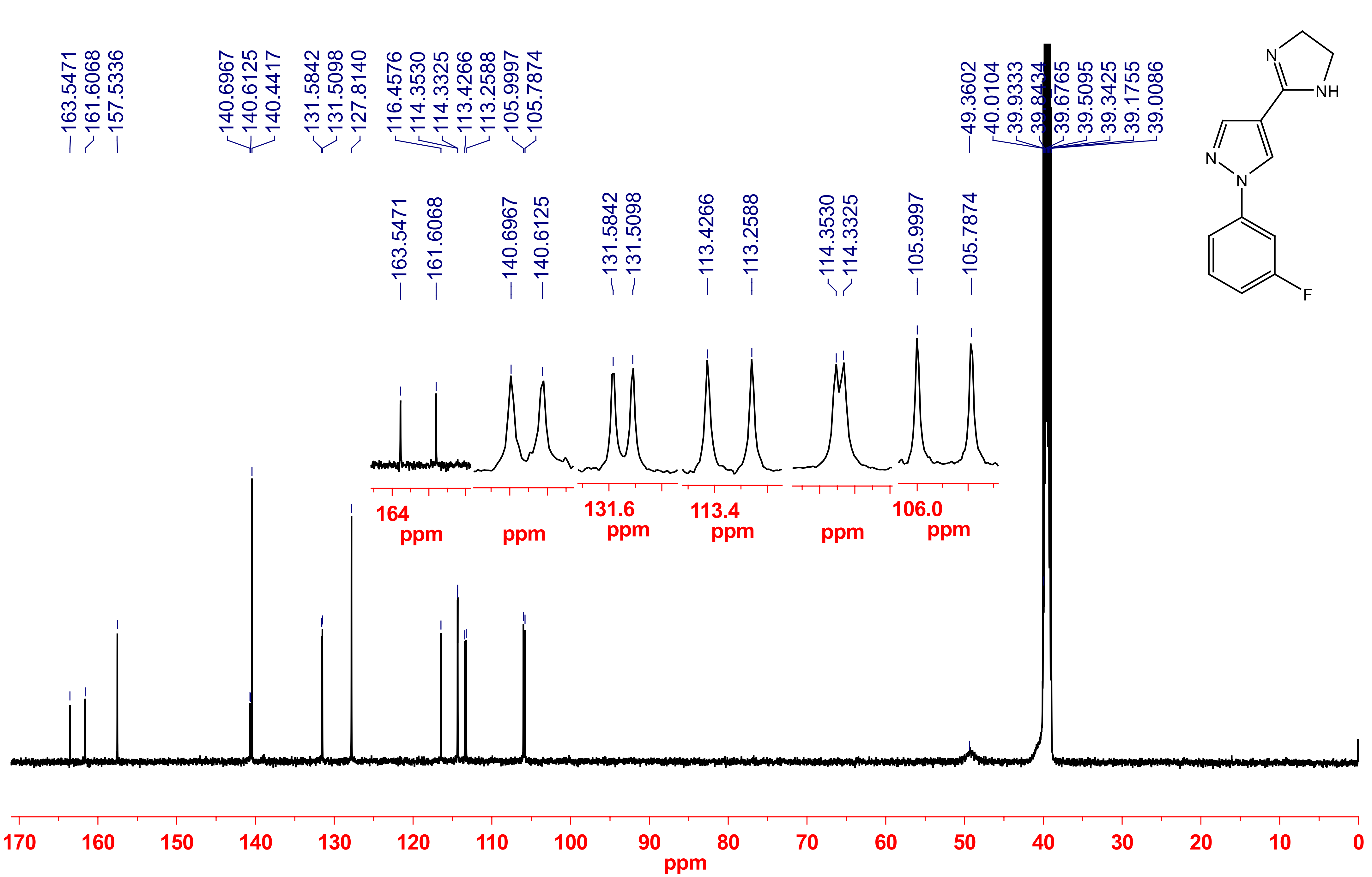

^{1}H NMR of compound **3j**

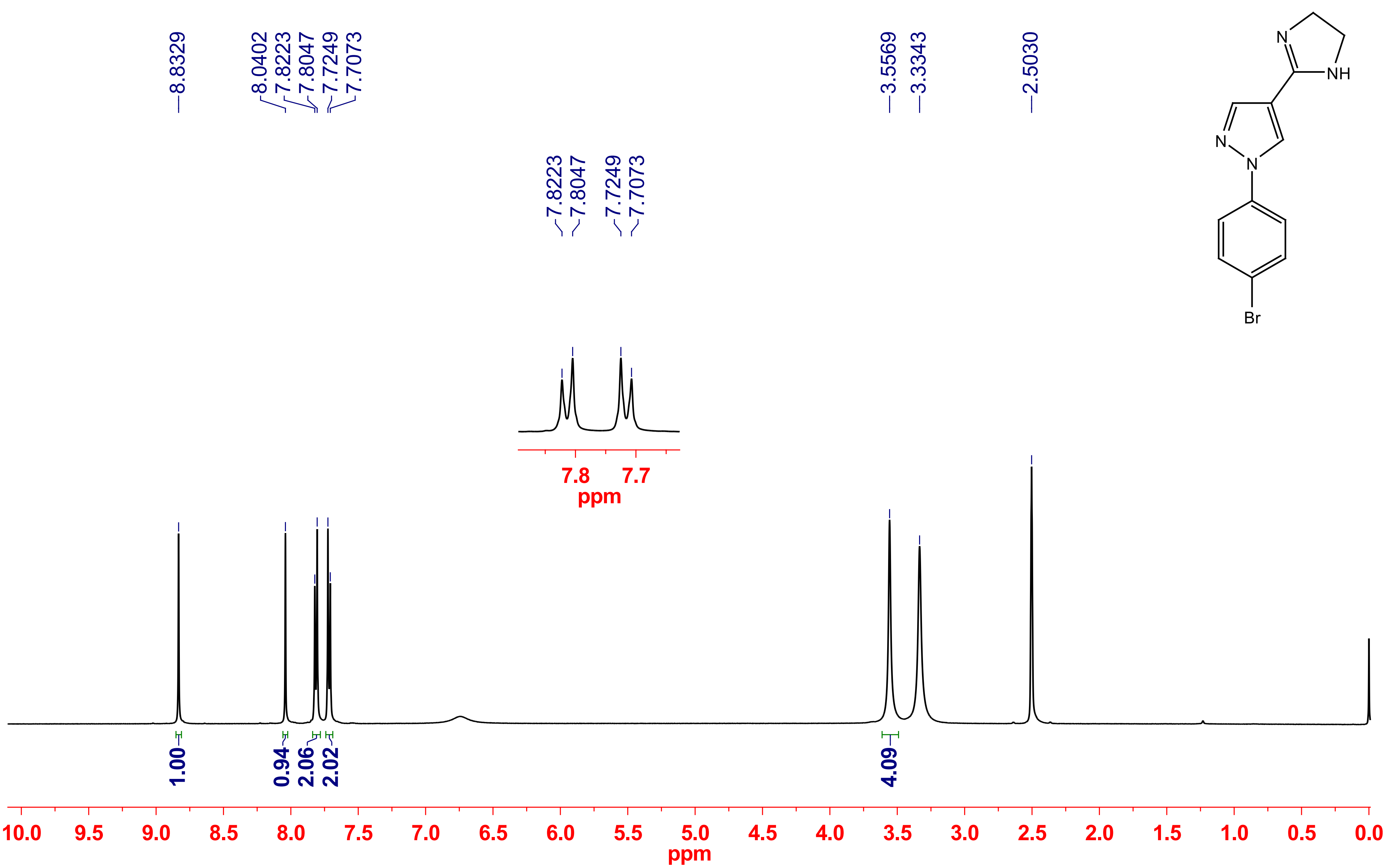

^{13}C NMR of compound **3j**

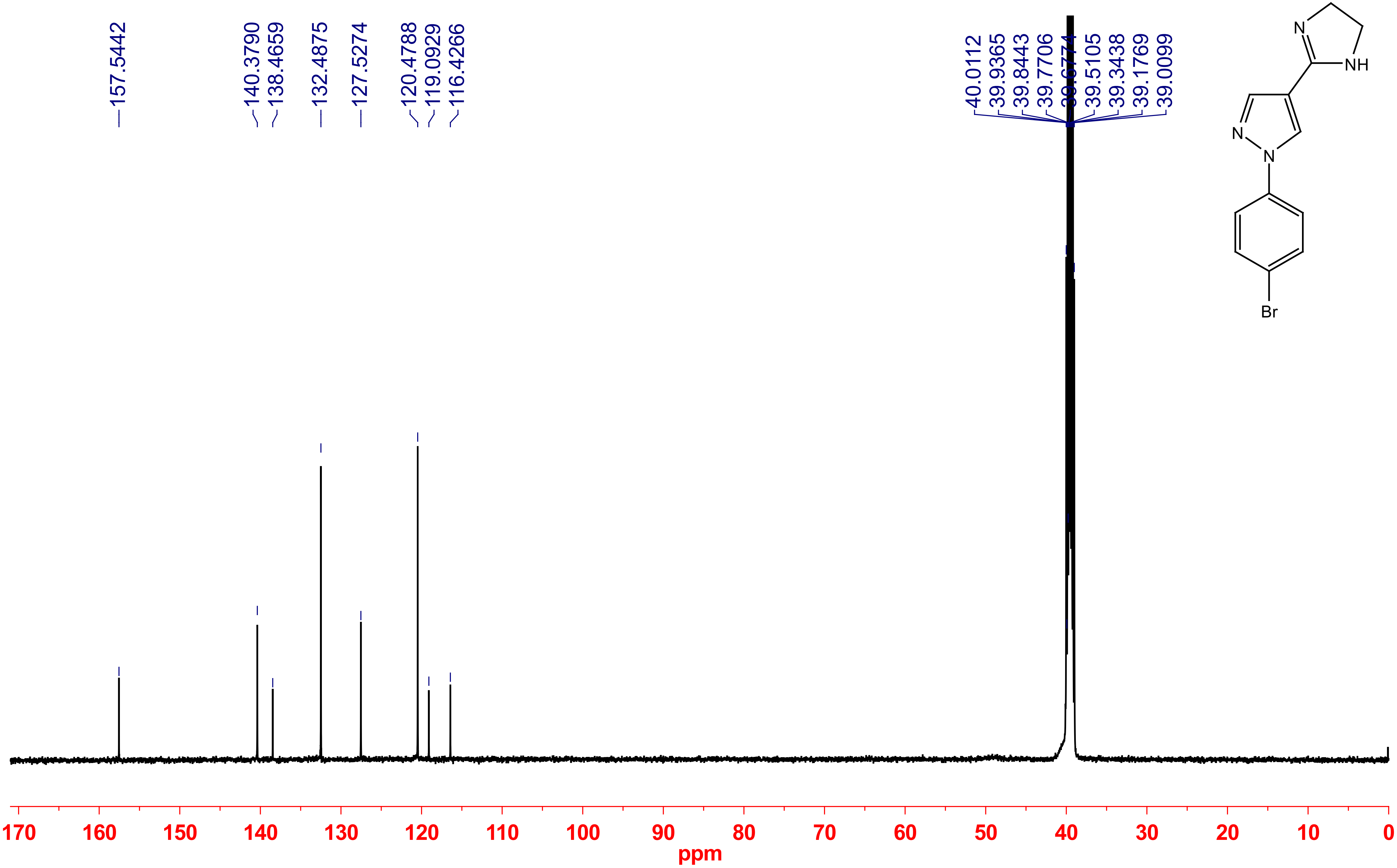

^{1}H NMR of compound **3k**

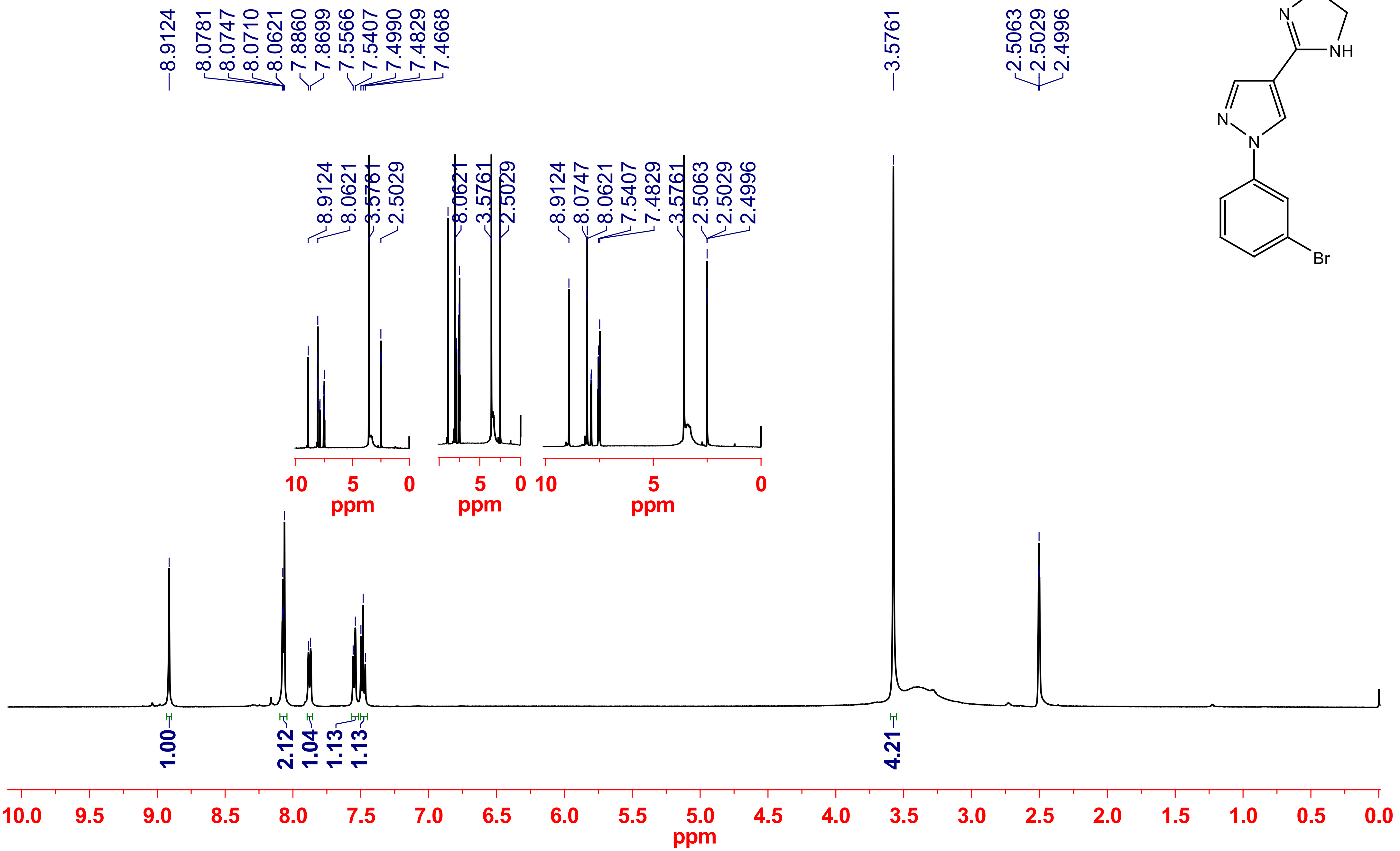

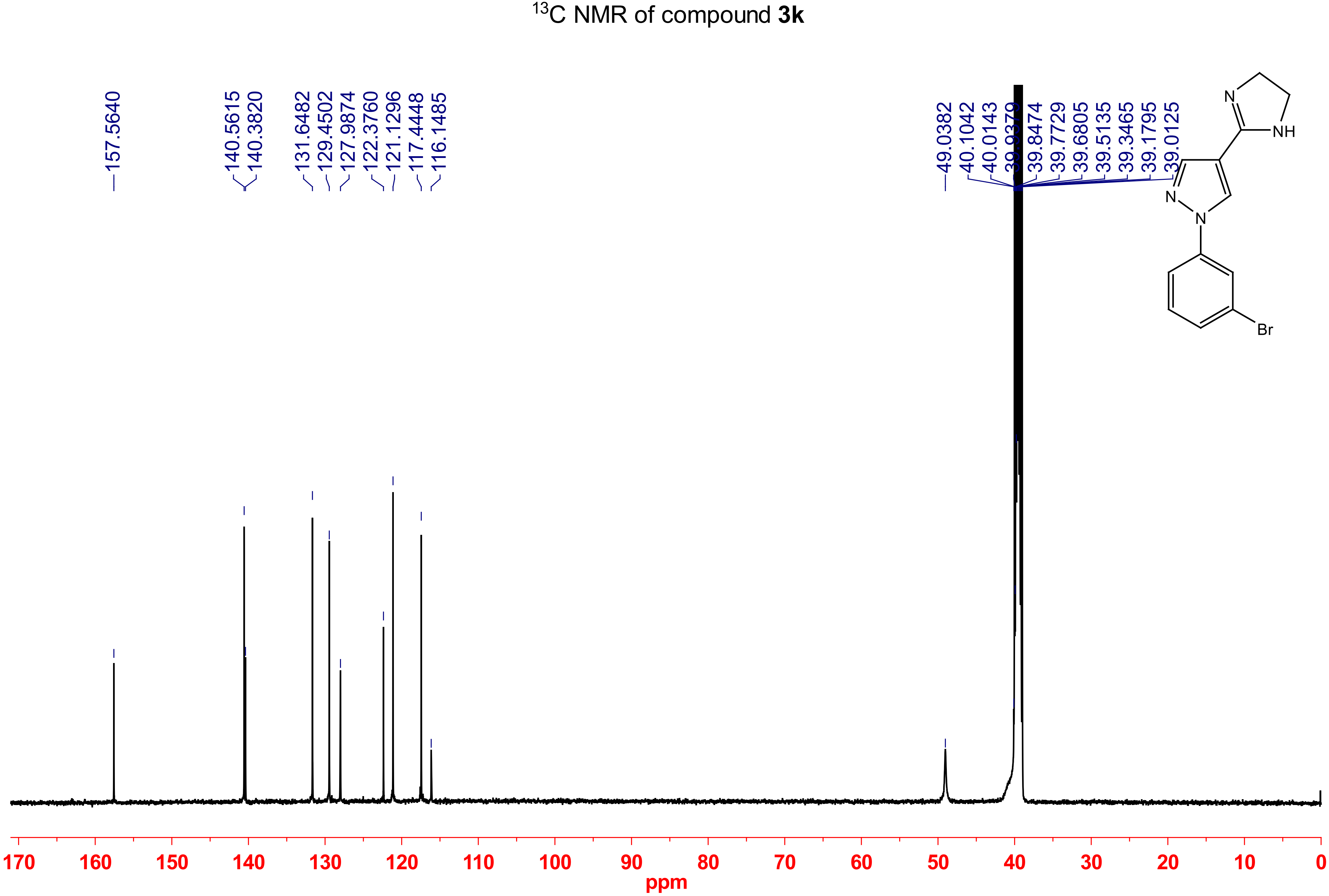

^{13}C NMR of compound **3k**
157.5640
140.5615
140.3820
131.6482
129.4502
127.9874
122.3760
121.1296
117.4448
116.1485
49.0382
40.1042
40.0143
39.9379
39.8474
39.7729
39.6805
39.5135
39.3465
39.1795
39.0125
N
NH
N
N
Br
170
160
150
140
130
120
110
100
90
80
70
60
50
40
30
20
10
0
ppm

^{1}H NMR of compound **3l**

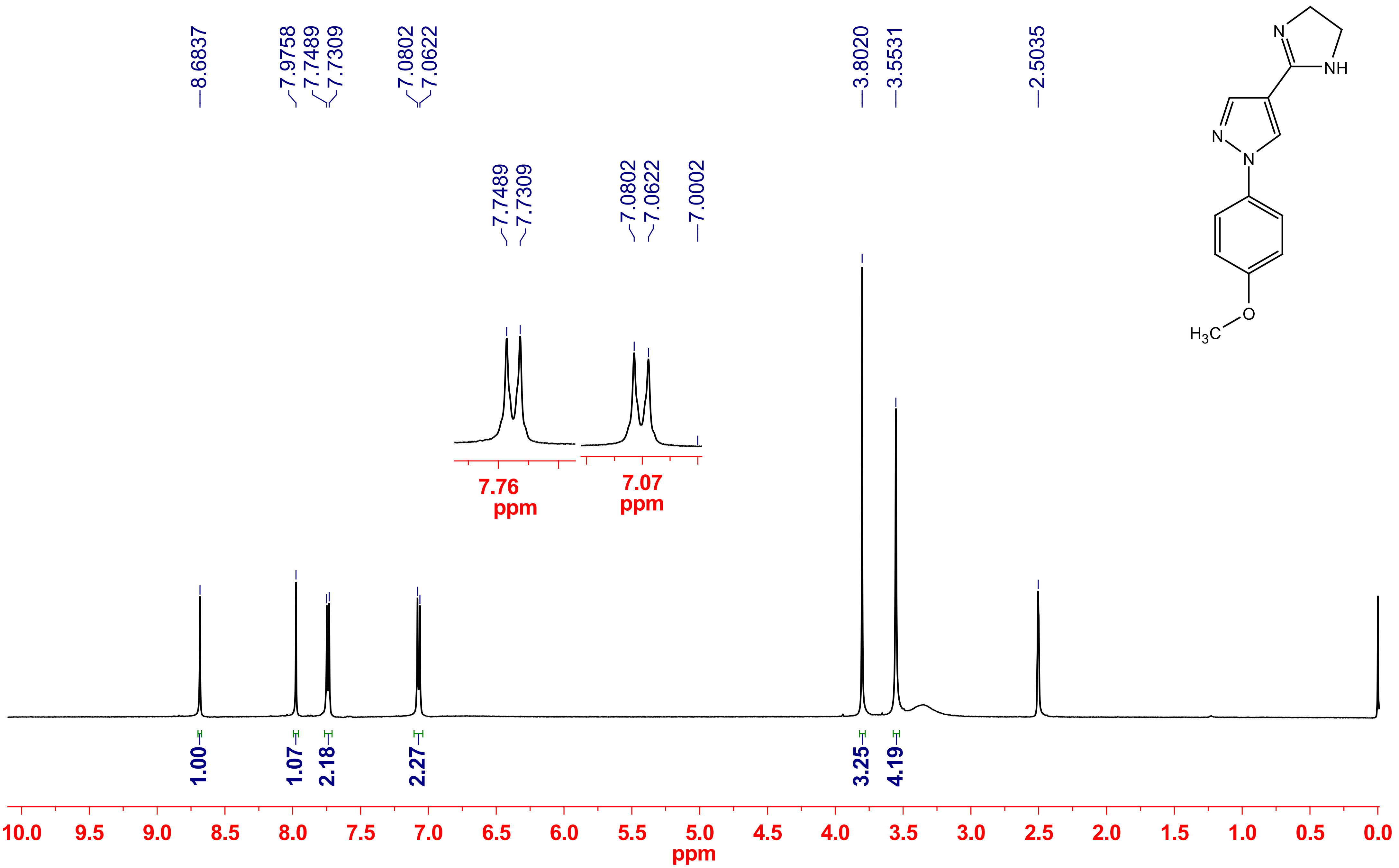

^{13}C NMR of compound **3l**

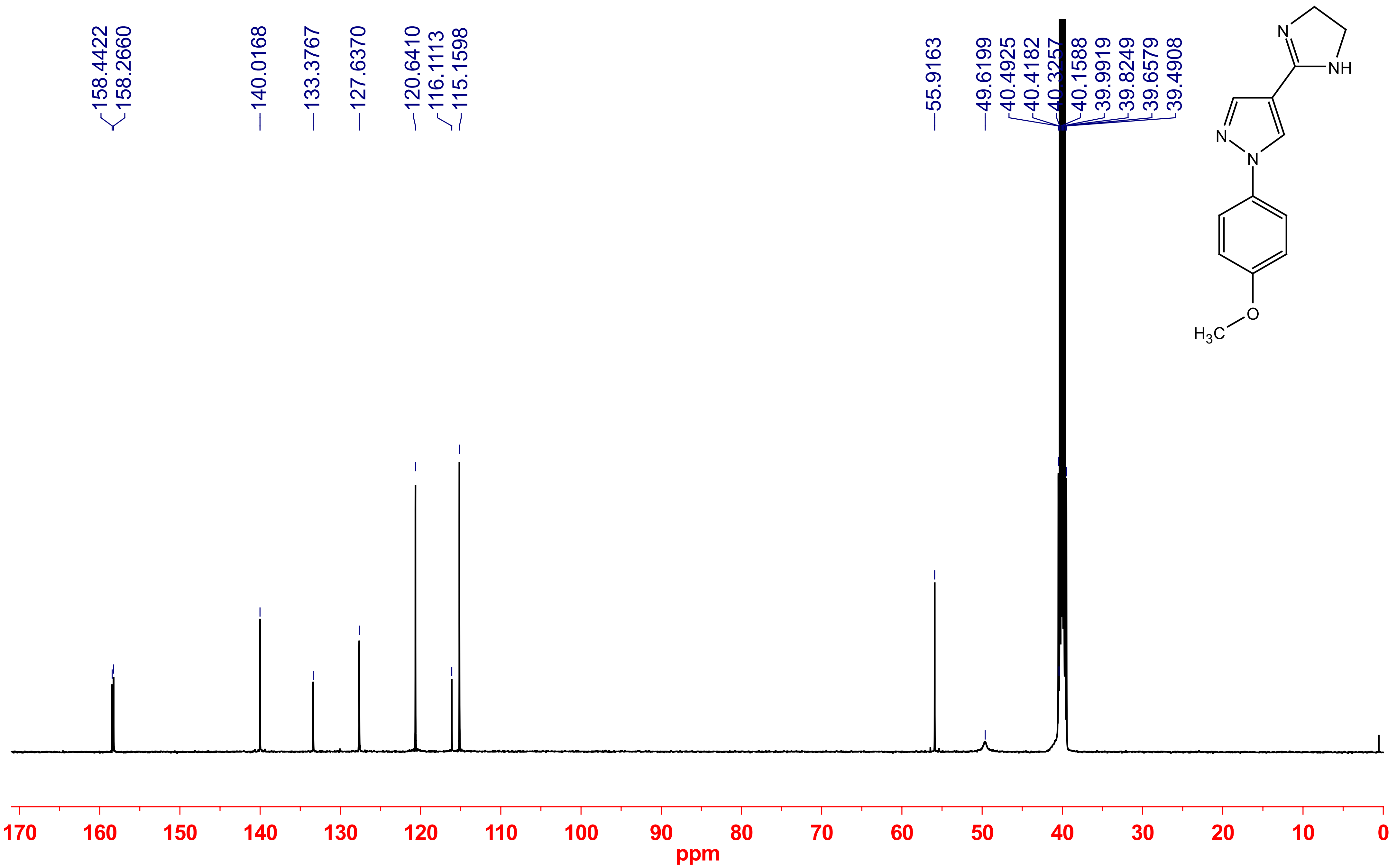

^{1}H NMR of compound **3m**

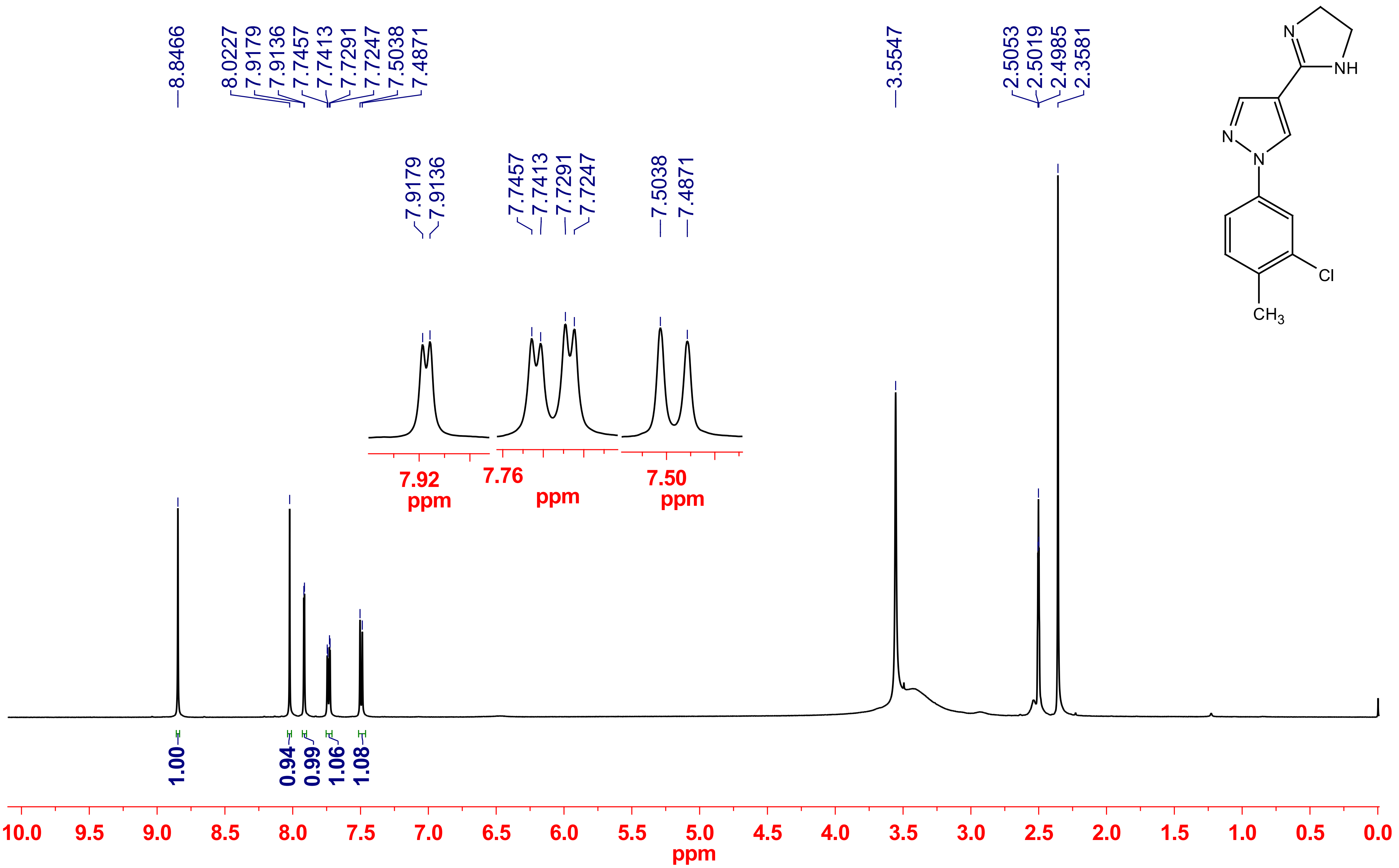

^{13}C NMR of compound **3m**

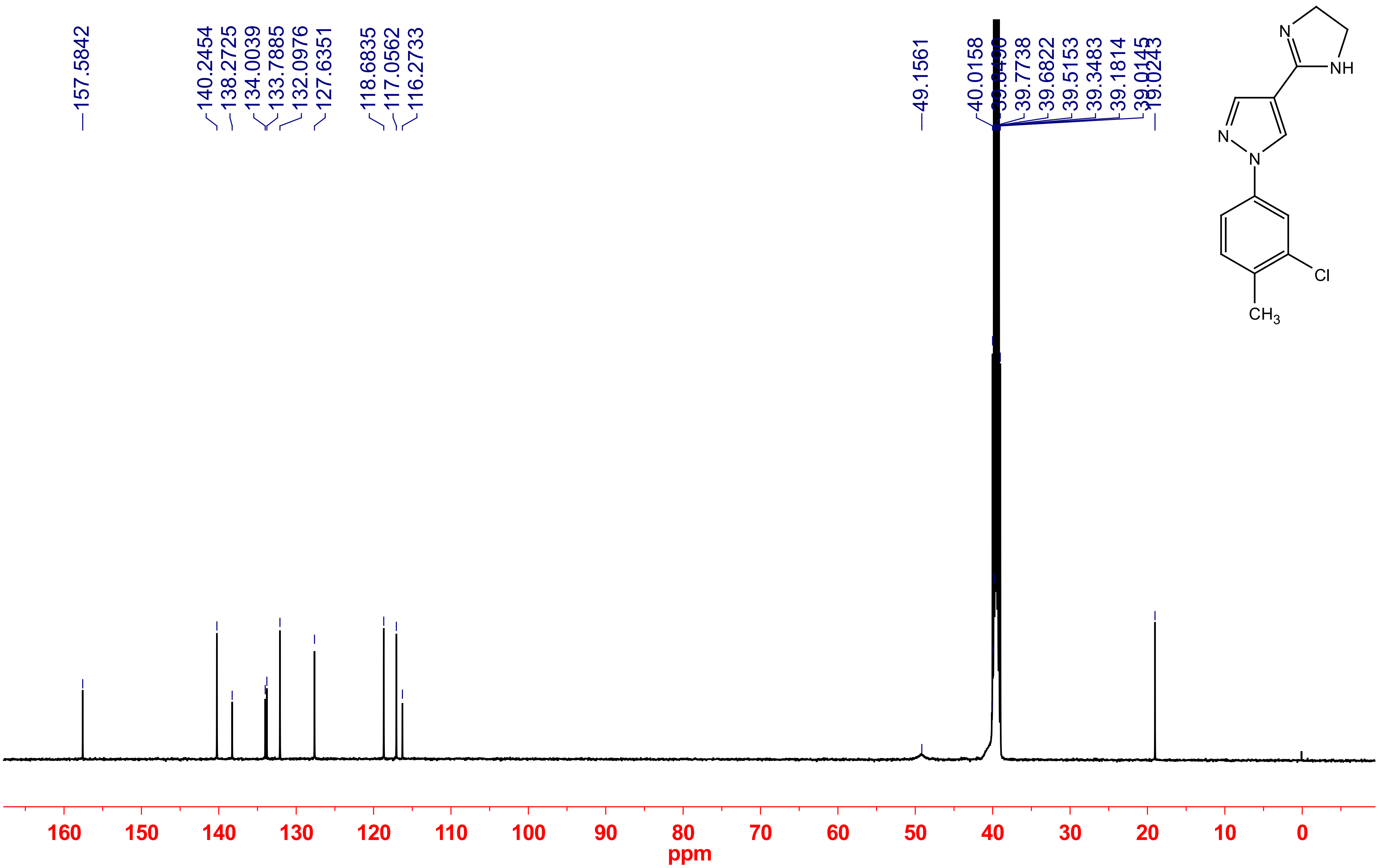

^{1}H NMR of compound **3n**

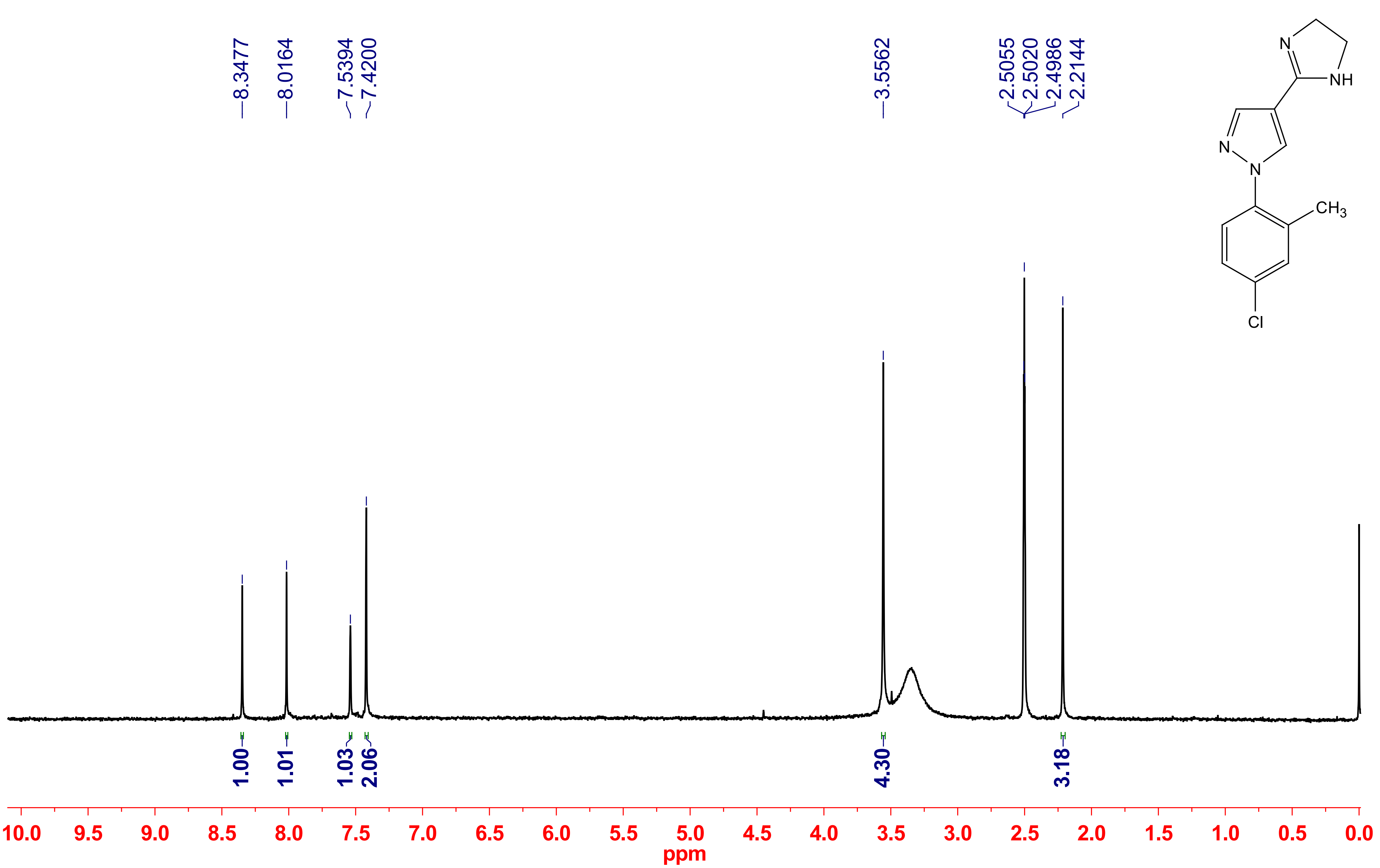

^{13}C NMR of compound **3n**

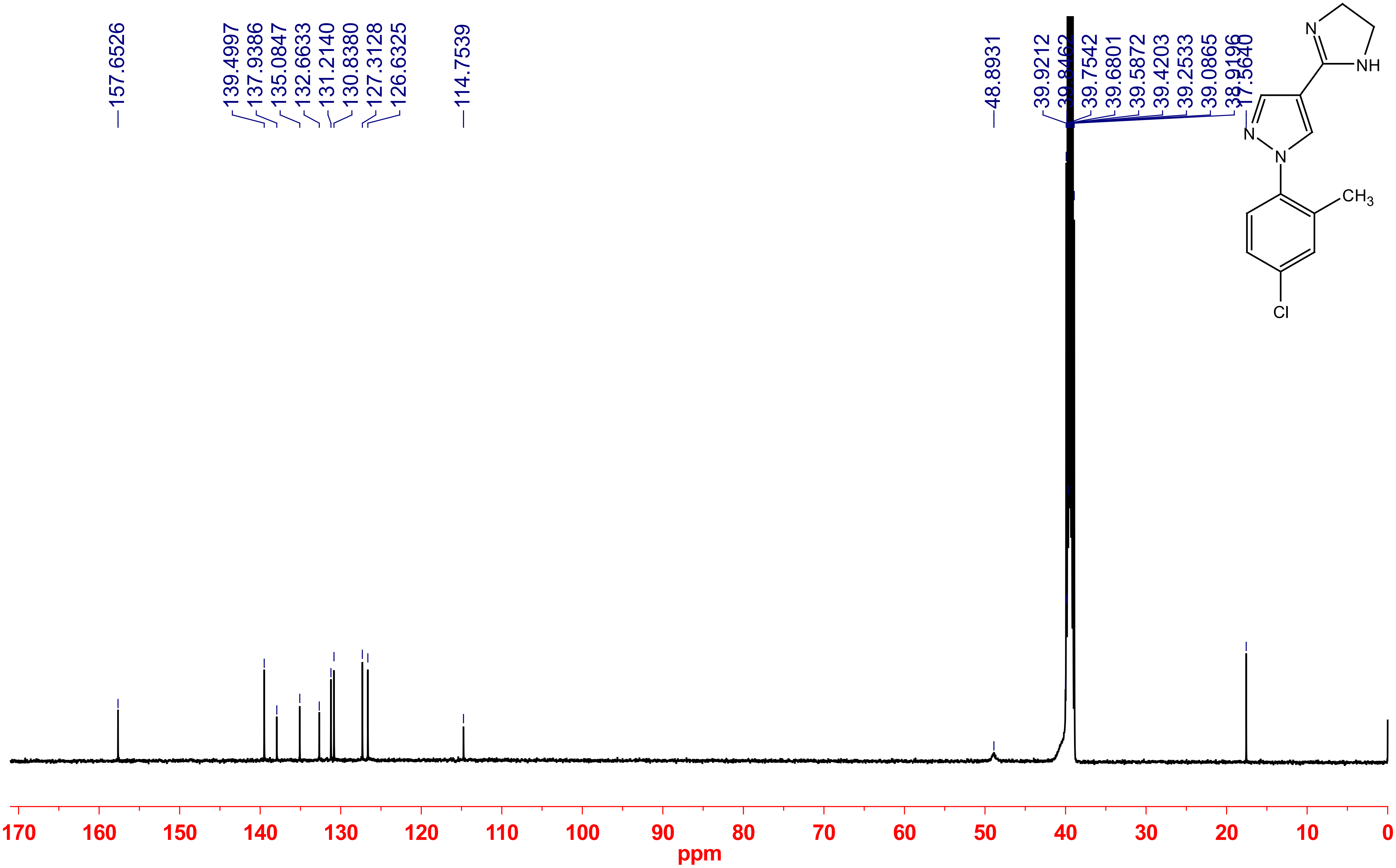

^{1}H NMR of compound **4a**

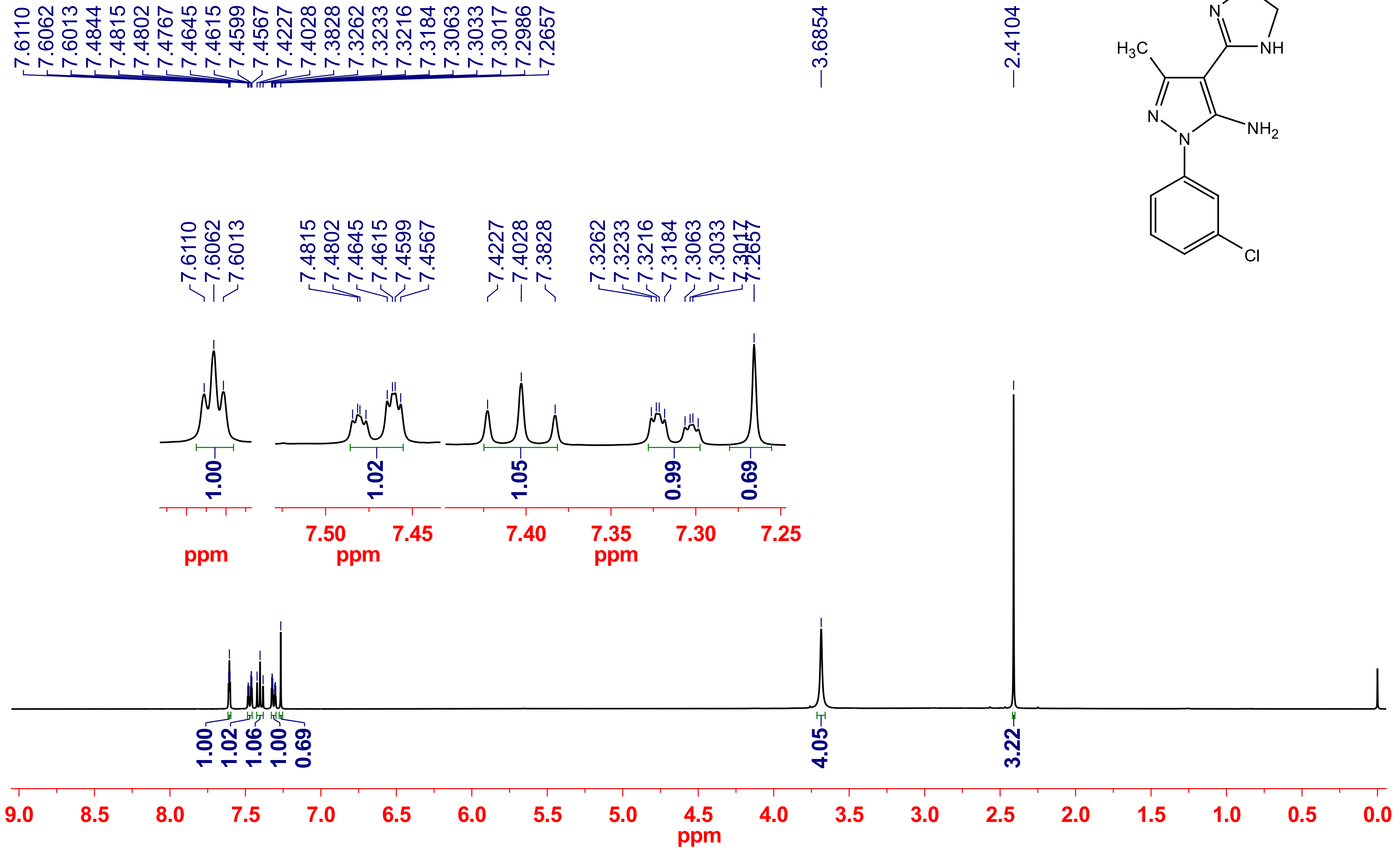

^{13}C NMR of compound **4a**

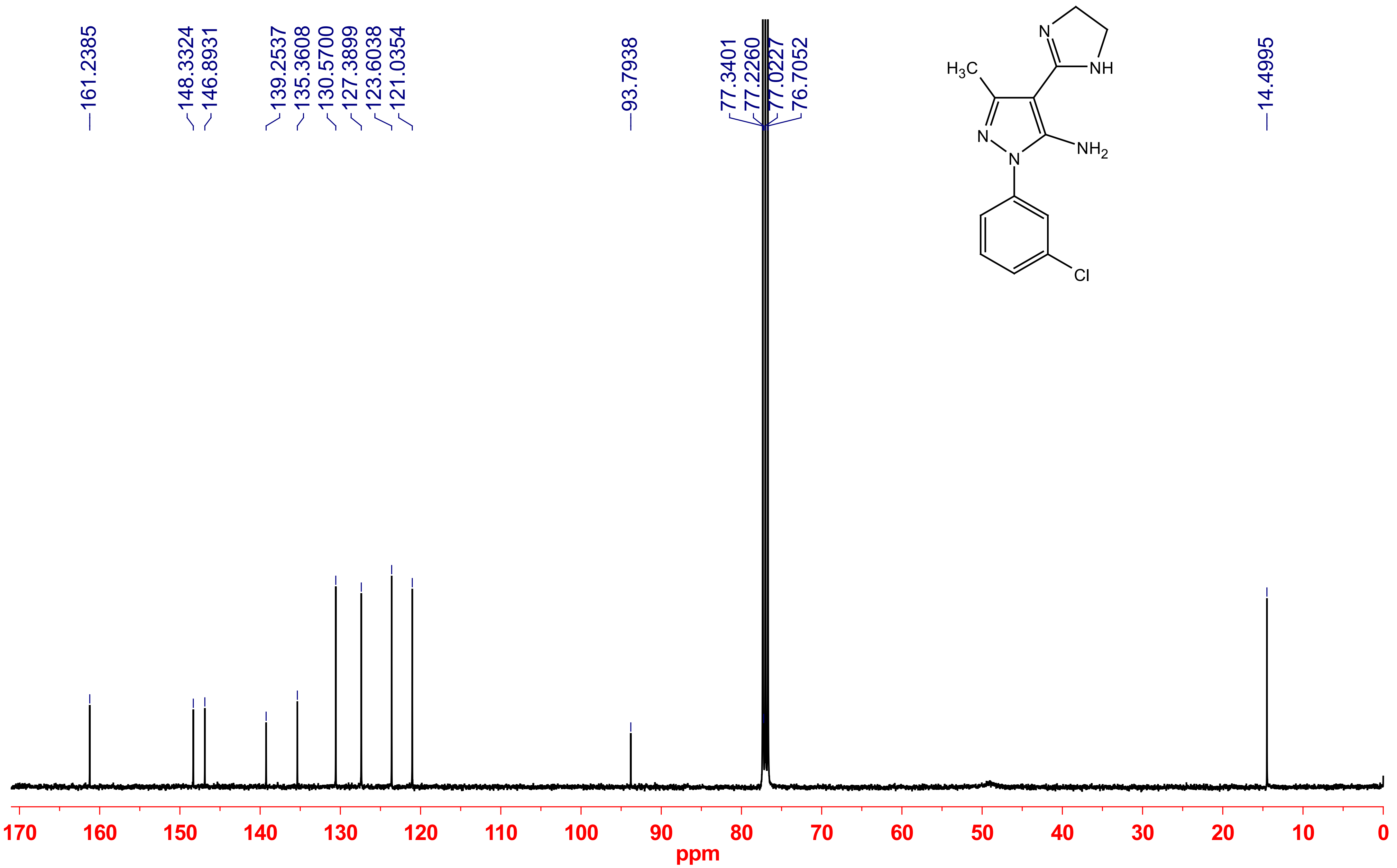

^{1}H NMR of compound **4b**

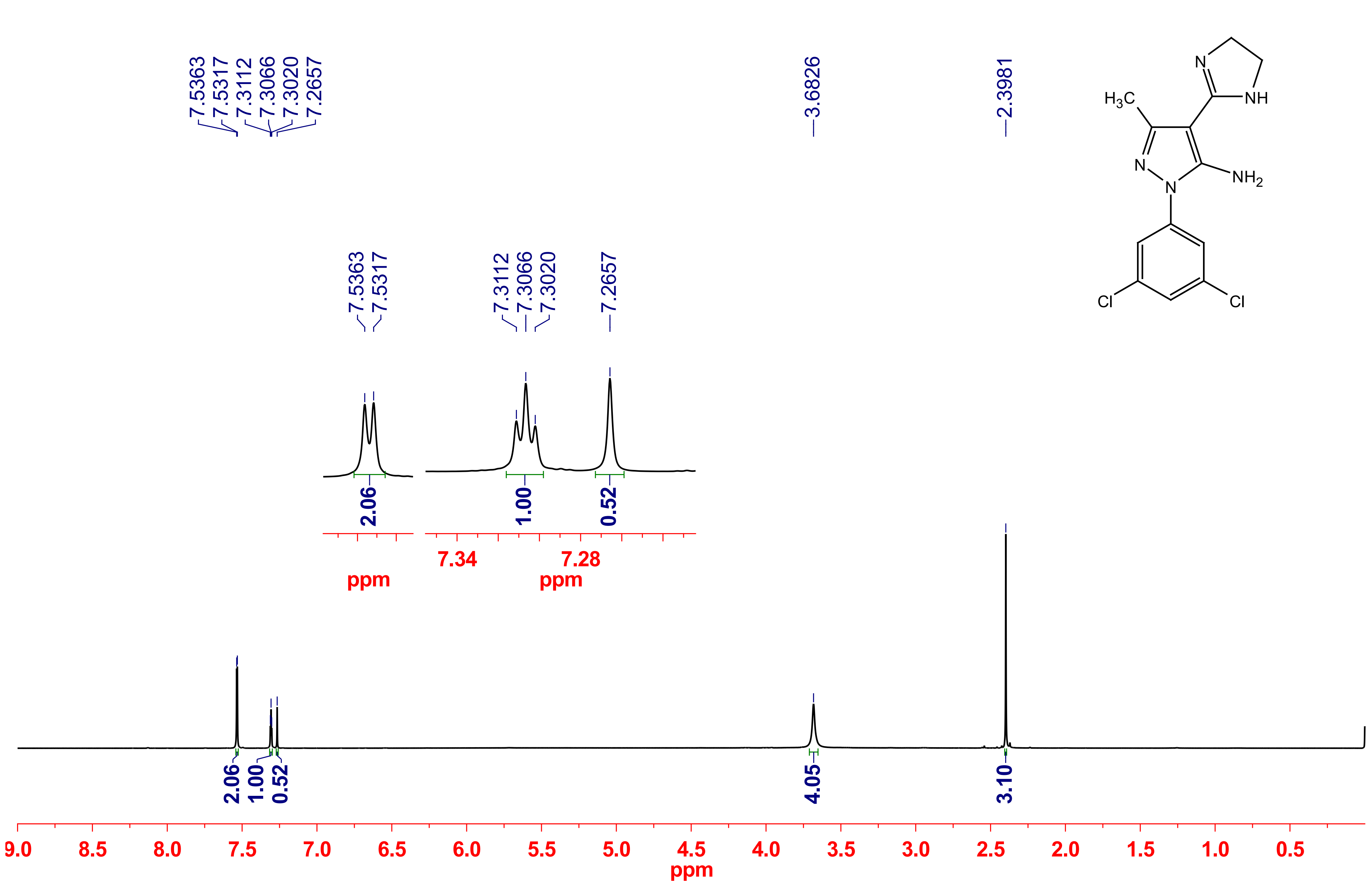

^{13}C NMR of compound **4b**

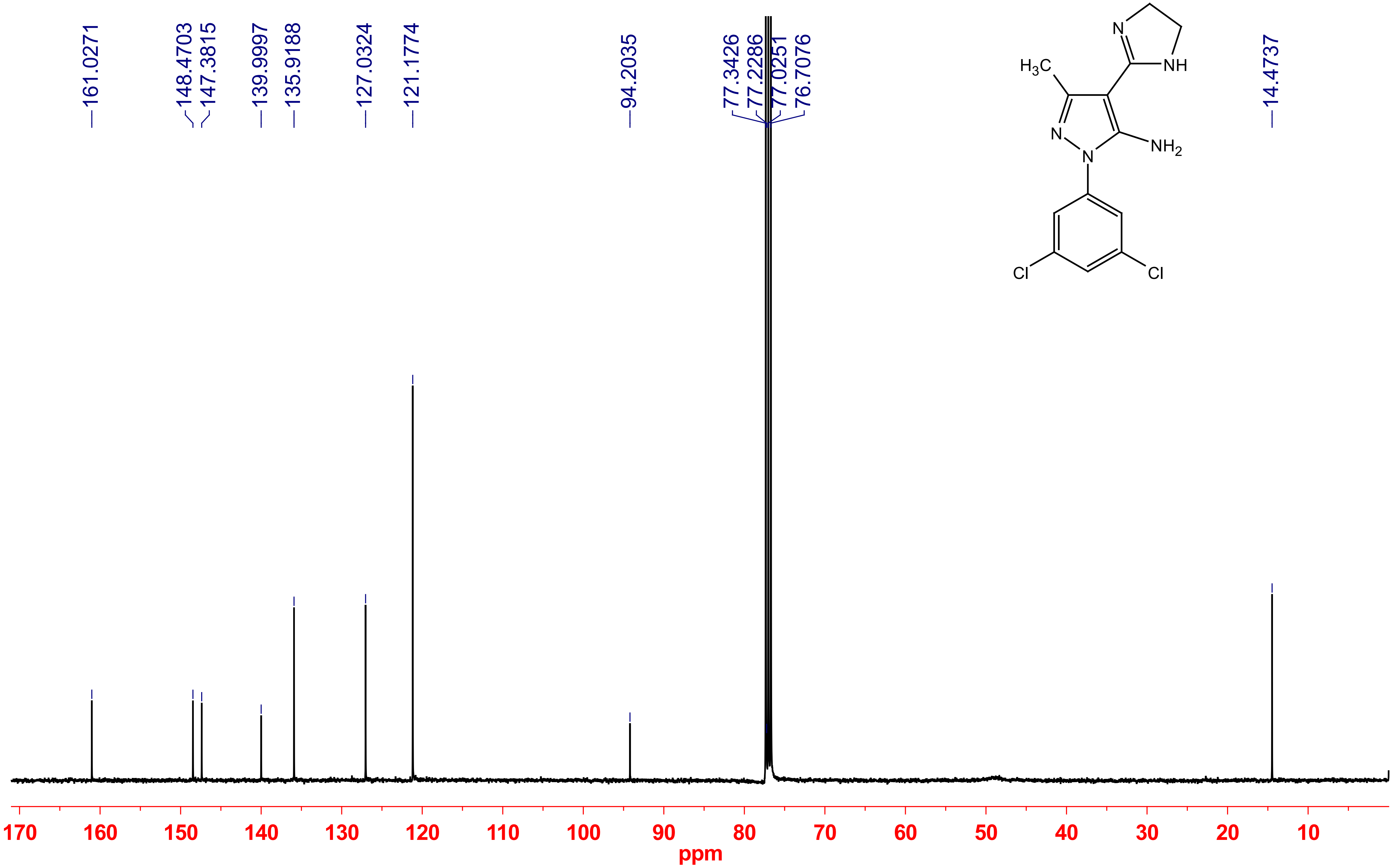

^{1}H NMR of compound **4c**

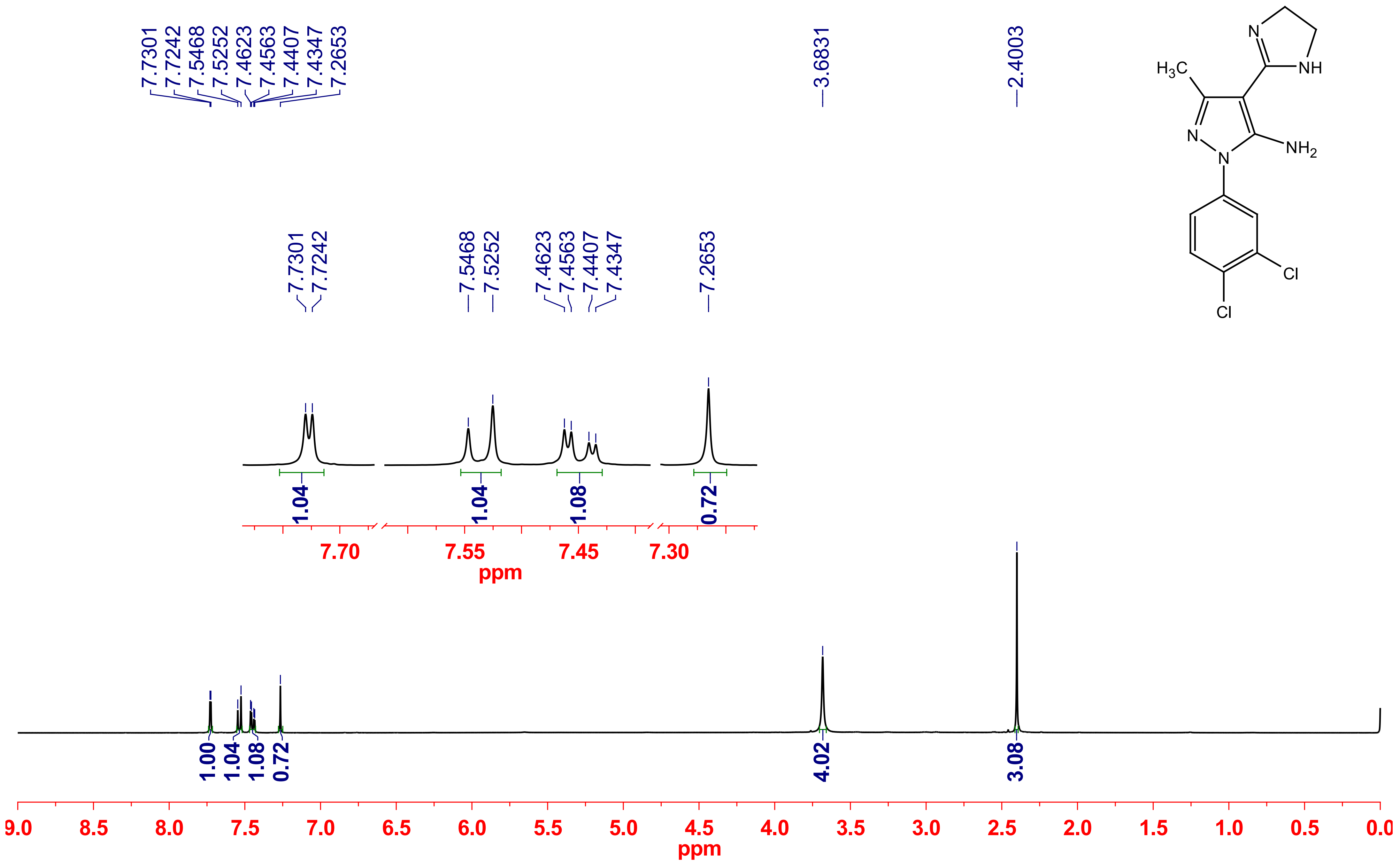

^{13}C NMR of compound **4c**

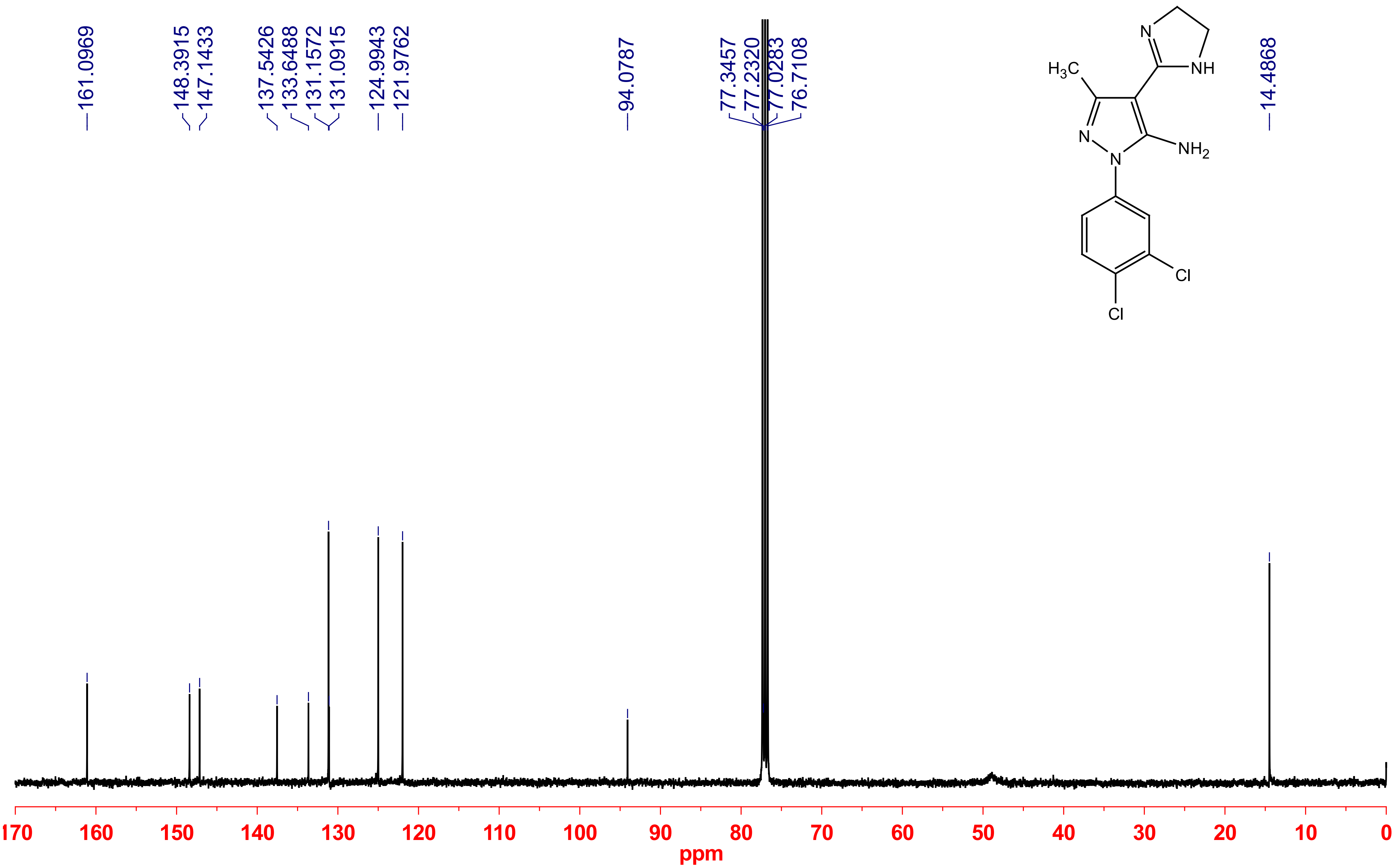

^{1}H NMR of compound **5a**

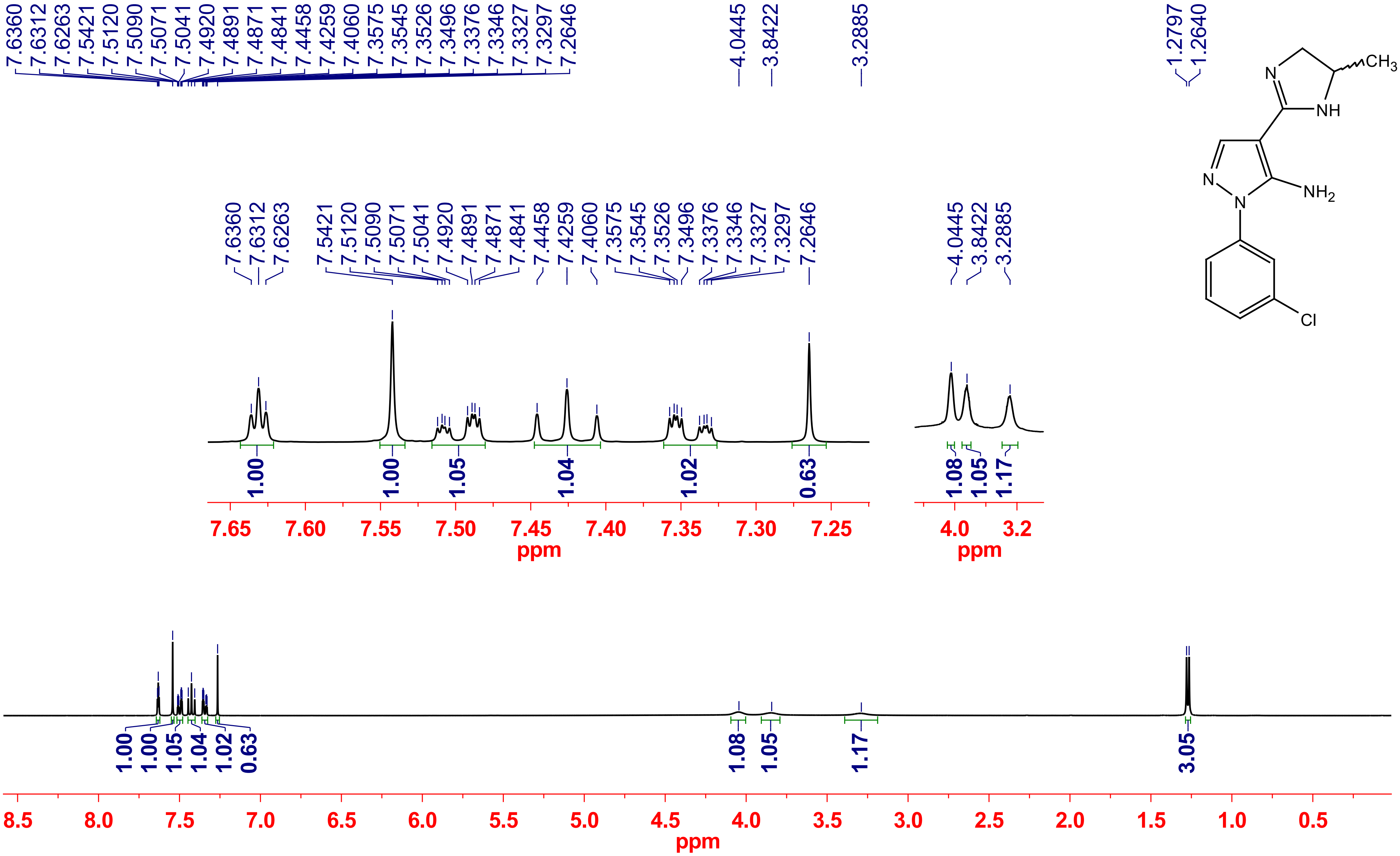

^{13}C NMR of compound **5a**

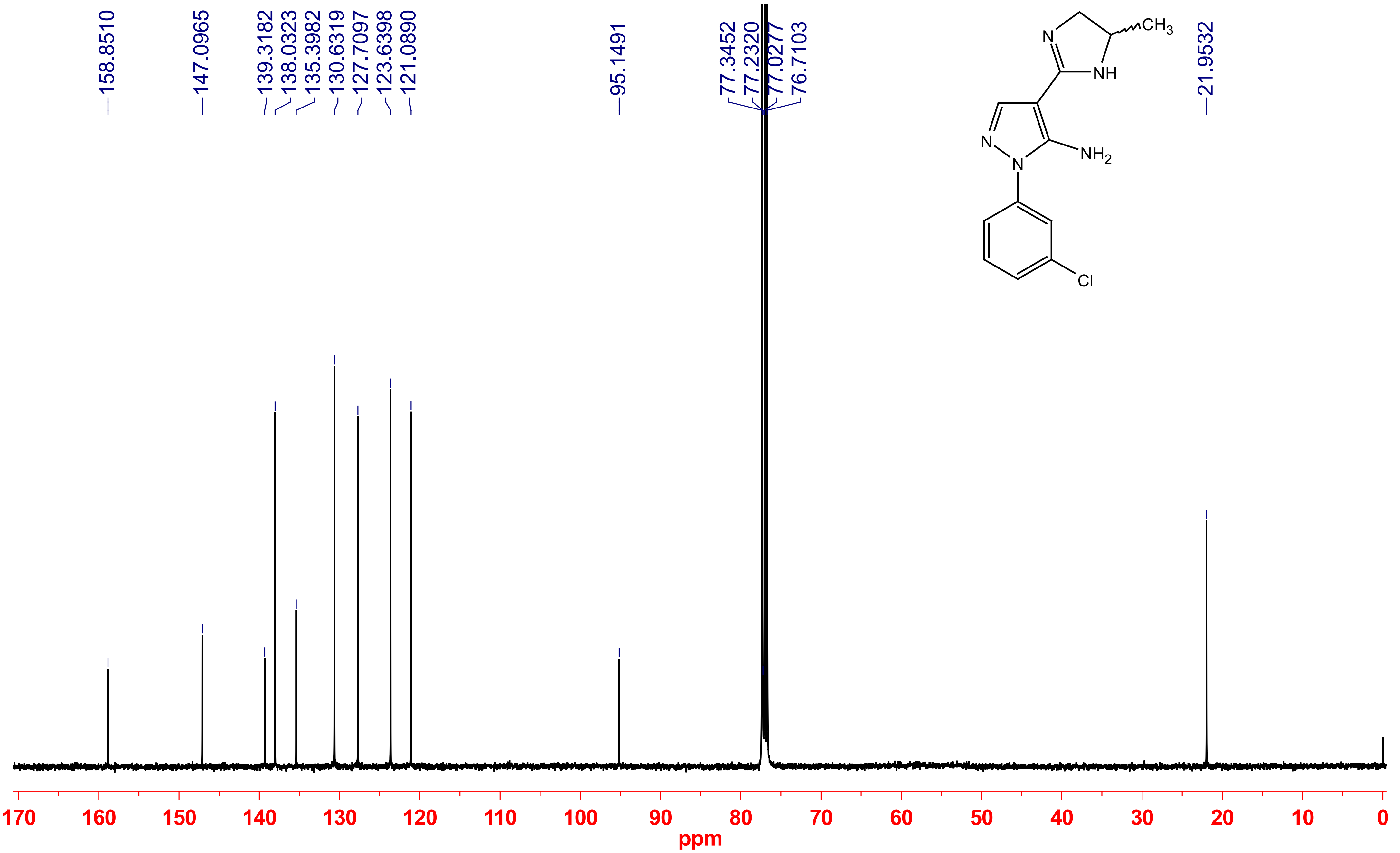

^{1}H NMR of compound **5b**

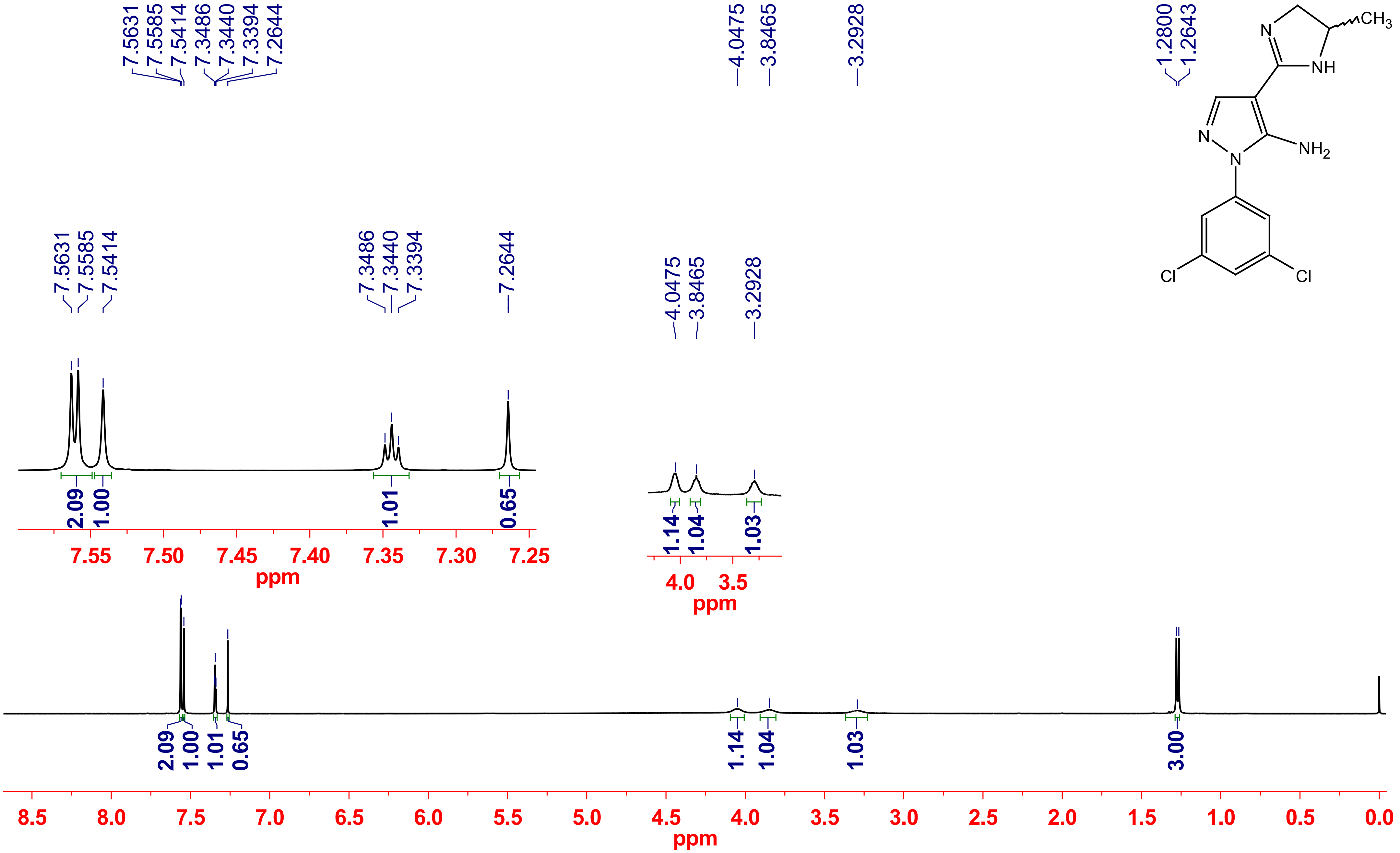

^{13}C NMR of compound **5b**

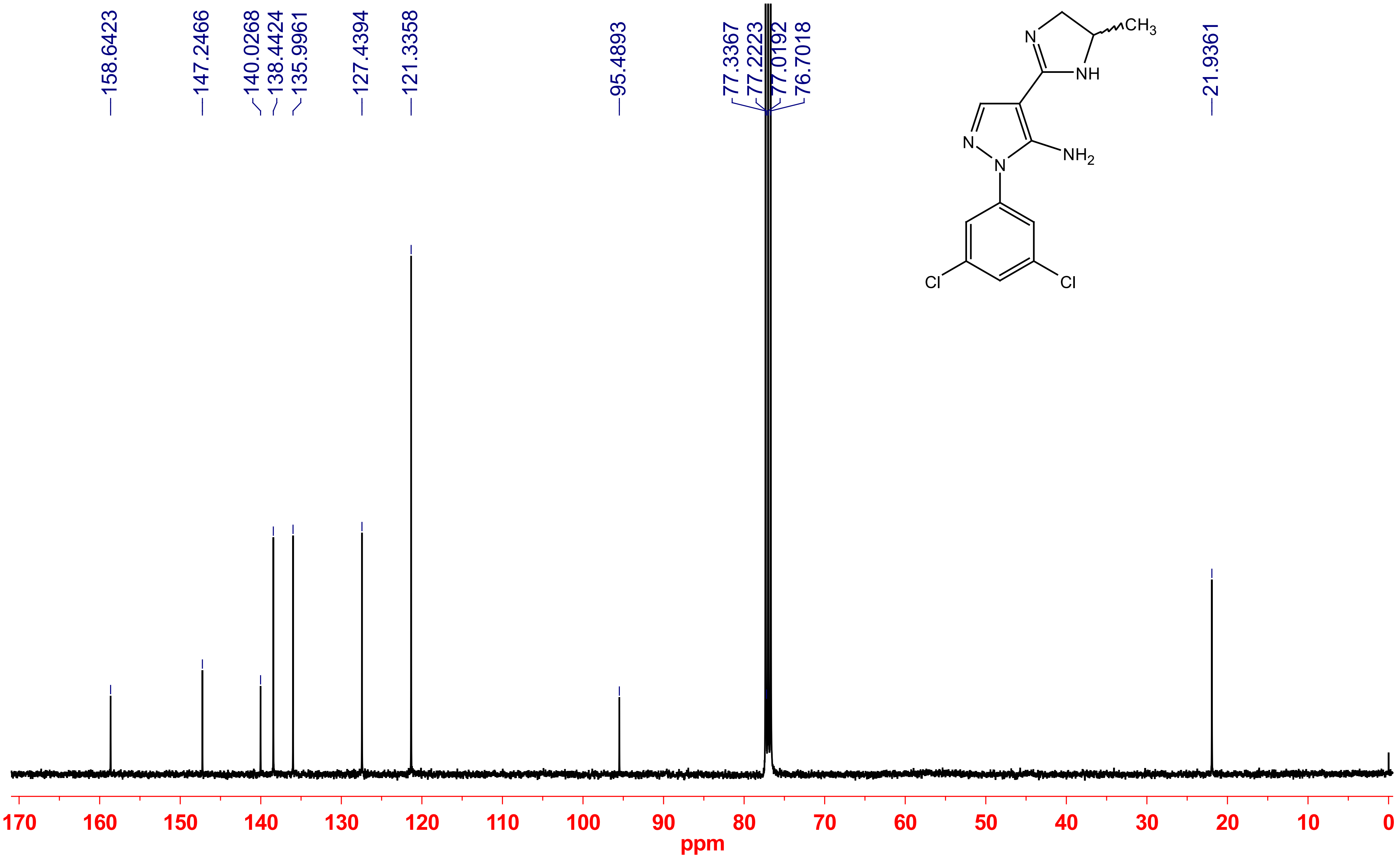

^{1}H NMR of compound **5c**

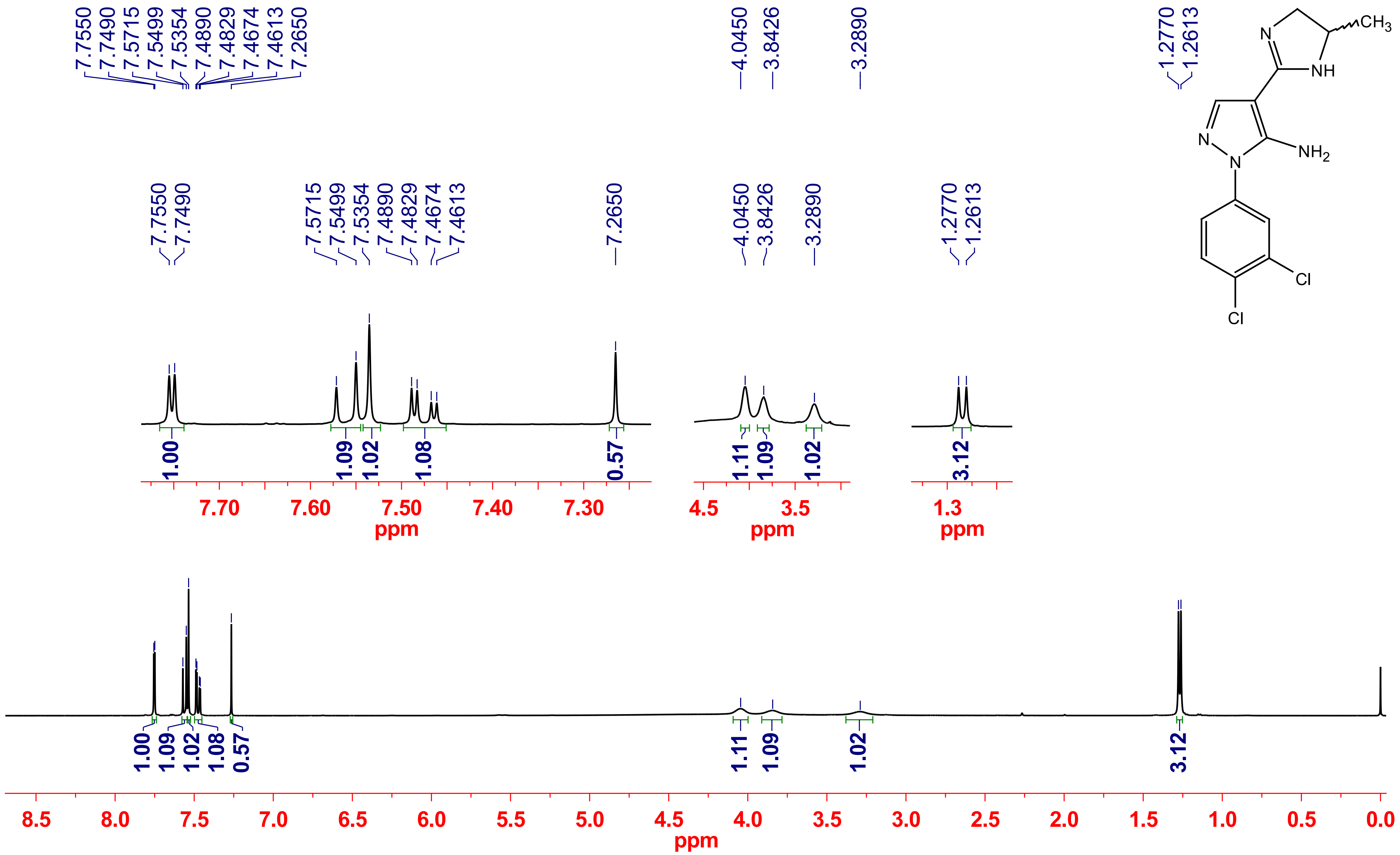

^{13}C NMR of compound **5c**

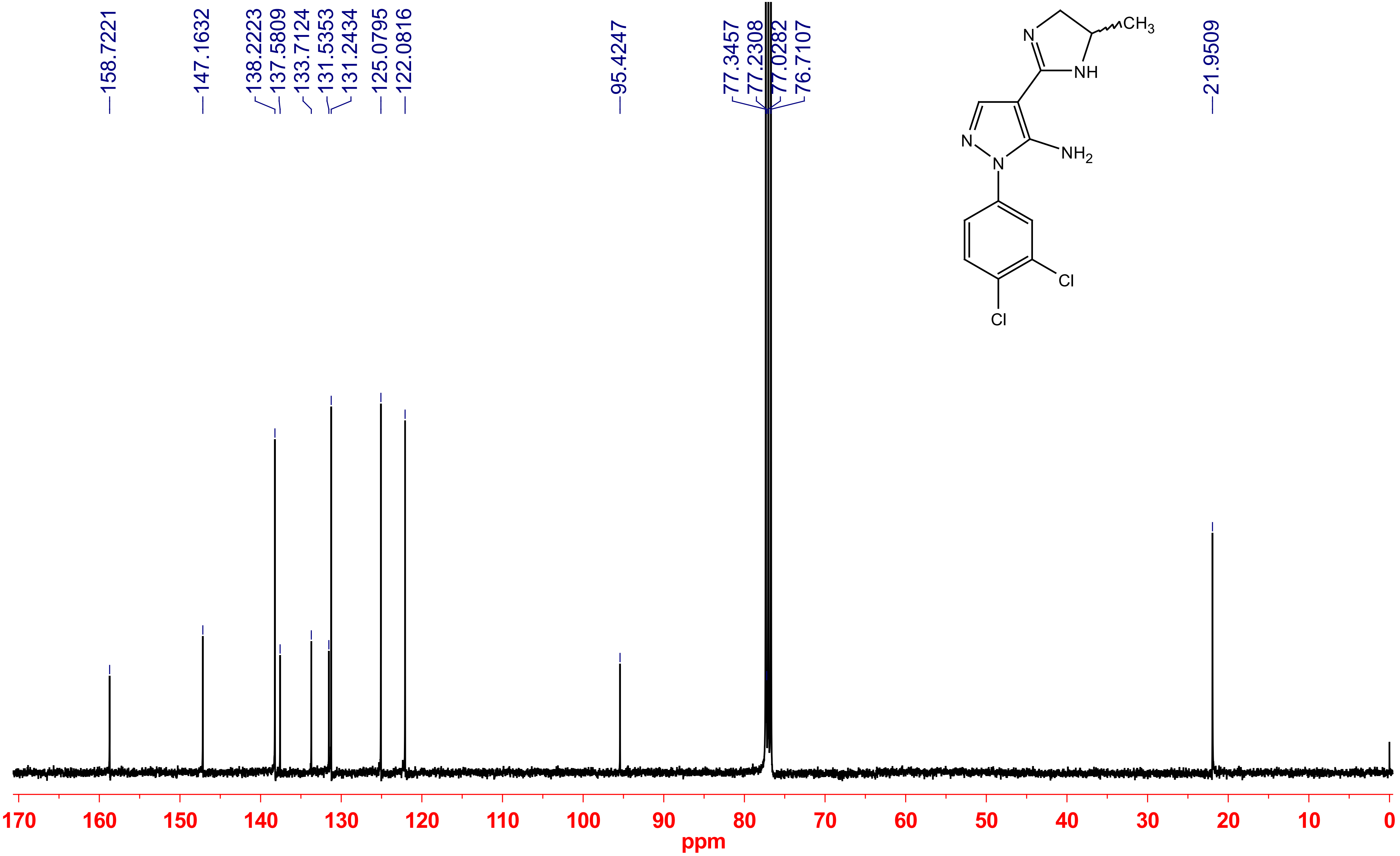

HRMS of compound **1a**

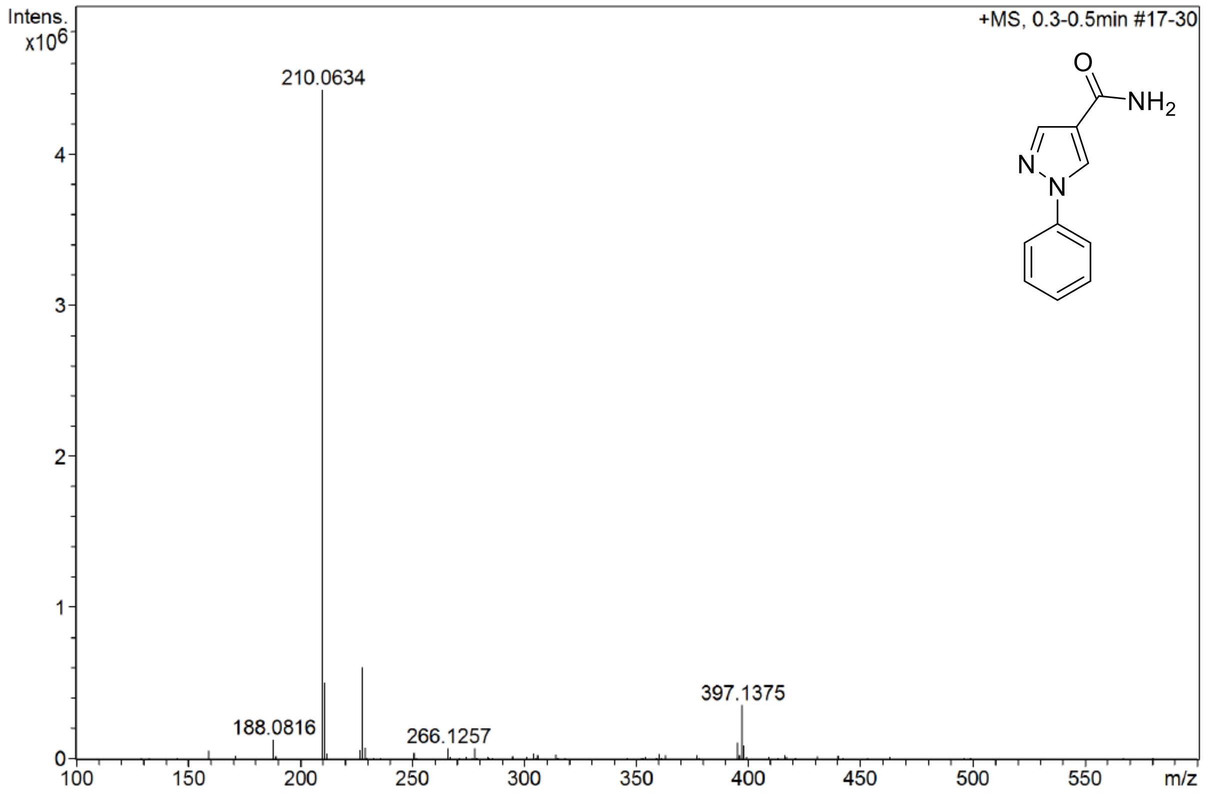

HRMS of compound **1b**

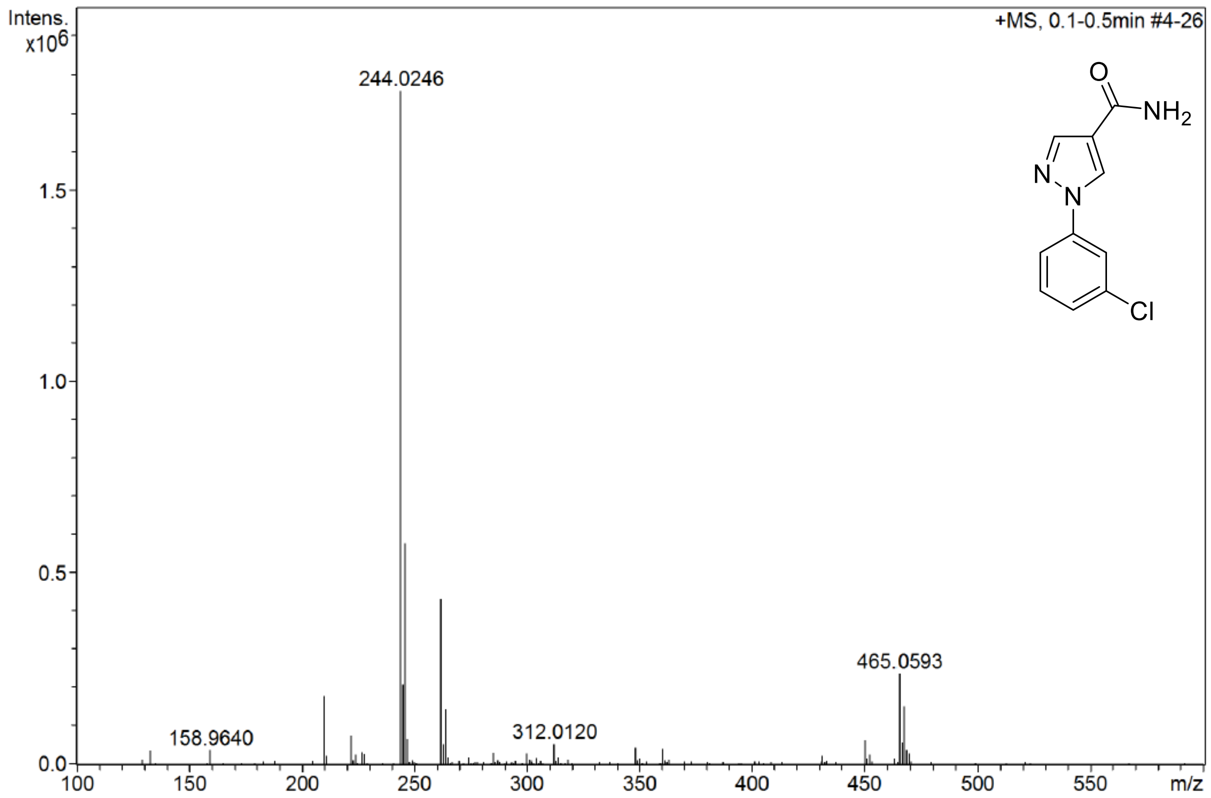

HRMS of compound **1c**

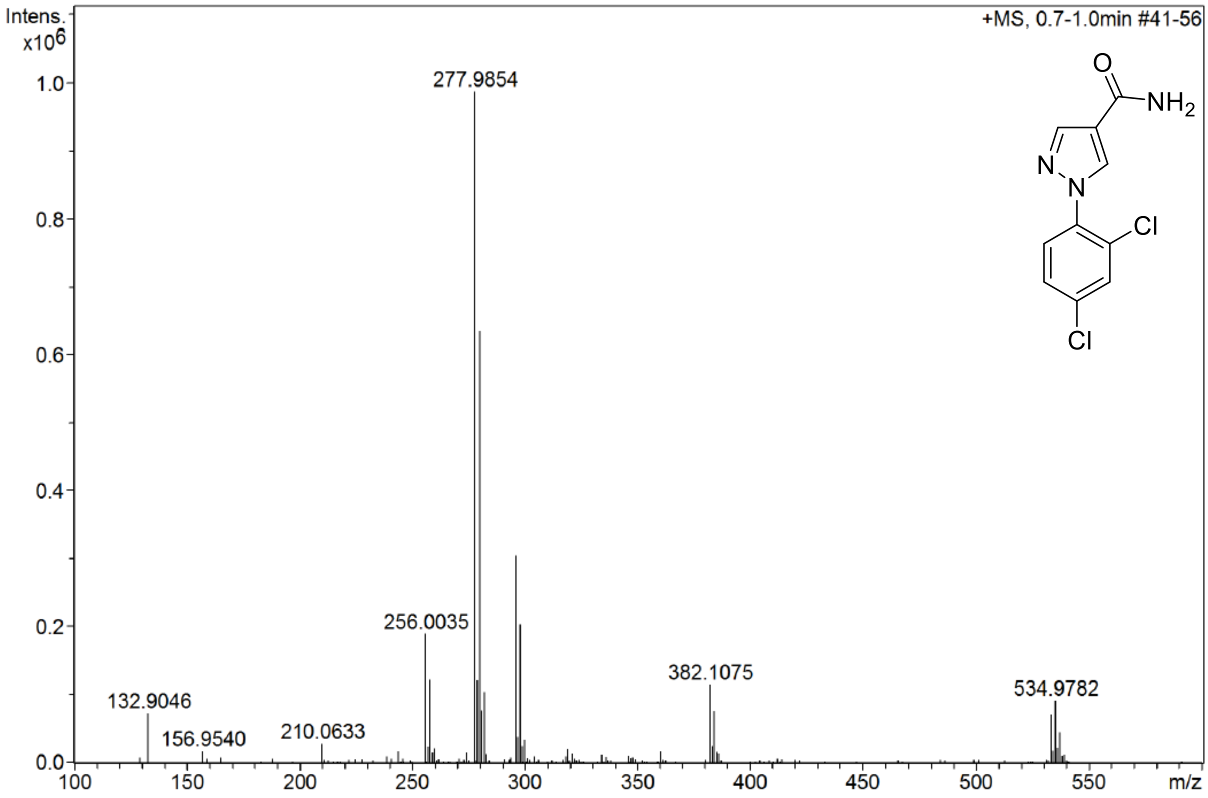

HRMS of compound **1d**

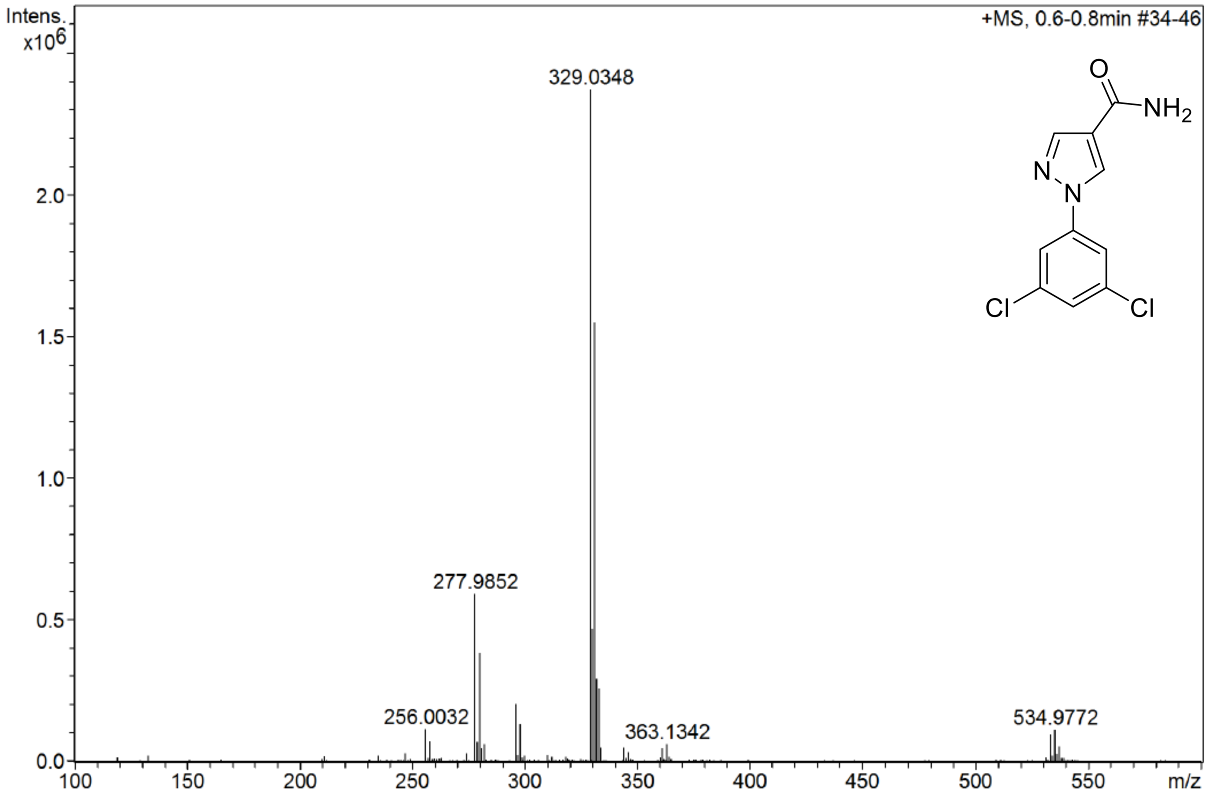

HRMS of compound **1e**

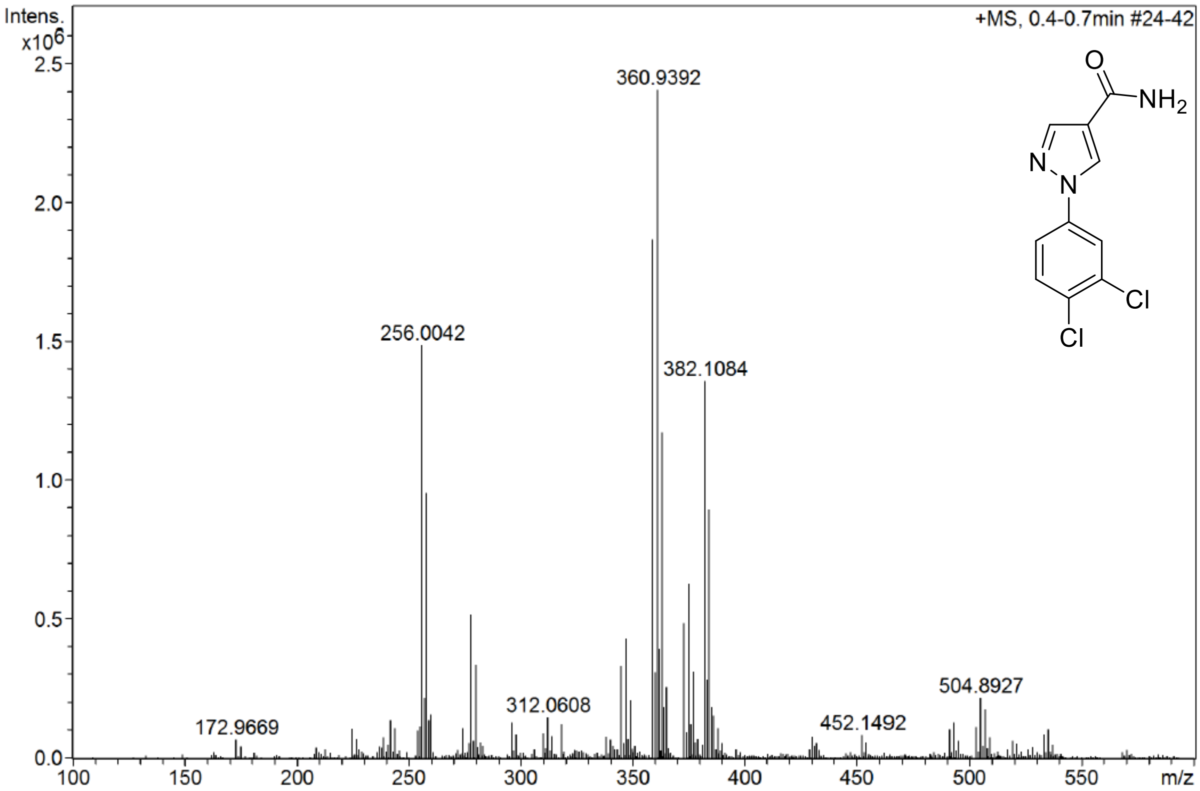

HRMS of compound **1f**

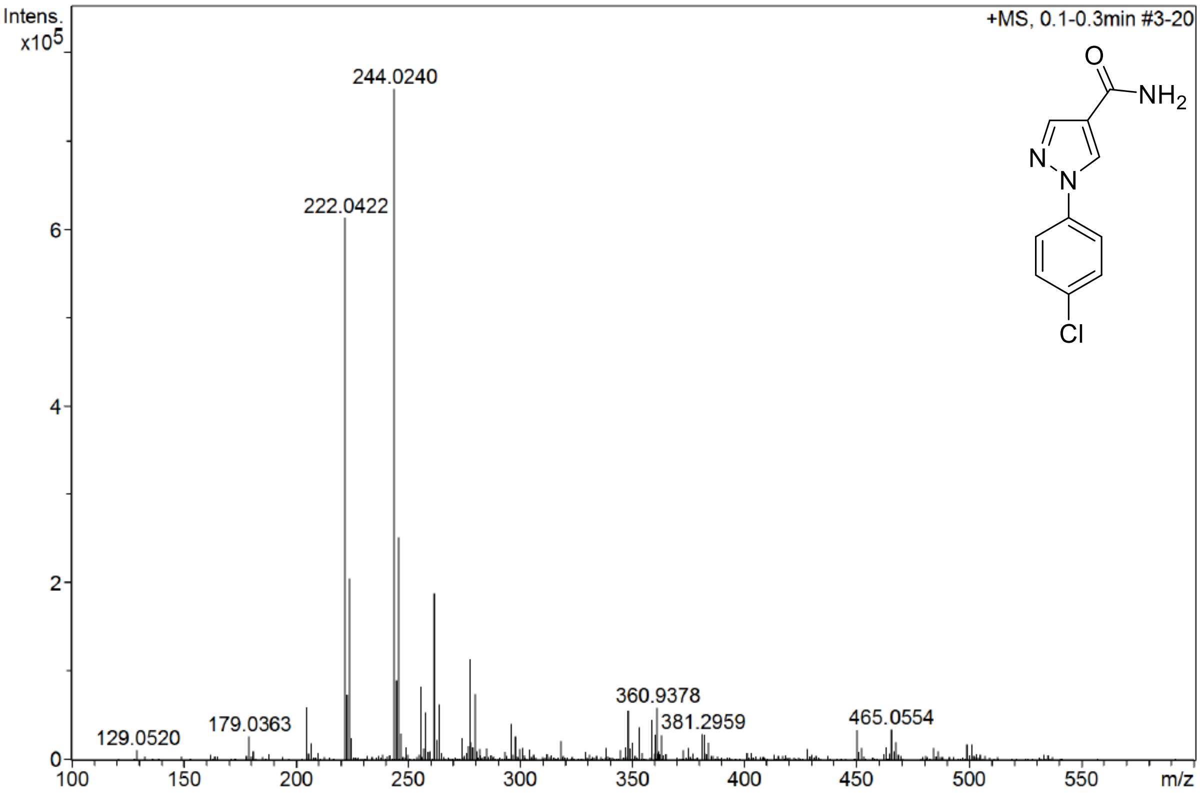

HRMS of compound **1g**

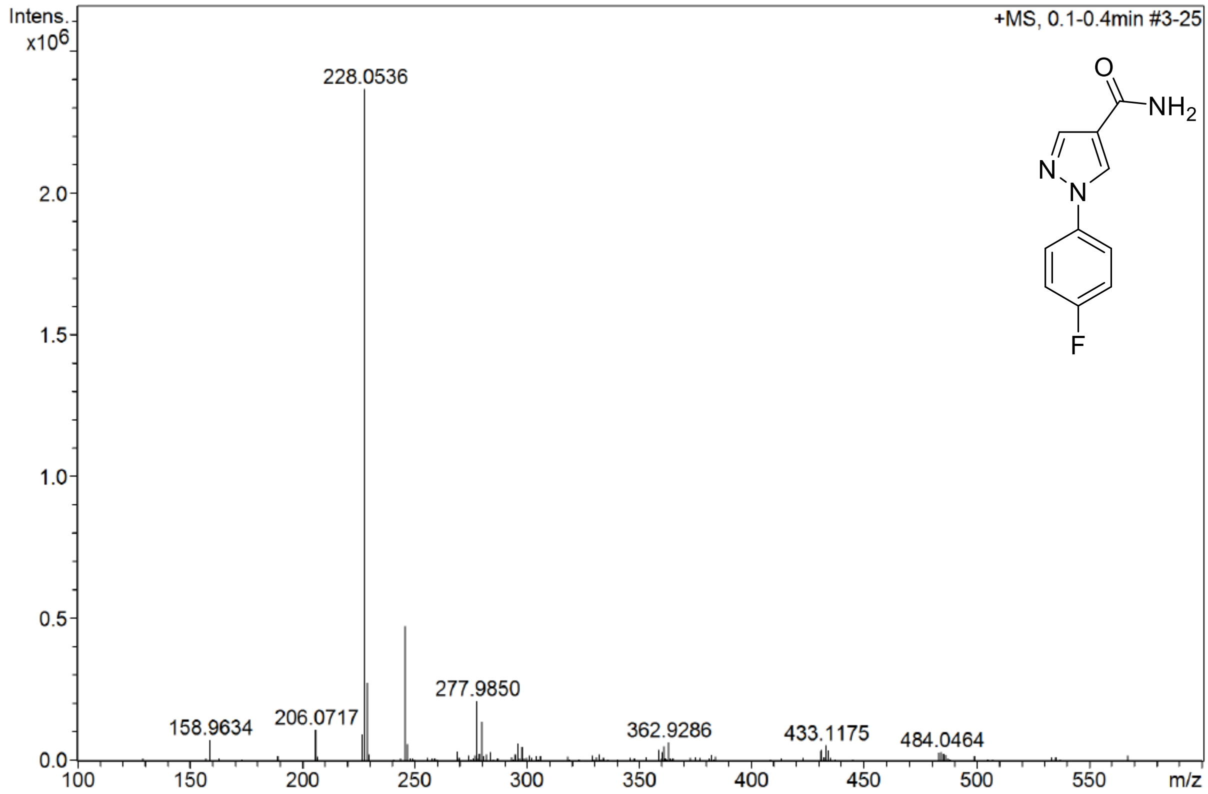

HRMS of compound **1h**

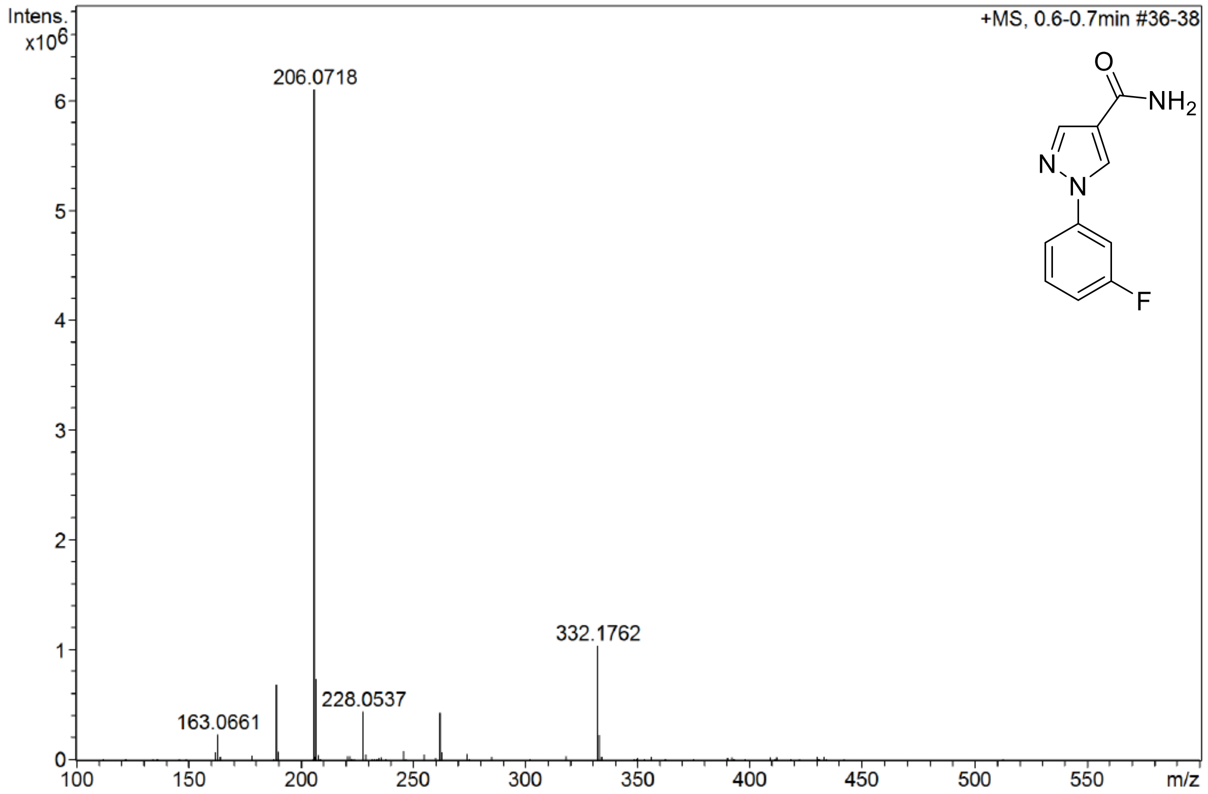

HRMS of compound **1i**

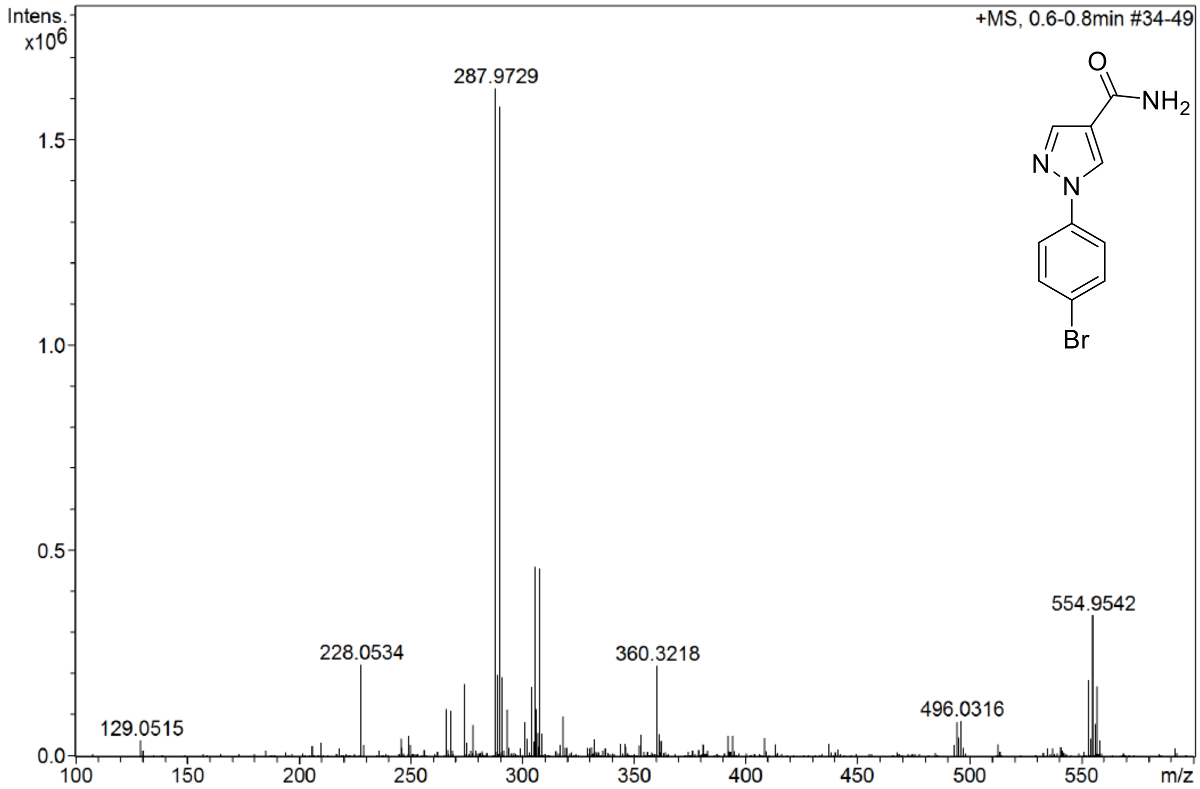

HRMS of compound **1j**

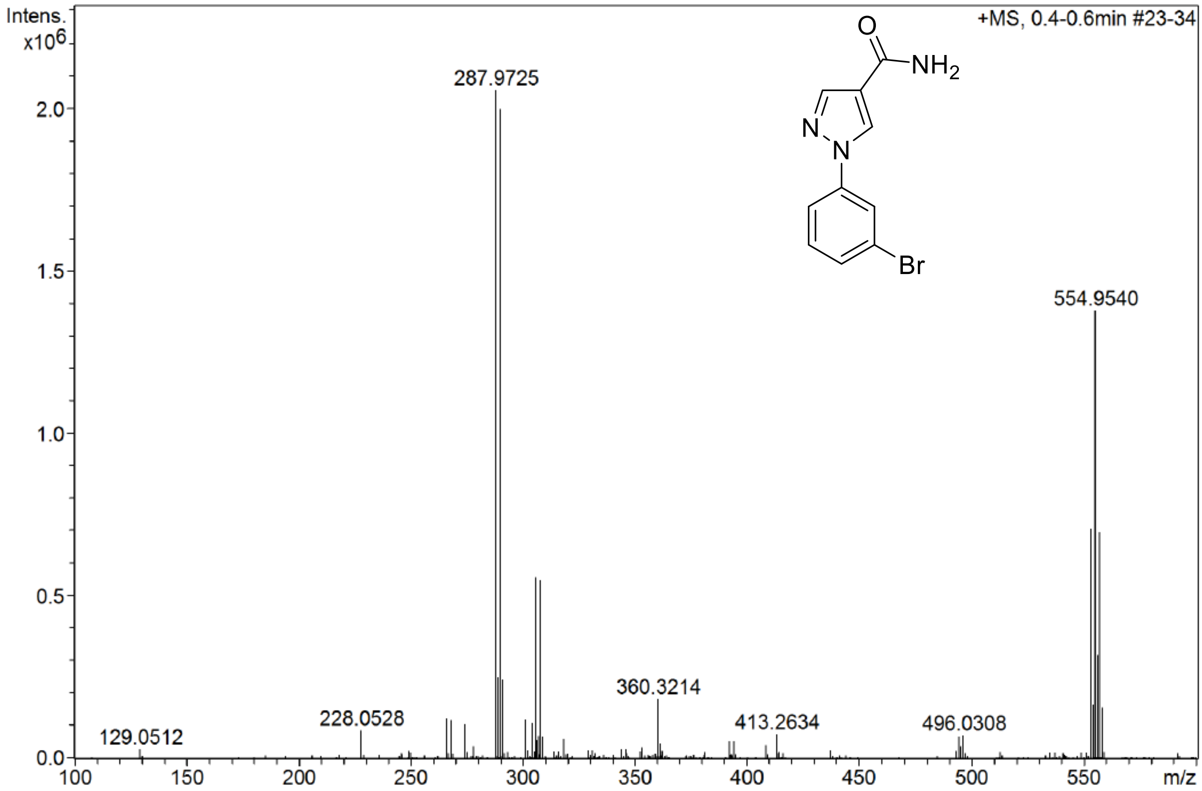

HRMS of compound **1k**

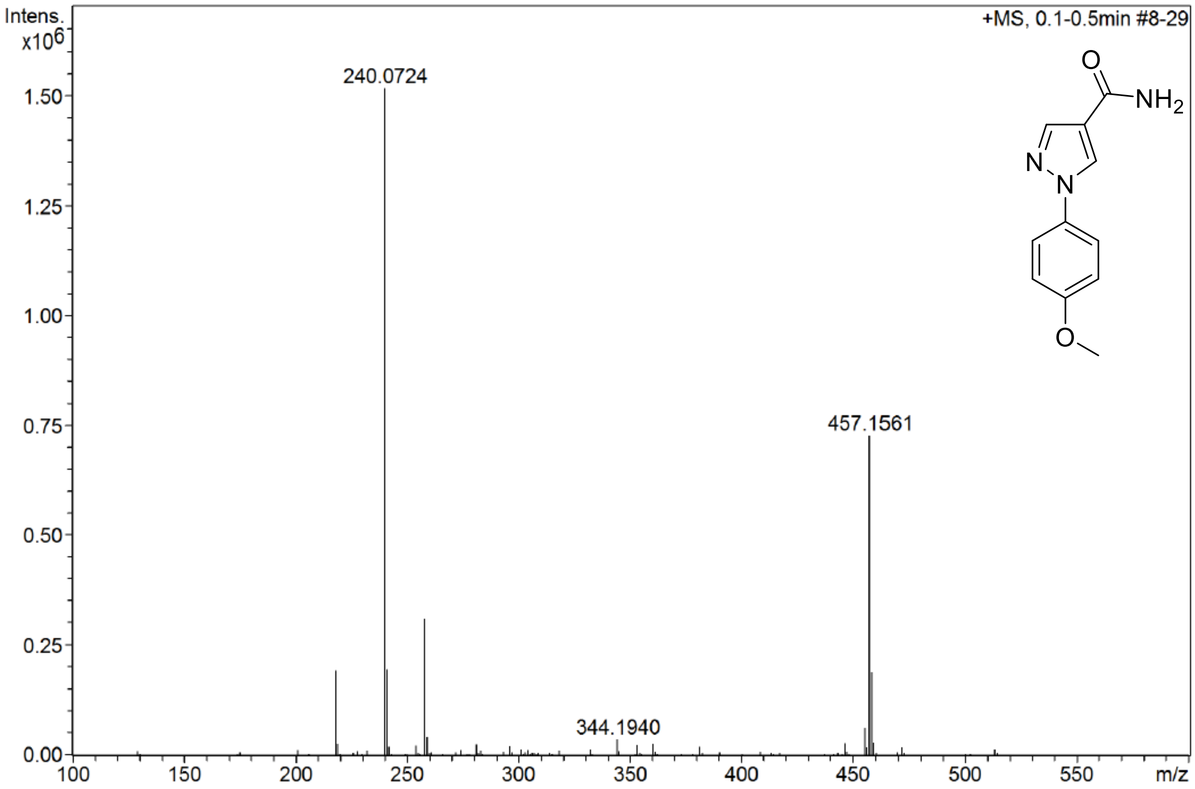

HRMS of compound **1l**

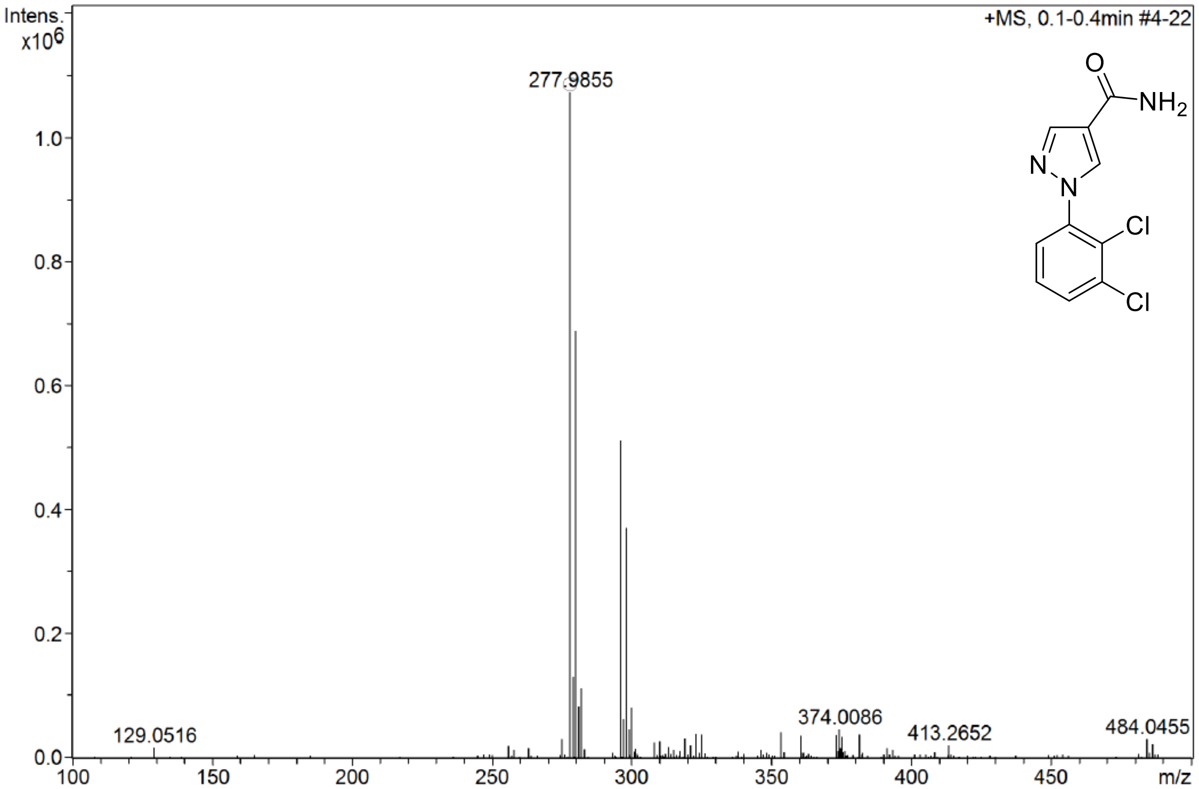

HRMS of compound **2a**

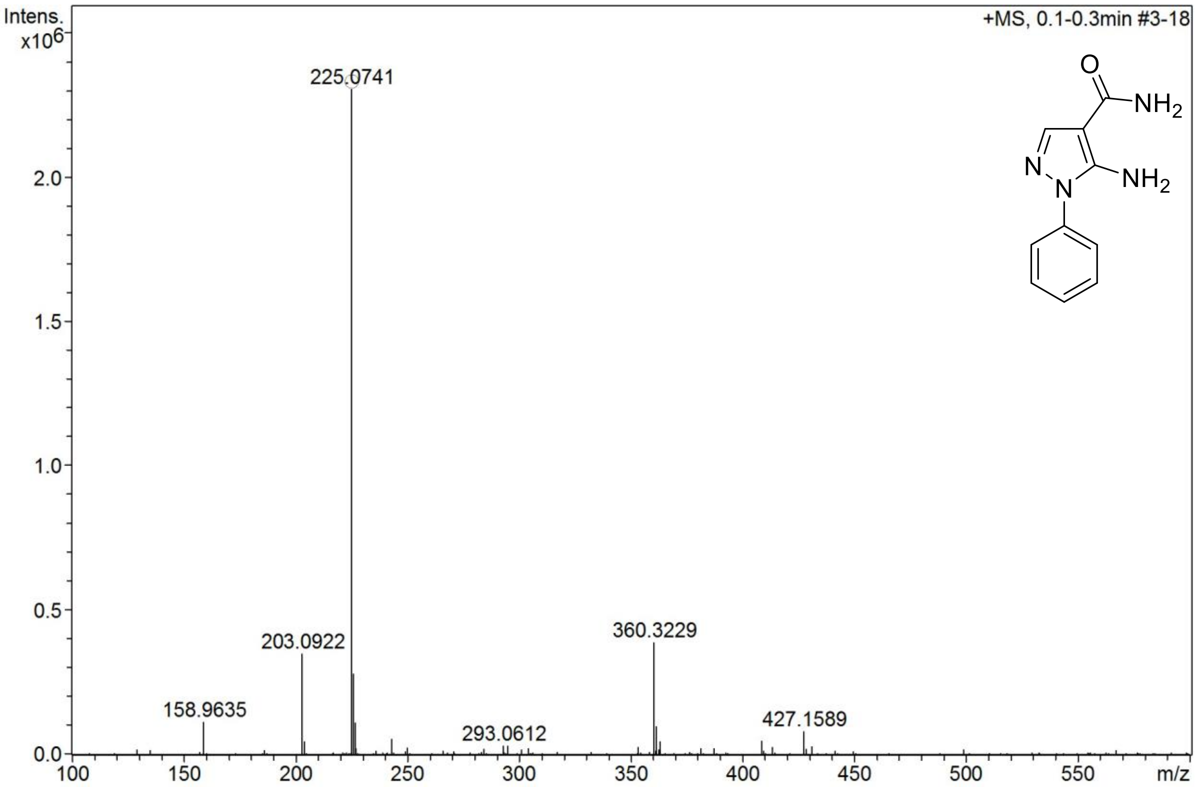

HRMS of compound **2b**

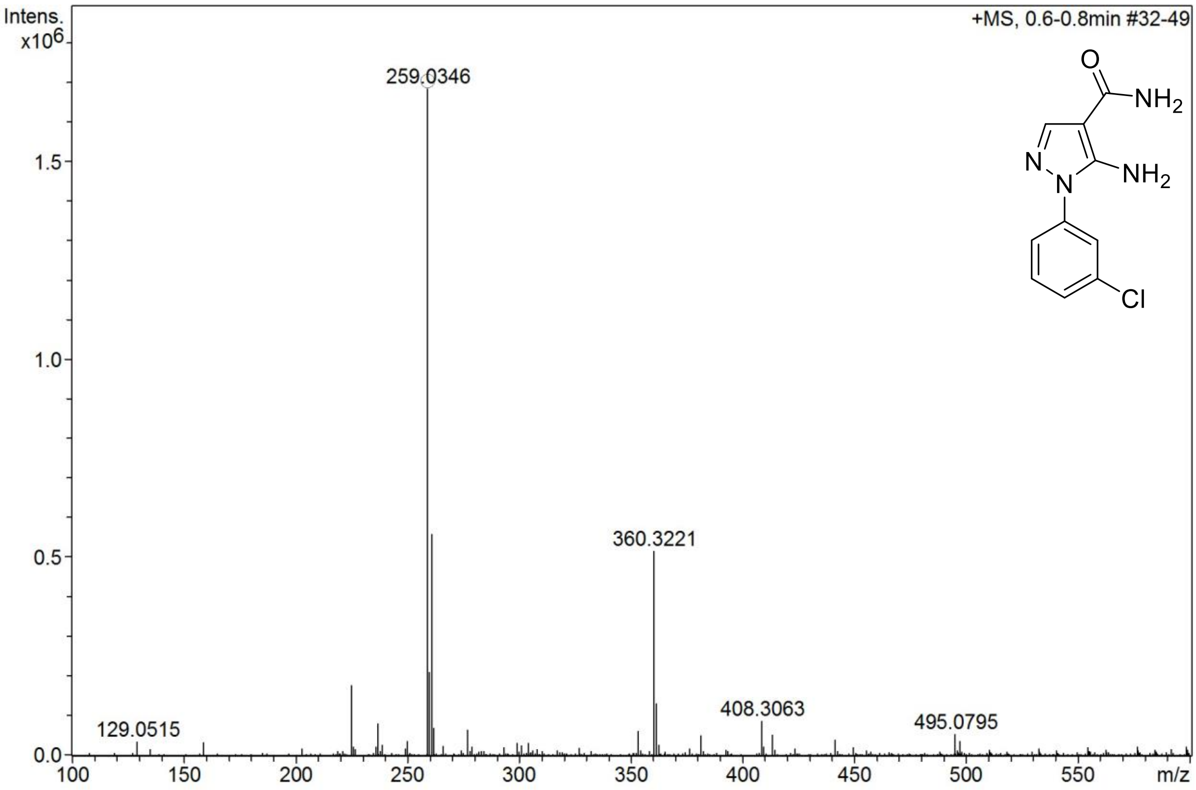

HRMS of compound **2c**

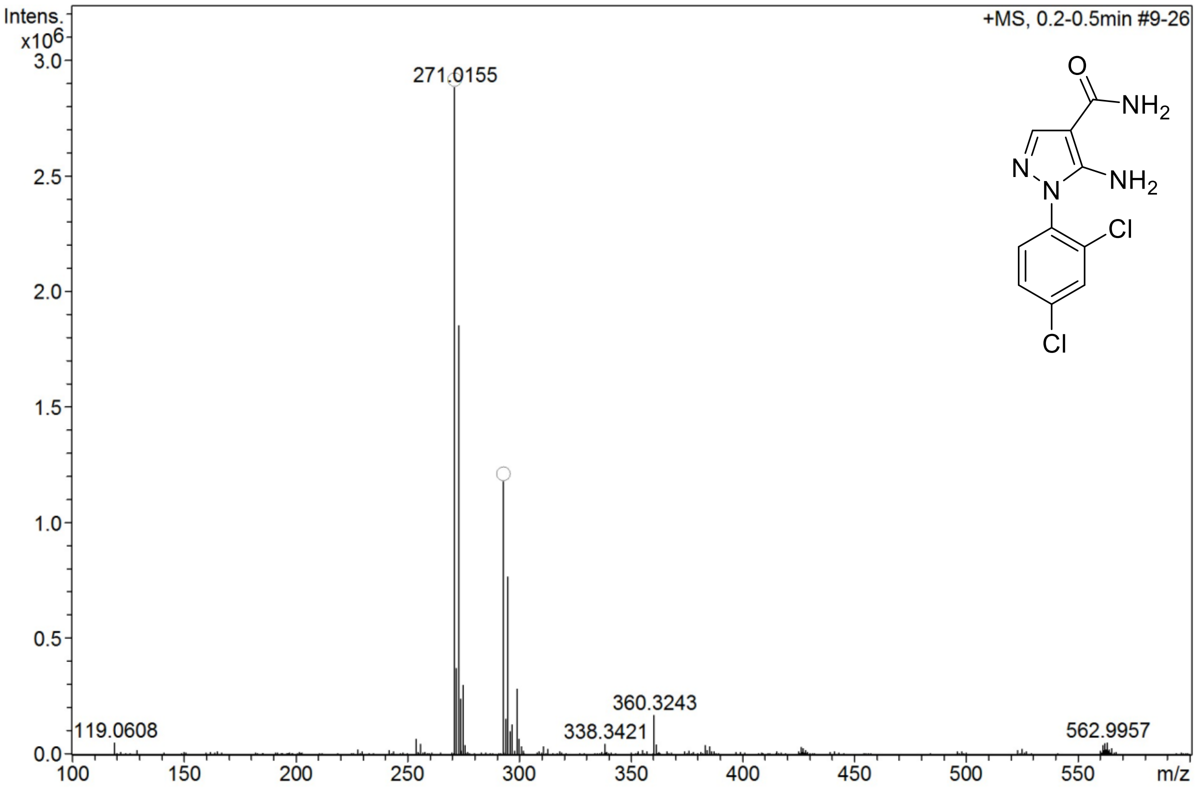

HRMS of compound **2d**

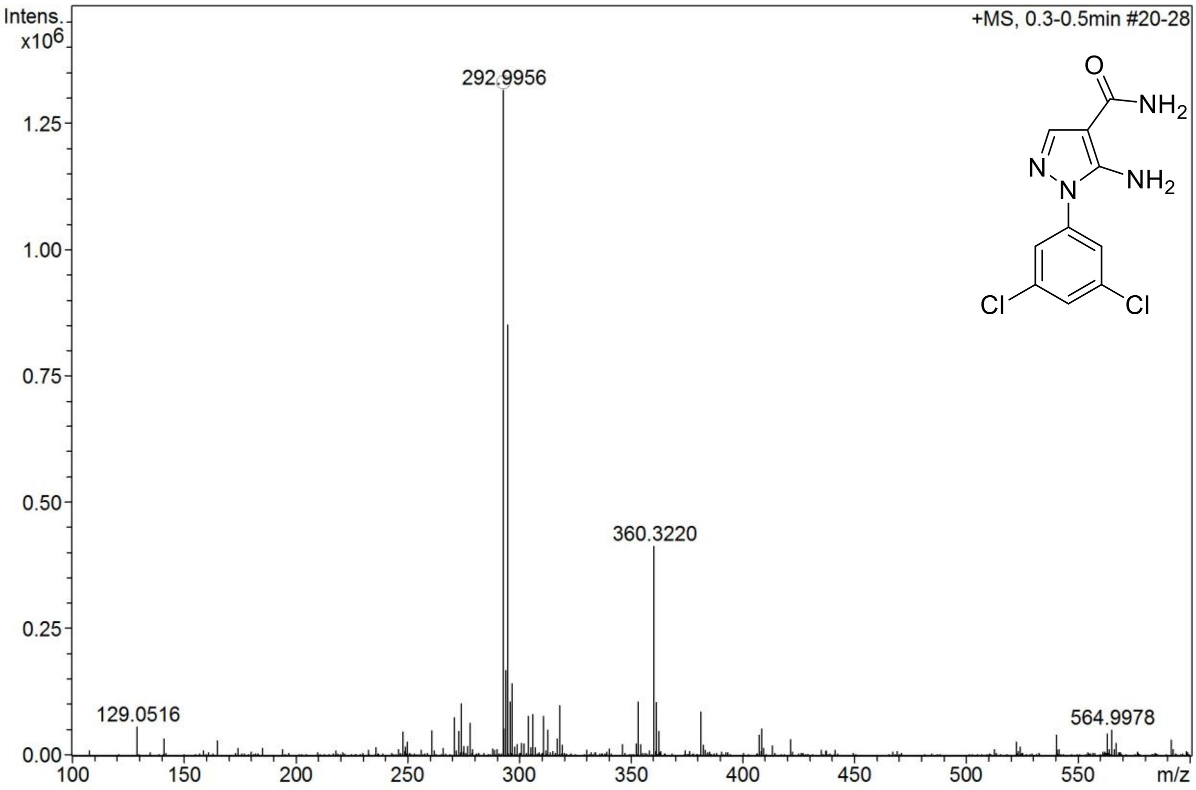

HRMS of compound **2e**

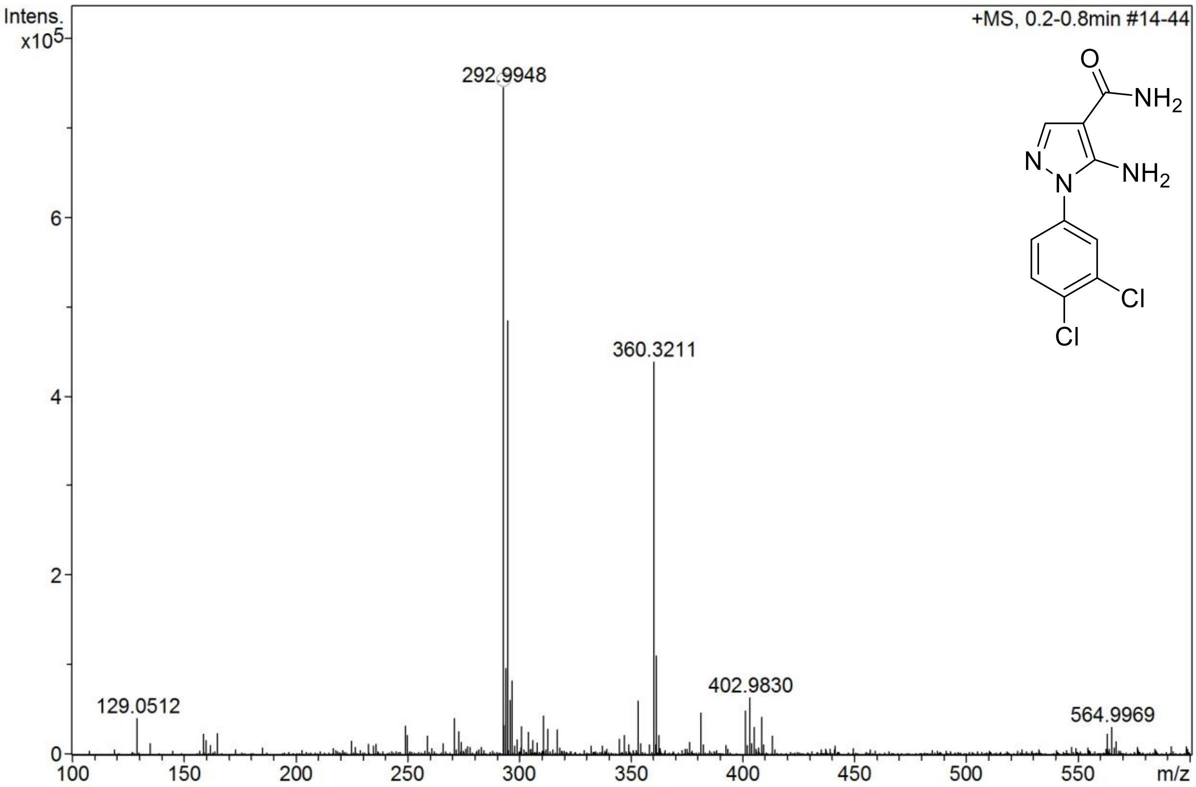

HRMS of compound **2f**

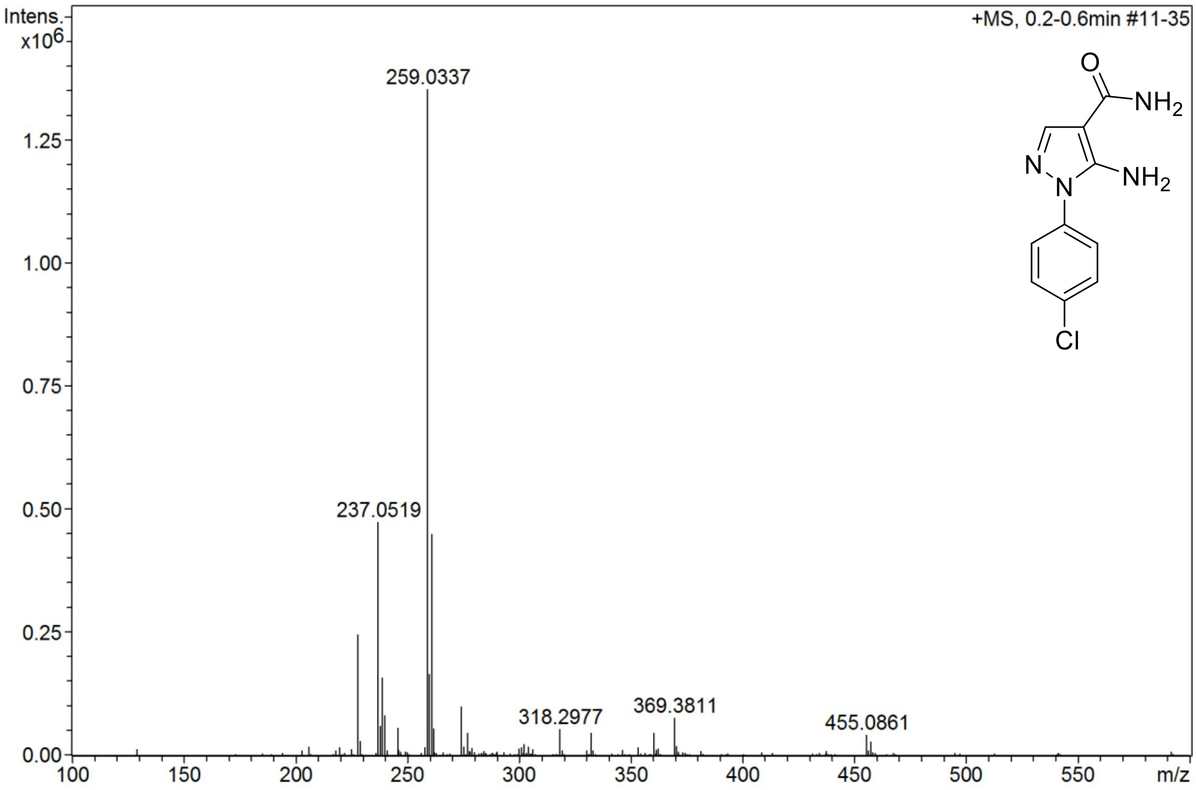

HRMS of compound **2g**

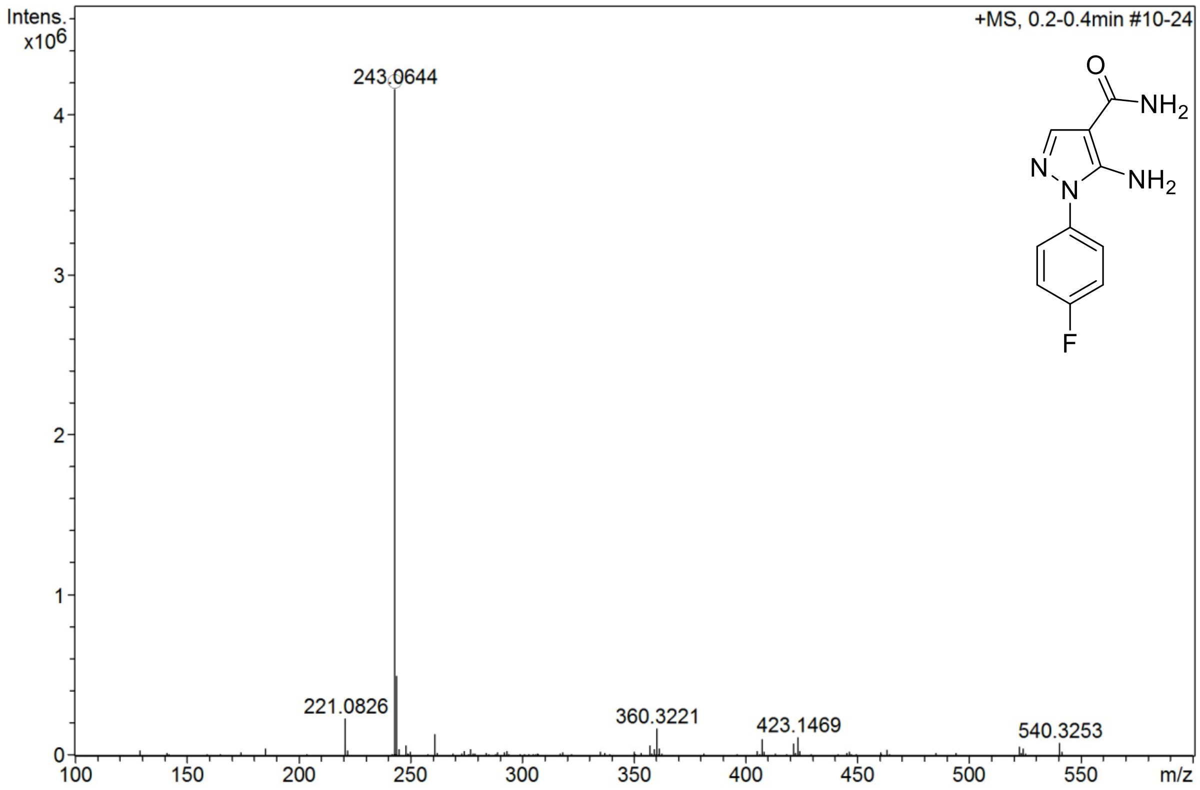

HRMS of compound **2h**

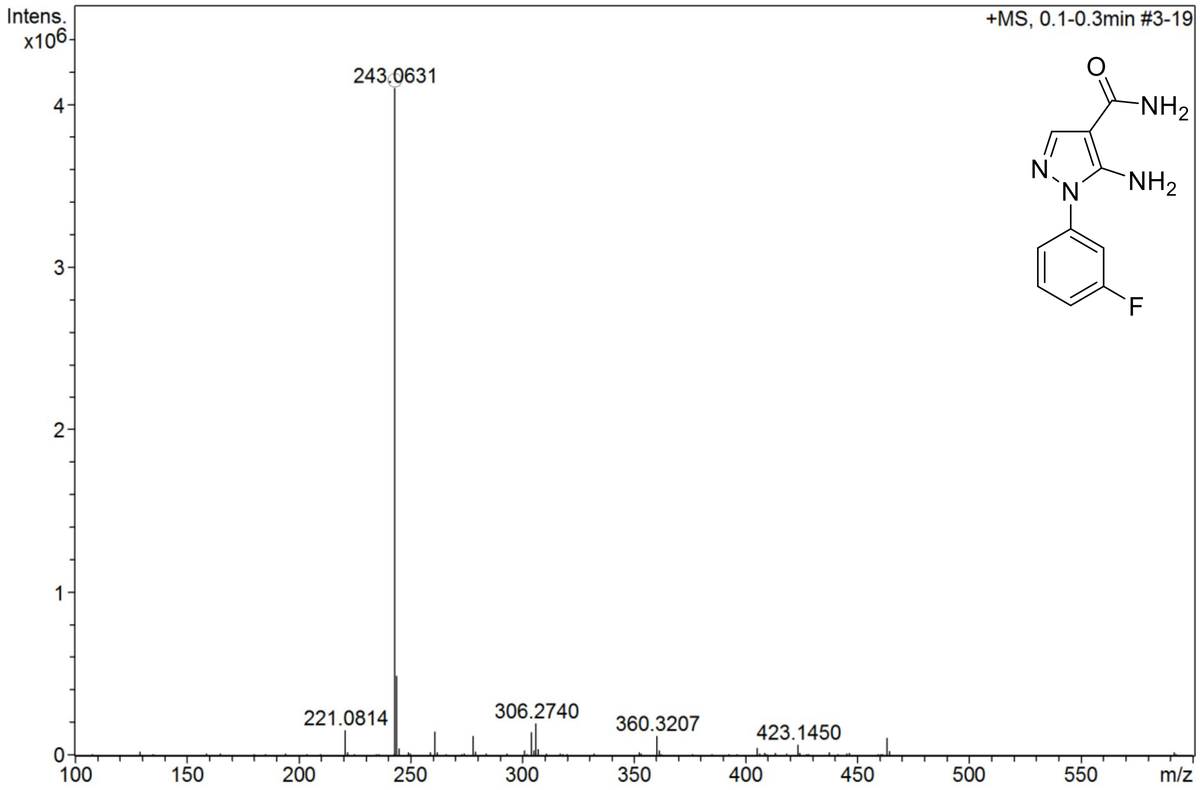

HRMS of compound **2i**

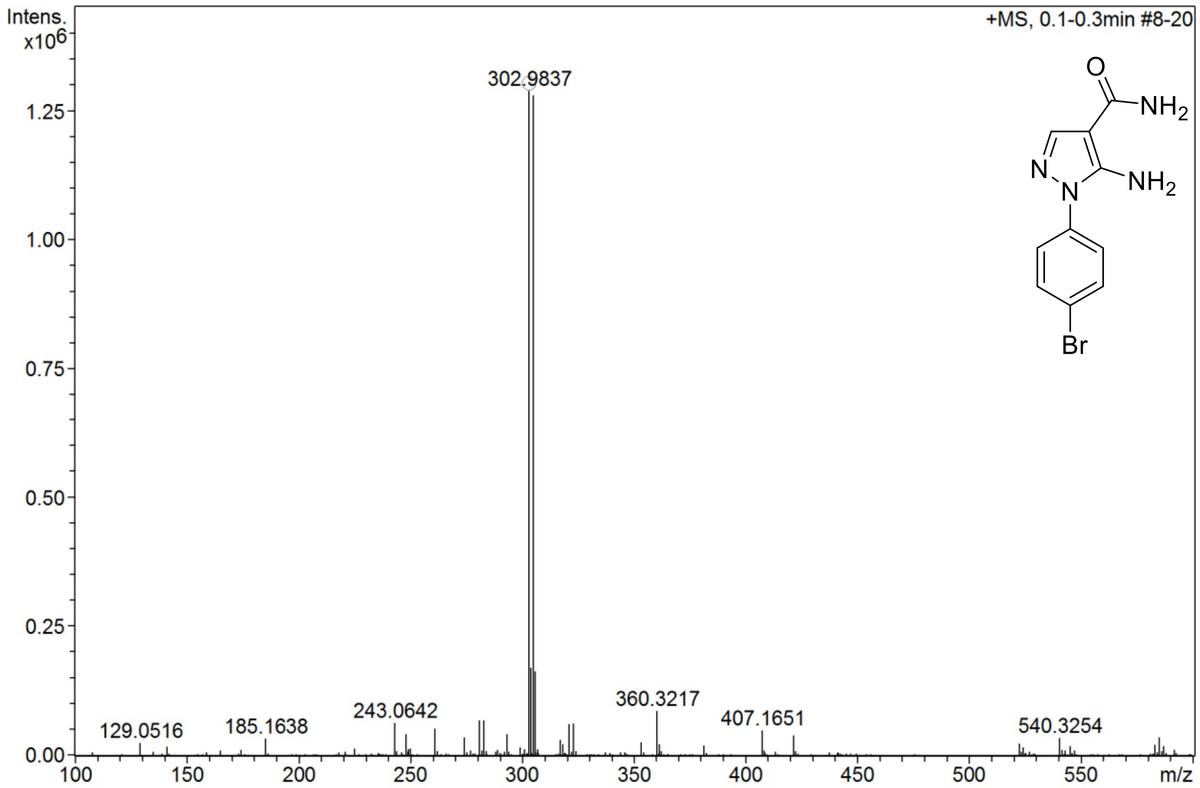

HRMS of compound **2j**

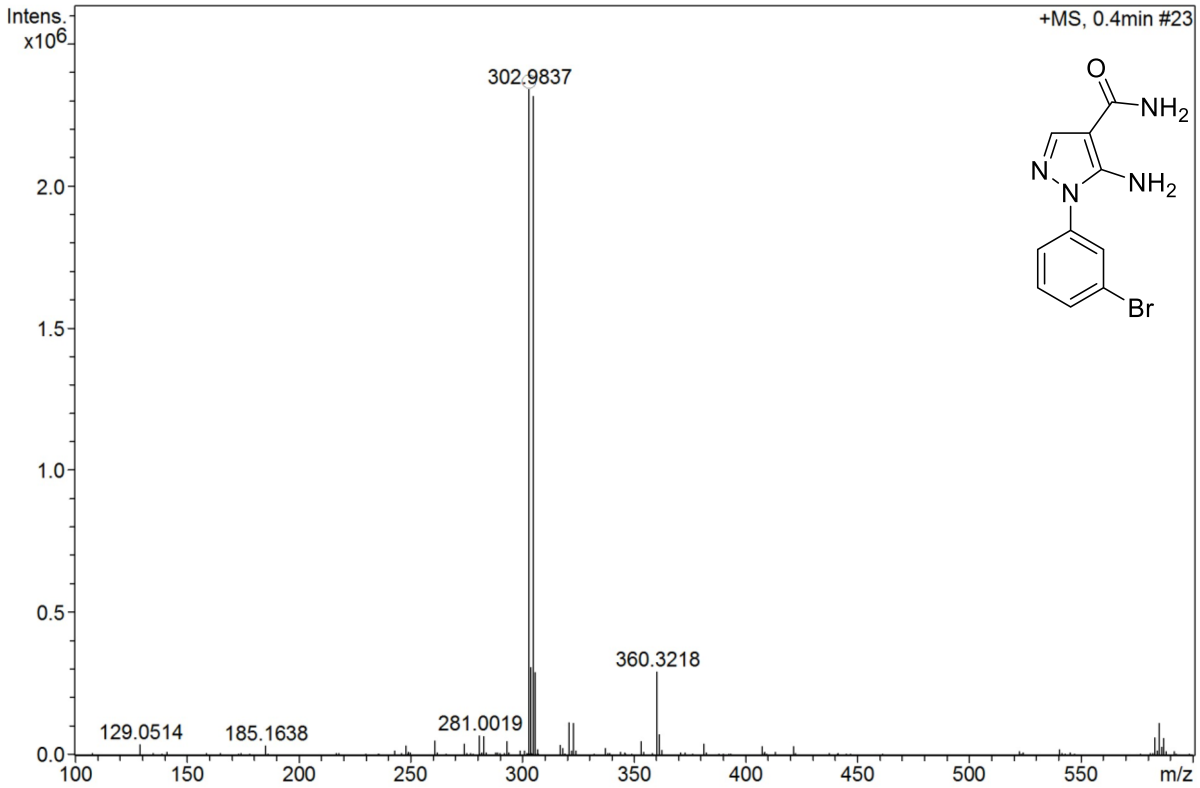

HRMS of compound **2k**

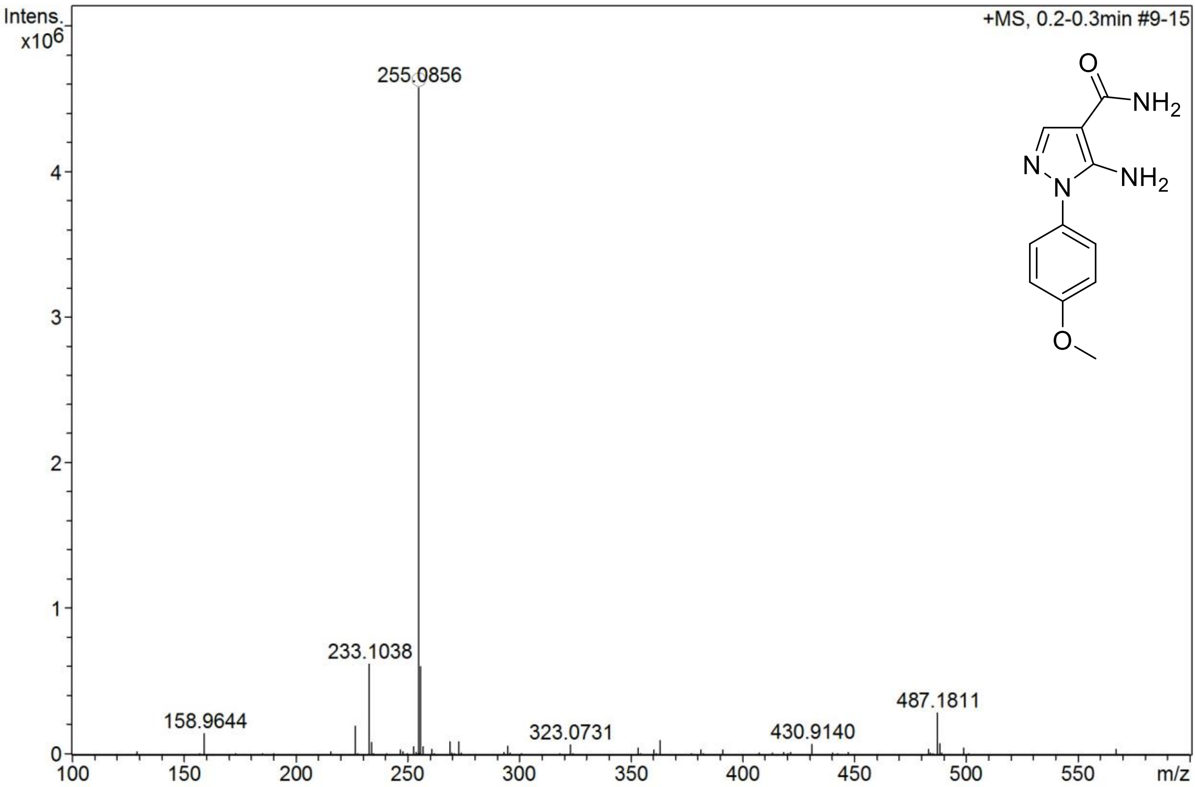

HRMS of compound **2l**

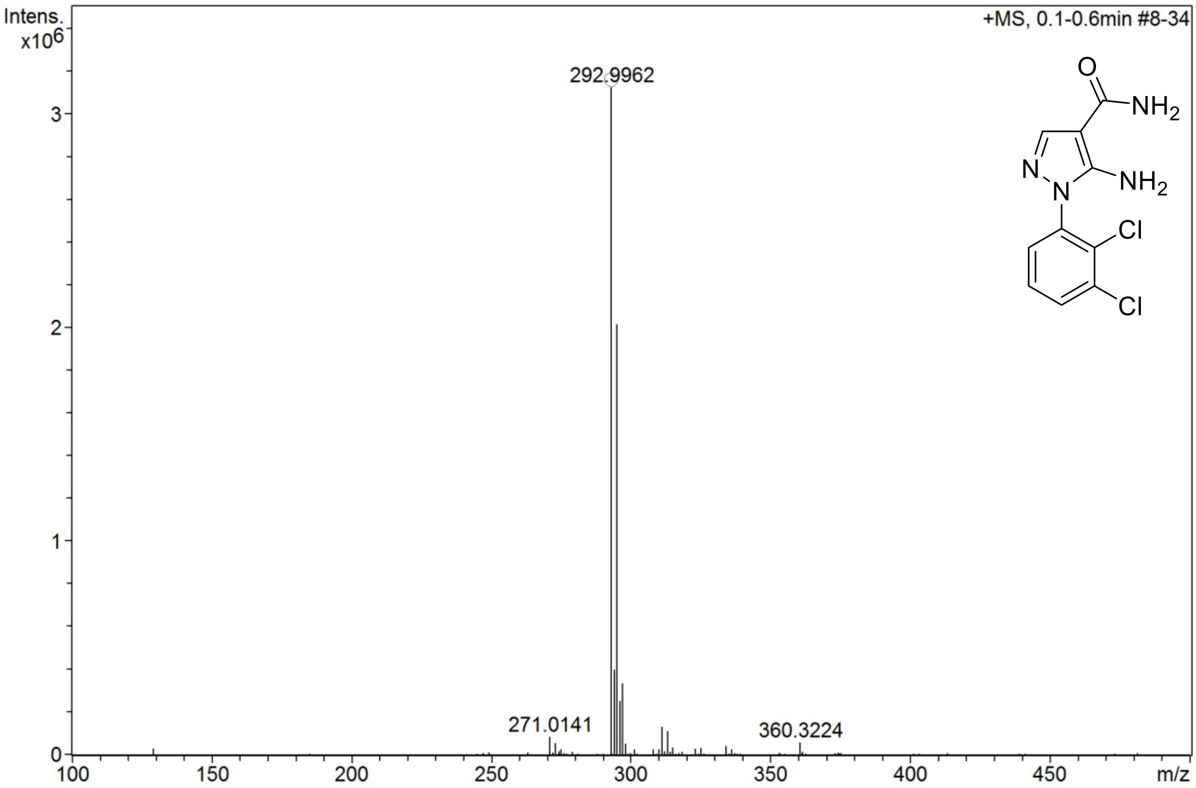

HRMS of compound **3a**

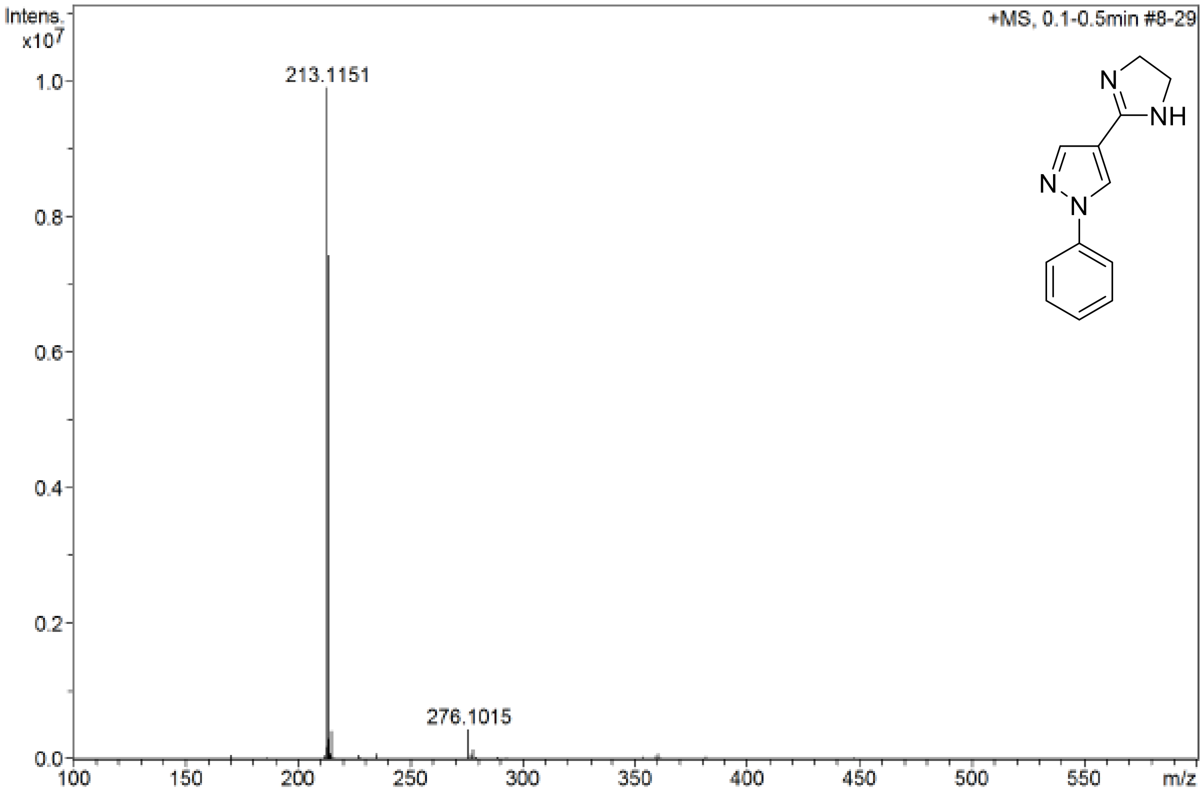

HRMS of compound **3b**

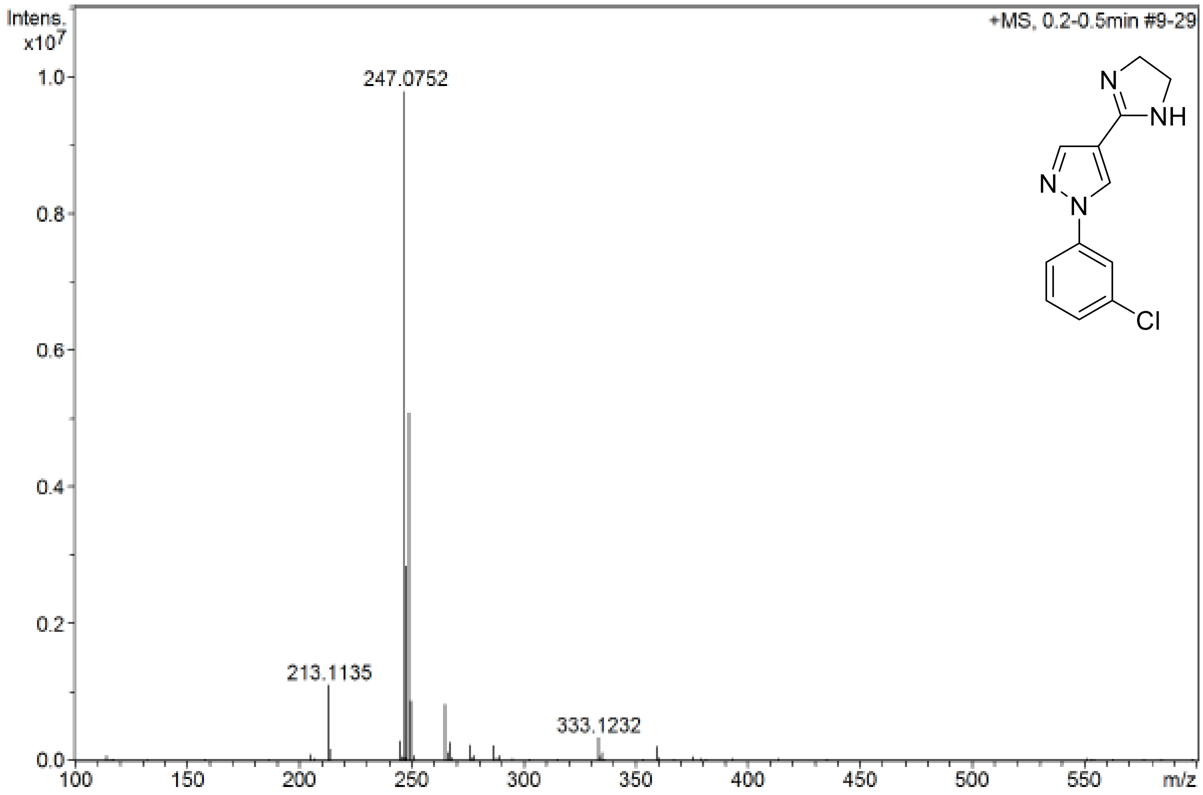

HRMS of compound **3c**

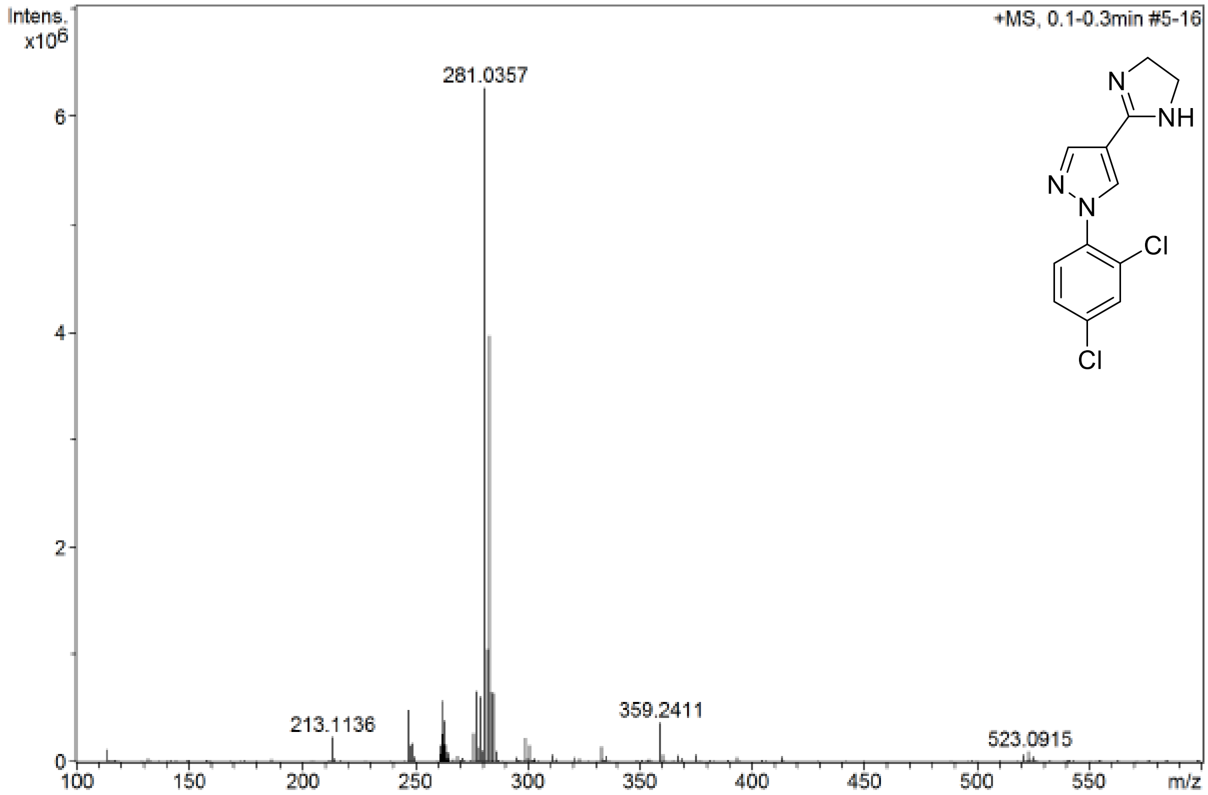

HRMS of compound **3d**

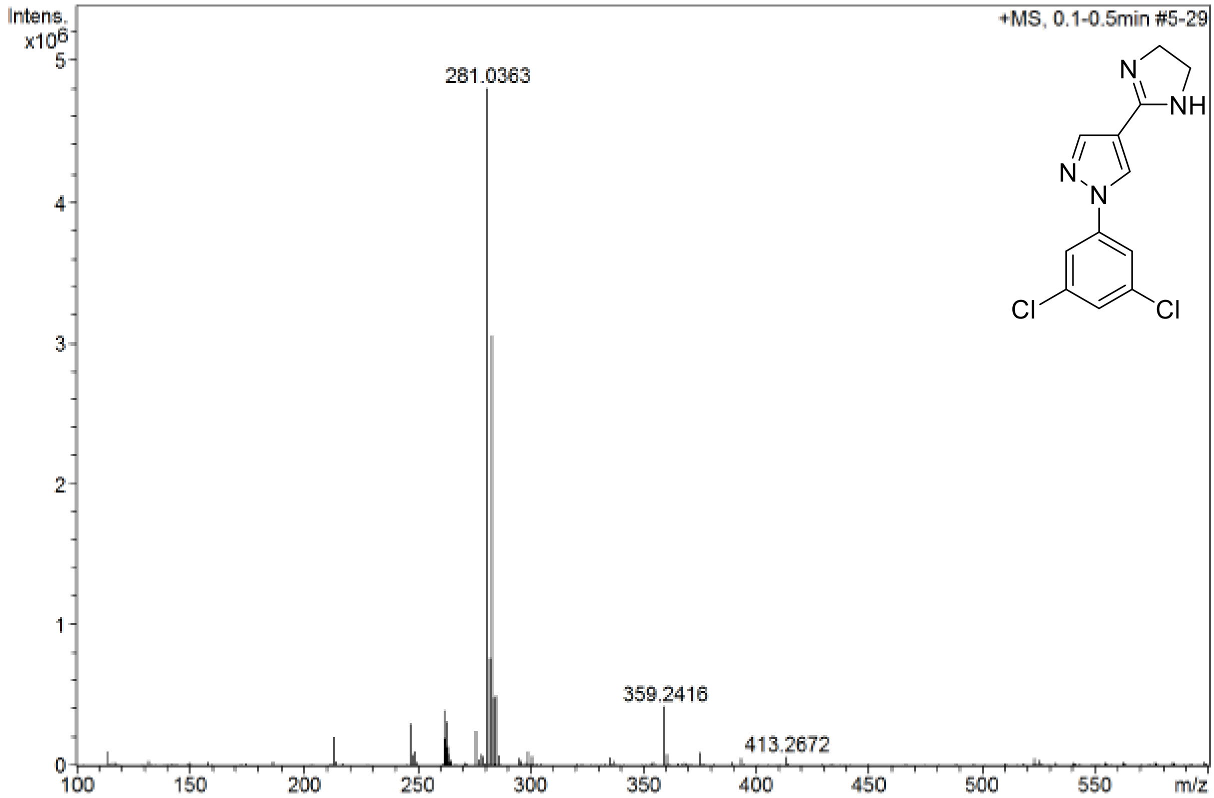

HRMS of compound **3e**

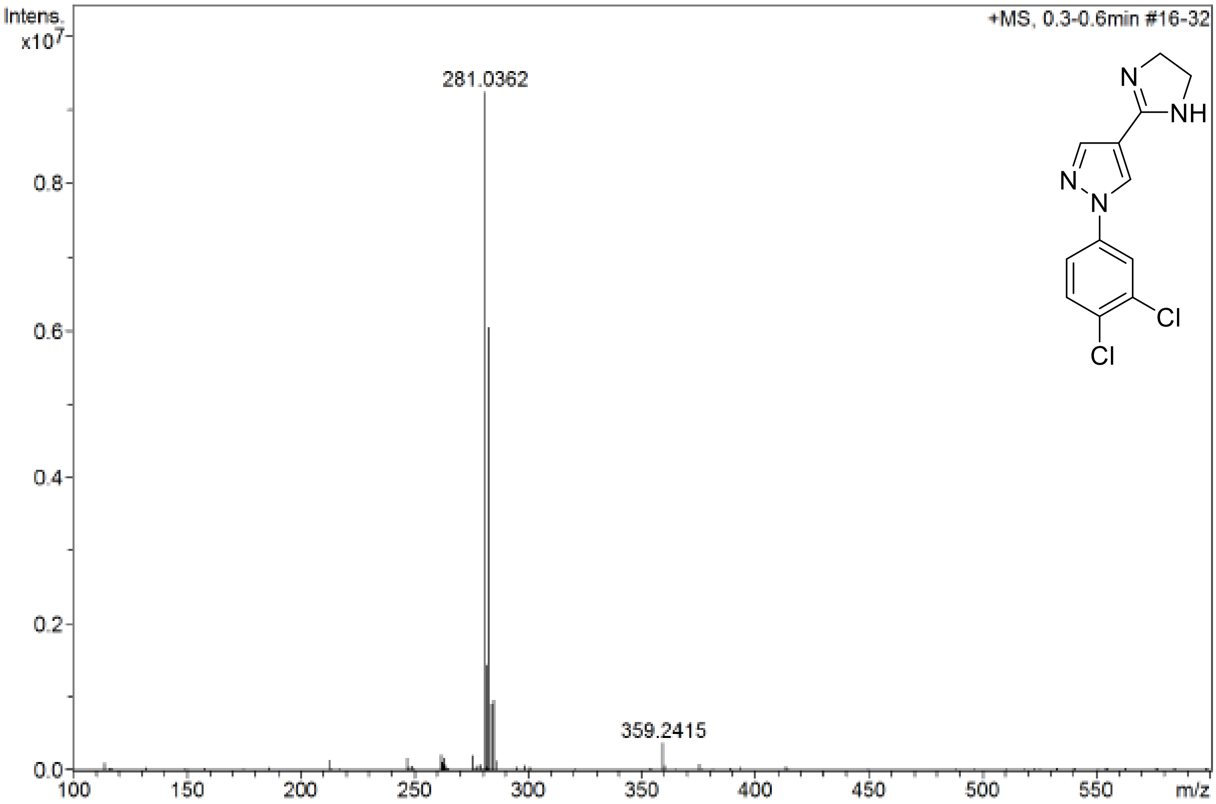

HRMS of compound **3f**

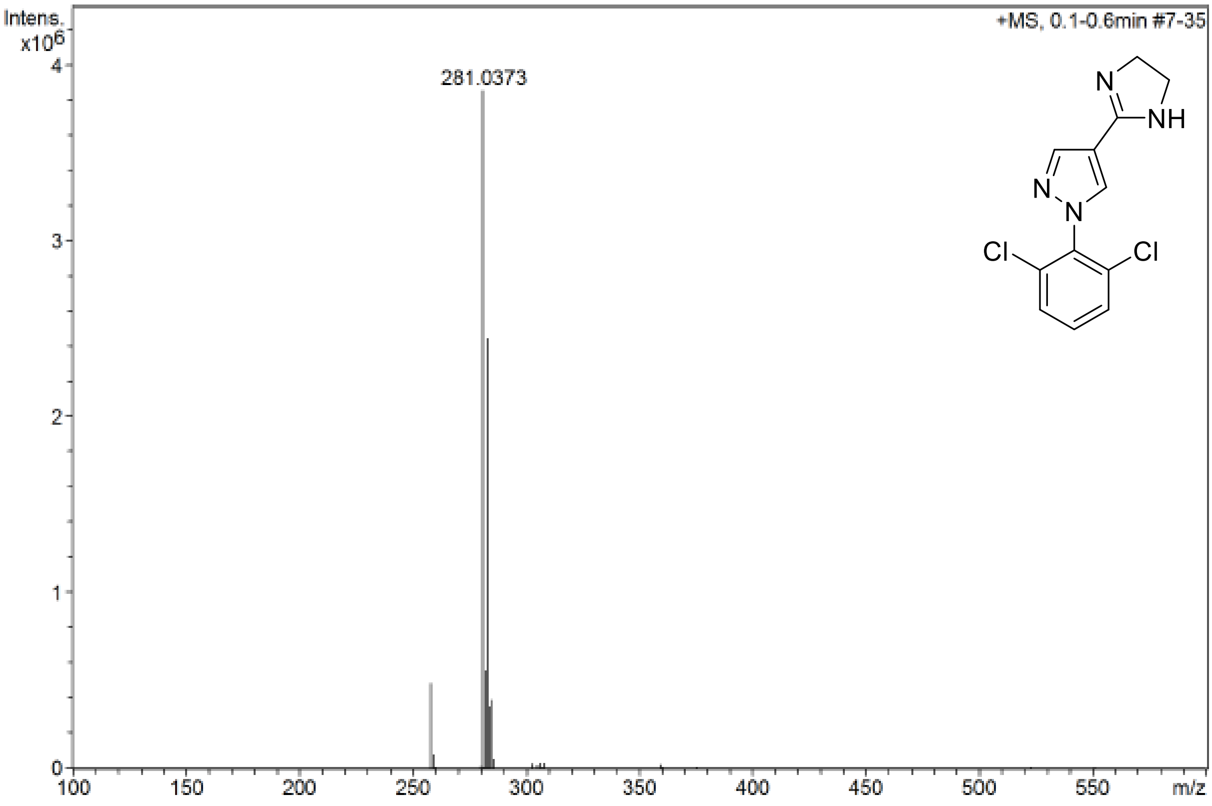

HRMS of compound **3g**

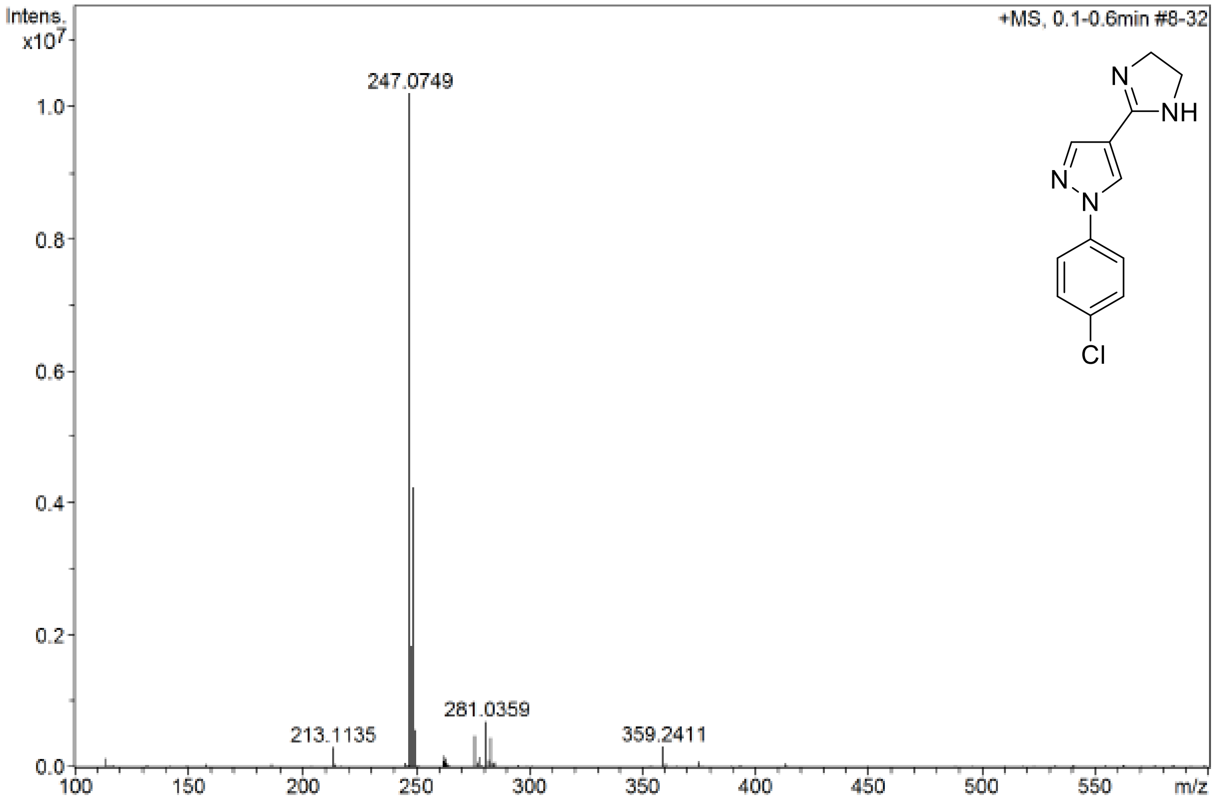

HRMS of compound **3h**

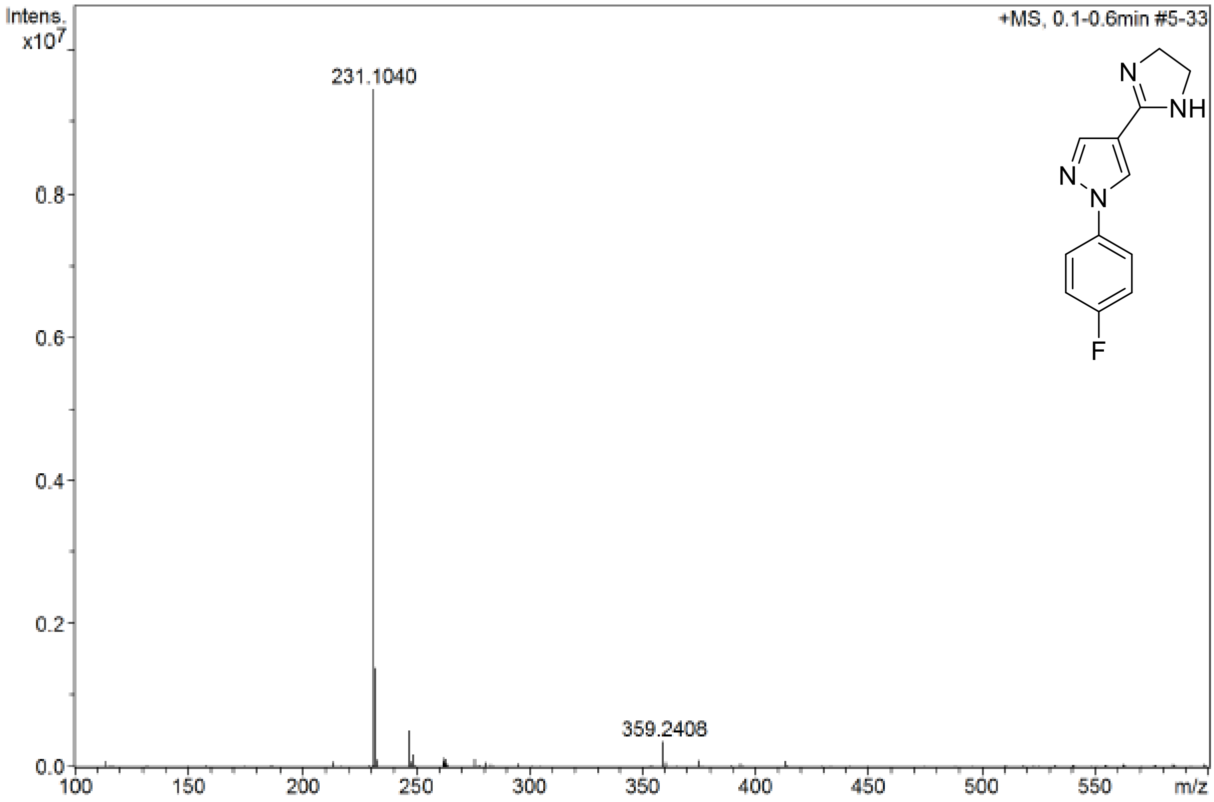

HRMS of compound **3i**

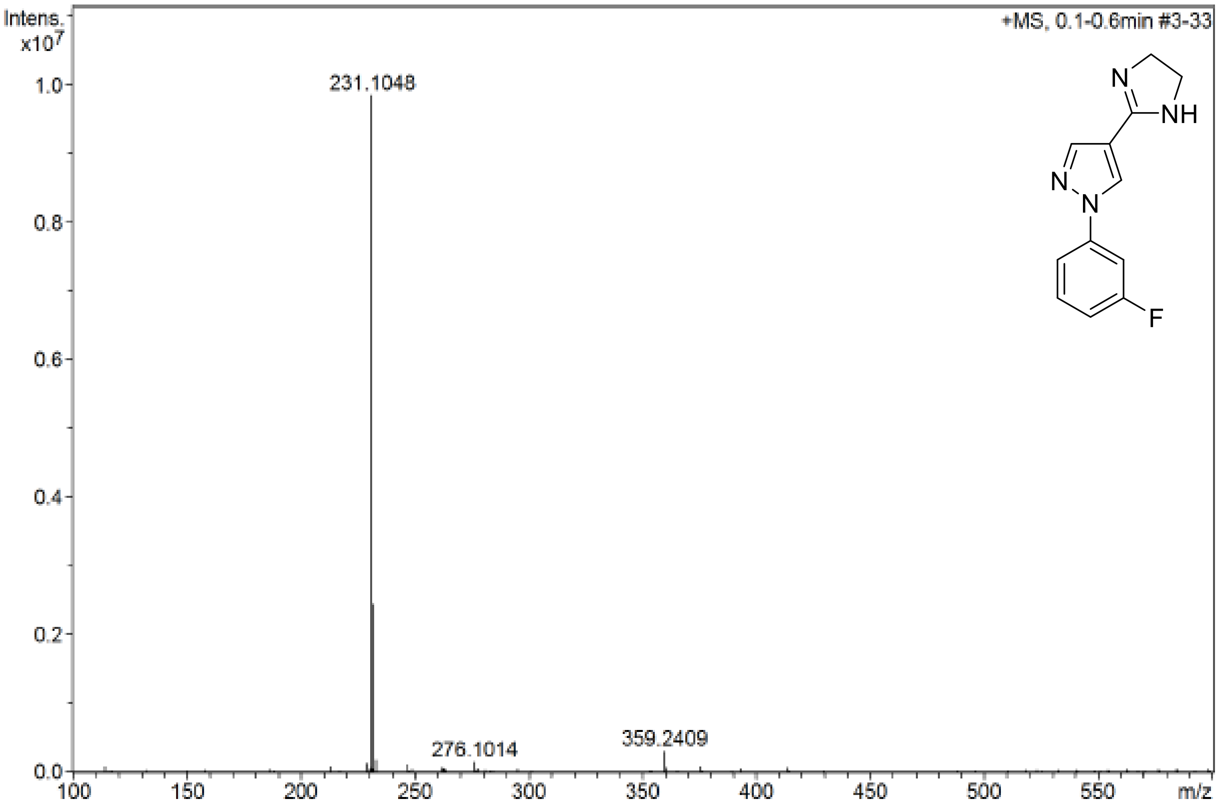

HRMS of compound **3j**

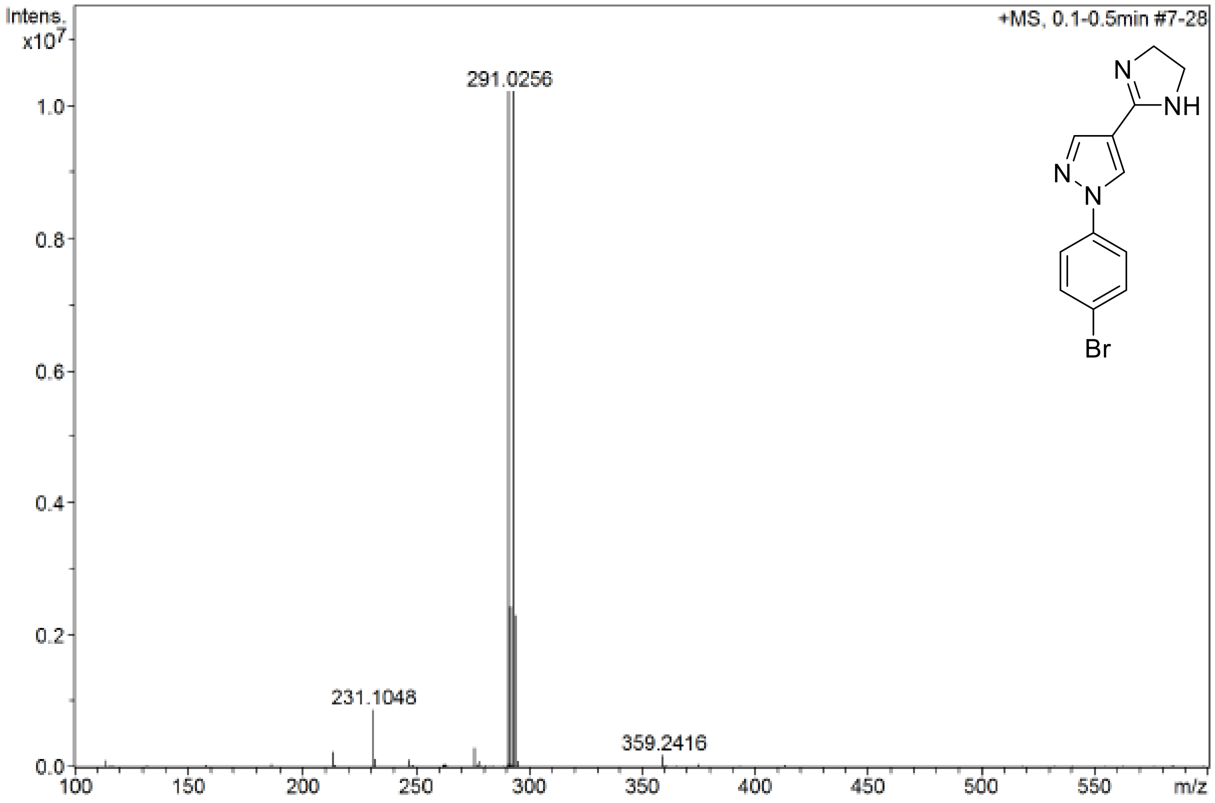

HRMS of compound **3k**

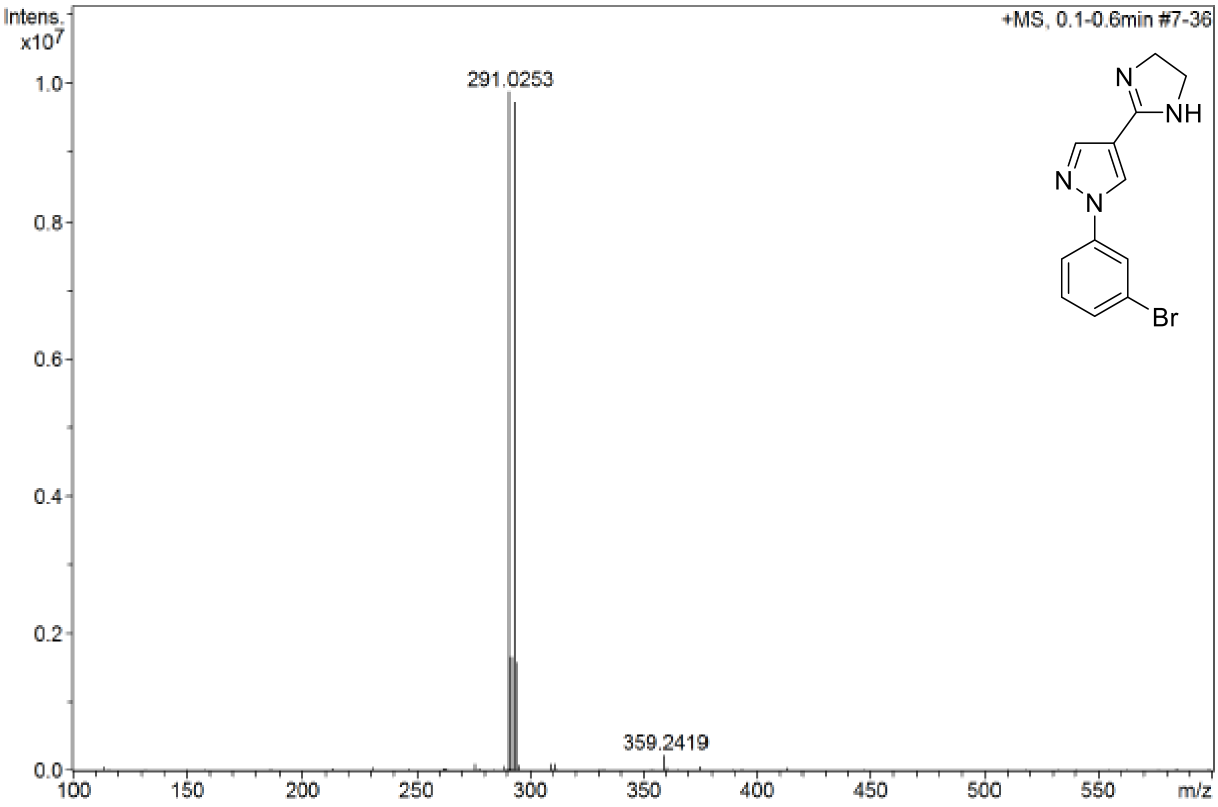

HRMS of compound **3l**

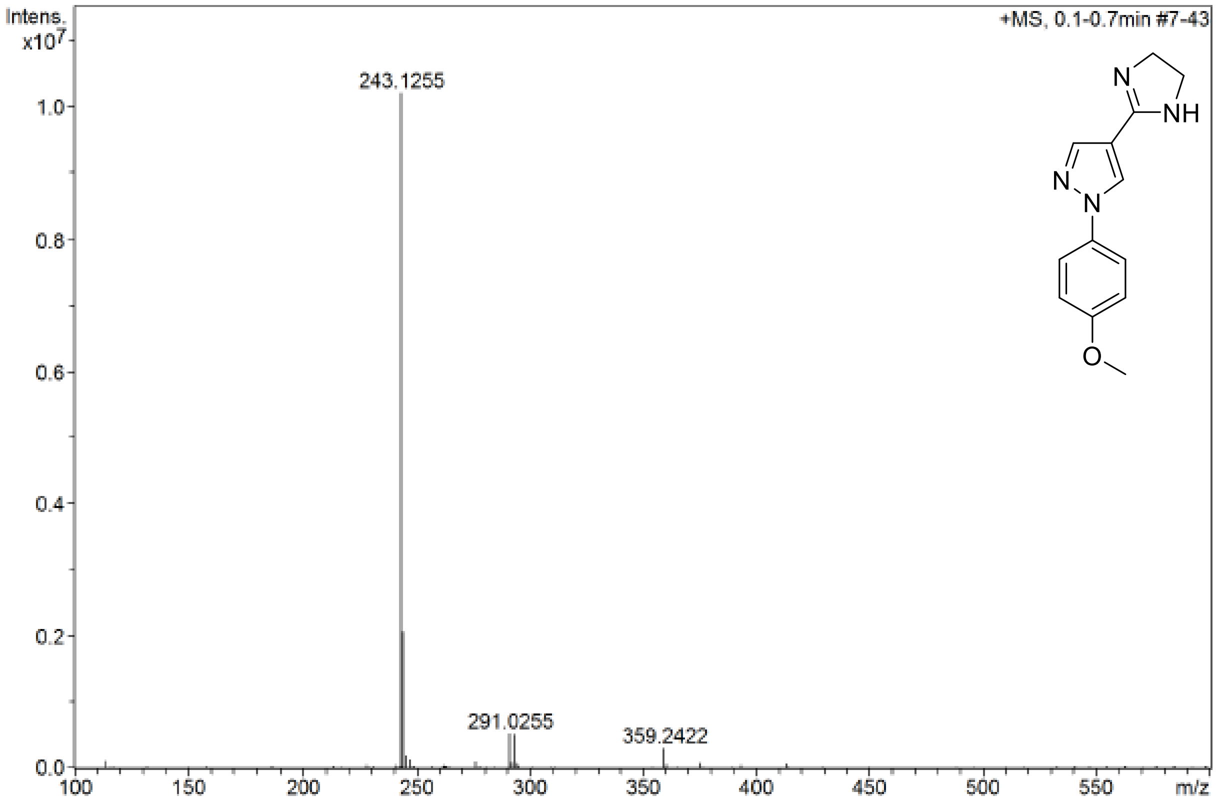

HRMS of compound **3m**

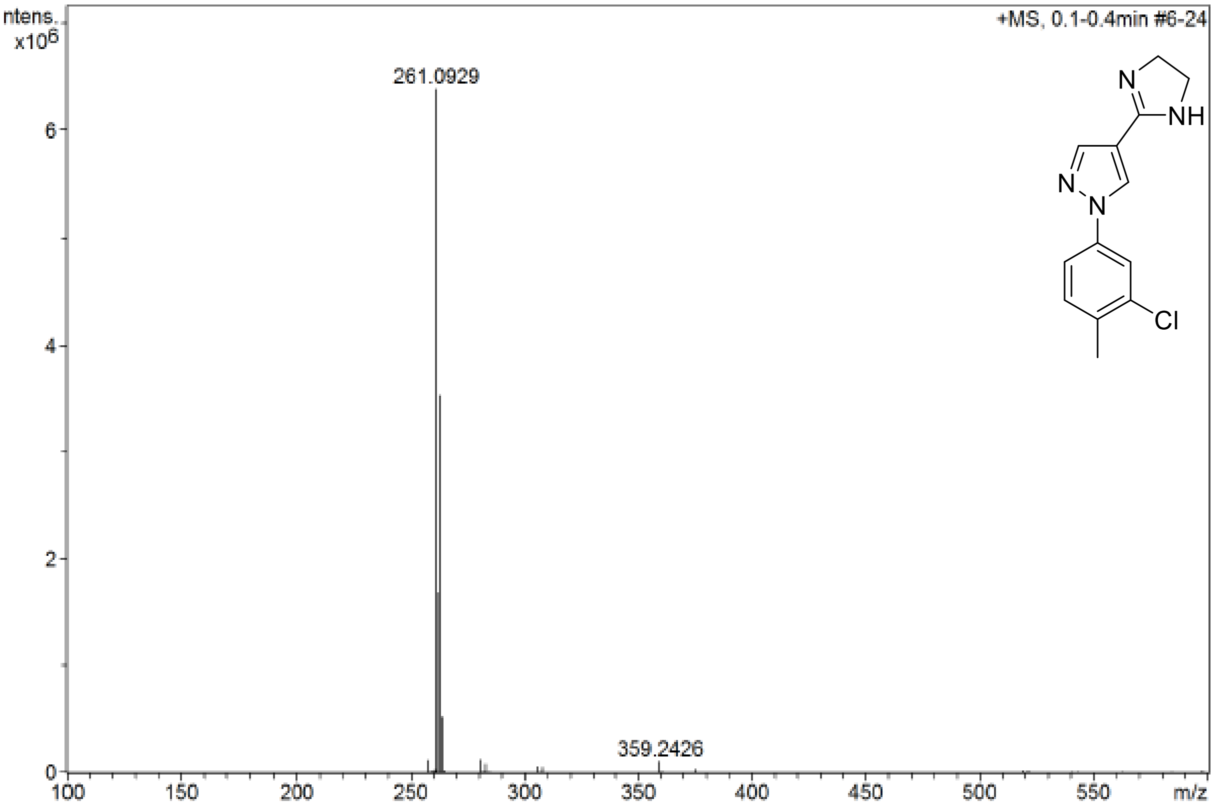

HRMS of compound **3n**

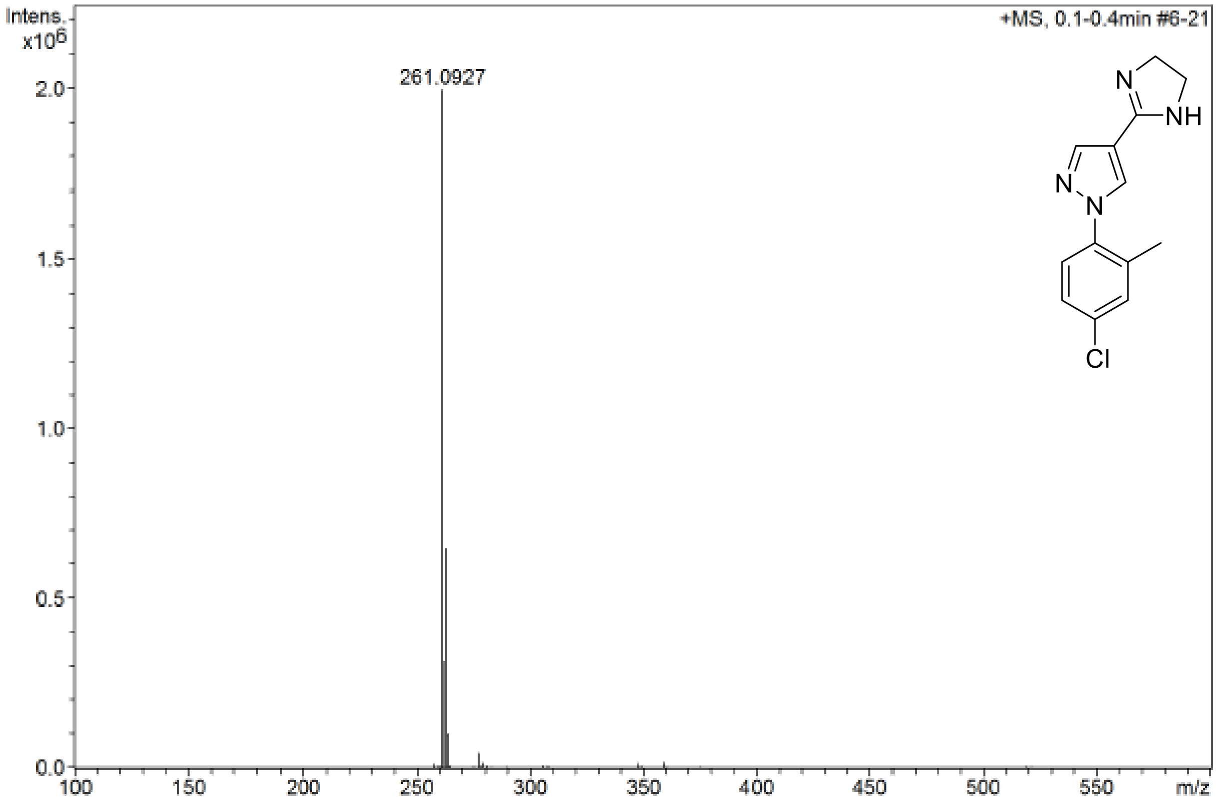

HRMS of compound **4a**

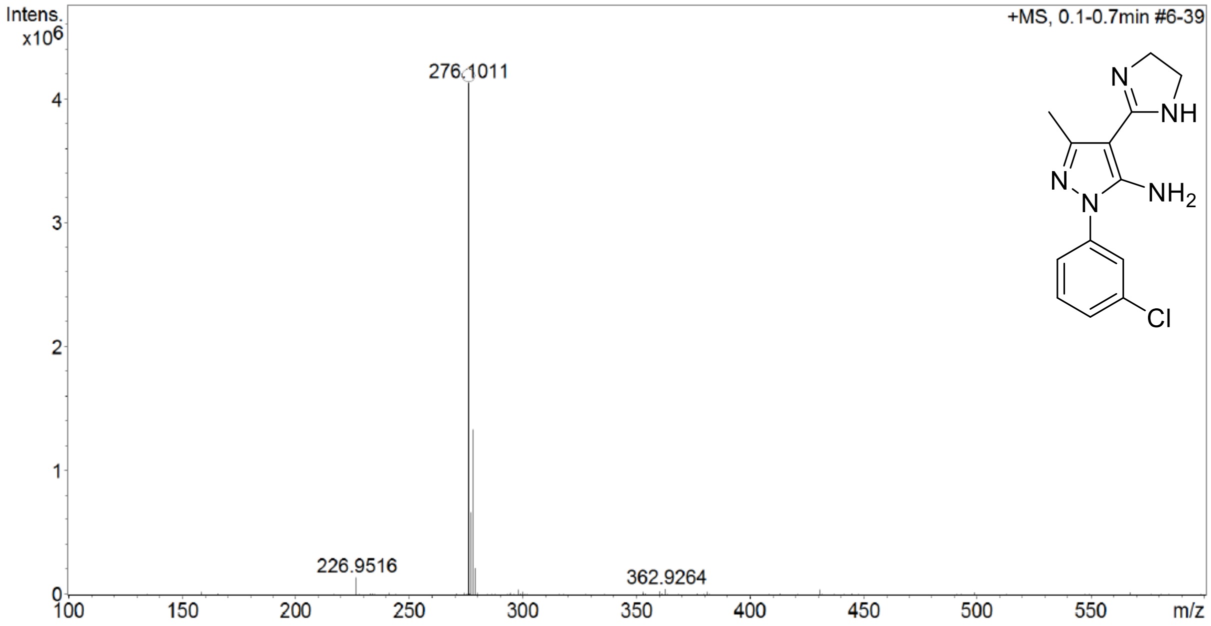

HRMS of compound **4b**

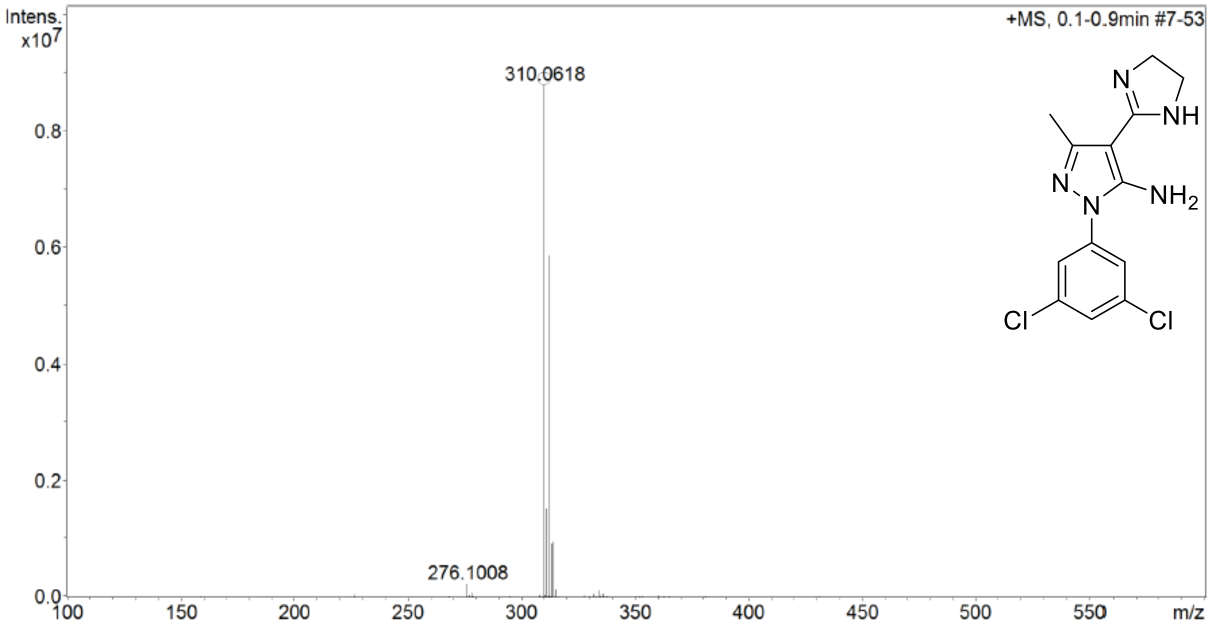

HRMS of compound **4c**

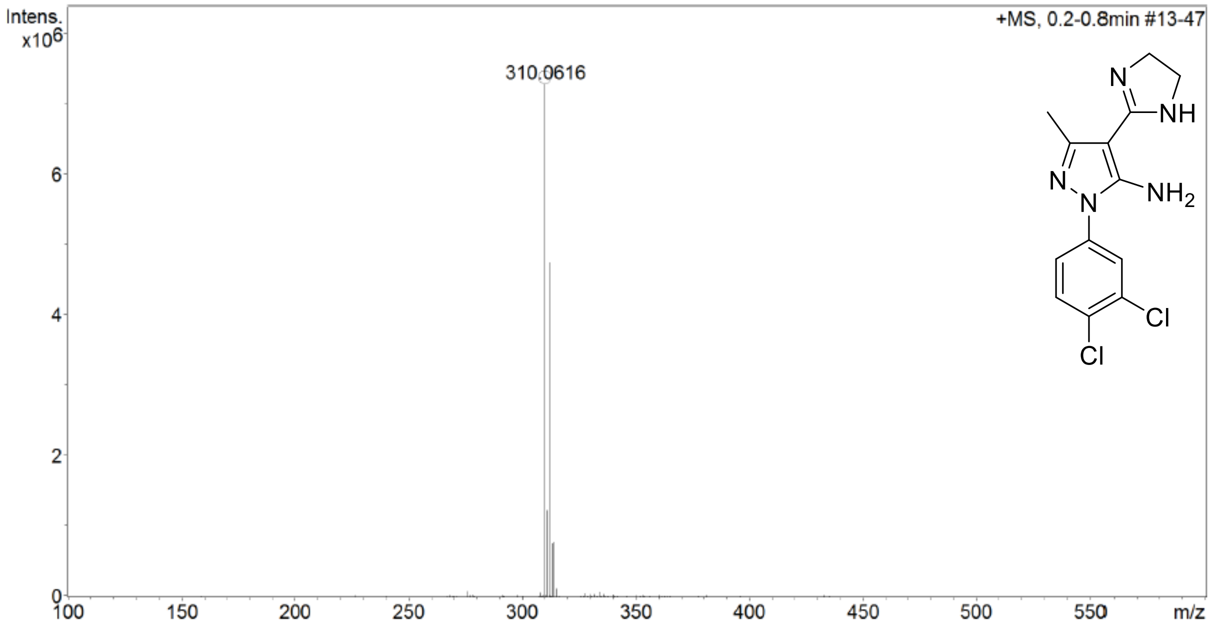

HRMS of compound **5a**

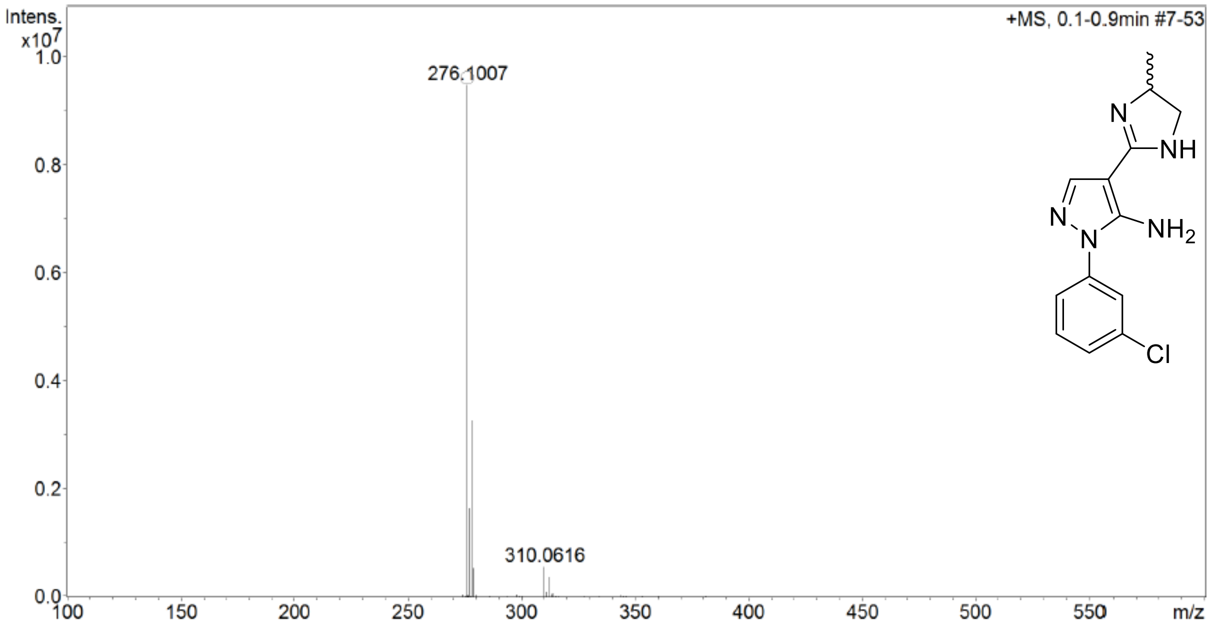

HRMS of compound **5b**

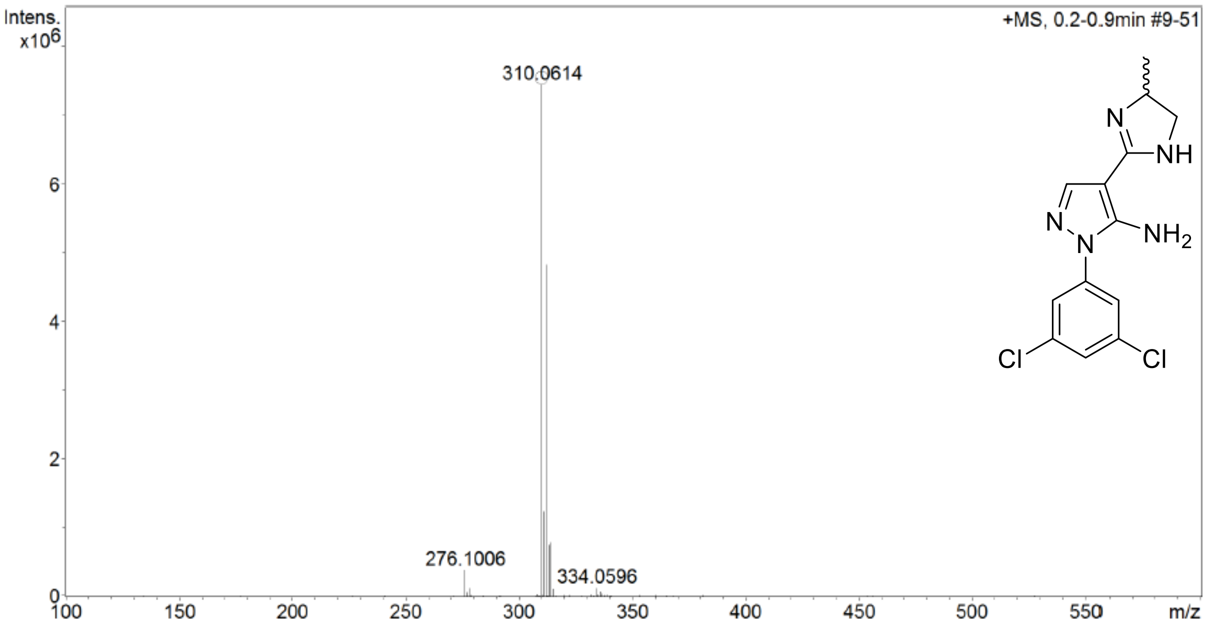

HRMS of compound **5c**

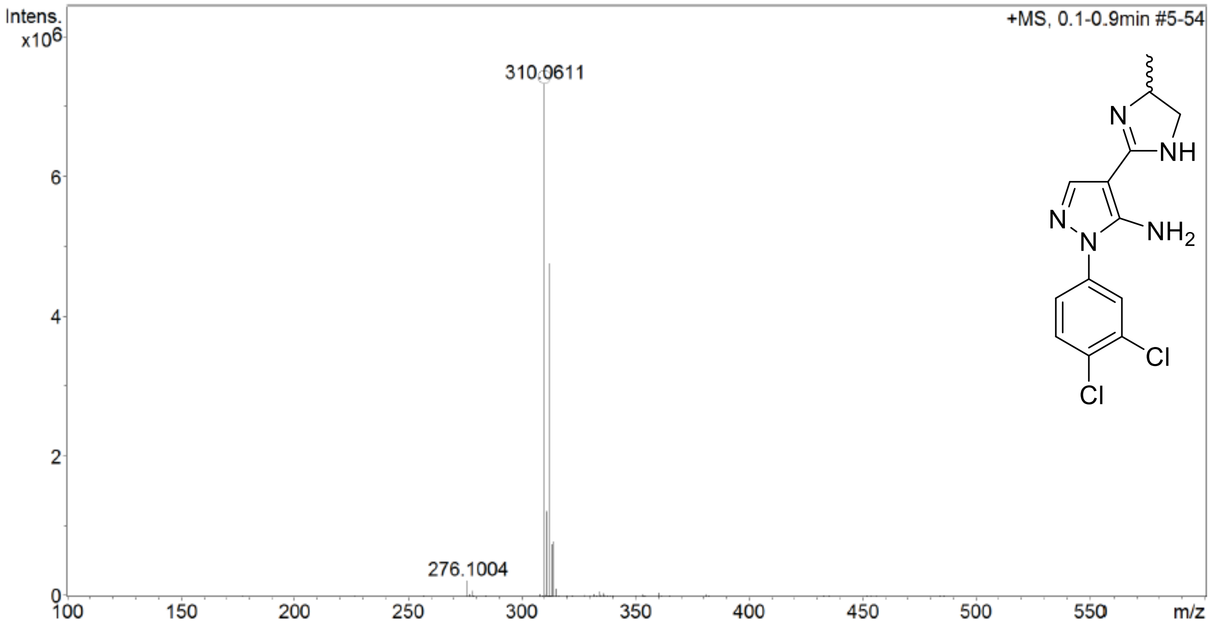

Supplement: Supplementary file 1 [file molecules-26-06742-s001.zip › Supplementary material-S5 (RMN).pdf]
